# Supplementary material for: Microbiome-based disease prediction with multimodal variational information bottlenecks
Source: PLoS Comput Biol. 2022 Apr 11;18(4):e1010050. doi: 10.1371/journal.pcbi.1010050 (PMC9022840; doi:10.1371/journal.pcbi.1010050)
Supplement: S1 Table — Results obtained optimising the JMVIB−T objective (Eq 8). Experiments are executed five times with random independent training-test splits. Values in brackets refer to the standard error over the repeated experiments. All values in the table refer to metrics computed on the test sets. ROC AUC: area under the receiver operating characteristic curve. AC: classification accuracy. F1: F1 score. P: precision. R: recall. D and J refer to the two pre-processing techniques adopted and the two collections of datasets obtained: default (D) and joint (J). (PDF) [file pcbi.1010050.s002.pdf]

S1 Table. Complete experimental results for the multimodal microbiome-based disease prediction task with MVIB.

Dataset	Metrics	MVIB	
		D	J
IBD	ROC AUC	0.922 (0.02)	0.932 (0.021)
	AC	0.827 (0.022)	0.827 (0.022)
	F1	0.8 (0.122)	0.8 (0.122)
	P	0.32 (0.049)	0.32 (0.049)
	R	0.457 (0.07)	0.457 (0.07)
EW-T2D	ROC AUC	0.859 (0.023)	0.863 (0.02)
	AC	0.76 (0.029)	0.75 (0.022)
	F1	0.8 (0.029)	0.797 (0.028)
	P	0.764 (0.062)	0.745 (0.053)
	R	0.773 (0.034)	0.763 (0.028)
C-T2D	ROC AUC	0.75 (0.009)	0.751 (0.013)
	AC	0.67 (0.018)	0.667 (0.018)
	F1	0.671 (0.009)	0.662 (0.006)
	P	0.647 (0.064)	0.659 (0.067)
	R	0.652 (0.035)	0.654 (0.037)
Obesity	ROC AUC	0.662 (0.024)	0.667 (0.026)
	AC	0.667 (0.019)	0.659 (0.016)
	F1	0.682 (0.011)	0.678 (0.009)
	P	0.909 (0.017)	0.903 (0.02)
	R	0.779 (0.012)	0.774 (0.011)
Cirrhosis	ROC AUC	0.925 (0.005)	0.925 (0.006)
	AC	0.838 (0.014)	0.838 (0.014)
	F1	0.893 (0.021)	0.893 (0.021)
	P	0.783 (0.048)	0.783 (0.048)
	R	0.829 (0.022)	0.829 (0.022)

(The table continues in the next page)

Dataset	Metrics	MVIB	
		D	J
Colorectal	ROC AUC	0.78 (0.071)	0.779 (0.073)
	AC	0.728 (0.027)	0.72 (0.022)
	F1	0.852 (0.078)	0.848 (0.078)
	P	0.42 (0.058)	0.4 (0.045)
	R	0.545 (0.051)	0.529 (0.039)
Obesity-Joint	ROC AUC	0.815 (0.019)	0.818 (0.018)
	AC	0.767 (0.022)	0.758 (0.022)
	F1	0.768 (0.019)	0.759 (0.018)
	P	0.916 (0.012)	0.916 (0.012)
	R	0.835 (0.013)	0.83 (0.014)
Colorectal-EMBL	ROC AUC	0.811 (0.01)	0.814 (0.013)
	AC	0.745 (0.024)	0.745 (0.017)
	F1	0.789 (0.042)	0.813 (0.035)
	P	0.642 (0.031)	0.611 (0.027)
	R	0.705 (0.027)	0.694 (0.019)
Early-Colorectal-EMBL	ROC AUC	0.535 (0.05)	0.543 (0.048)
	AC	0.55 (0.042)	0.56 (0.04)
	F1	0.513 (0.063)	0.527 (0.06)
	P	0.378 (0.027)	0.4 (0.027)
	R	0.434 (0.041)	0.453 (0.038)
Hypertension	ROC AUC	0.603 (0.045)	0.591 (0.041)
	AC	0.8 (0.008)	0.8 (0.008)
	F1	0.803 (0.004)	0.803 (0.004)
	P	0.994 (0.006)	0.994 (0.006)
	R	0.888 (0.004)	0.888 (0.004)

Results obtained optimising the J_{MVIB-T} objective. Experiments are executed five times with random independent training-test splits. Values in brackets refer to the standard error over the repeated experiments. All values in the table refer to metrics computed on the test sets. ROC AUC: area under the receiver operating characteristic curve. AC: classification accuracy. F1: F1 score. P: precision. R: recall. D and J refer to the two pre-processing techniques adopted and the two collections of datasets obtained: *default* (D) and *joint* (J).
