# Supplementary material for: Microbiome-based disease prediction with multimodal variational information bottlenecks
Source: PLoS Comput Biol. 2022 Apr 11;18(4):e1010050. doi: 10.1371/journal.pcbi.1010050 (PMC9022840; doi:10.1371/journal.pcbi.1010050)
Supplement: S2 Table — All values are ROC AUC computed on the test sets. Values in brackets refer to the standard error over five repeated experiments. The first group of columns presents the results obtained optimising the JMVIB−T objective (Eq 8), which includes the triplet margin loss. The second group of columns presents the results obtained optimising the original objective function JMVIB (Eq 5). D and J refer to the two pre-processing techniques adopted and the two collections of datasets obtained: default (D) and joint (J). (PDF) [file pcbi.1010050.s003.pdf]

S2 Table. Comparison of different objective functions and pre-processing techniques.

Dataset	J_{MVIB-T}		J_{MVIB}	
	D	J	D	J
IBD	0.922 (0.020)	**0.936 (0.014)**	0.915 (0.018)	0.915 (0.018)
EW-T2D	**0.859 (0.023)**	0.853 (0.025)	0.859 (0.024)	0.855 (0.027)
C-T2D	0.750 (0.009)	**0.758 (0.012)**	0.754 (0.014)	0.756 (0.015)
Obesity	0.662 (0.024)	0.666 (0.027)	**0.673 (0.028)**	0.672 (0.030)
Cirrhosis	0.925 (0.005)	0.924 (0.005)	**0.930 (0.002)**	0.928 (0.002)
Colorectal	0.780 (0.071)	0.777 (0.069)	0.788 (0.059)	**0.796 (0.055)**
Obesity-Joint	0.815 (0.019)	0.818 (0.018)	0.825 (0.019)	**0.825 (0.018)**
Colorectal-EMBL	0.811 (0.010)	0.814 (0.013)	0.827 (0.012)	**0.830 (0.011)**
Early-Colorectal-EMBL	0.535 (0.050)	**0.543 (0.048)**	0.525 (0.051)	0.533 (0.053)
Hypertension	0.602 (0.045)	0.608 (0.043)	0.622 (0.048)	**0.624 (0.049)**
