# Supplementary material for: Microbiome-based disease prediction with multimodal variational information bottlenecks
Source: PLoS Comput Biol. 2022 Apr 11;18(4):e1010050. doi: 10.1371/journal.pcbi.1010050 (PMC9022840; doi:10.1371/journal.pcbi.1010050)
Supplement: S3 Table — The first column presents the results obtained with randomly initialised models. The second column displays classification results obtained by first pre-training the models on all source datasets, and then fine-tuning them on the target disease (see Section Transfer learning). The JMVIB−T objective was adopted for both columns (Eq 8). J refers to the adopted pre-processing technique: joint. Reported values are ROC AUC computed on the test sets. In brackets, the standard error over five repeated experiments is reported. (PDF) [file pcbi.1010050.s004.pdf]

S3 Table. Comparison of pre-trained models against randomly initialised models.

Dataset	Random initialisation	Pre-trained model
	J	J
IBD	**0.936 (0.014)**	0.882 (0.021)
EW-T2D	**0.853 (0.025)**	0.780 (0.024)
C-T2D	0.758 (0.012)	**0.774 (0.013)**
Obesity	0.666 (0.027)	**0.679 (0.024)**
Cirrhosis	**0.924 (0.005)**	0.918 (0.007)
Colorectal	**0.777 (0.069)**	0.763 (0.068)
Hypertension	0.591 (0.041)	**0.645 (0.047)**
