# Supplementary material for: Microbiome-based disease prediction with multimodal variational information bottlenecks
Source: PLoS Comput Biol. 2022 Apr 11;18(4):e1010050. doi: 10.1371/journal.pcbi.1010050 (PMC9022840; doi:10.1371/journal.pcbi.1010050)
Supplement: S4 Table — Experiments are executed five times with random independent training-test splits. Values in brackets refer to the standard error over the repeated experiments. Reported values are test ROC AUC. (PDF) [file pcbi.1010050.s005.pdf]

S4 Table. Experimental results for the Random Forest with default Scikit-learn implementation

	**Random Forest - Default Scikit-learn implementation (RF-DEF)**		
**Dataset**	**A**	**M**	**A+M**
IBD	0.878 (0.038)	0.900 (0.018)	0.878 (0.038)
EW-T2D	0.796 (0.027)	0.791 (0.041)	0.796 (0.027)
C-T2D	0.709 (0.022)	0.743 (0.017)	0.709 (0.022)
Obesity	0.641 (0.026)	0.567 (0.016)	0.641 (0.026)
Cirrhosis	0.890 (0.011)	0.894 (0.012)	0.890 (0.011)
Colorectal	0.835 (0.038)	0.785 (0.043)	0.835 (0.038)
Obesity-joint	0.798 (0.018)	0.768 (0.030)	0.798 (0.018)
Colorectal-EMBL	0.866 (0.015)	0.822 (0.024)	0.866 (0.015)
Early-Colorectal-EMBL	0.517 (0.048)	0.521 (0.015)	0.517 (0.048)
Hypertension	0.652 (0.043)	0.653 (0.025)	0.652 (0.043)

Experiments are executed five times with random independent training-test splits. Values in brackets refer to the standard error over the repeated experiments. Reported values are test ROC AUC.
