# Supplementary figures and images for: Microbiome-based disease prediction with multimodal variational information bottlenecks (part 3 of 4)
Source: PLoS Comput Biol. 2022 Apr 11;18(4):e1010050. doi: 10.1371/journal.pcbi.1010050 (PMC9022840; doi:10.1371/journal.pcbi.1010050)

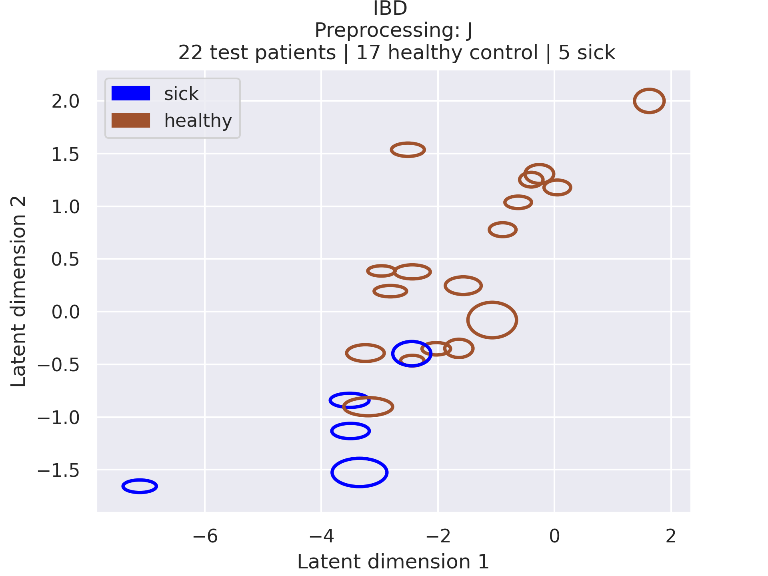

Supplement: S2 File — For all datasets considered in this work, this file presents plots of the 2D MVIB stochastic encodings analogous to Fig 3. The depicted curves are the 95% confidence intervals of the samples’ stochastic encodings z∼p(z|x)=N(μ,σ2I); the points are their means μ. The displayed encodings consist only in the test samples obtained from random training-test splits (i.e. the 20% of the dataset not used for training). The K dimension of the latent space has been set to 2 in order to allow a 2D visualisation. Plots derived from both the optimisation of the JMVIB−T objective (Eq 8) and the optimisation of the JMVIB objective (Eq 5) are included. Five copies of all plots are available, as they are obtained by training the model with five different independent training-test random splits. (ZIP) [file pcbi.1010050.s007.zip › s5-file/bce-only/J/IBD/4_embeddings_95_confidence.png]

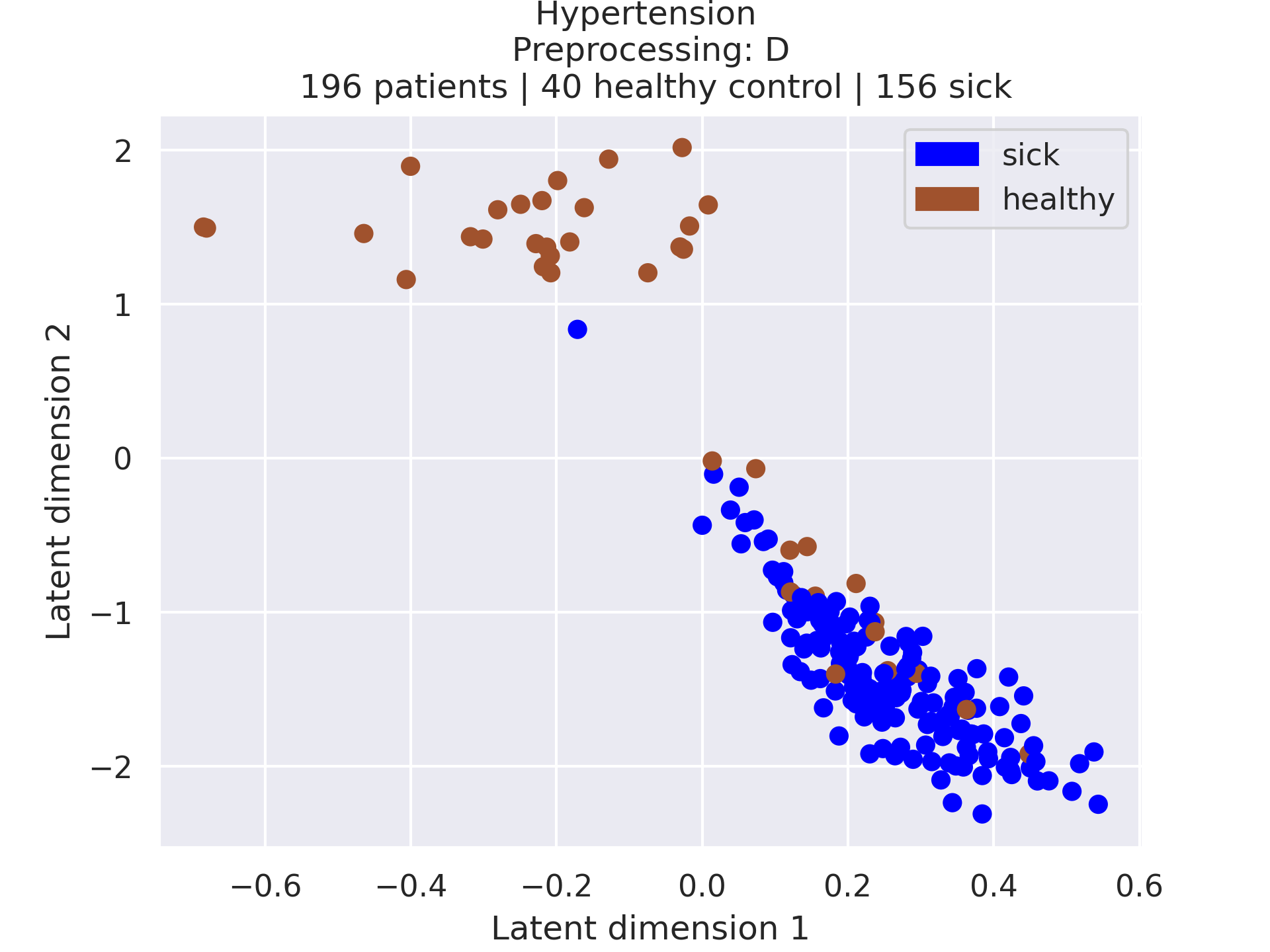

Supplement: S3 File — This file presents, for each dataset, the plots of the PCA 2D projections, as well as the plots of the mean of the MVIB 2D stochastic encodings. For the MVIB stochastic encodings z∼p(z|x)=N(μ,σ2I), the depicted points represent the mean μ. The K dimension of the latent space has been set to 2 in order to allow a 2D visualisation of the encodings. For training MVIB, the JMVIB−T objective (Eq 8) has been optimised. For MVIB, five copies of the means plots are available, as they are obtained by training the model with five different independent training-test random splits. Both the PCA and the MVIB plots have been created starting from the default datasets collection. (ZIP) [file pcbi.1010050.s008.zip › s6-file/Hypertension/3_embeddings.png]

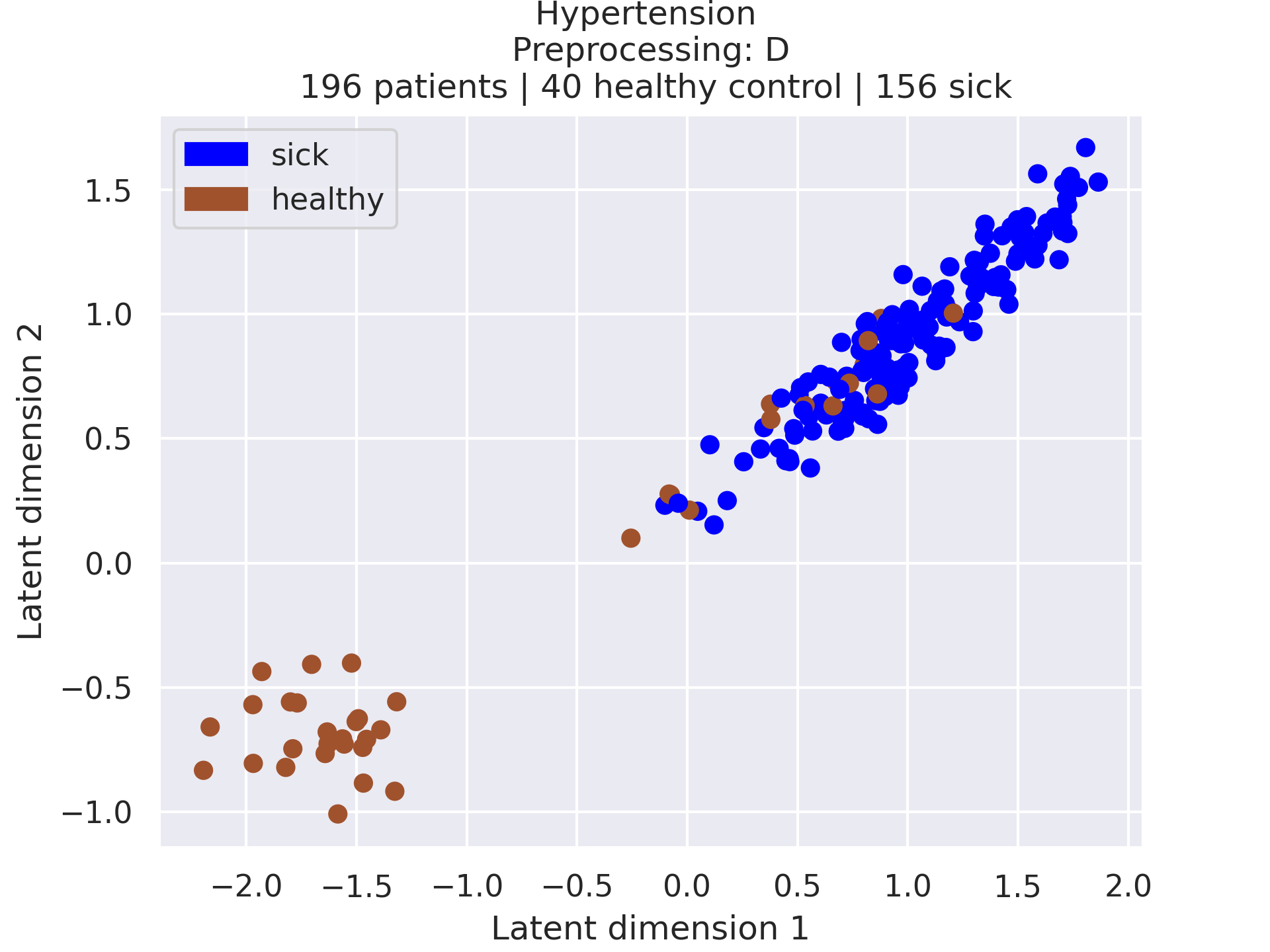

Supplement: S3 File — This file presents, for each dataset, the plots of the PCA 2D projections, as well as the plots of the mean of the MVIB 2D stochastic encodings. For the MVIB stochastic encodings z∼p(z|x)=N(μ,σ2I), the depicted points represent the mean μ. The K dimension of the latent space has been set to 2 in order to allow a 2D visualisation of the encodings. For training MVIB, the JMVIB−T objective (Eq 8) has been optimised. For MVIB, five copies of the means plots are available, as they are obtained by training the model with five different independent training-test random splits. Both the PCA and the MVIB plots have been created starting from the default datasets collection. (ZIP) [file pcbi.1010050.s008.zip › s6-file/Hypertension/4_embeddings.png]

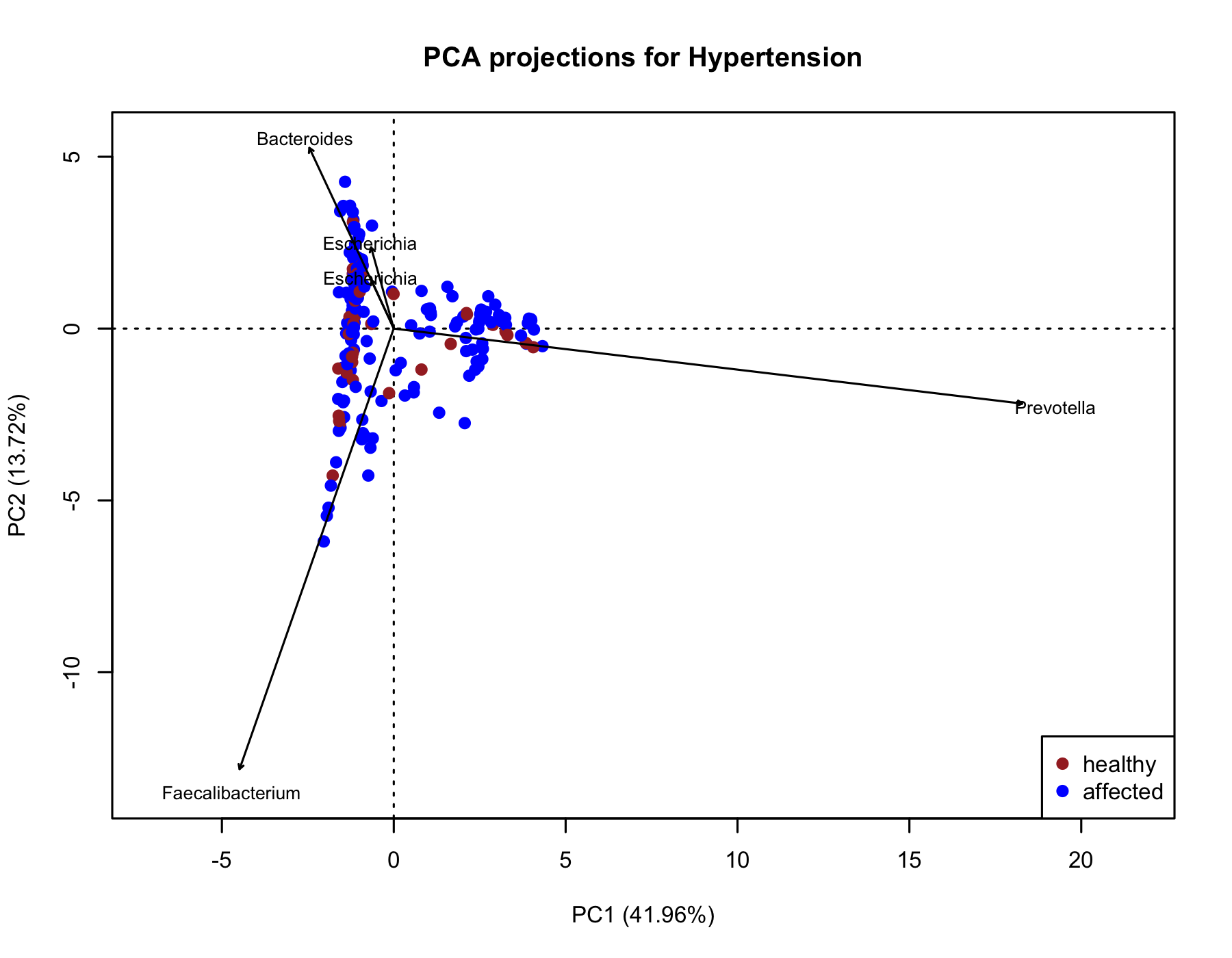

Supplement: S3 File — This file presents, for each dataset, the plots of the PCA 2D projections, as well as the plots of the mean of the MVIB 2D stochastic encodings. For the MVIB stochastic encodings z∼p(z|x)=N(μ,σ2I), the depicted points represent the mean μ. The K dimension of the latent space has been set to 2 in order to allow a 2D visualisation of the encodings. For training MVIB, the JMVIB−T objective (Eq 8) has been optimised. For MVIB, five copies of the means plots are available, as they are obtained by training the model with five different independent training-test random splits. Both the PCA and the MVIB plots have been created starting from the default datasets collection. (ZIP) [file pcbi.1010050.s008.zip › s6-file/Hypertension/PCA_projections.png]

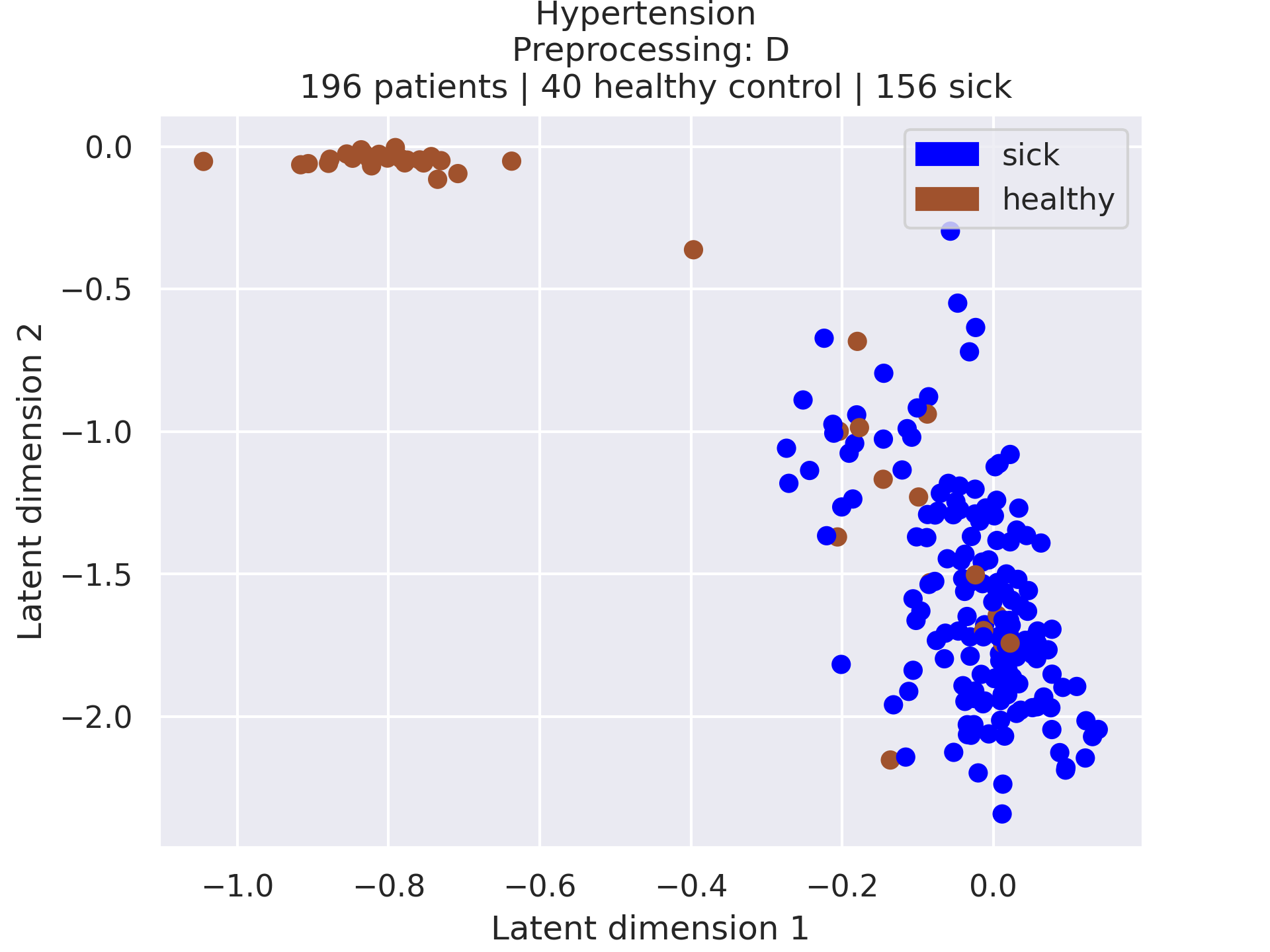

Supplement: S3 File — This file presents, for each dataset, the plots of the PCA 2D projections, as well as the plots of the mean of the MVIB 2D stochastic encodings. For the MVIB stochastic encodings z∼p(z|x)=N(μ,σ2I), the depicted points represent the mean μ. The K dimension of the latent space has been set to 2 in order to allow a 2D visualisation of the encodings. For training MVIB, the JMVIB−T objective (Eq 8) has been optimised. For MVIB, five copies of the means plots are available, as they are obtained by training the model with five different independent training-test random splits. Both the PCA and the MVIB plots have been created starting from the default datasets collection. (ZIP) [file pcbi.1010050.s008.zip › s6-file/Hypertension/2_embeddings.png]

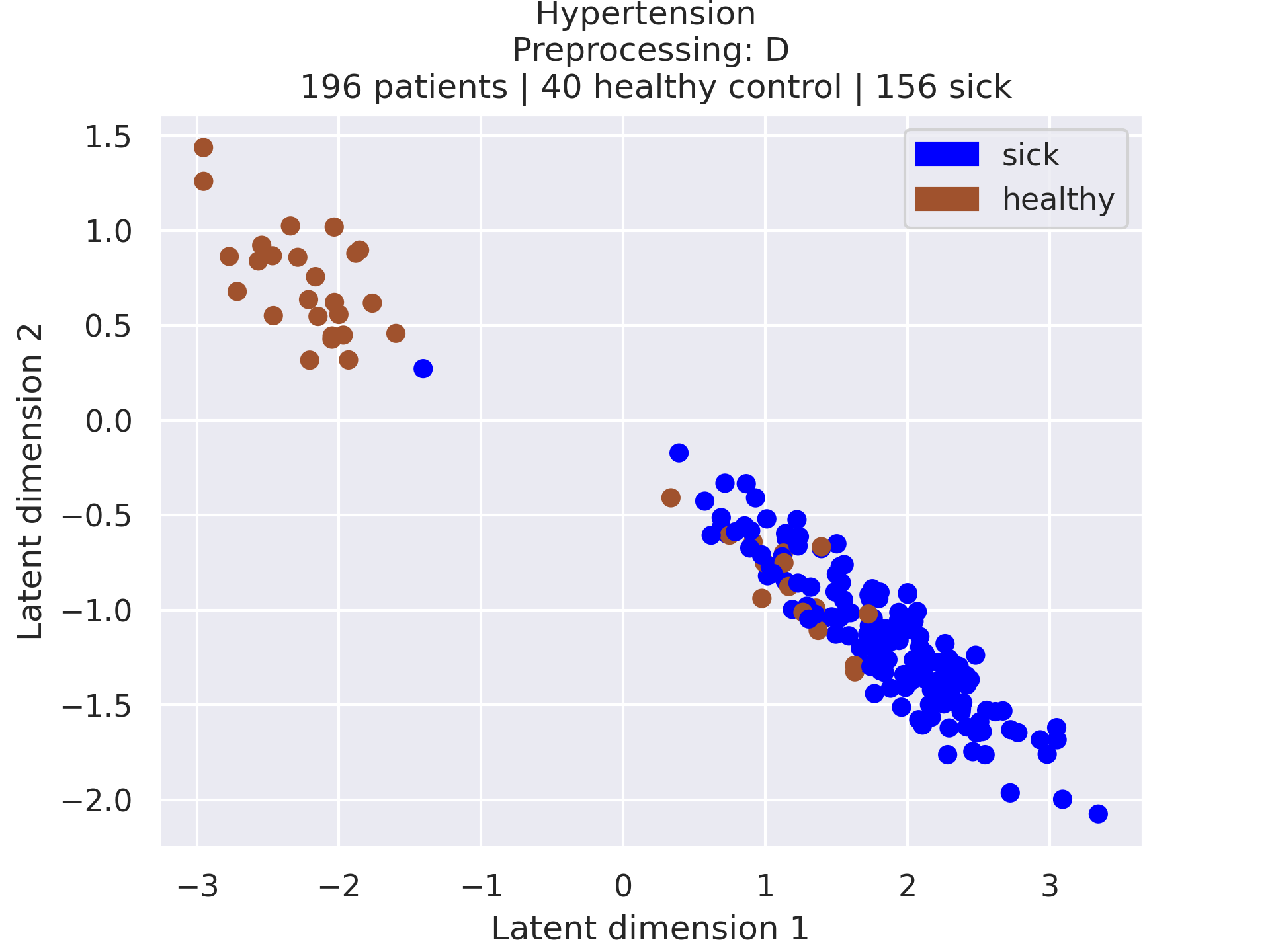

Supplement: S3 File — This file presents, for each dataset, the plots of the PCA 2D projections, as well as the plots of the mean of the MVIB 2D stochastic encodings. For the MVIB stochastic encodings z∼p(z|x)=N(μ,σ2I), the depicted points represent the mean μ. The K dimension of the latent space has been set to 2 in order to allow a 2D visualisation of the encodings. For training MVIB, the JMVIB−T objective (Eq 8) has been optimised. For MVIB, five copies of the means plots are available, as they are obtained by training the model with five different independent training-test random splits. Both the PCA and the MVIB plots have been created starting from the default datasets collection. (ZIP) [file pcbi.1010050.s008.zip › s6-file/Hypertension/0_embeddings.png]

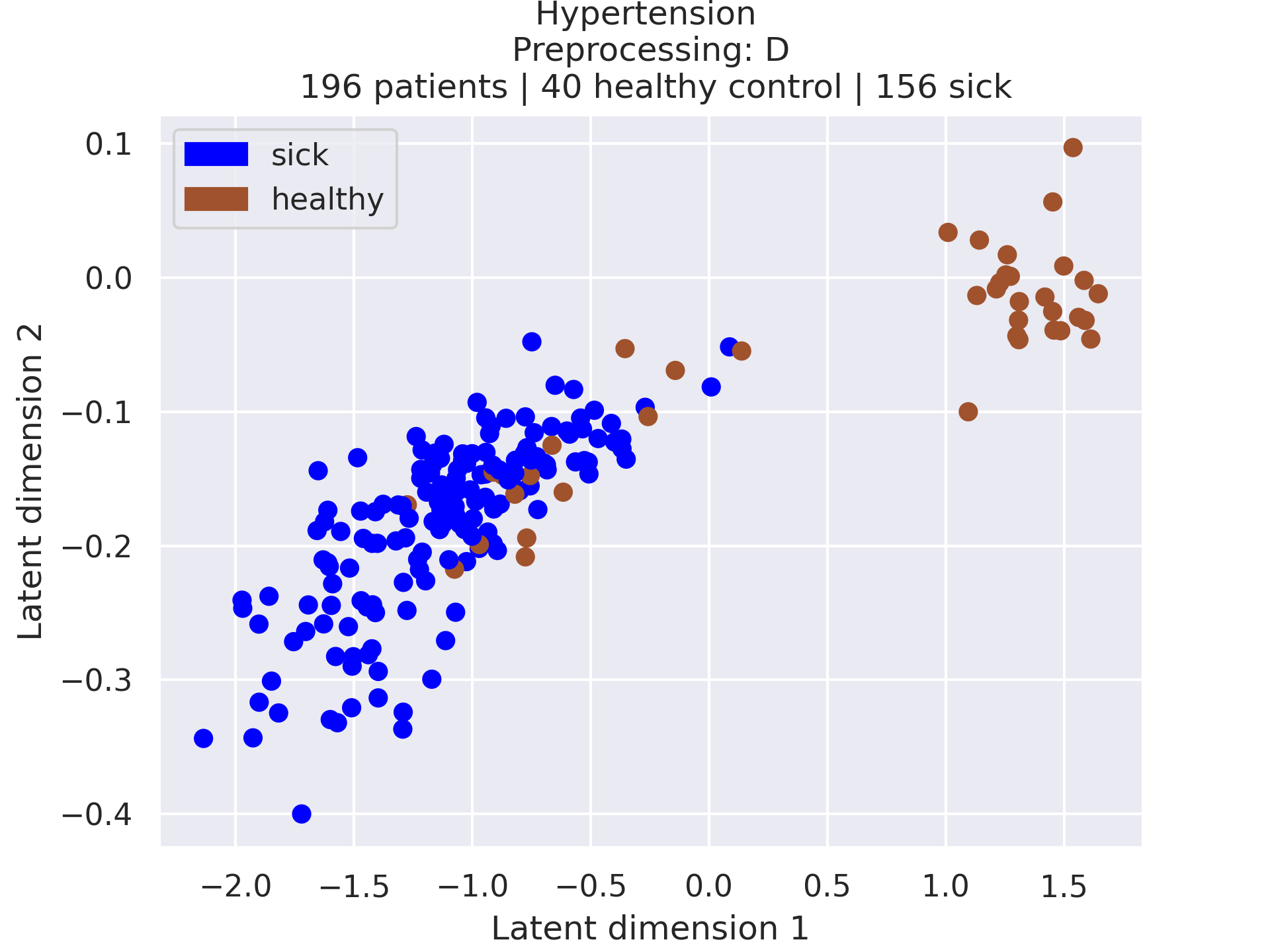

Supplement: S3 File — This file presents, for each dataset, the plots of the PCA 2D projections, as well as the plots of the mean of the MVIB 2D stochastic encodings. For the MVIB stochastic encodings z∼p(z|x)=N(μ,σ2I), the depicted points represent the mean μ. The K dimension of the latent space has been set to 2 in order to allow a 2D visualisation of the encodings. For training MVIB, the JMVIB−T objective (Eq 8) has been optimised. For MVIB, five copies of the means plots are available, as they are obtained by training the model with five different independent training-test random splits. Both the PCA and the MVIB plots have been created starting from the default datasets collection. (ZIP) [file pcbi.1010050.s008.zip › s6-file/Hypertension/1_embeddings.png]

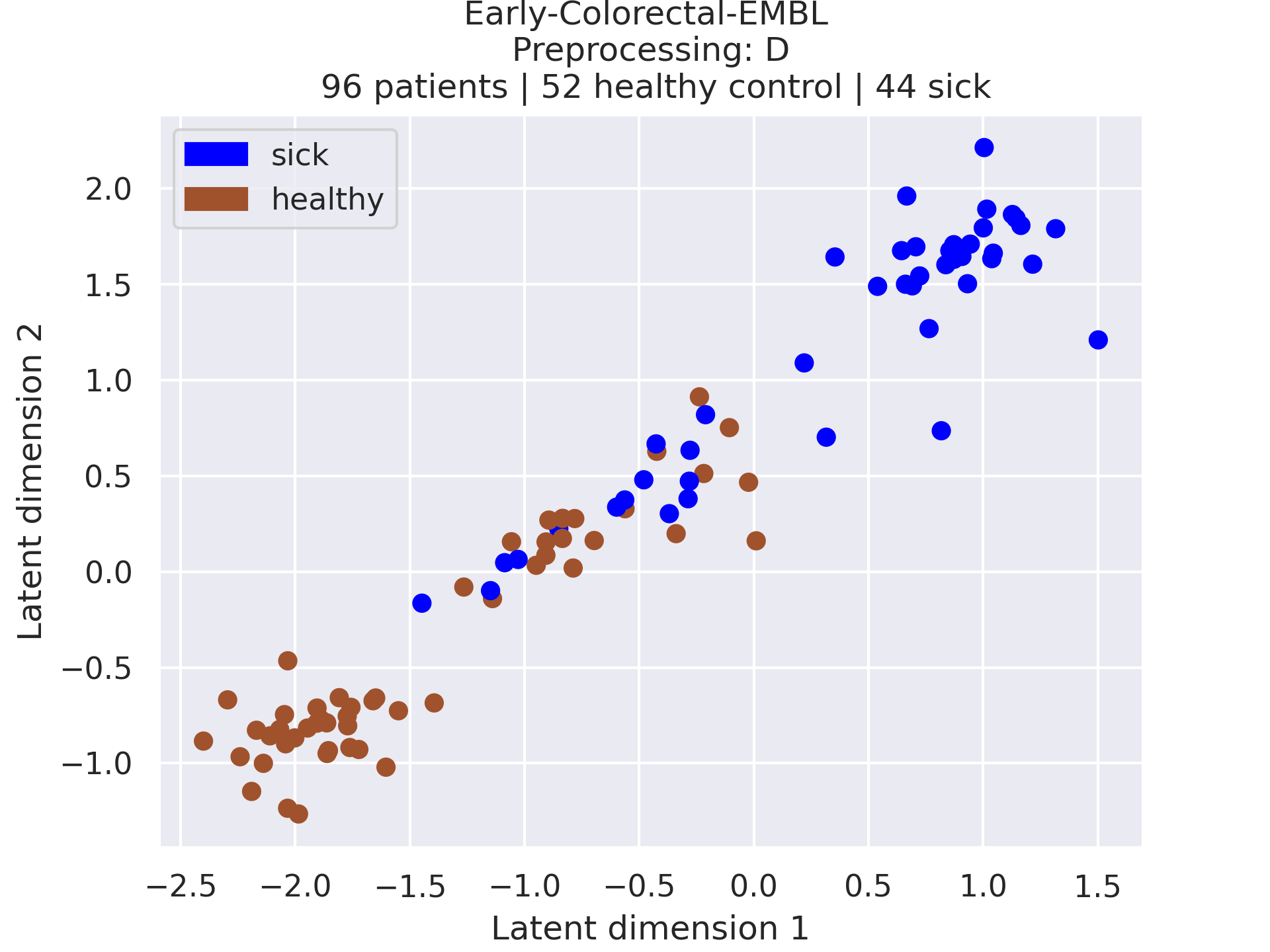

Supplement: S3 File — This file presents, for each dataset, the plots of the PCA 2D projections, as well as the plots of the mean of the MVIB 2D stochastic encodings. For the MVIB stochastic encodings z∼p(z|x)=N(μ,σ2I), the depicted points represent the mean μ. The K dimension of the latent space has been set to 2 in order to allow a 2D visualisation of the encodings. For training MVIB, the JMVIB−T objective (Eq 8) has been optimised. For MVIB, five copies of the means plots are available, as they are obtained by training the model with five different independent training-test random splits. Both the PCA and the MVIB plots have been created starting from the default datasets collection. (ZIP) [file pcbi.1010050.s008.zip › s6-file/Early-Colorectal-EMBL/3_embeddings.png]

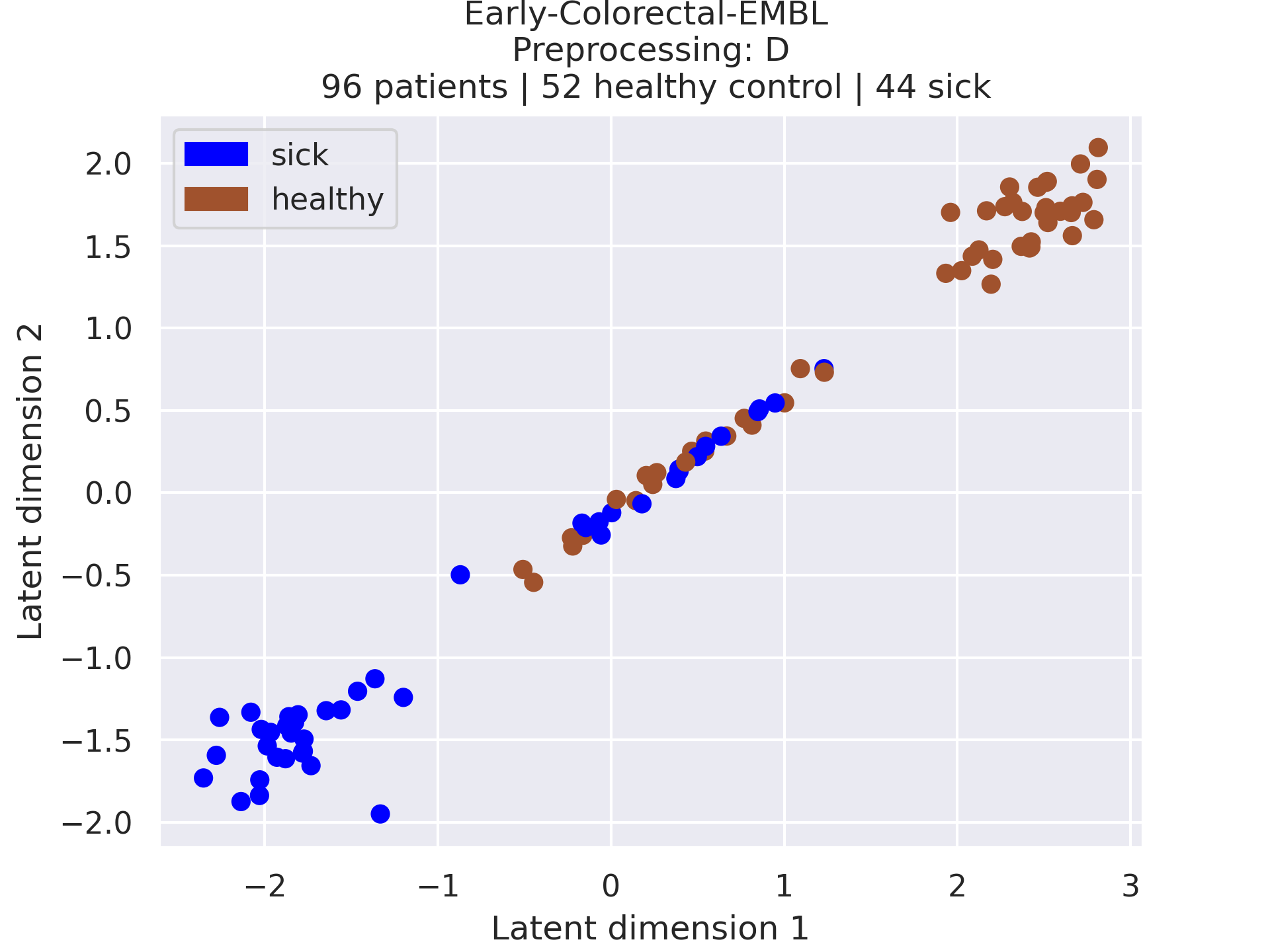

Supplement: S3 File — This file presents, for each dataset, the plots of the PCA 2D projections, as well as the plots of the mean of the MVIB 2D stochastic encodings. For the MVIB stochastic encodings z∼p(z|x)=N(μ,σ2I), the depicted points represent the mean μ. The K dimension of the latent space has been set to 2 in order to allow a 2D visualisation of the encodings. For training MVIB, the JMVIB−T objective (Eq 8) has been optimised. For MVIB, five copies of the means plots are available, as they are obtained by training the model with five different independent training-test random splits. Both the PCA and the MVIB plots have been created starting from the default datasets collection. (ZIP) [file pcbi.1010050.s008.zip › s6-file/Early-Colorectal-EMBL/4_embeddings.png]

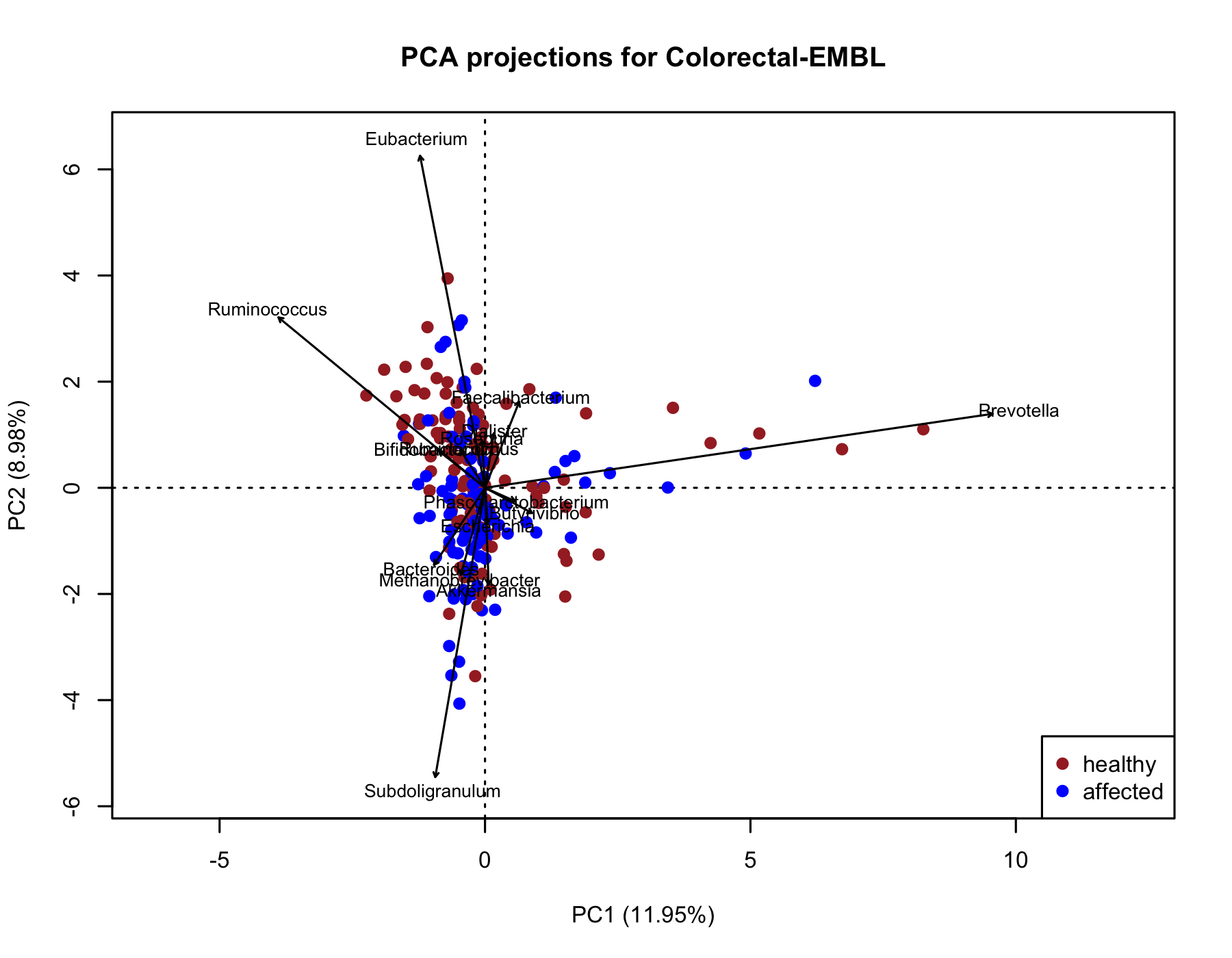

Supplement: S3 File — This file presents, for each dataset, the plots of the PCA 2D projections, as well as the plots of the mean of the MVIB 2D stochastic encodings. For the MVIB stochastic encodings z∼p(z|x)=N(μ,σ2I), the depicted points represent the mean μ. The K dimension of the latent space has been set to 2 in order to allow a 2D visualisation of the encodings. For training MVIB, the JMVIB−T objective (Eq 8) has been optimised. For MVIB, five copies of the means plots are available, as they are obtained by training the model with five different independent training-test random splits. Both the PCA and the MVIB plots have been created starting from the default datasets collection. (ZIP) [file pcbi.1010050.s008.zip › s6-file/Early-Colorectal-EMBL/PCA_projections.png]

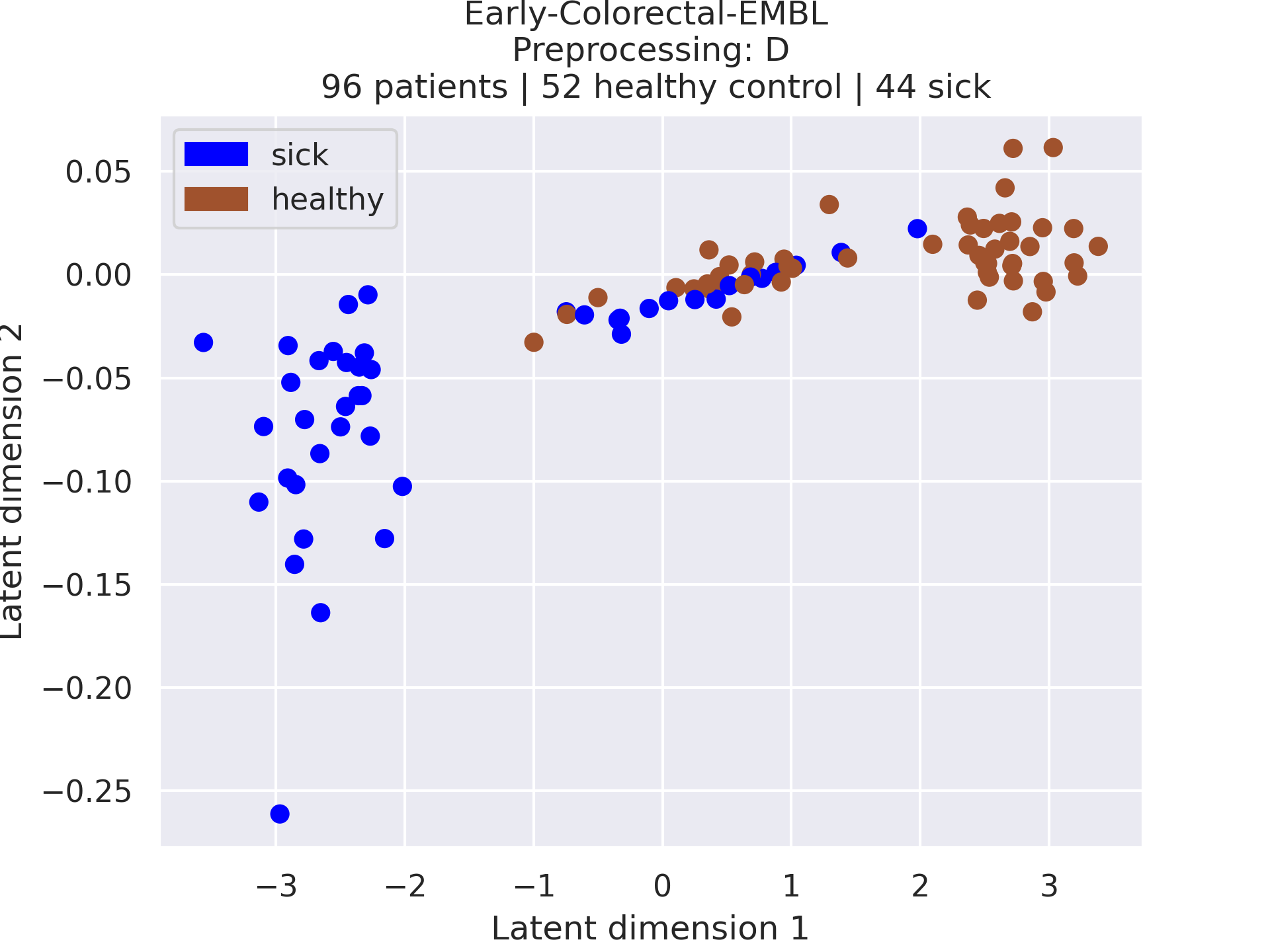

Supplement: S3 File — This file presents, for each dataset, the plots of the PCA 2D projections, as well as the plots of the mean of the MVIB 2D stochastic encodings. For the MVIB stochastic encodings z∼p(z|x)=N(μ,σ2I), the depicted points represent the mean μ. The K dimension of the latent space has been set to 2 in order to allow a 2D visualisation of the encodings. For training MVIB, the JMVIB−T objective (Eq 8) has been optimised. For MVIB, five copies of the means plots are available, as they are obtained by training the model with five different independent training-test random splits. Both the PCA and the MVIB plots have been created starting from the default datasets collection. (ZIP) [file pcbi.1010050.s008.zip › s6-file/Early-Colorectal-EMBL/2_embeddings.png]

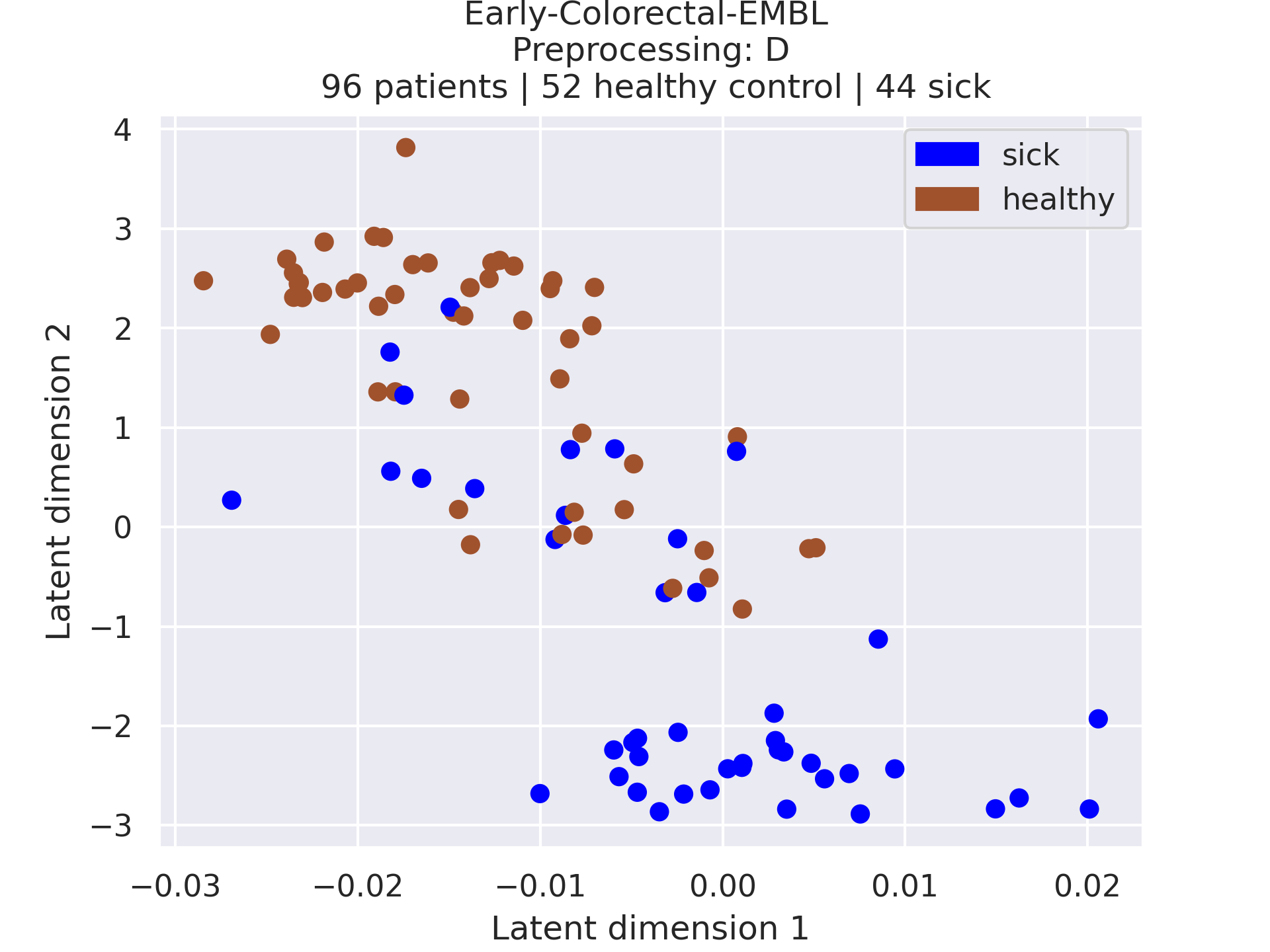

Supplement: S3 File — This file presents, for each dataset, the plots of the PCA 2D projections, as well as the plots of the mean of the MVIB 2D stochastic encodings. For the MVIB stochastic encodings z∼p(z|x)=N(μ,σ2I), the depicted points represent the mean μ. The K dimension of the latent space has been set to 2 in order to allow a 2D visualisation of the encodings. For training MVIB, the JMVIB−T objective (Eq 8) has been optimised. For MVIB, five copies of the means plots are available, as they are obtained by training the model with five different independent training-test random splits. Both the PCA and the MVIB plots have been created starting from the default datasets collection. (ZIP) [file pcbi.1010050.s008.zip › s6-file/Early-Colorectal-EMBL/0_embeddings.png]

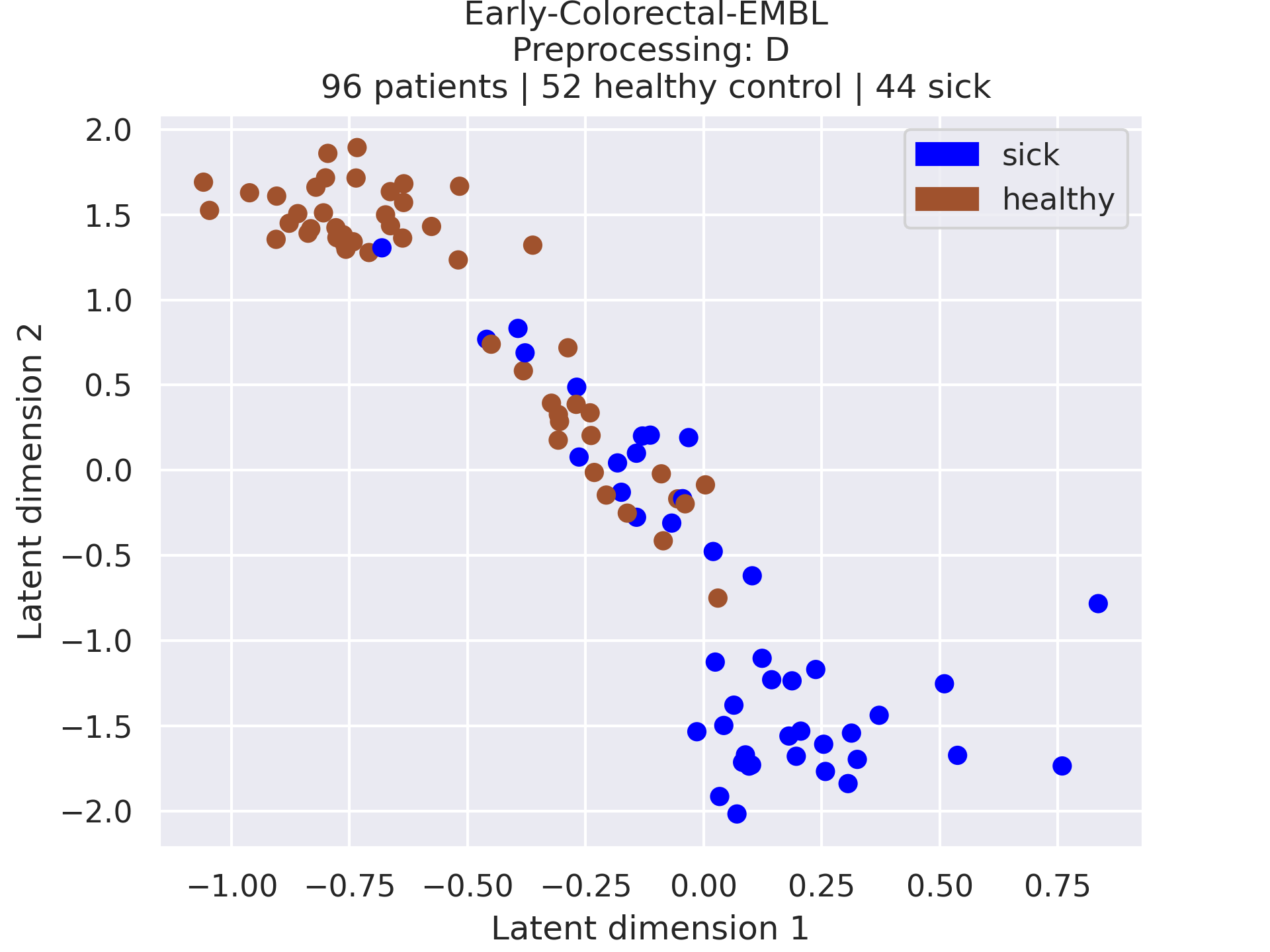

Supplement: S3 File — This file presents, for each dataset, the plots of the PCA 2D projections, as well as the plots of the mean of the MVIB 2D stochastic encodings. For the MVIB stochastic encodings z∼p(z|x)=N(μ,σ2I), the depicted points represent the mean μ. The K dimension of the latent space has been set to 2 in order to allow a 2D visualisation of the encodings. For training MVIB, the JMVIB−T objective (Eq 8) has been optimised. For MVIB, five copies of the means plots are available, as they are obtained by training the model with five different independent training-test random splits. Both the PCA and the MVIB plots have been created starting from the default datasets collection. (ZIP) [file pcbi.1010050.s008.zip › s6-file/Early-Colorectal-EMBL/1_embeddings.png]

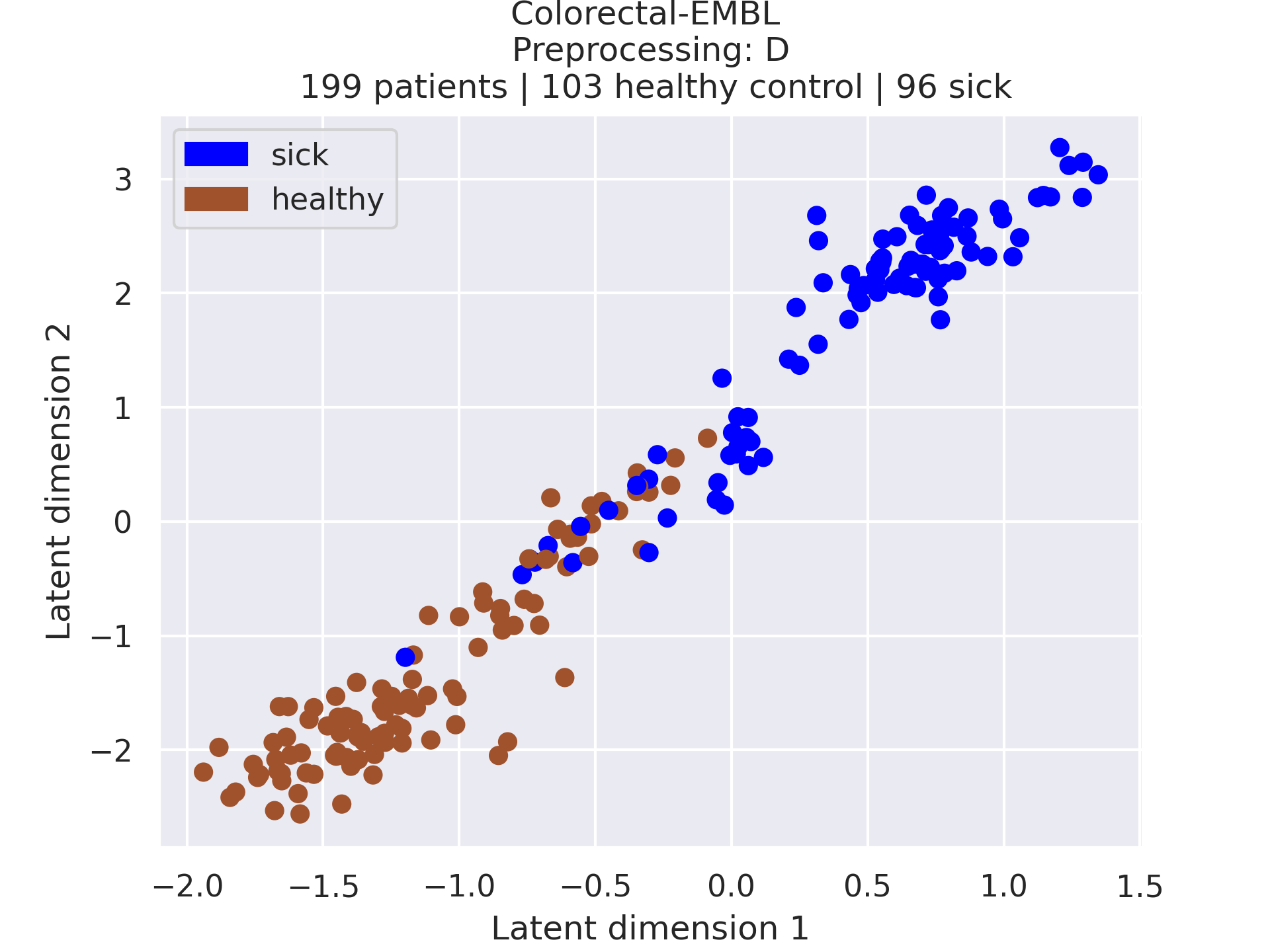

Supplement: S3 File — This file presents, for each dataset, the plots of the PCA 2D projections, as well as the plots of the mean of the MVIB 2D stochastic encodings. For the MVIB stochastic encodings z∼p(z|x)=N(μ,σ2I), the depicted points represent the mean μ. The K dimension of the latent space has been set to 2 in order to allow a 2D visualisation of the encodings. For training MVIB, the JMVIB−T objective (Eq 8) has been optimised. For MVIB, five copies of the means plots are available, as they are obtained by training the model with five different independent training-test random splits. Both the PCA and the MVIB plots have been created starting from the default datasets collection. (ZIP) [file pcbi.1010050.s008.zip › s6-file/Colorectal-EMBL/3_embeddings.png]

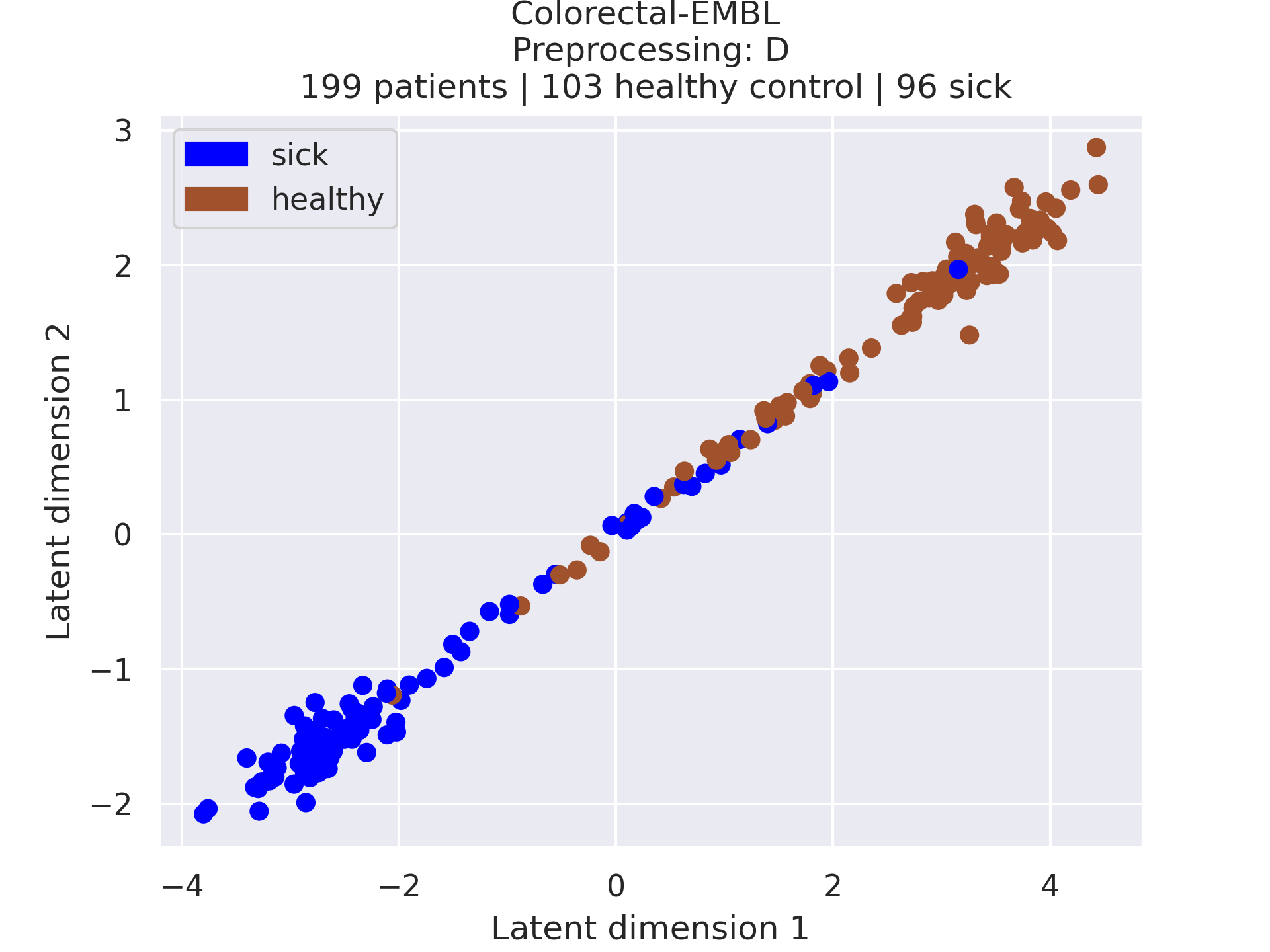

Supplement: S3 File — This file presents, for each dataset, the plots of the PCA 2D projections, as well as the plots of the mean of the MVIB 2D stochastic encodings. For the MVIB stochastic encodings z∼p(z|x)=N(μ,σ2I), the depicted points represent the mean μ. The K dimension of the latent space has been set to 2 in order to allow a 2D visualisation of the encodings. For training MVIB, the JMVIB−T objective (Eq 8) has been optimised. For MVIB, five copies of the means plots are available, as they are obtained by training the model with five different independent training-test random splits. Both the PCA and the MVIB plots have been created starting from the default datasets collection. (ZIP) [file pcbi.1010050.s008.zip › s6-file/Colorectal-EMBL/4_embeddings.png]

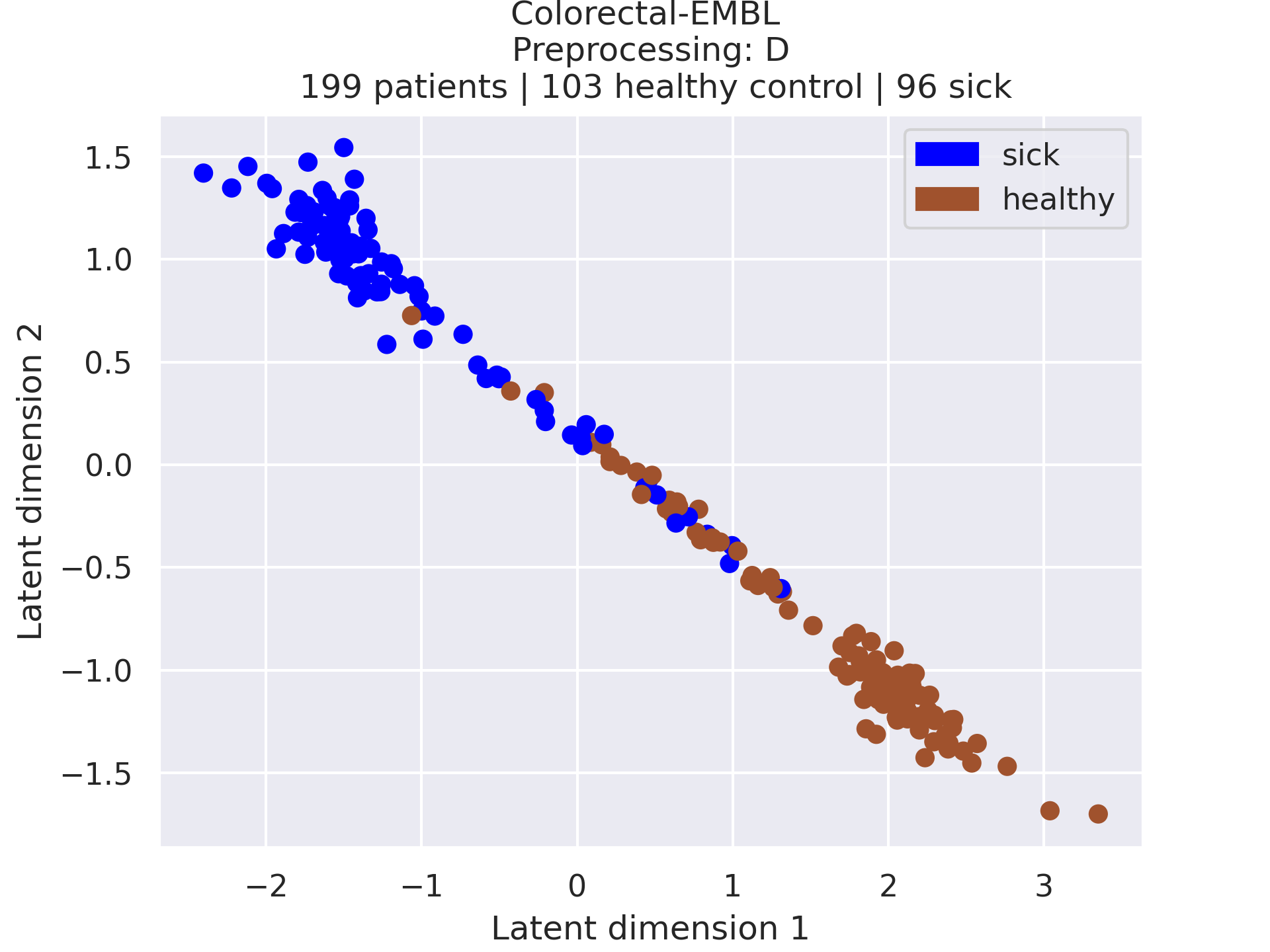

Supplement: S3 File — This file presents, for each dataset, the plots of the PCA 2D projections, as well as the plots of the mean of the MVIB 2D stochastic encodings. For the MVIB stochastic encodings z∼p(z|x)=N(μ,σ2I), the depicted points represent the mean μ. The K dimension of the latent space has been set to 2 in order to allow a 2D visualisation of the encodings. For training MVIB, the JMVIB−T objective (Eq 8) has been optimised. For MVIB, five copies of the means plots are available, as they are obtained by training the model with five different independent training-test random splits. Both the PCA and the MVIB plots have been created starting from the default datasets collection. (ZIP) [file pcbi.1010050.s008.zip › s6-file/Colorectal-EMBL/2_embeddings.png]

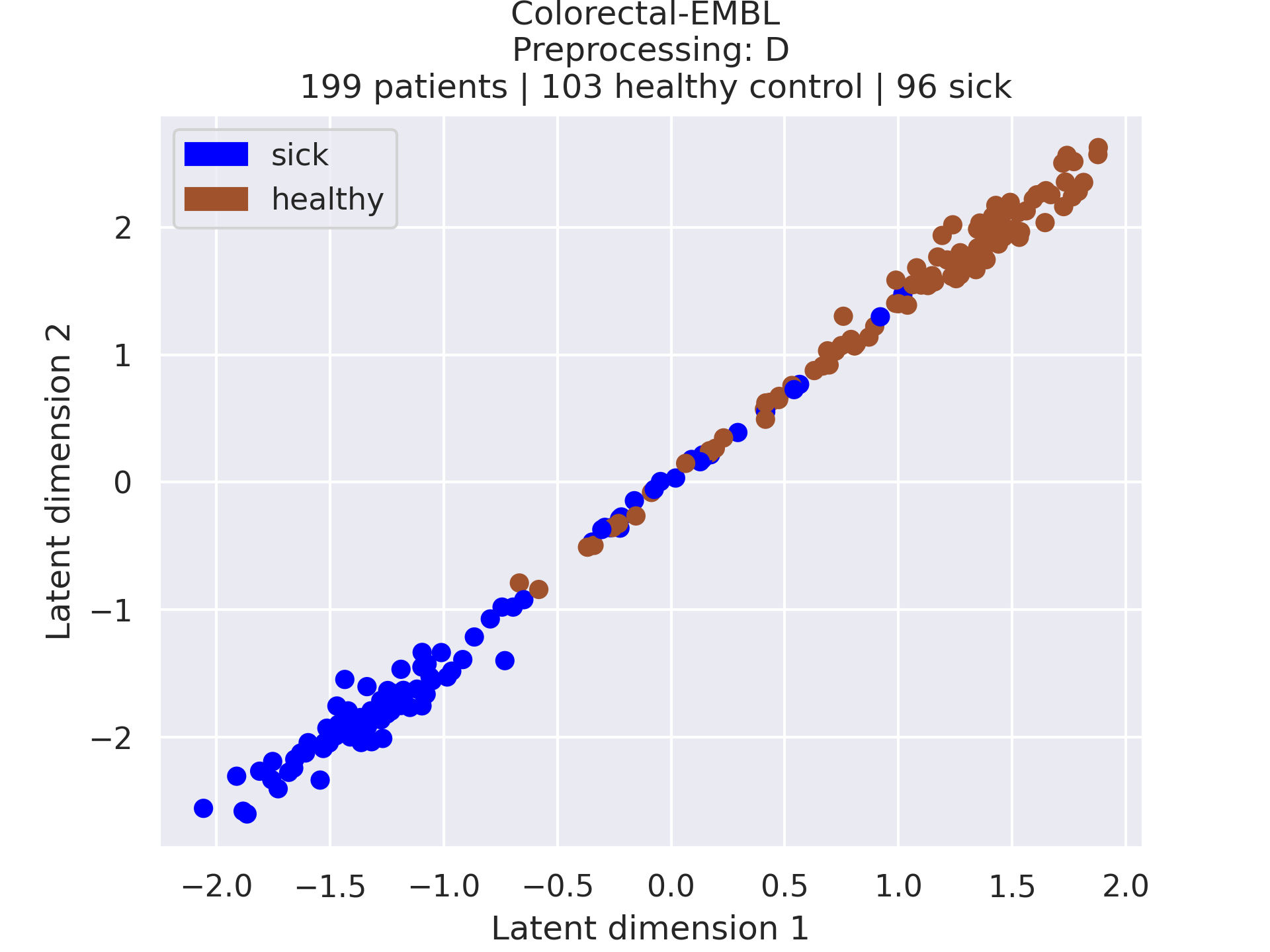

Supplement: S3 File — This file presents, for each dataset, the plots of the PCA 2D projections, as well as the plots of the mean of the MVIB 2D stochastic encodings. For the MVIB stochastic encodings z∼p(z|x)=N(μ,σ2I), the depicted points represent the mean μ. The K dimension of the latent space has been set to 2 in order to allow a 2D visualisation of the encodings. For training MVIB, the JMVIB−T objective (Eq 8) has been optimised. For MVIB, five copies of the means plots are available, as they are obtained by training the model with five different independent training-test random splits. Both the PCA and the MVIB plots have been created starting from the default datasets collection. (ZIP) [file pcbi.1010050.s008.zip › s6-file/Colorectal-EMBL/0_embeddings.png]

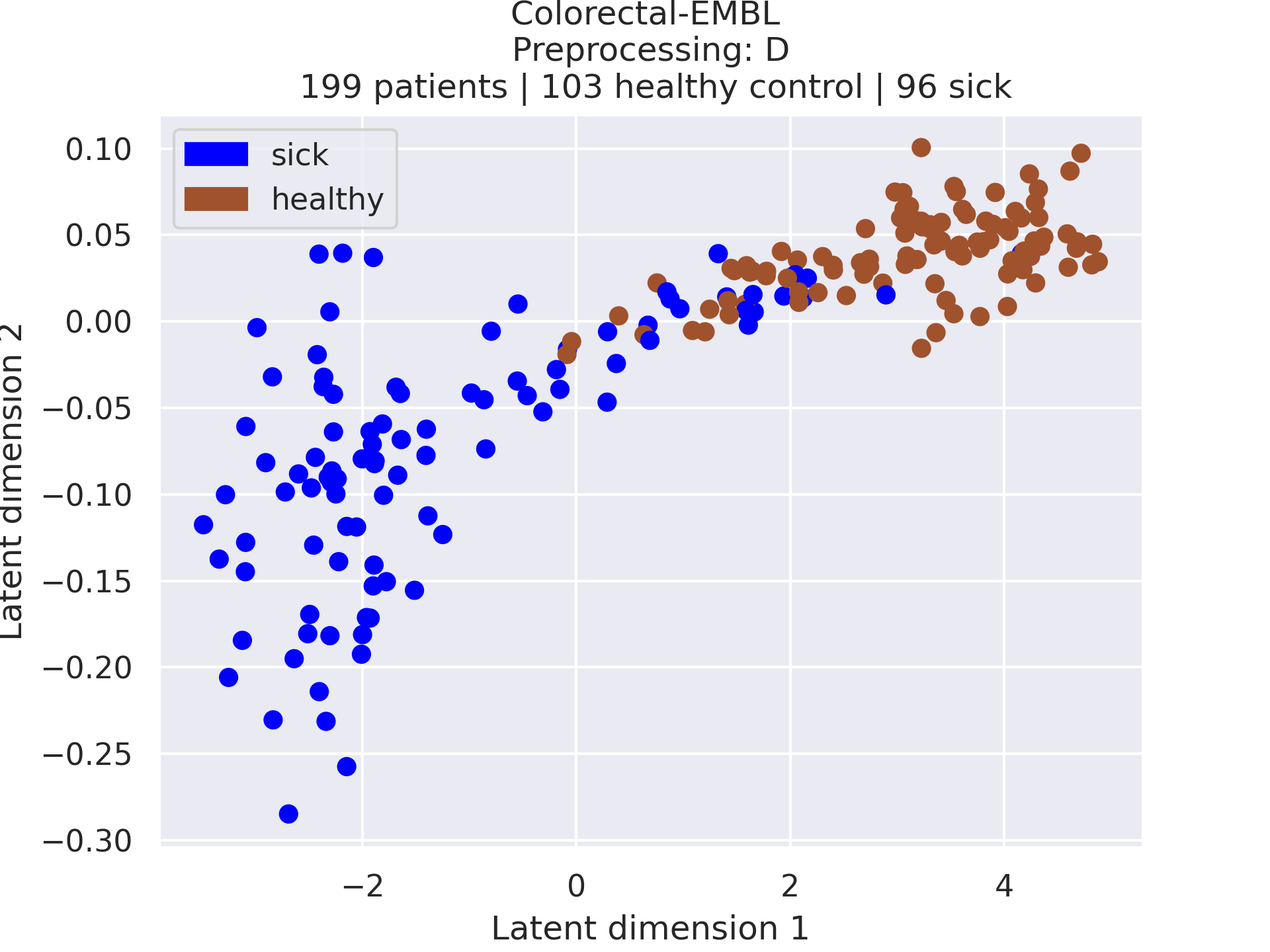

Supplement: S3 File — This file presents, for each dataset, the plots of the PCA 2D projections, as well as the plots of the mean of the MVIB 2D stochastic encodings. For the MVIB stochastic encodings z∼p(z|x)=N(μ,σ2I), the depicted points represent the mean μ. The K dimension of the latent space has been set to 2 in order to allow a 2D visualisation of the encodings. For training MVIB, the JMVIB−T objective (Eq 8) has been optimised. For MVIB, five copies of the means plots are available, as they are obtained by training the model with five different independent training-test random splits. Both the PCA and the MVIB plots have been created starting from the default datasets collection. (ZIP) [file pcbi.1010050.s008.zip › s6-file/Colorectal-EMBL/1_embeddings.png]

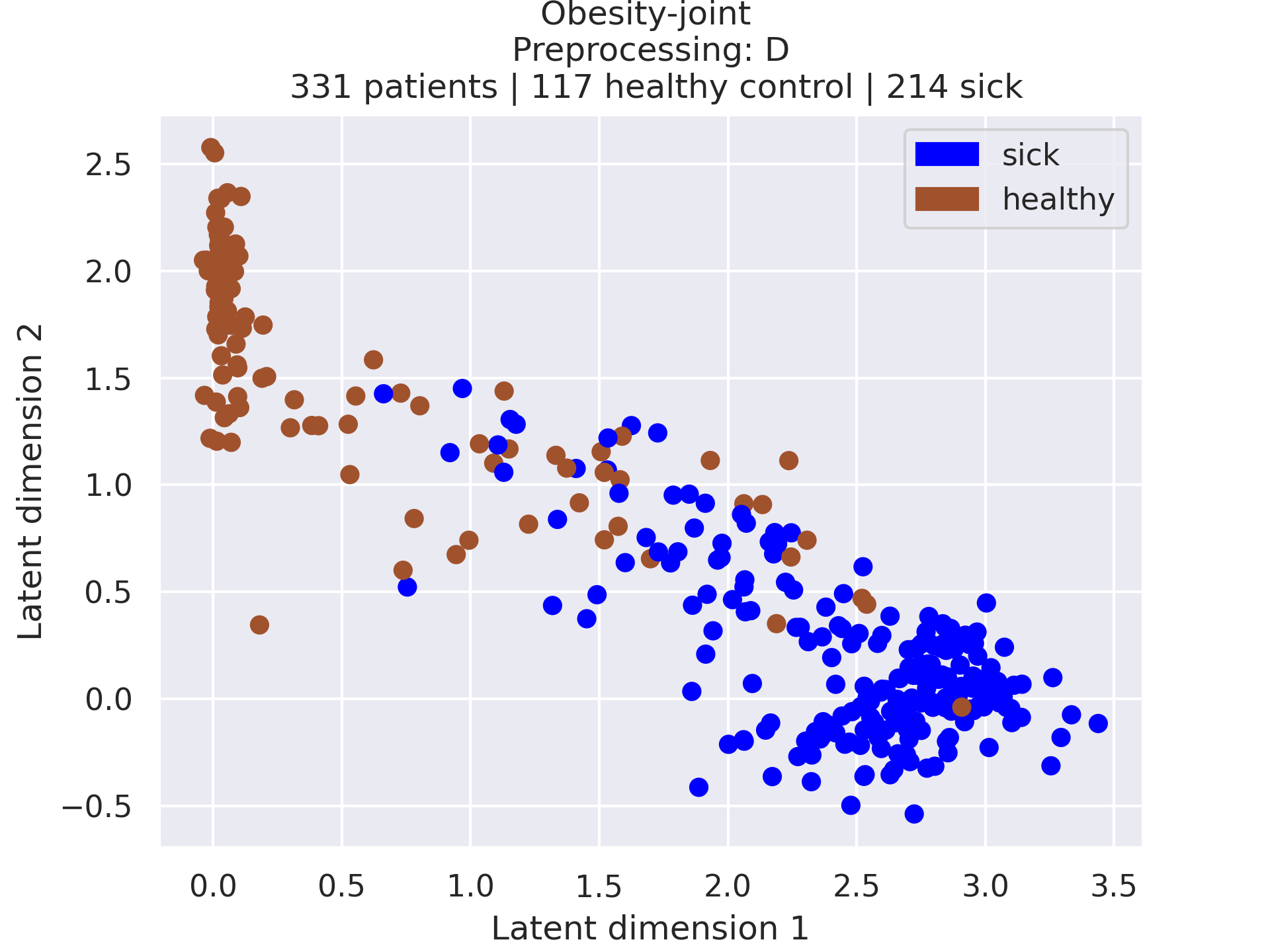

Supplement: S3 File — This file presents, for each dataset, the plots of the PCA 2D projections, as well as the plots of the mean of the MVIB 2D stochastic encodings. For the MVIB stochastic encodings z∼p(z|x)=N(μ,σ2I), the depicted points represent the mean μ. The K dimension of the latent space has been set to 2 in order to allow a 2D visualisation of the encodings. For training MVIB, the JMVIB−T objective (Eq 8) has been optimised. For MVIB, five copies of the means plots are available, as they are obtained by training the model with five different independent training-test random splits. Both the PCA and the MVIB plots have been created starting from the default datasets collection. (ZIP) [file pcbi.1010050.s008.zip › s6-file/Obesity-joint/3_embeddings.png]

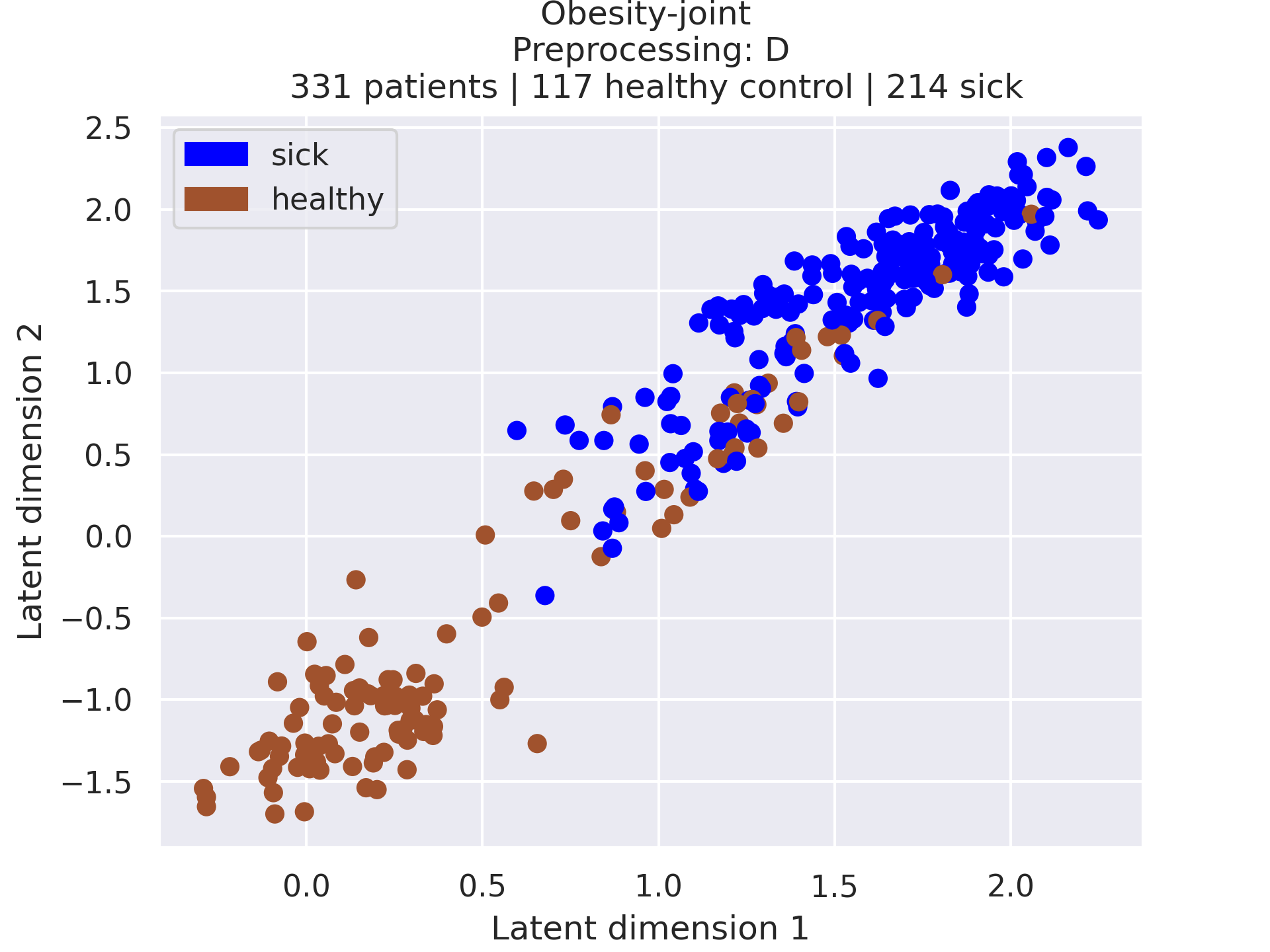

Supplement: S3 File — This file presents, for each dataset, the plots of the PCA 2D projections, as well as the plots of the mean of the MVIB 2D stochastic encodings. For the MVIB stochastic encodings z∼p(z|x)=N(μ,σ2I), the depicted points represent the mean μ. The K dimension of the latent space has been set to 2 in order to allow a 2D visualisation of the encodings. For training MVIB, the JMVIB−T objective (Eq 8) has been optimised. For MVIB, five copies of the means plots are available, as they are obtained by training the model with five different independent training-test random splits. Both the PCA and the MVIB plots have been created starting from the default datasets collection. (ZIP) [file pcbi.1010050.s008.zip › s6-file/Obesity-joint/4_embeddings.png]

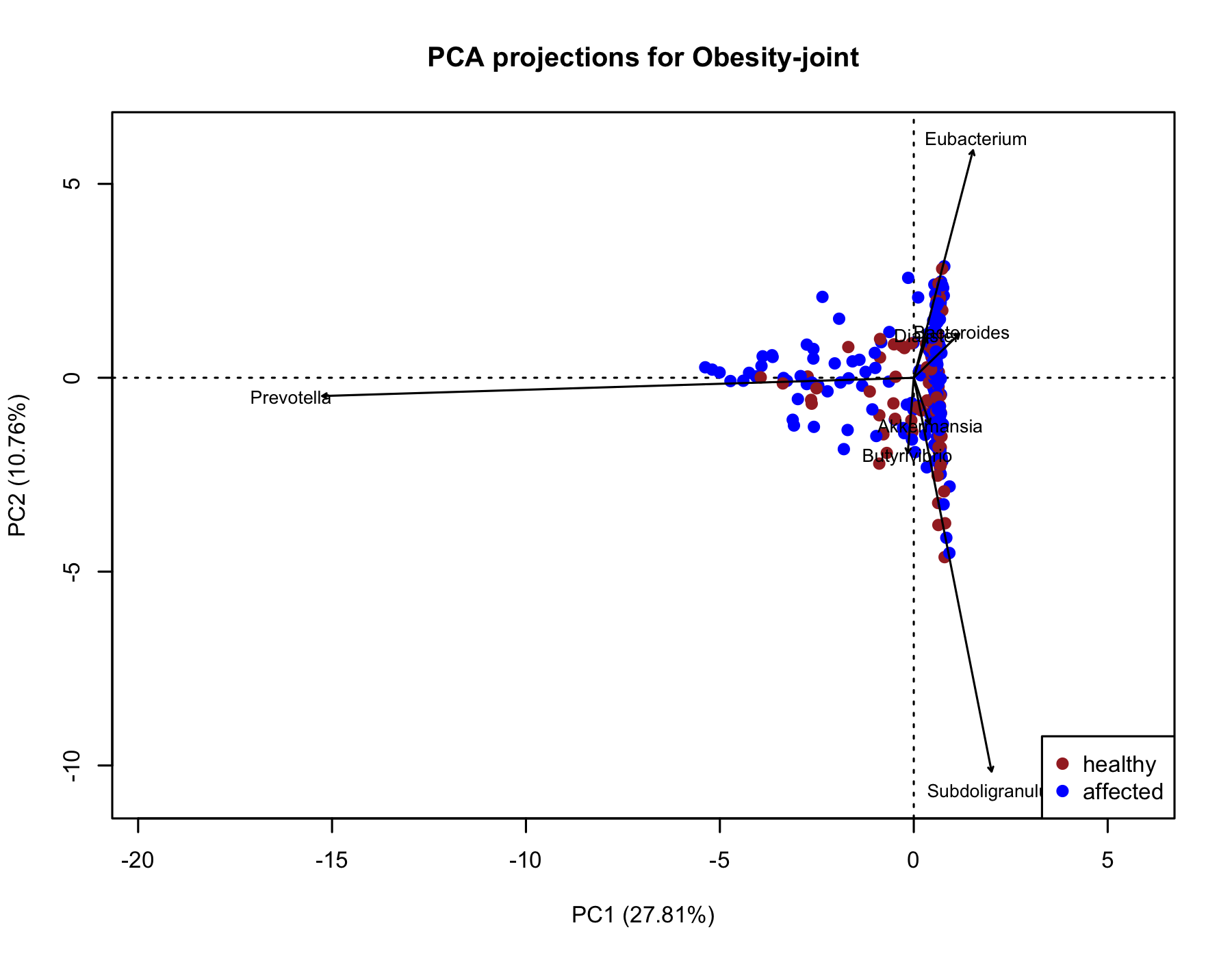

Supplement: S3 File — This file presents, for each dataset, the plots of the PCA 2D projections, as well as the plots of the mean of the MVIB 2D stochastic encodings. For the MVIB stochastic encodings z∼p(z|x)=N(μ,σ2I), the depicted points represent the mean μ. The K dimension of the latent space has been set to 2 in order to allow a 2D visualisation of the encodings. For training MVIB, the JMVIB−T objective (Eq 8) has been optimised. For MVIB, five copies of the means plots are available, as they are obtained by training the model with five different independent training-test random splits. Both the PCA and the MVIB plots have been created starting from the default datasets collection. (ZIP) [file pcbi.1010050.s008.zip › s6-file/Obesity-joint/PCA_projections.png]

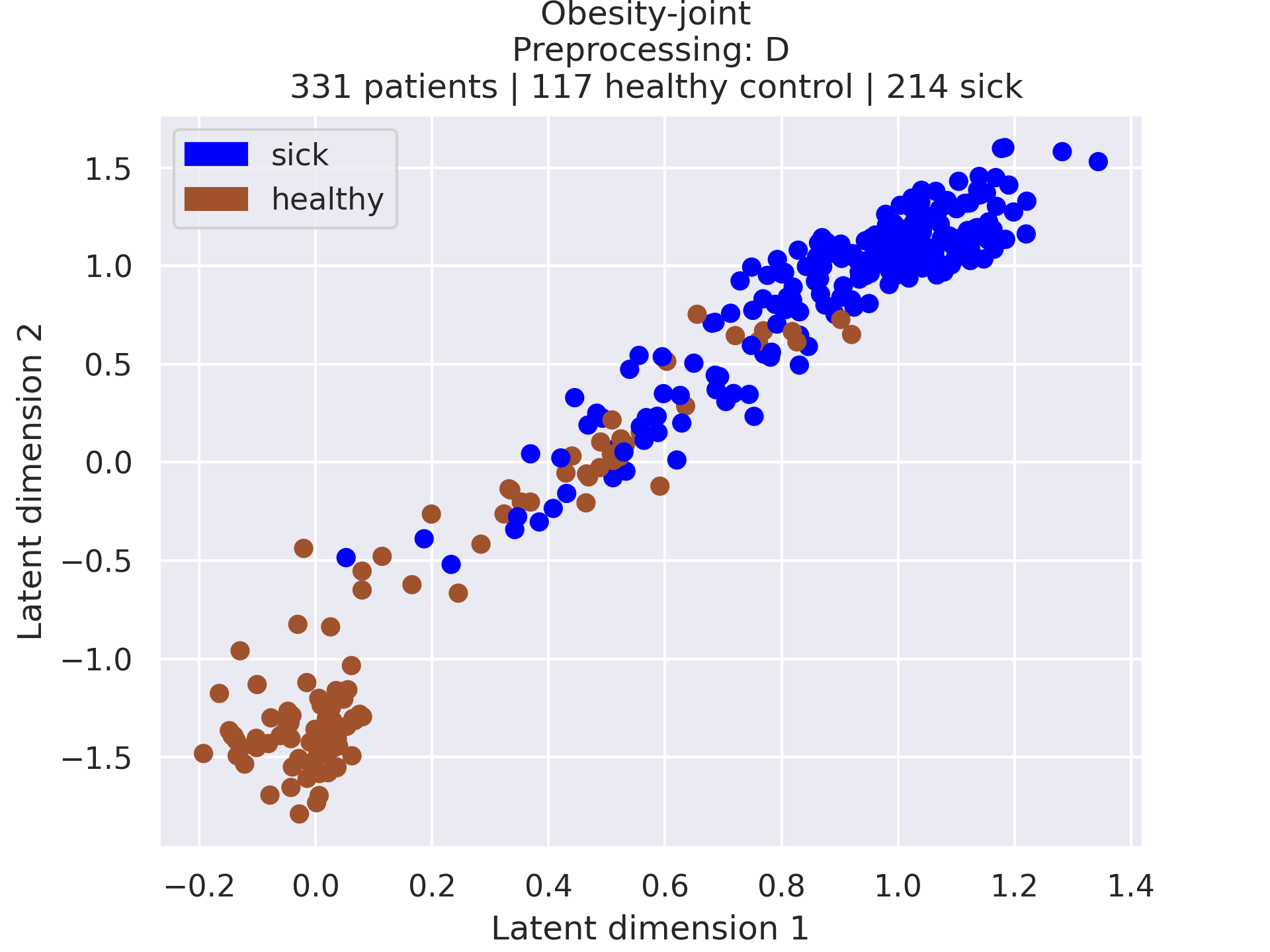

Supplement: S3 File — This file presents, for each dataset, the plots of the PCA 2D projections, as well as the plots of the mean of the MVIB 2D stochastic encodings. For the MVIB stochastic encodings z∼p(z|x)=N(μ,σ2I), the depicted points represent the mean μ. The K dimension of the latent space has been set to 2 in order to allow a 2D visualisation of the encodings. For training MVIB, the JMVIB−T objective (Eq 8) has been optimised. For MVIB, five copies of the means plots are available, as they are obtained by training the model with five different independent training-test random splits. Both the PCA and the MVIB plots have been created starting from the default datasets collection. (ZIP) [file pcbi.1010050.s008.zip › s6-file/Obesity-joint/2_embeddings.png]

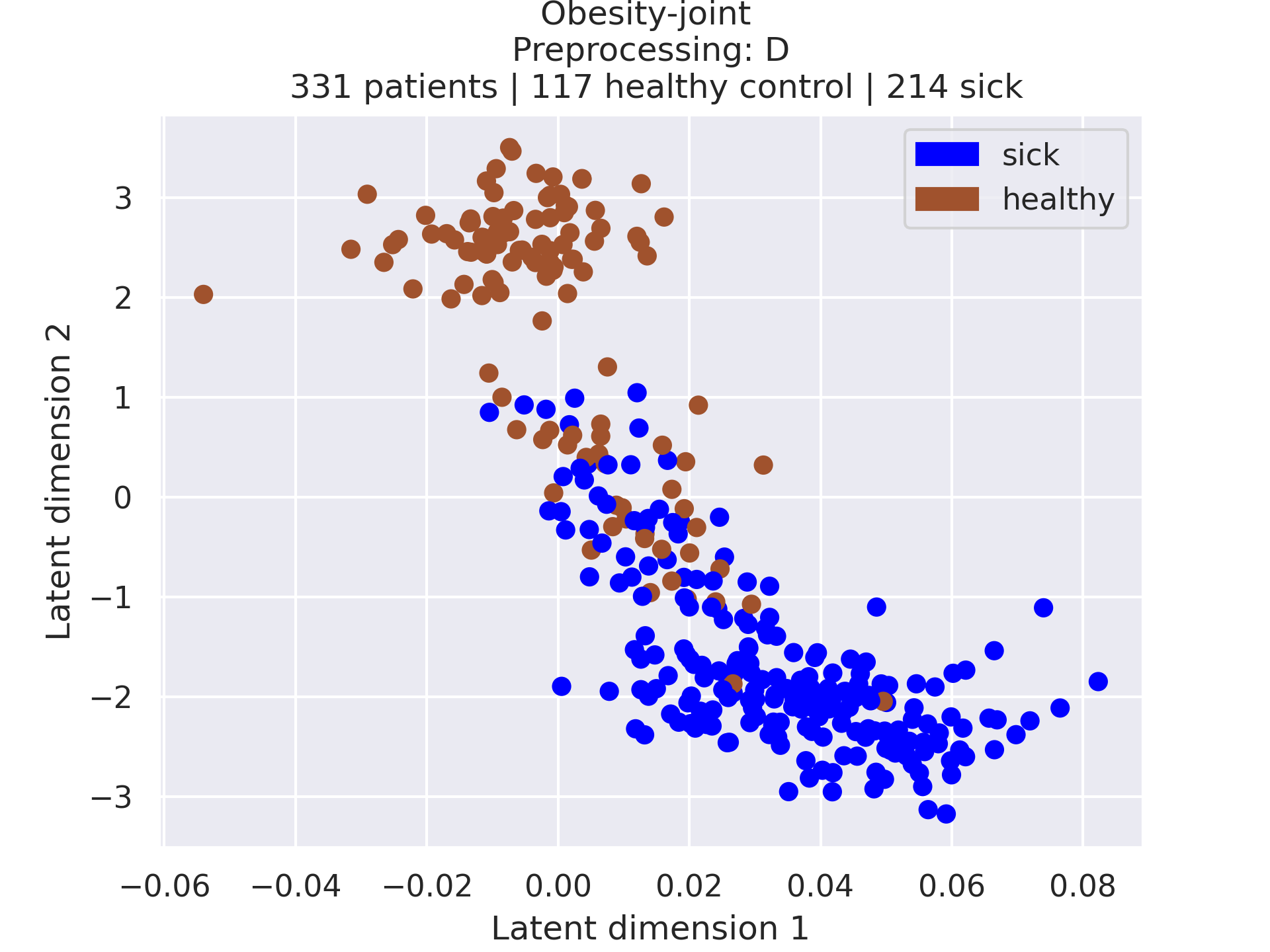

Supplement: S3 File — This file presents, for each dataset, the plots of the PCA 2D projections, as well as the plots of the mean of the MVIB 2D stochastic encodings. For the MVIB stochastic encodings z∼p(z|x)=N(μ,σ2I), the depicted points represent the mean μ. The K dimension of the latent space has been set to 2 in order to allow a 2D visualisation of the encodings. For training MVIB, the JMVIB−T objective (Eq 8) has been optimised. For MVIB, five copies of the means plots are available, as they are obtained by training the model with five different independent training-test random splits. Both the PCA and the MVIB plots have been created starting from the default datasets collection. (ZIP) [file pcbi.1010050.s008.zip › s6-file/Obesity-joint/0_embeddings.png]

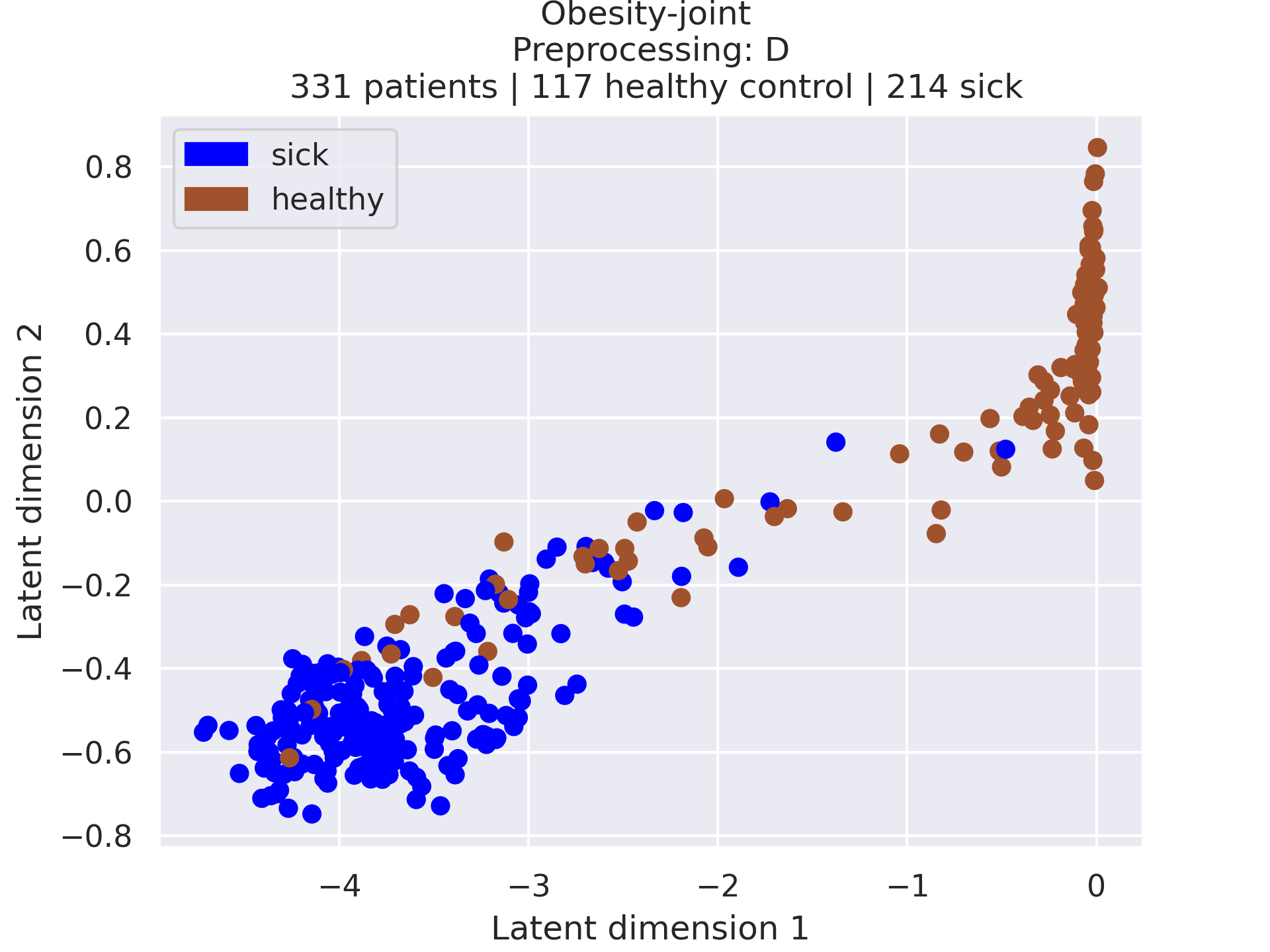

Supplement: S3 File — This file presents, for each dataset, the plots of the PCA 2D projections, as well as the plots of the mean of the MVIB 2D stochastic encodings. For the MVIB stochastic encodings z∼p(z|x)=N(μ,σ2I), the depicted points represent the mean μ. The K dimension of the latent space has been set to 2 in order to allow a 2D visualisation of the encodings. For training MVIB, the JMVIB−T objective (Eq 8) has been optimised. For MVIB, five copies of the means plots are available, as they are obtained by training the model with five different independent training-test random splits. Both the PCA and the MVIB plots have been created starting from the default datasets collection. (ZIP) [file pcbi.1010050.s008.zip › s6-file/Obesity-joint/1_embeddings.png]

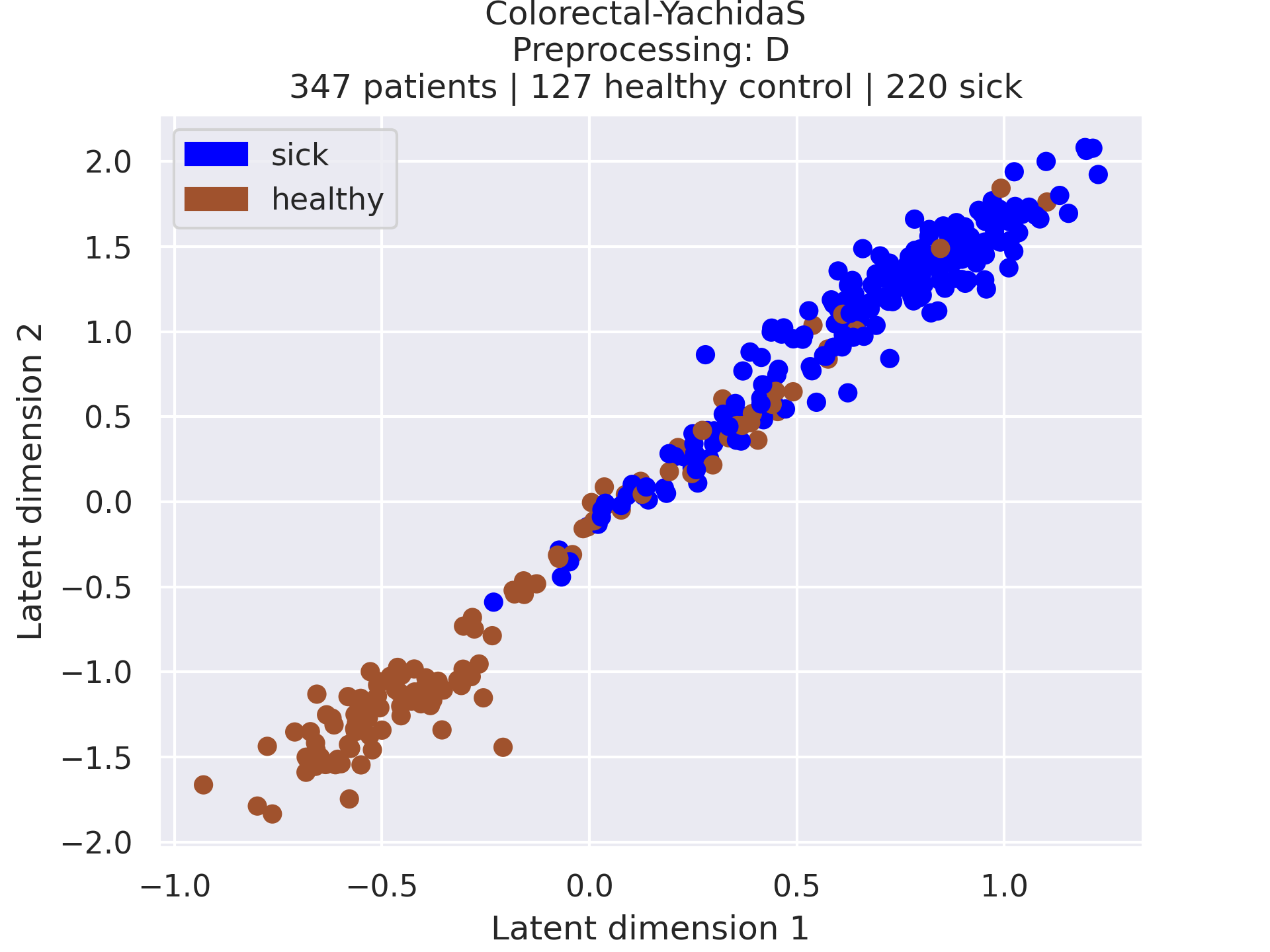

Supplement: S3 File — This file presents, for each dataset, the plots of the PCA 2D projections, as well as the plots of the mean of the MVIB 2D stochastic encodings. For the MVIB stochastic encodings z∼p(z|x)=N(μ,σ2I), the depicted points represent the mean μ. The K dimension of the latent space has been set to 2 in order to allow a 2D visualisation of the encodings. For training MVIB, the JMVIB−T objective (Eq 8) has been optimised. For MVIB, five copies of the means plots are available, as they are obtained by training the model with five different independent training-test random splits. Both the PCA and the MVIB plots have been created starting from the default datasets collection. (ZIP) [file pcbi.1010050.s008.zip › s6-file/Colorectal-YachidaS/3_embeddings.png]

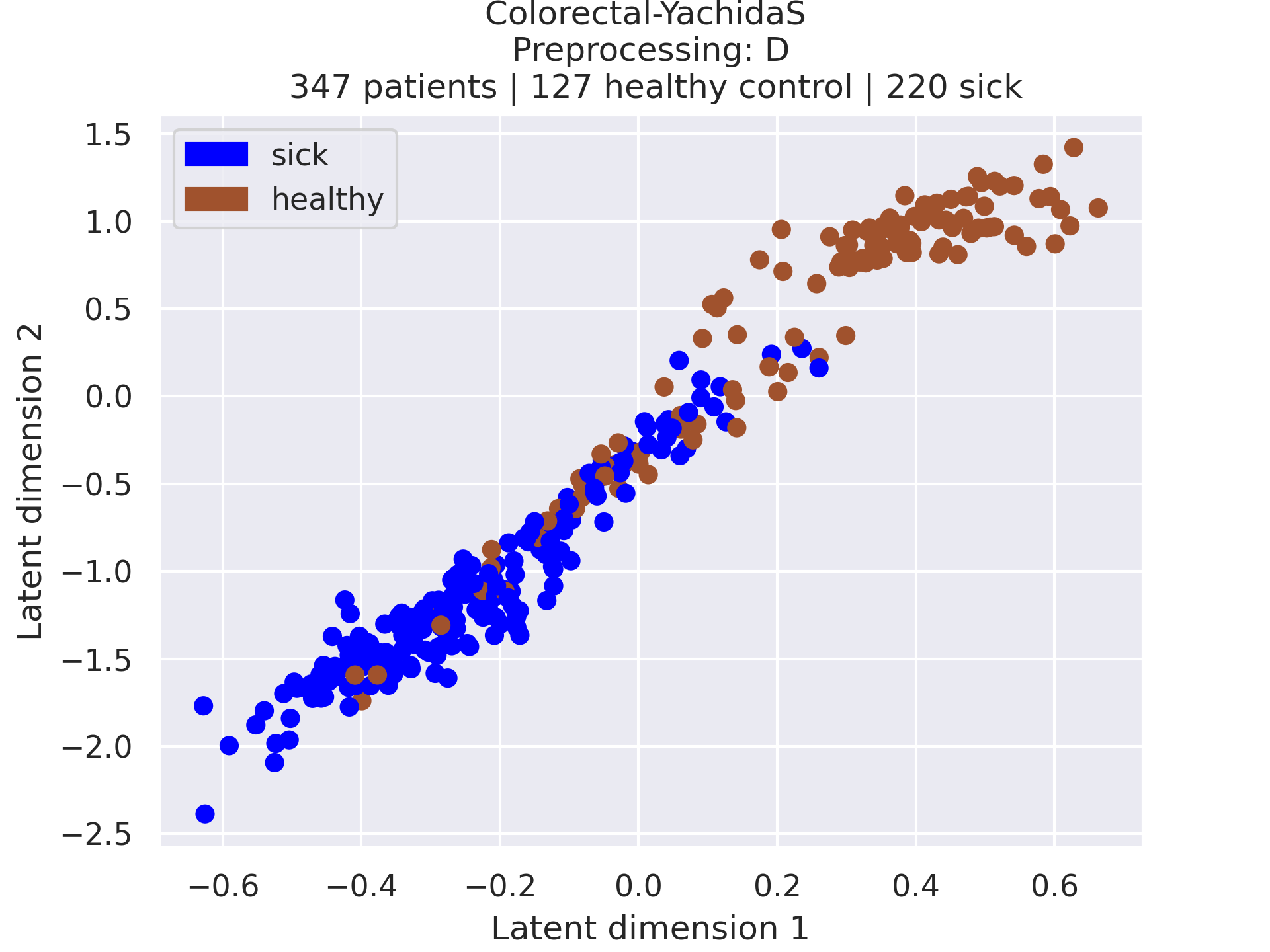

Supplement: S3 File — This file presents, for each dataset, the plots of the PCA 2D projections, as well as the plots of the mean of the MVIB 2D stochastic encodings. For the MVIB stochastic encodings z∼p(z|x)=N(μ,σ2I), the depicted points represent the mean μ. The K dimension of the latent space has been set to 2 in order to allow a 2D visualisation of the encodings. For training MVIB, the JMVIB−T objective (Eq 8) has been optimised. For MVIB, five copies of the means plots are available, as they are obtained by training the model with five different independent training-test random splits. Both the PCA and the MVIB plots have been created starting from the default datasets collection. (ZIP) [file pcbi.1010050.s008.zip › s6-file/Colorectal-YachidaS/4_embeddings.png]

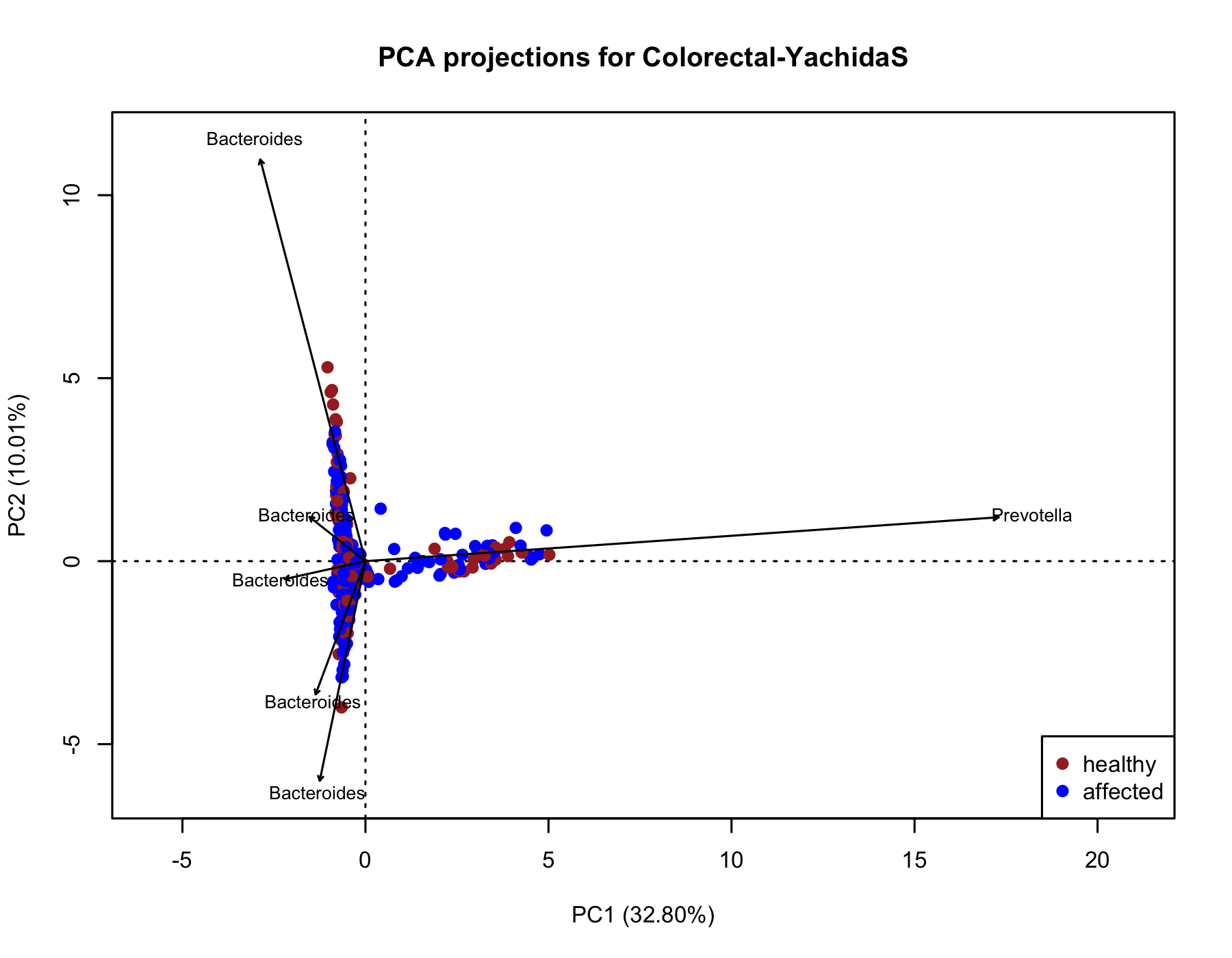

Supplement: S3 File — This file presents, for each dataset, the plots of the PCA 2D projections, as well as the plots of the mean of the MVIB 2D stochastic encodings. For the MVIB stochastic encodings z∼p(z|x)=N(μ,σ2I), the depicted points represent the mean μ. The K dimension of the latent space has been set to 2 in order to allow a 2D visualisation of the encodings. For training MVIB, the JMVIB−T objective (Eq 8) has been optimised. For MVIB, five copies of the means plots are available, as they are obtained by training the model with five different independent training-test random splits. Both the PCA and the MVIB plots have been created starting from the default datasets collection. (ZIP) [file pcbi.1010050.s008.zip › s6-file/Colorectal-YachidaS/PCA_projections.png]

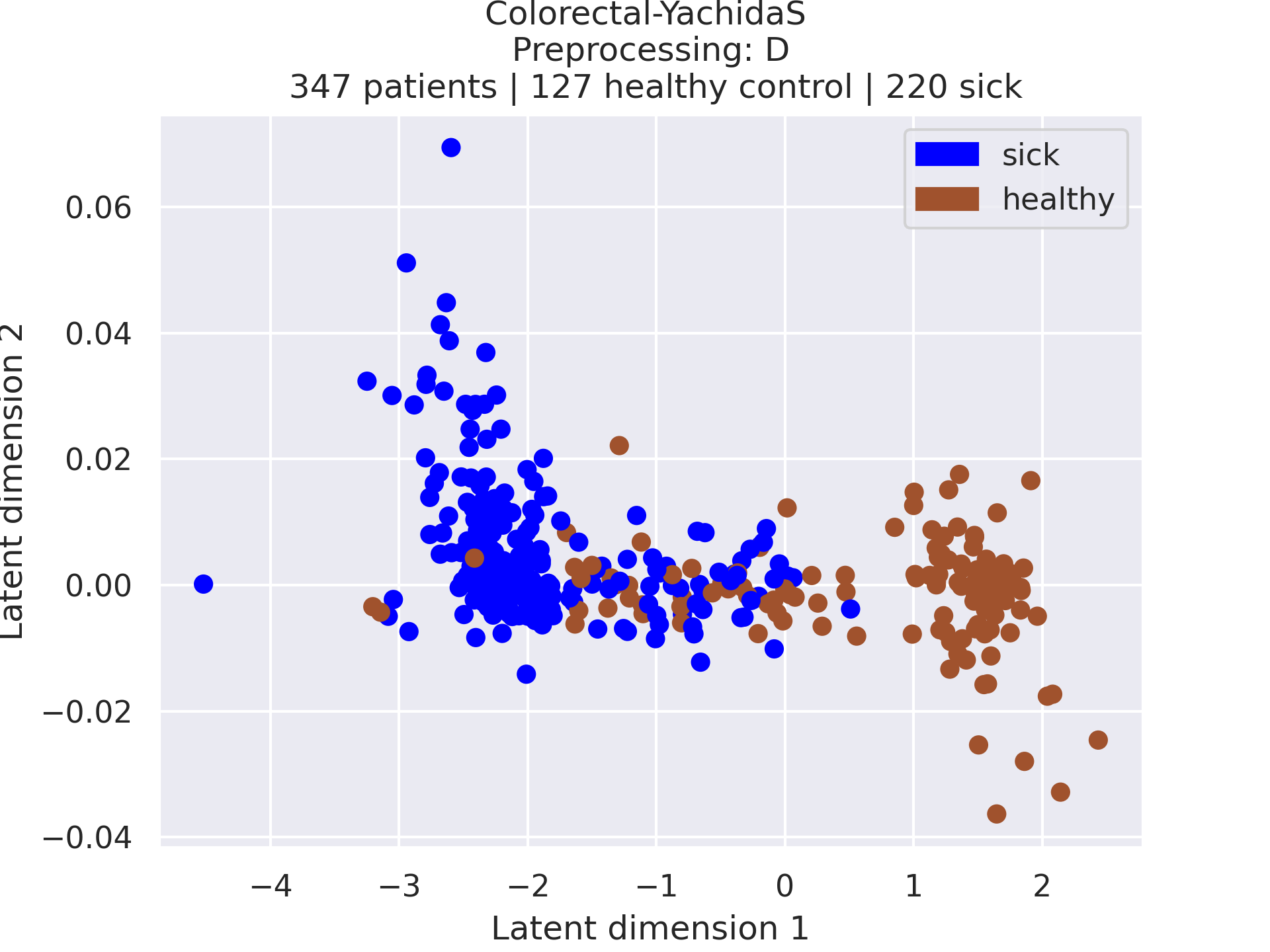

Supplement: S3 File — This file presents, for each dataset, the plots of the PCA 2D projections, as well as the plots of the mean of the MVIB 2D stochastic encodings. For the MVIB stochastic encodings z∼p(z|x)=N(μ,σ2I), the depicted points represent the mean μ. The K dimension of the latent space has been set to 2 in order to allow a 2D visualisation of the encodings. For training MVIB, the JMVIB−T objective (Eq 8) has been optimised. For MVIB, five copies of the means plots are available, as they are obtained by training the model with five different independent training-test random splits. Both the PCA and the MVIB plots have been created starting from the default datasets collection. (ZIP) [file pcbi.1010050.s008.zip › s6-file/Colorectal-YachidaS/2_embeddings.png]

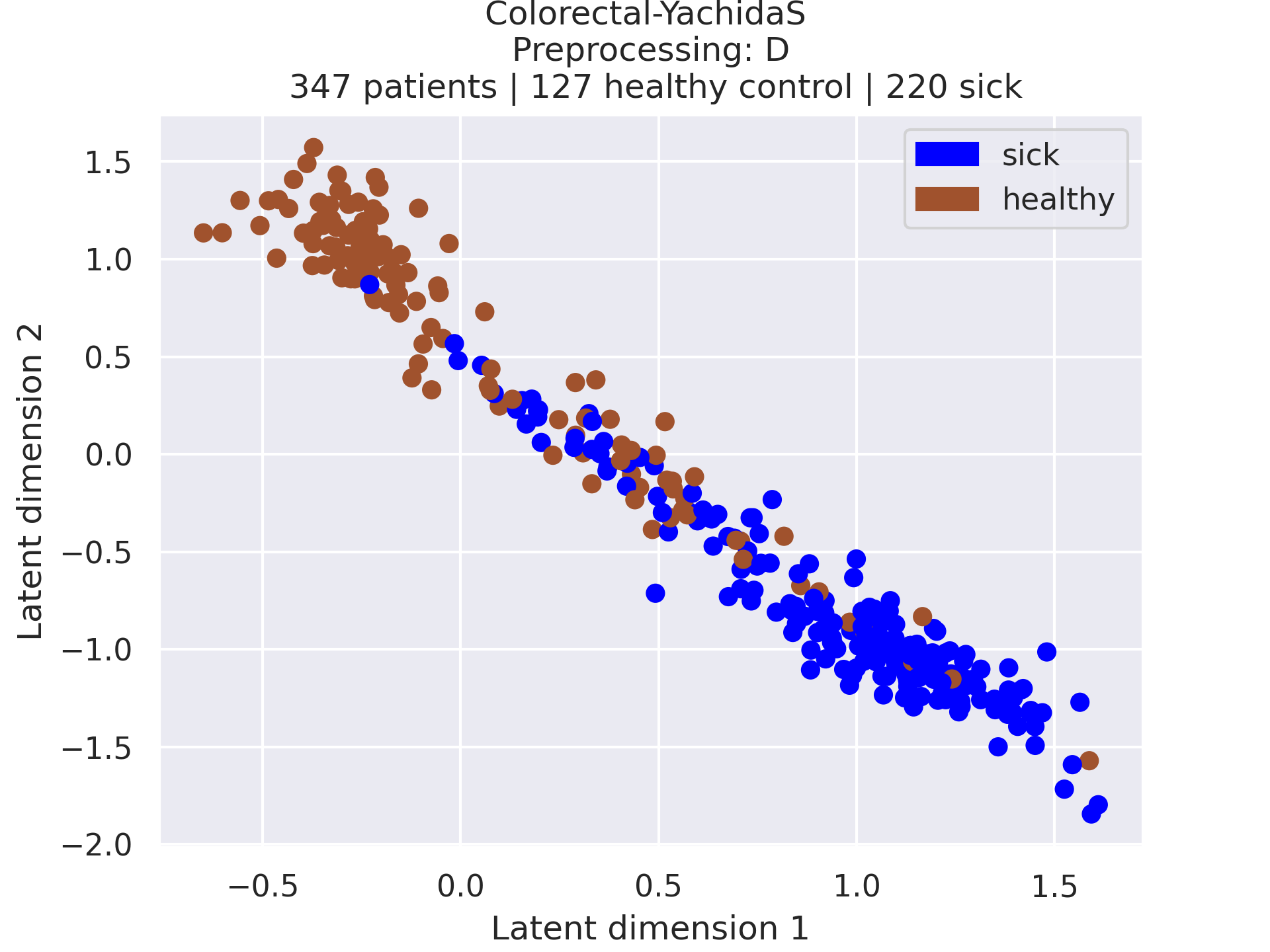

Supplement: S3 File — This file presents, for each dataset, the plots of the PCA 2D projections, as well as the plots of the mean of the MVIB 2D stochastic encodings. For the MVIB stochastic encodings z∼p(z|x)=N(μ,σ2I), the depicted points represent the mean μ. The K dimension of the latent space has been set to 2 in order to allow a 2D visualisation of the encodings. For training MVIB, the JMVIB−T objective (Eq 8) has been optimised. For MVIB, five copies of the means plots are available, as they are obtained by training the model with five different independent training-test random splits. Both the PCA and the MVIB plots have been created starting from the default datasets collection. (ZIP) [file pcbi.1010050.s008.zip › s6-file/Colorectal-YachidaS/0_embeddings.png]

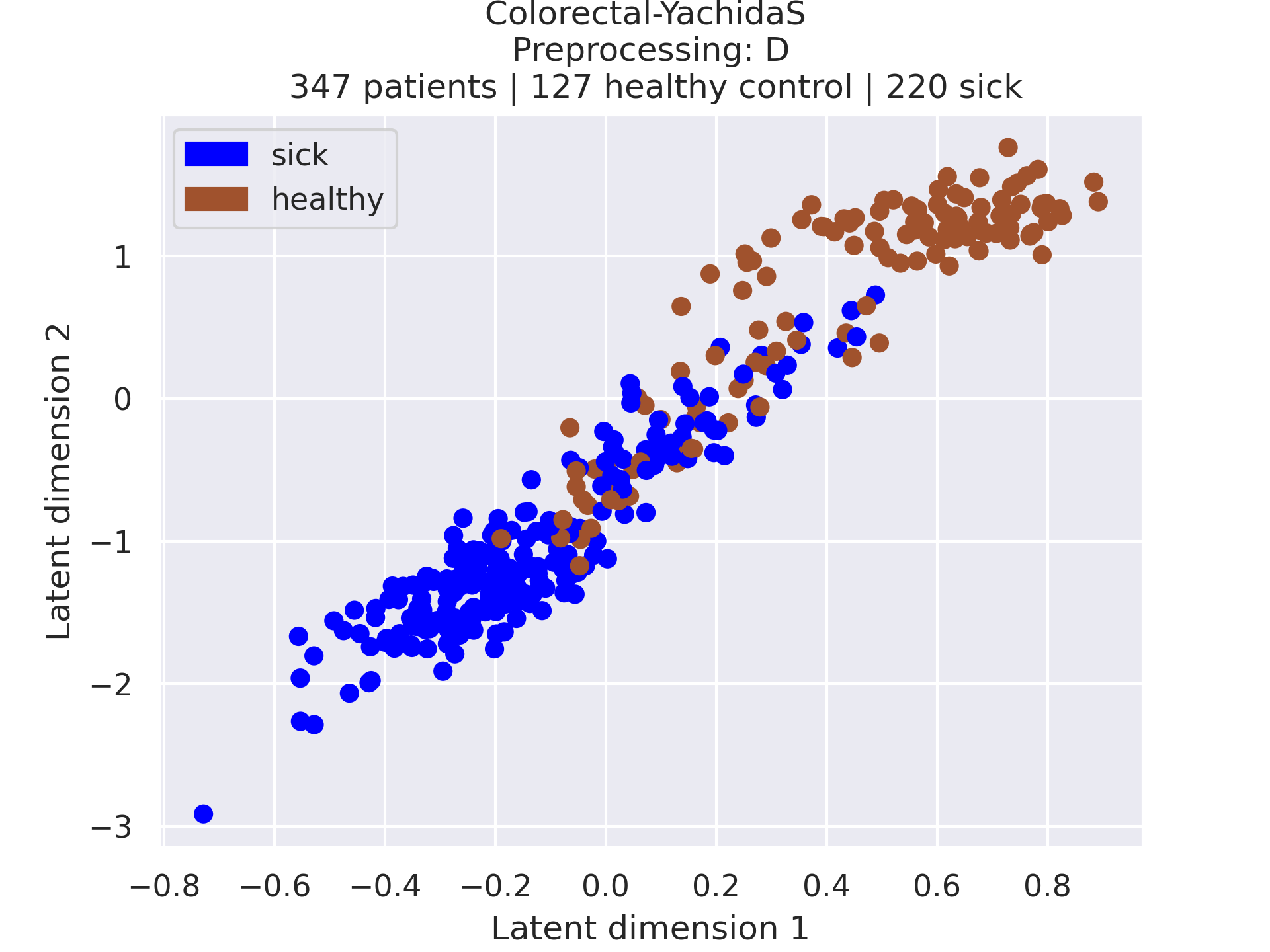

Supplement: S3 File — This file presents, for each dataset, the plots of the PCA 2D projections, as well as the plots of the mean of the MVIB 2D stochastic encodings. For the MVIB stochastic encodings z∼p(z|x)=N(μ,σ2I), the depicted points represent the mean μ. The K dimension of the latent space has been set to 2 in order to allow a 2D visualisation of the encodings. For training MVIB, the JMVIB−T objective (Eq 8) has been optimised. For MVIB, five copies of the means plots are available, as they are obtained by training the model with five different independent training-test random splits. Both the PCA and the MVIB plots have been created starting from the default datasets collection. (ZIP) [file pcbi.1010050.s008.zip › s6-file/Colorectal-YachidaS/1_embeddings.png]

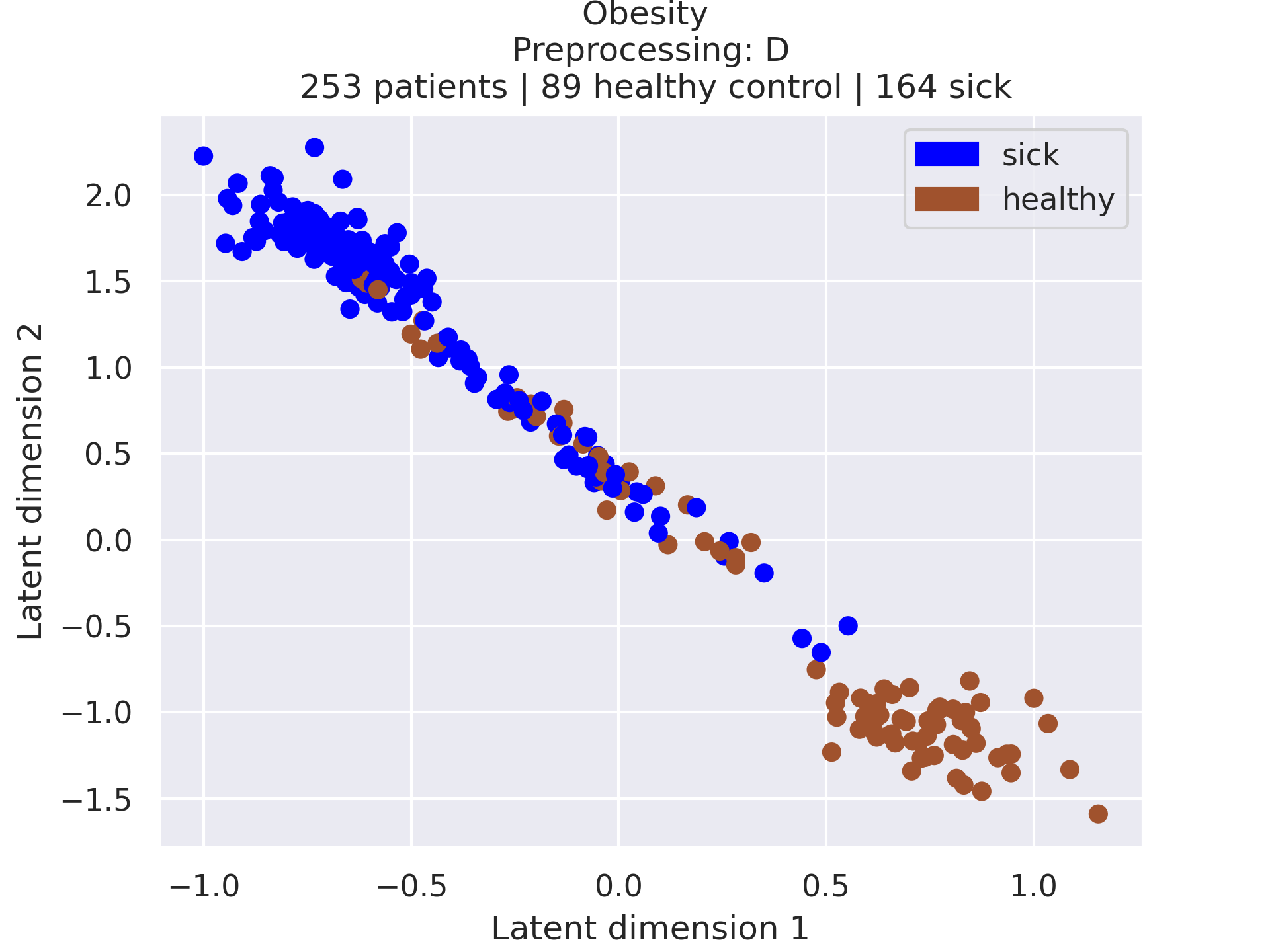

Supplement: S3 File — This file presents, for each dataset, the plots of the PCA 2D projections, as well as the plots of the mean of the MVIB 2D stochastic encodings. For the MVIB stochastic encodings z∼p(z|x)=N(μ,σ2I), the depicted points represent the mean μ. The K dimension of the latent space has been set to 2 in order to allow a 2D visualisation of the encodings. For training MVIB, the JMVIB−T objective (Eq 8) has been optimised. For MVIB, five copies of the means plots are available, as they are obtained by training the model with five different independent training-test random splits. Both the PCA and the MVIB plots have been created starting from the default datasets collection. (ZIP) [file pcbi.1010050.s008.zip › s6-file/Obesity/3_embeddings.png]

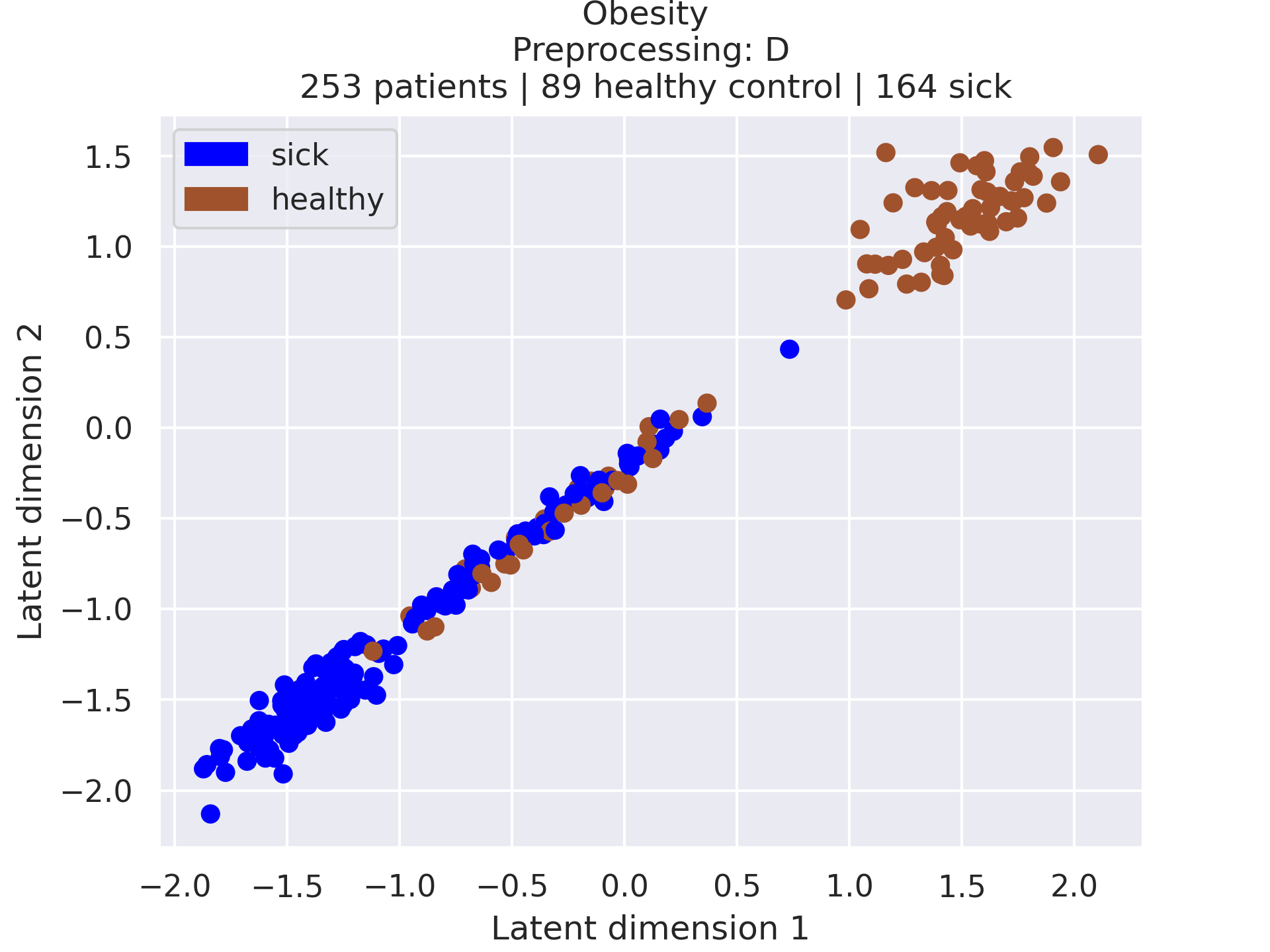

Supplement: S3 File — This file presents, for each dataset, the plots of the PCA 2D projections, as well as the plots of the mean of the MVIB 2D stochastic encodings. For the MVIB stochastic encodings z∼p(z|x)=N(μ,σ2I), the depicted points represent the mean μ. The K dimension of the latent space has been set to 2 in order to allow a 2D visualisation of the encodings. For training MVIB, the JMVIB−T objective (Eq 8) has been optimised. For MVIB, five copies of the means plots are available, as they are obtained by training the model with five different independent training-test random splits. Both the PCA and the MVIB plots have been created starting from the default datasets collection. (ZIP) [file pcbi.1010050.s008.zip › s6-file/Obesity/4_embeddings.png]

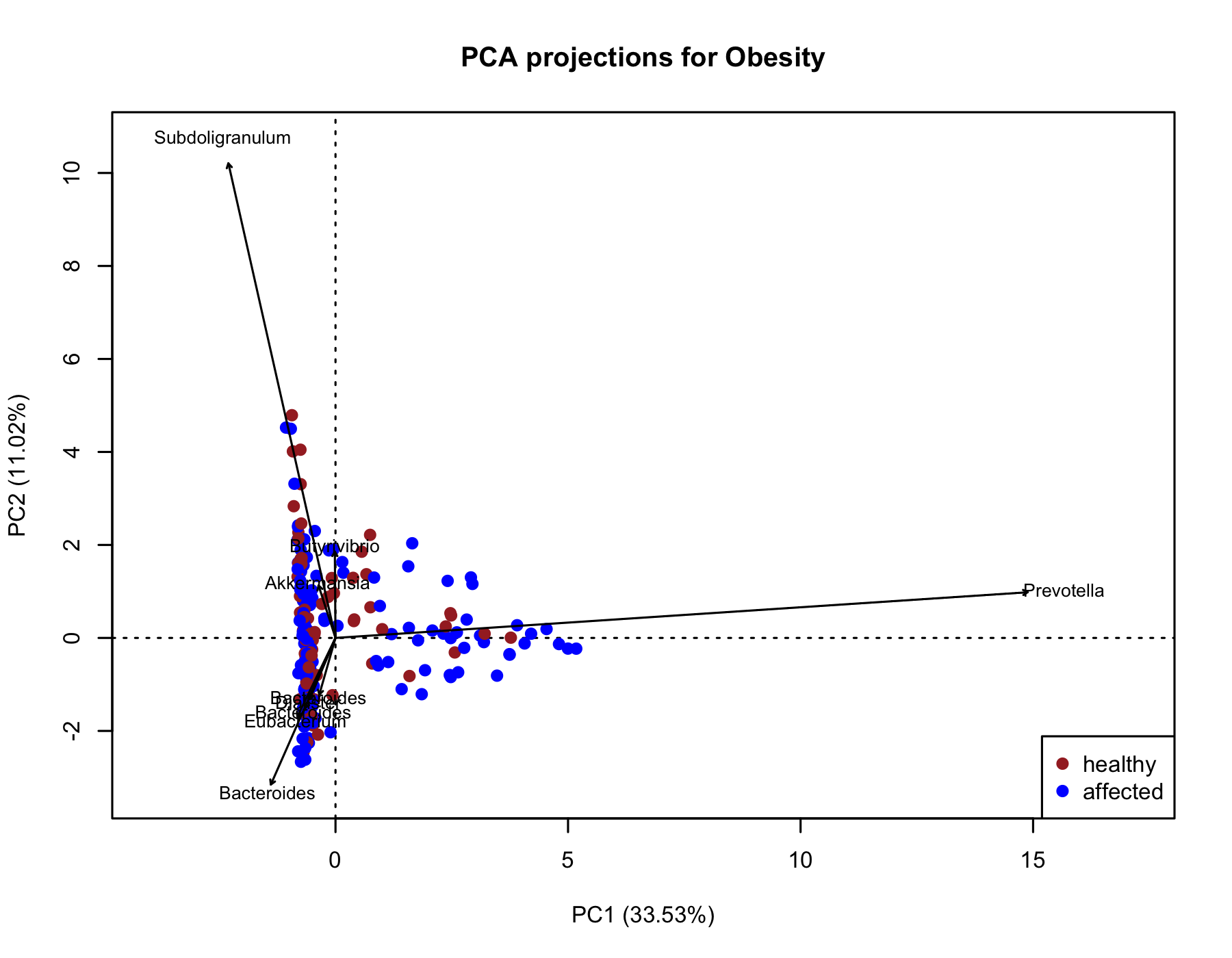

Supplement: S3 File — This file presents, for each dataset, the plots of the PCA 2D projections, as well as the plots of the mean of the MVIB 2D stochastic encodings. For the MVIB stochastic encodings z∼p(z|x)=N(μ,σ2I), the depicted points represent the mean μ. The K dimension of the latent space has been set to 2 in order to allow a 2D visualisation of the encodings. For training MVIB, the JMVIB−T objective (Eq 8) has been optimised. For MVIB, five copies of the means plots are available, as they are obtained by training the model with five different independent training-test random splits. Both the PCA and the MVIB plots have been created starting from the default datasets collection. (ZIP) [file pcbi.1010050.s008.zip › s6-file/Obesity/PCA_projections.png]

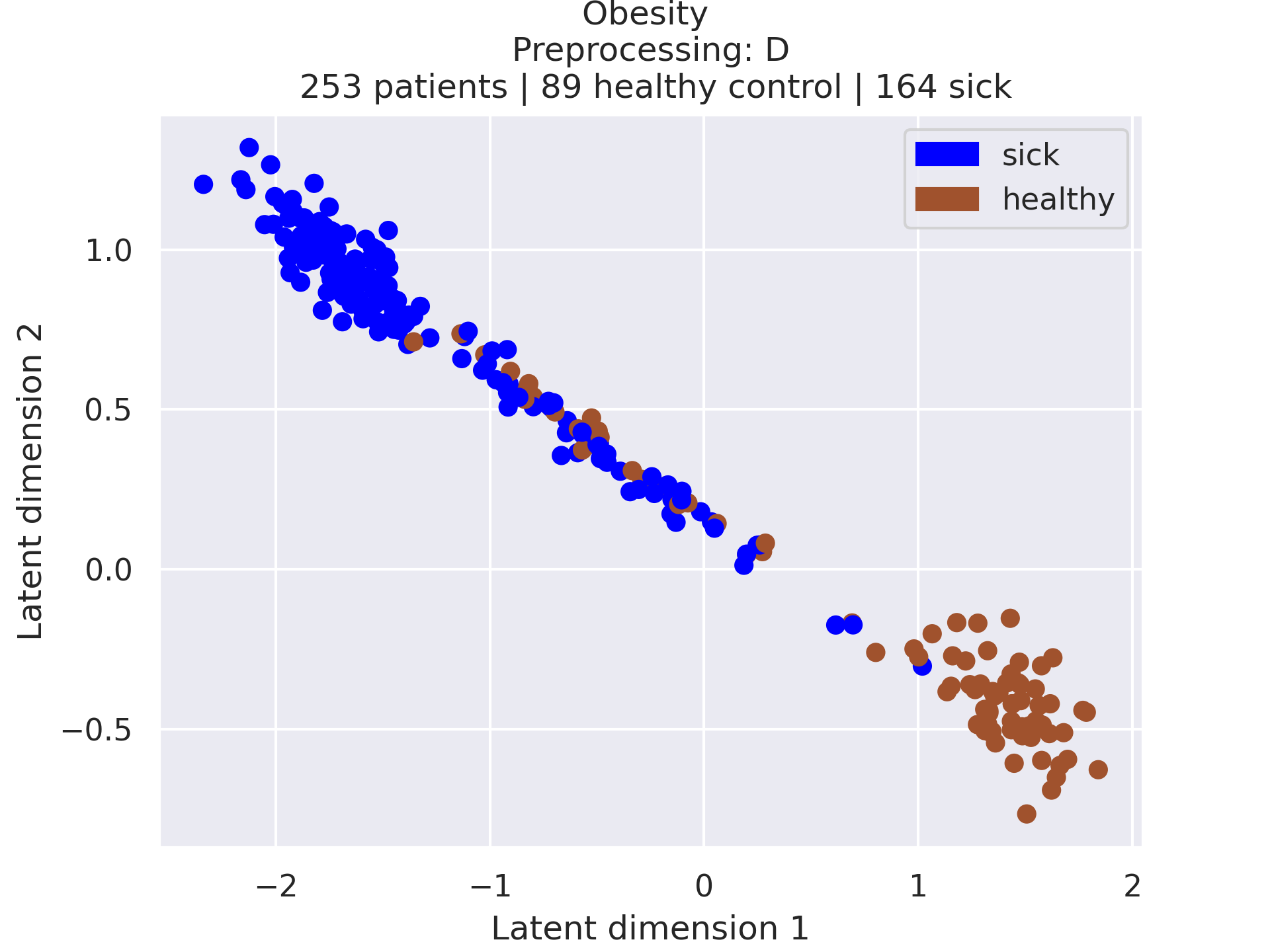

Supplement: S3 File — This file presents, for each dataset, the plots of the PCA 2D projections, as well as the plots of the mean of the MVIB 2D stochastic encodings. For the MVIB stochastic encodings z∼p(z|x)=N(μ,σ2I), the depicted points represent the mean μ. The K dimension of the latent space has been set to 2 in order to allow a 2D visualisation of the encodings. For training MVIB, the JMVIB−T objective (Eq 8) has been optimised. For MVIB, five copies of the means plots are available, as they are obtained by training the model with five different independent training-test random splits. Both the PCA and the MVIB plots have been created starting from the default datasets collection. (ZIP) [file pcbi.1010050.s008.zip › s6-file/Obesity/2_embeddings.png]

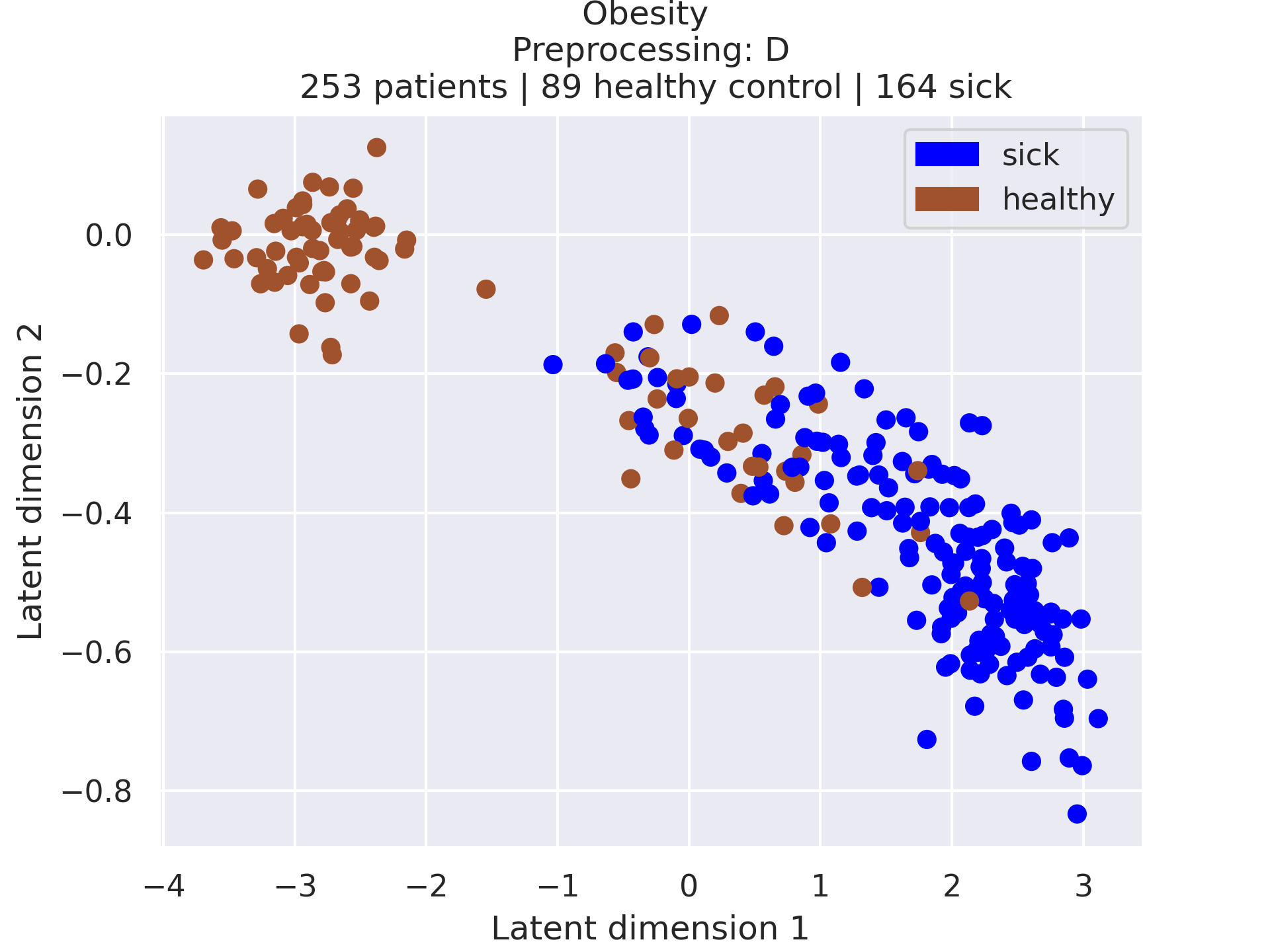

Supplement: S3 File — This file presents, for each dataset, the plots of the PCA 2D projections, as well as the plots of the mean of the MVIB 2D stochastic encodings. For the MVIB stochastic encodings z∼p(z|x)=N(μ,σ2I), the depicted points represent the mean μ. The K dimension of the latent space has been set to 2 in order to allow a 2D visualisation of the encodings. For training MVIB, the JMVIB−T objective (Eq 8) has been optimised. For MVIB, five copies of the means plots are available, as they are obtained by training the model with five different independent training-test random splits. Both the PCA and the MVIB plots have been created starting from the default datasets collection. (ZIP) [file pcbi.1010050.s008.zip › s6-file/Obesity/0_embeddings.png]

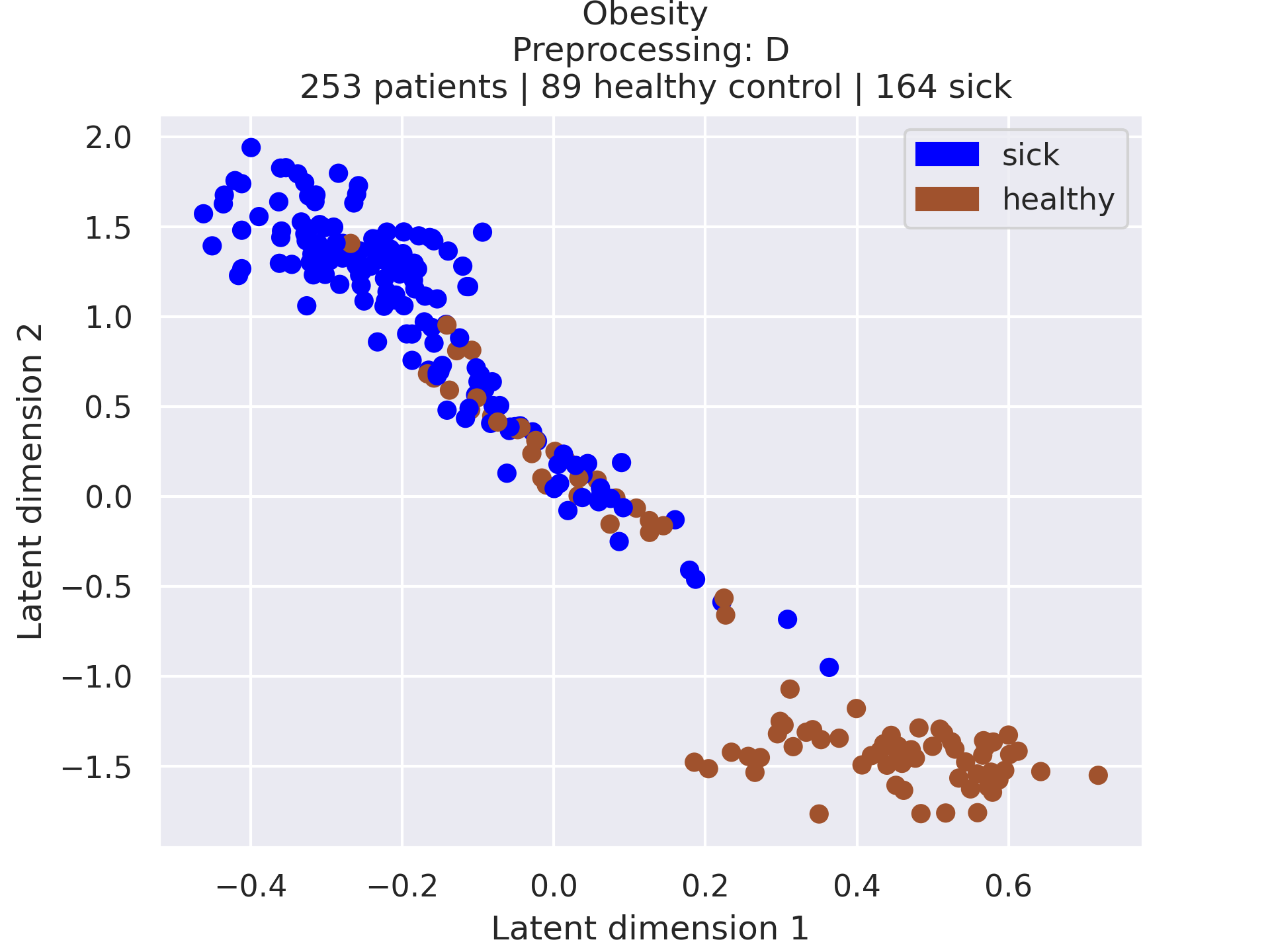

Supplement: S3 File — This file presents, for each dataset, the plots of the PCA 2D projections, as well as the plots of the mean of the MVIB 2D stochastic encodings. For the MVIB stochastic encodings z∼p(z|x)=N(μ,σ2I), the depicted points represent the mean μ. The K dimension of the latent space has been set to 2 in order to allow a 2D visualisation of the encodings. For training MVIB, the JMVIB−T objective (Eq 8) has been optimised. For MVIB, five copies of the means plots are available, as they are obtained by training the model with five different independent training-test random splits. Both the PCA and the MVIB plots have been created starting from the default datasets collection. (ZIP) [file pcbi.1010050.s008.zip › s6-file/Obesity/1_embeddings.png]

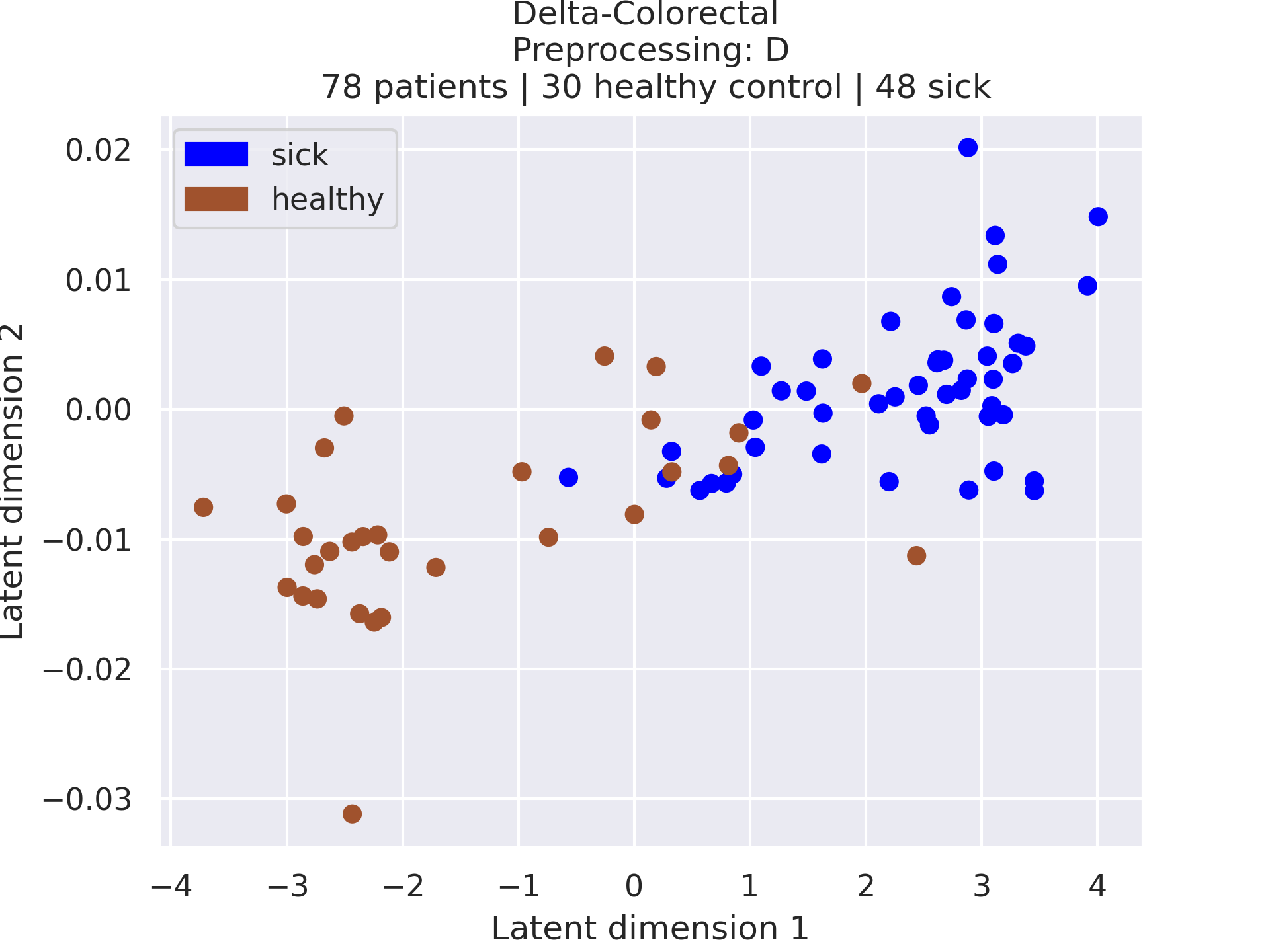

Supplement: S3 File — This file presents, for each dataset, the plots of the PCA 2D projections, as well as the plots of the mean of the MVIB 2D stochastic encodings. For the MVIB stochastic encodings z∼p(z|x)=N(μ,σ2I), the depicted points represent the mean μ. The K dimension of the latent space has been set to 2 in order to allow a 2D visualisation of the encodings. For training MVIB, the JMVIB−T objective (Eq 8) has been optimised. For MVIB, five copies of the means plots are available, as they are obtained by training the model with five different independent training-test random splits. Both the PCA and the MVIB plots have been created starting from the default datasets collection. (ZIP) [file pcbi.1010050.s008.zip › s6-file/Delta-Colorectal/3_embeddings.png]

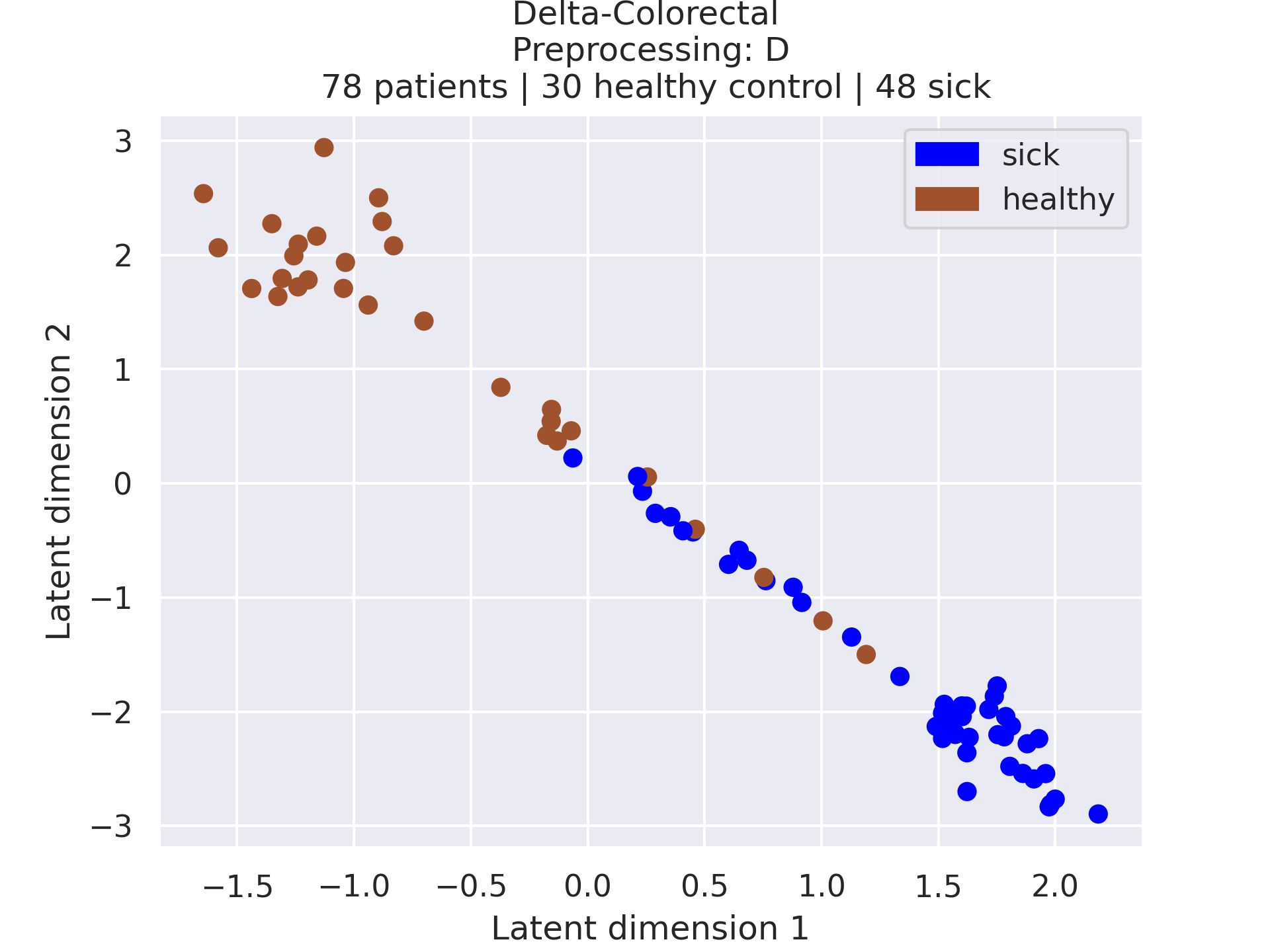

Supplement: S3 File — This file presents, for each dataset, the plots of the PCA 2D projections, as well as the plots of the mean of the MVIB 2D stochastic encodings. For the MVIB stochastic encodings z∼p(z|x)=N(μ,σ2I), the depicted points represent the mean μ. The K dimension of the latent space has been set to 2 in order to allow a 2D visualisation of the encodings. For training MVIB, the JMVIB−T objective (Eq 8) has been optimised. For MVIB, five copies of the means plots are available, as they are obtained by training the model with five different independent training-test random splits. Both the PCA and the MVIB plots have been created starting from the default datasets collection. (ZIP) [file pcbi.1010050.s008.zip › s6-file/Delta-Colorectal/4_embeddings.png]

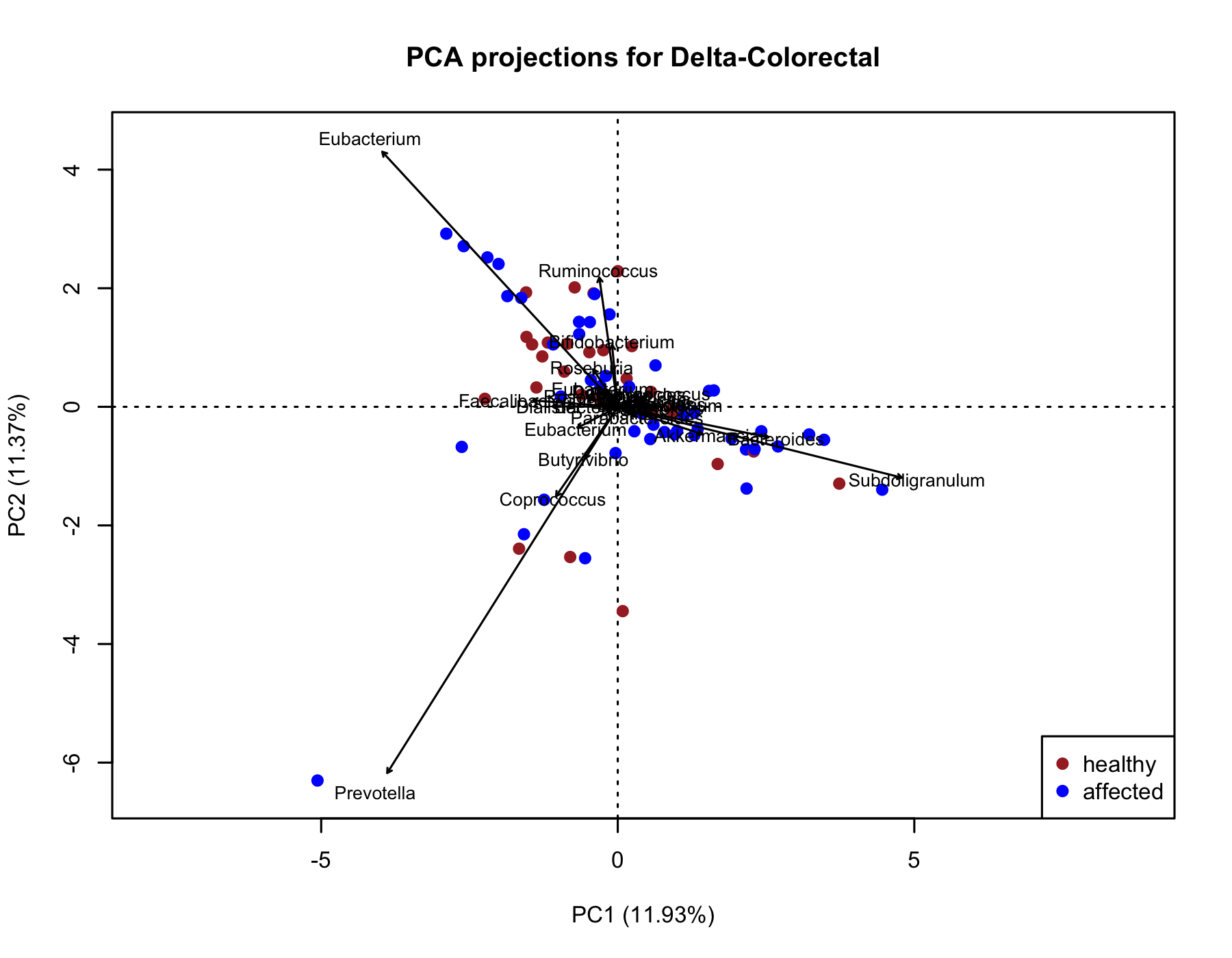

Supplement: S3 File — This file presents, for each dataset, the plots of the PCA 2D projections, as well as the plots of the mean of the MVIB 2D stochastic encodings. For the MVIB stochastic encodings z∼p(z|x)=N(μ,σ2I), the depicted points represent the mean μ. The K dimension of the latent space has been set to 2 in order to allow a 2D visualisation of the encodings. For training MVIB, the JMVIB−T objective (Eq 8) has been optimised. For MVIB, five copies of the means plots are available, as they are obtained by training the model with five different independent training-test random splits. Both the PCA and the MVIB plots have been created starting from the default datasets collection. (ZIP) [file pcbi.1010050.s008.zip › s6-file/Delta-Colorectal/PCA_projections.png]

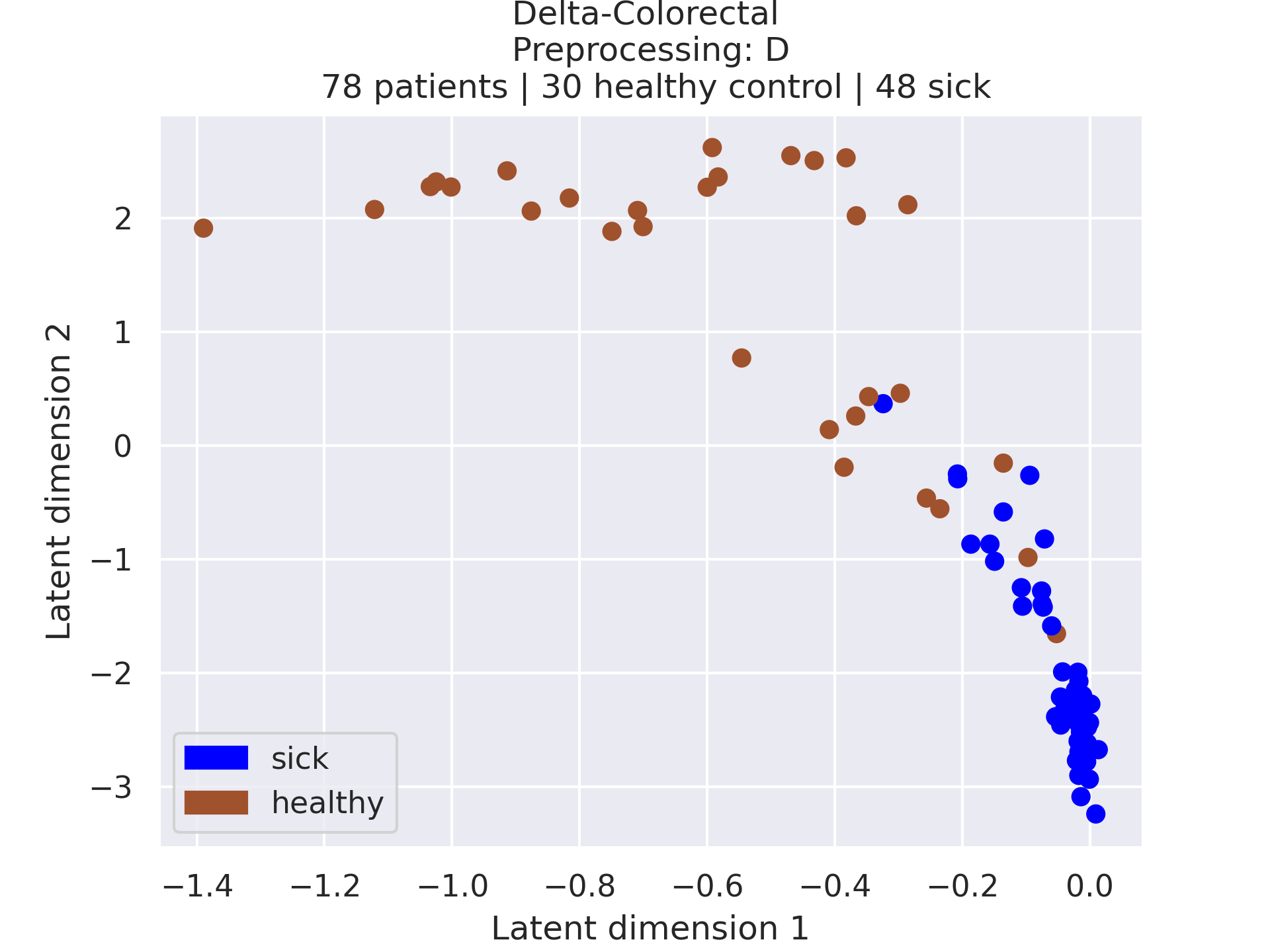

Supplement: S3 File — This file presents, for each dataset, the plots of the PCA 2D projections, as well as the plots of the mean of the MVIB 2D stochastic encodings. For the MVIB stochastic encodings z∼p(z|x)=N(μ,σ2I), the depicted points represent the mean μ. The K dimension of the latent space has been set to 2 in order to allow a 2D visualisation of the encodings. For training MVIB, the JMVIB−T objective (Eq 8) has been optimised. For MVIB, five copies of the means plots are available, as they are obtained by training the model with five different independent training-test random splits. Both the PCA and the MVIB plots have been created starting from the default datasets collection. (ZIP) [file pcbi.1010050.s008.zip › s6-file/Delta-Colorectal/2_embeddings.png]

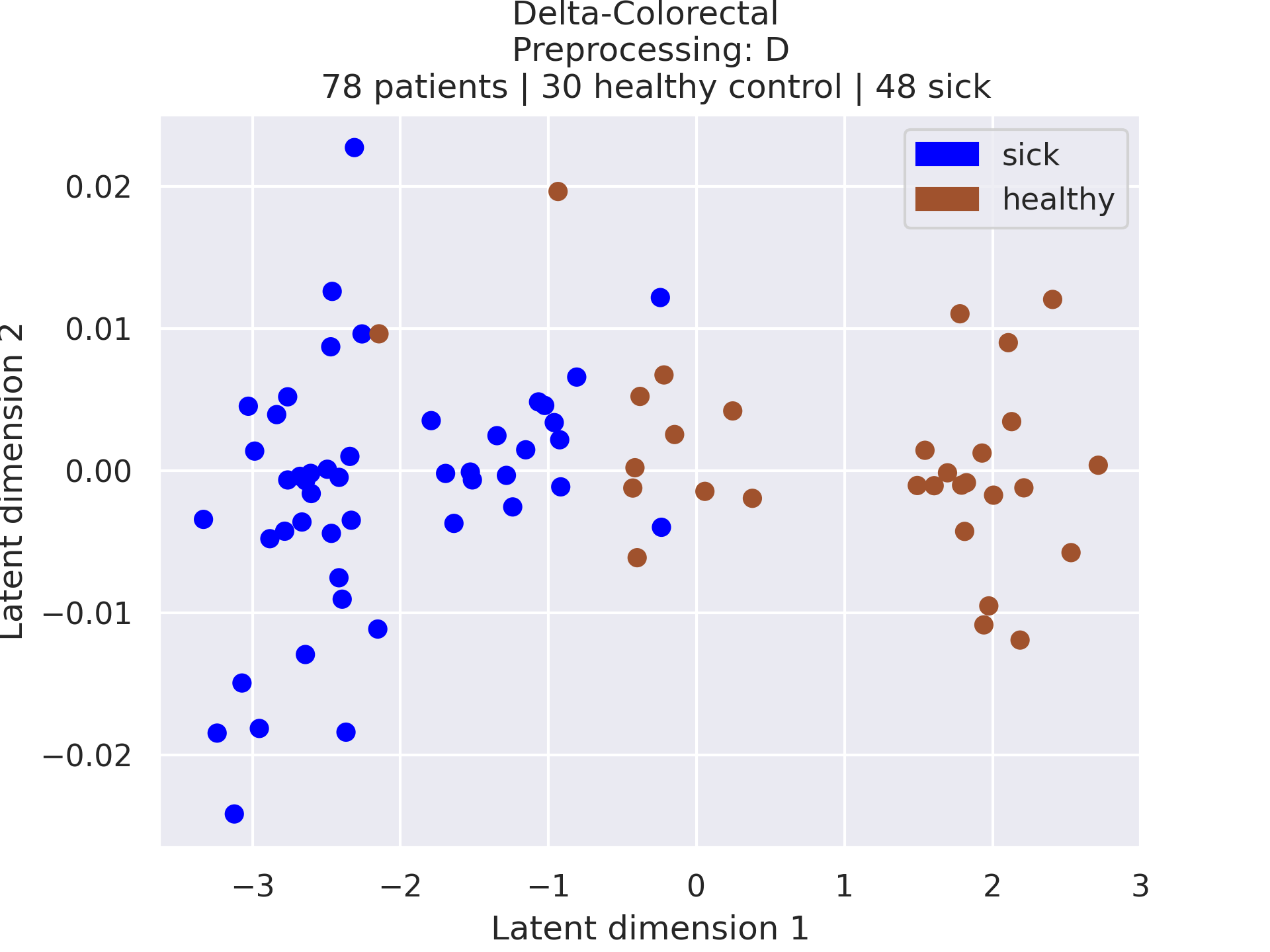

Supplement: S3 File — This file presents, for each dataset, the plots of the PCA 2D projections, as well as the plots of the mean of the MVIB 2D stochastic encodings. For the MVIB stochastic encodings z∼p(z|x)=N(μ,σ2I), the depicted points represent the mean μ. The K dimension of the latent space has been set to 2 in order to allow a 2D visualisation of the encodings. For training MVIB, the JMVIB−T objective (Eq 8) has been optimised. For MVIB, five copies of the means plots are available, as they are obtained by training the model with five different independent training-test random splits. Both the PCA and the MVIB plots have been created starting from the default datasets collection. (ZIP) [file pcbi.1010050.s008.zip › s6-file/Delta-Colorectal/0_embeddings.png]

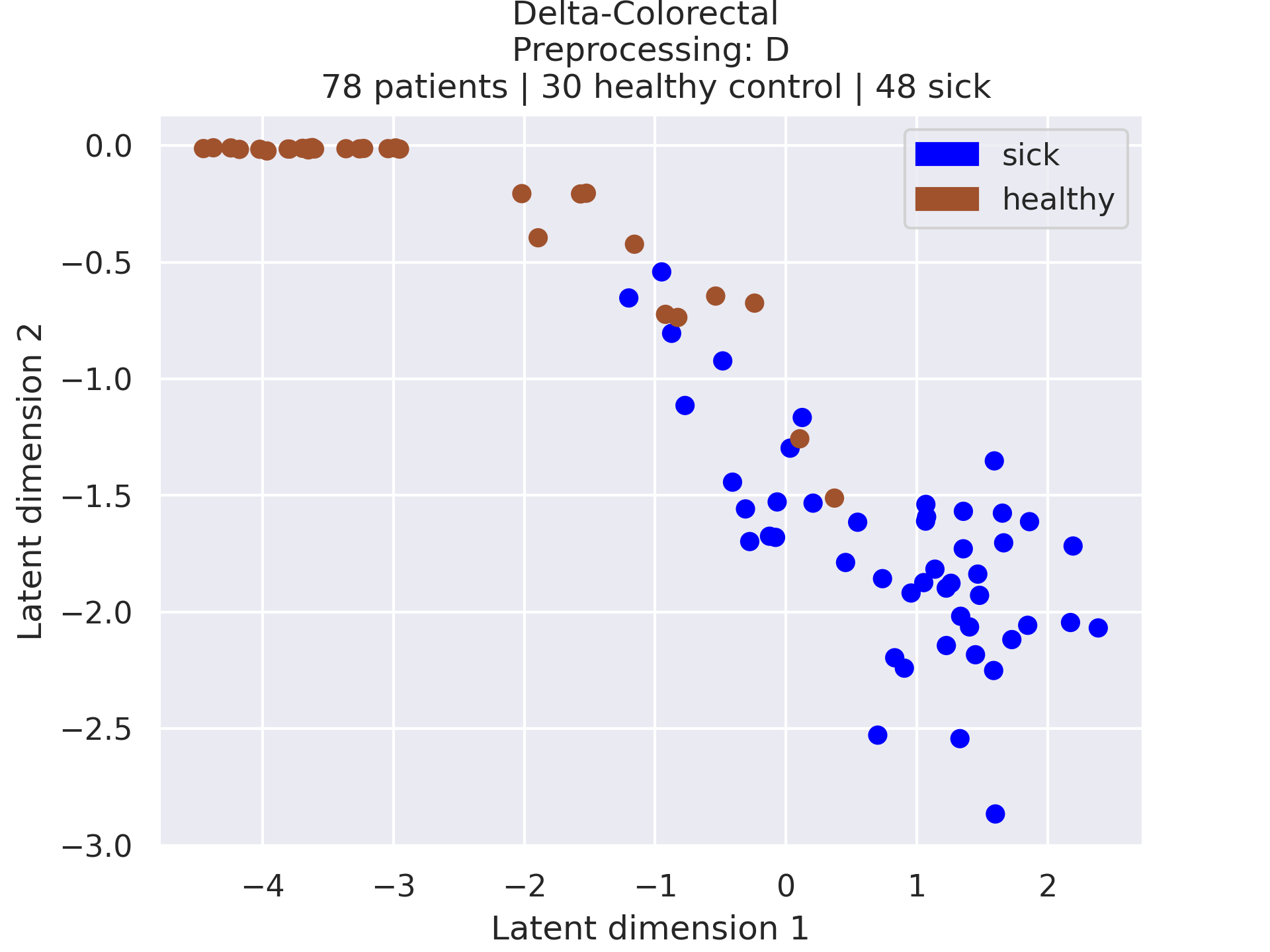

Supplement: S3 File — This file presents, for each dataset, the plots of the PCA 2D projections, as well as the plots of the mean of the MVIB 2D stochastic encodings. For the MVIB stochastic encodings z∼p(z|x)=N(μ,σ2I), the depicted points represent the mean μ. The K dimension of the latent space has been set to 2 in order to allow a 2D visualisation of the encodings. For training MVIB, the JMVIB−T objective (Eq 8) has been optimised. For MVIB, five copies of the means plots are available, as they are obtained by training the model with five different independent training-test random splits. Both the PCA and the MVIB plots have been created starting from the default datasets collection. (ZIP) [file pcbi.1010050.s008.zip › s6-file/Delta-Colorectal/1_embeddings.png]

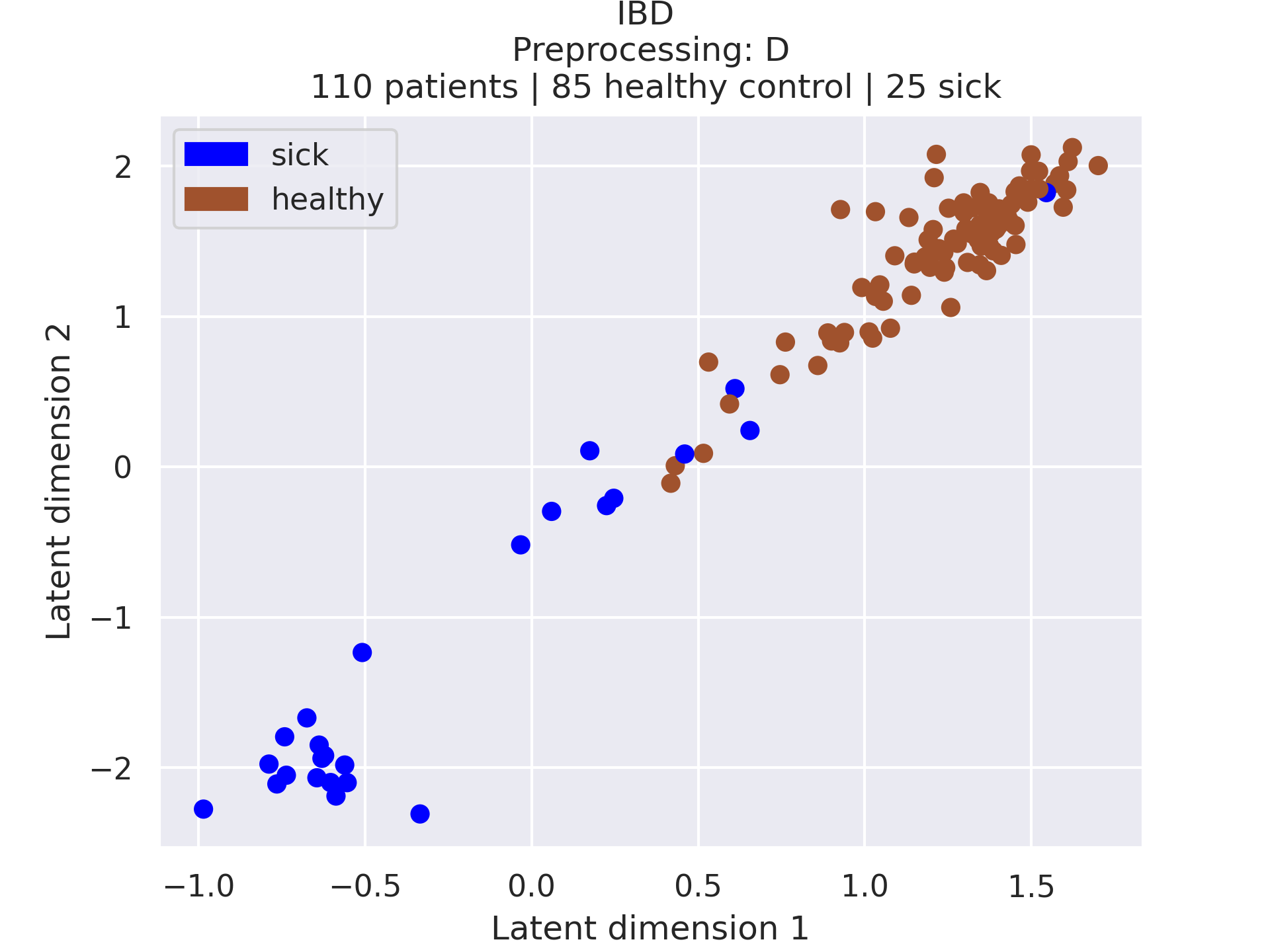

Supplement: S3 File — This file presents, for each dataset, the plots of the PCA 2D projections, as well as the plots of the mean of the MVIB 2D stochastic encodings. For the MVIB stochastic encodings z∼p(z|x)=N(μ,σ2I), the depicted points represent the mean μ. The K dimension of the latent space has been set to 2 in order to allow a 2D visualisation of the encodings. For training MVIB, the JMVIB−T objective (Eq 8) has been optimised. For MVIB, five copies of the means plots are available, as they are obtained by training the model with five different independent training-test random splits. Both the PCA and the MVIB plots have been created starting from the default datasets collection. (ZIP) [file pcbi.1010050.s008.zip › s6-file/IBD/3_embeddings.png]

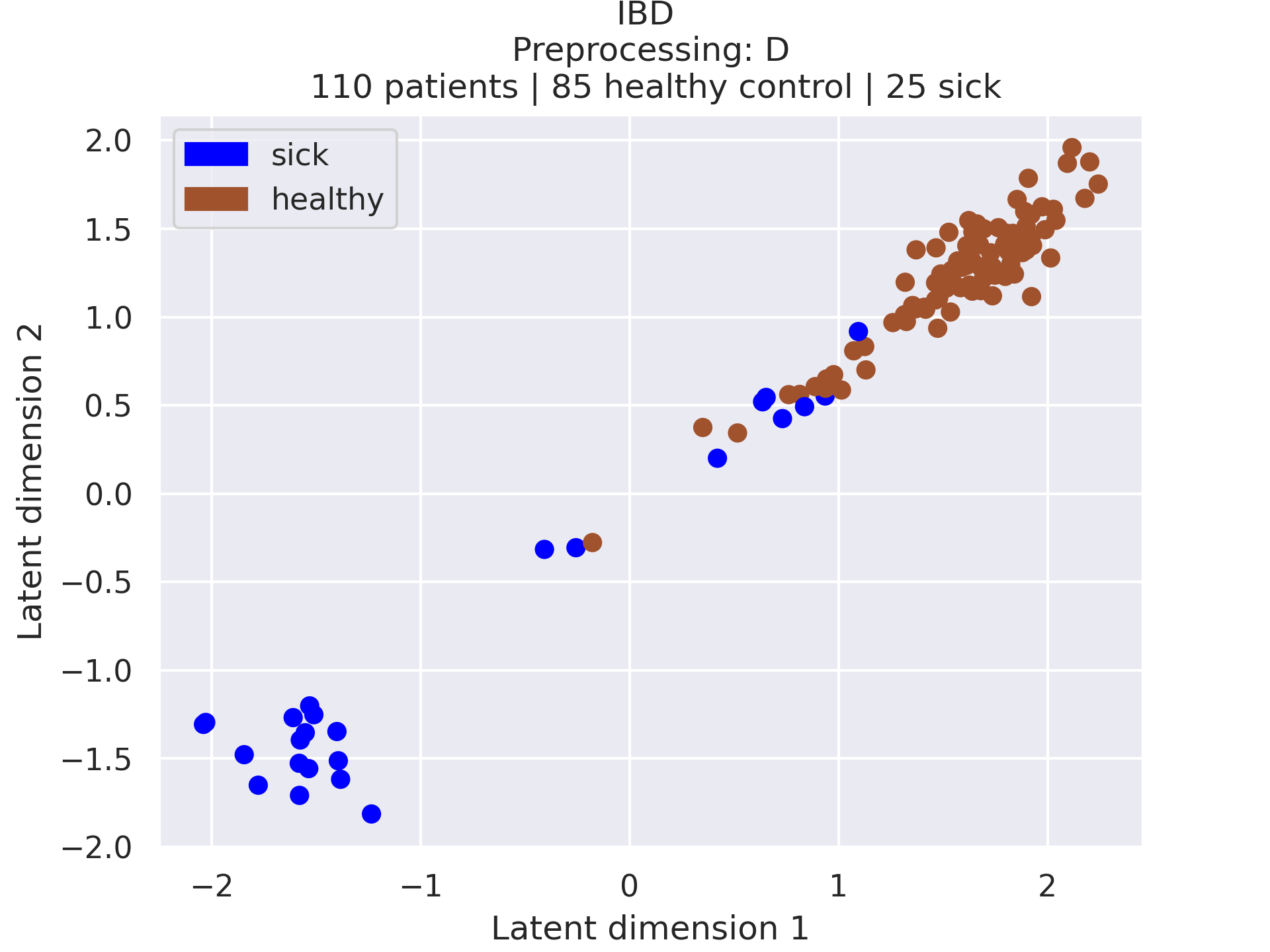

Supplement: S3 File — This file presents, for each dataset, the plots of the PCA 2D projections, as well as the plots of the mean of the MVIB 2D stochastic encodings. For the MVIB stochastic encodings z∼p(z|x)=N(μ,σ2I), the depicted points represent the mean μ. The K dimension of the latent space has been set to 2 in order to allow a 2D visualisation of the encodings. For training MVIB, the JMVIB−T objective (Eq 8) has been optimised. For MVIB, five copies of the means plots are available, as they are obtained by training the model with five different independent training-test random splits. Both the PCA and the MVIB plots have been created starting from the default datasets collection. (ZIP) [file pcbi.1010050.s008.zip › s6-file/IBD/4_embeddings.png]

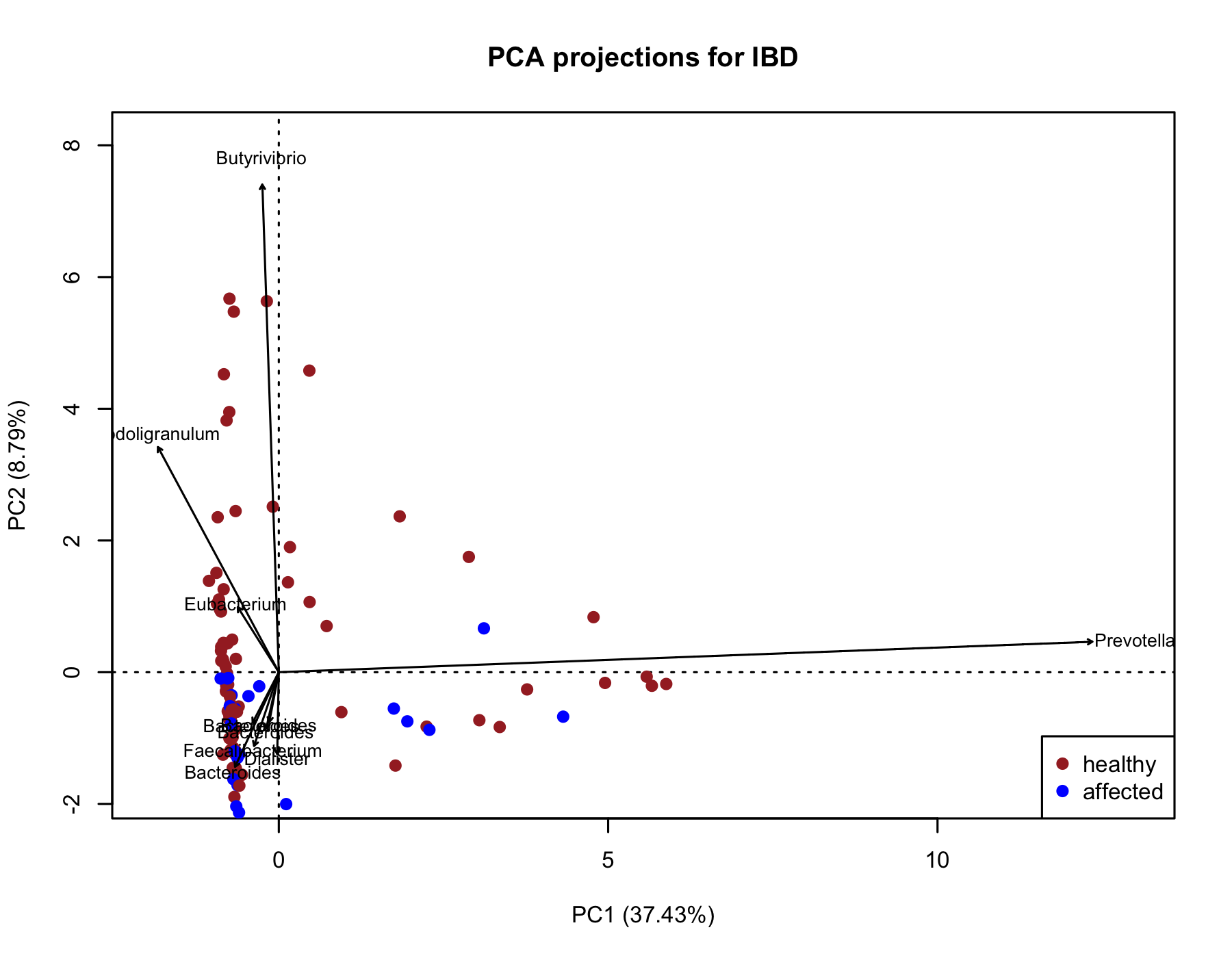

Supplement: S3 File — This file presents, for each dataset, the plots of the PCA 2D projections, as well as the plots of the mean of the MVIB 2D stochastic encodings. For the MVIB stochastic encodings z∼p(z|x)=N(μ,σ2I), the depicted points represent the mean μ. The K dimension of the latent space has been set to 2 in order to allow a 2D visualisation of the encodings. For training MVIB, the JMVIB−T objective (Eq 8) has been optimised. For MVIB, five copies of the means plots are available, as they are obtained by training the model with five different independent training-test random splits. Both the PCA and the MVIB plots have been created starting from the default datasets collection. (ZIP) [file pcbi.1010050.s008.zip › s6-file/IBD/PCA_projections.png]

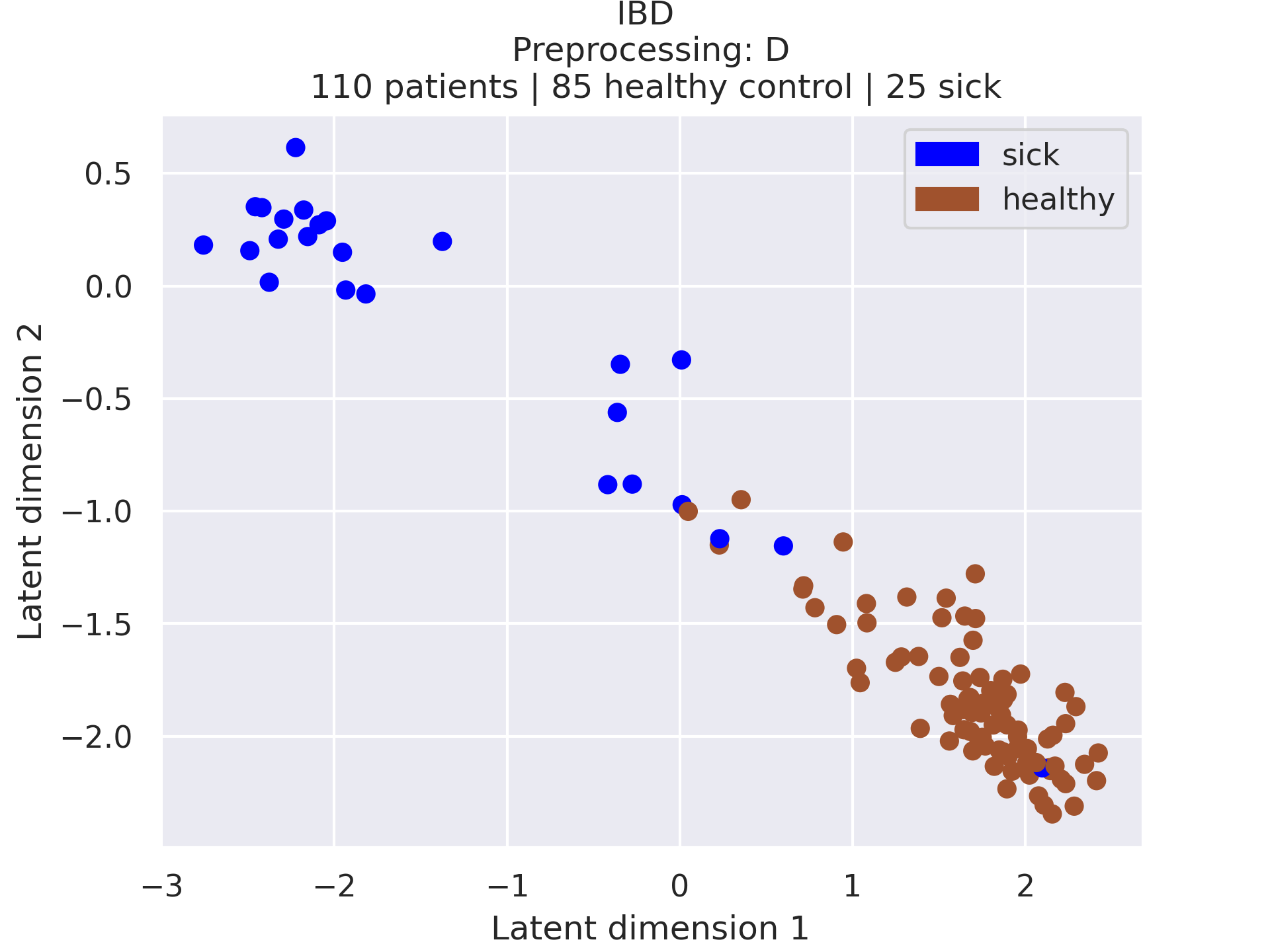

Supplement: S3 File — This file presents, for each dataset, the plots of the PCA 2D projections, as well as the plots of the mean of the MVIB 2D stochastic encodings. For the MVIB stochastic encodings z∼p(z|x)=N(μ,σ2I), the depicted points represent the mean μ. The K dimension of the latent space has been set to 2 in order to allow a 2D visualisation of the encodings. For training MVIB, the JMVIB−T objective (Eq 8) has been optimised. For MVIB, five copies of the means plots are available, as they are obtained by training the model with five different independent training-test random splits. Both the PCA and the MVIB plots have been created starting from the default datasets collection. (ZIP) [file pcbi.1010050.s008.zip › s6-file/IBD/2_embeddings.png]

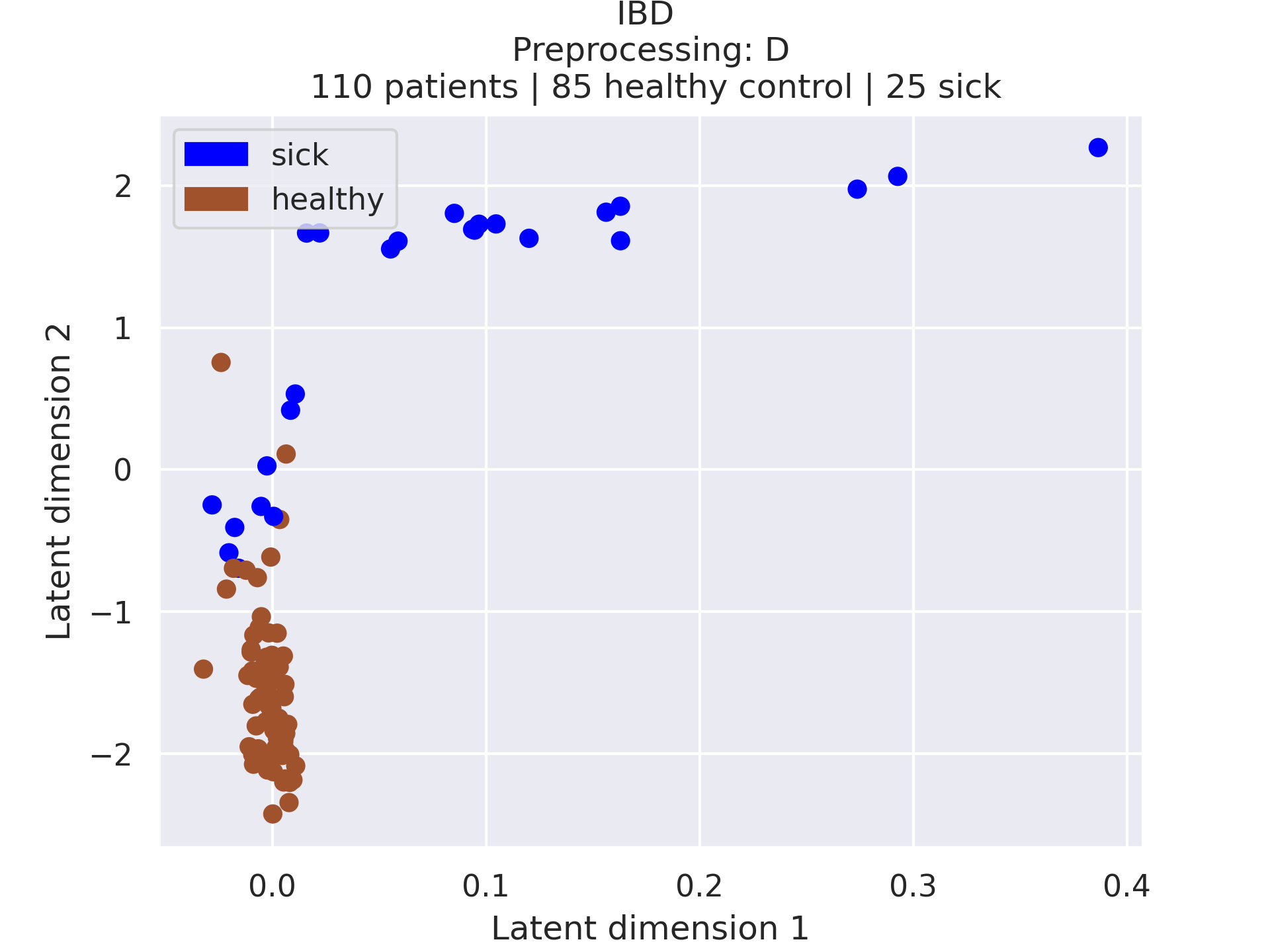

Supplement: S3 File — This file presents, for each dataset, the plots of the PCA 2D projections, as well as the plots of the mean of the MVIB 2D stochastic encodings. For the MVIB stochastic encodings z∼p(z|x)=N(μ,σ2I), the depicted points represent the mean μ. The K dimension of the latent space has been set to 2 in order to allow a 2D visualisation of the encodings. For training MVIB, the JMVIB−T objective (Eq 8) has been optimised. For MVIB, five copies of the means plots are available, as they are obtained by training the model with five different independent training-test random splits. Both the PCA and the MVIB plots have been created starting from the default datasets collection. (ZIP) [file pcbi.1010050.s008.zip › s6-file/IBD/0_embeddings.png]

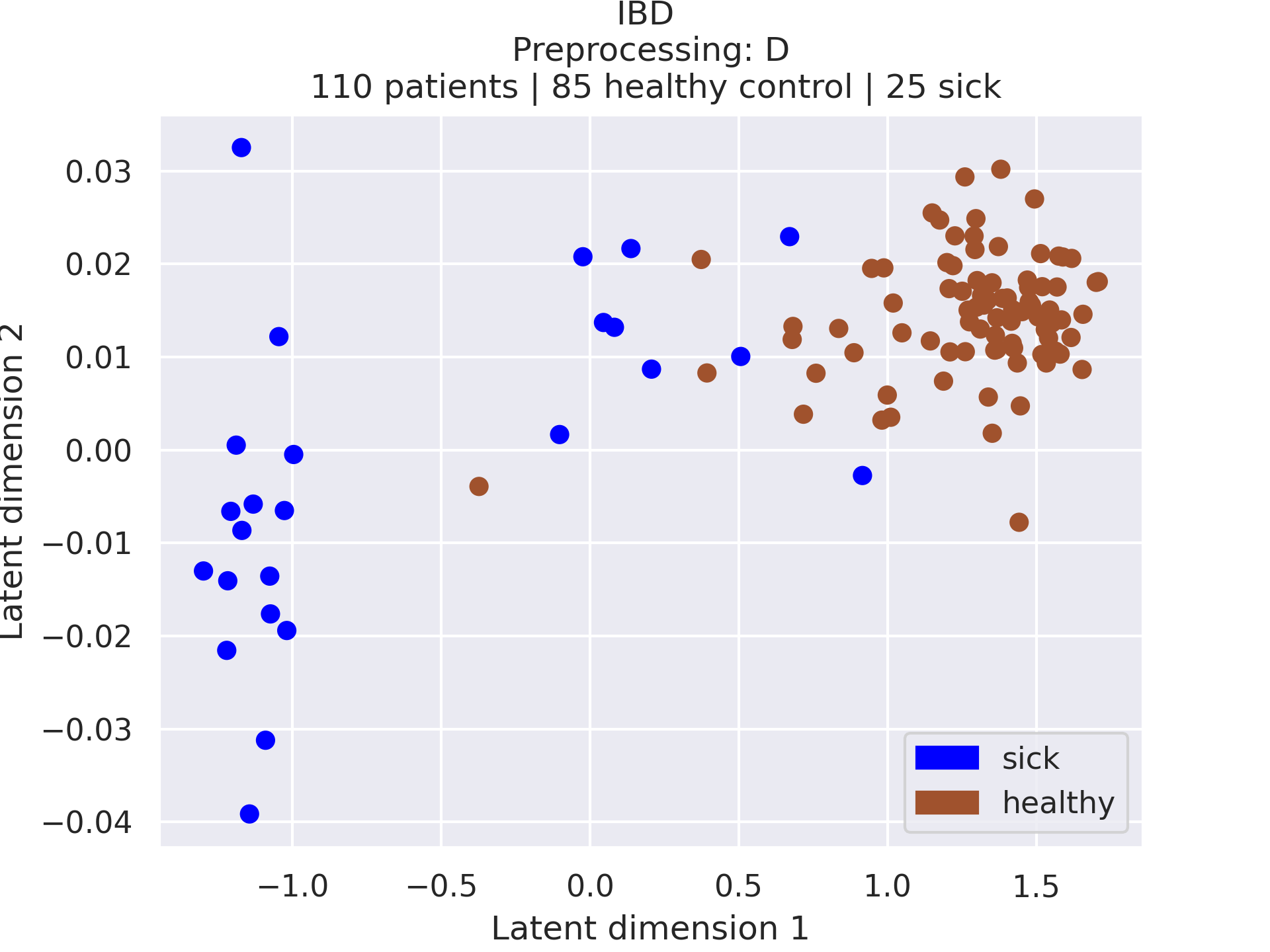

Supplement: S3 File — This file presents, for each dataset, the plots of the PCA 2D projections, as well as the plots of the mean of the MVIB 2D stochastic encodings. For the MVIB stochastic encodings z∼p(z|x)=N(μ,σ2I), the depicted points represent the mean μ. The K dimension of the latent space has been set to 2 in order to allow a 2D visualisation of the encodings. For training MVIB, the JMVIB−T objective (Eq 8) has been optimised. For MVIB, five copies of the means plots are available, as they are obtained by training the model with five different independent training-test random splits. Both the PCA and the MVIB plots have been created starting from the default datasets collection. (ZIP) [file pcbi.1010050.s008.zip › s6-file/IBD/1_embeddings.png]

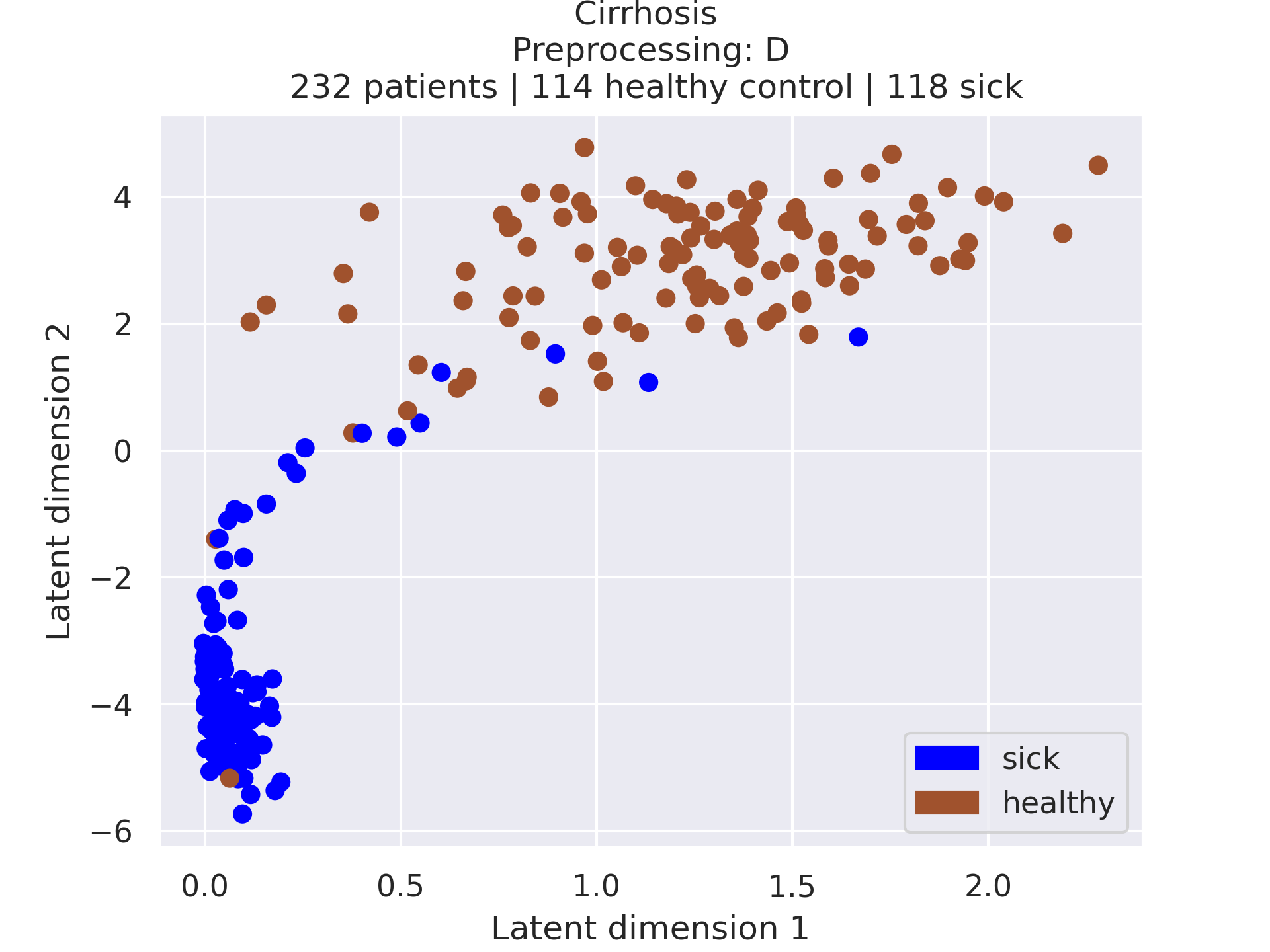

Supplement: S3 File — This file presents, for each dataset, the plots of the PCA 2D projections, as well as the plots of the mean of the MVIB 2D stochastic encodings. For the MVIB stochastic encodings z∼p(z|x)=N(μ,σ2I), the depicted points represent the mean μ. The K dimension of the latent space has been set to 2 in order to allow a 2D visualisation of the encodings. For training MVIB, the JMVIB−T objective (Eq 8) has been optimised. For MVIB, five copies of the means plots are available, as they are obtained by training the model with five different independent training-test random splits. Both the PCA and the MVIB plots have been created starting from the default datasets collection. (ZIP) [file pcbi.1010050.s008.zip › s6-file/Cirrhosis/3_embeddings.png]

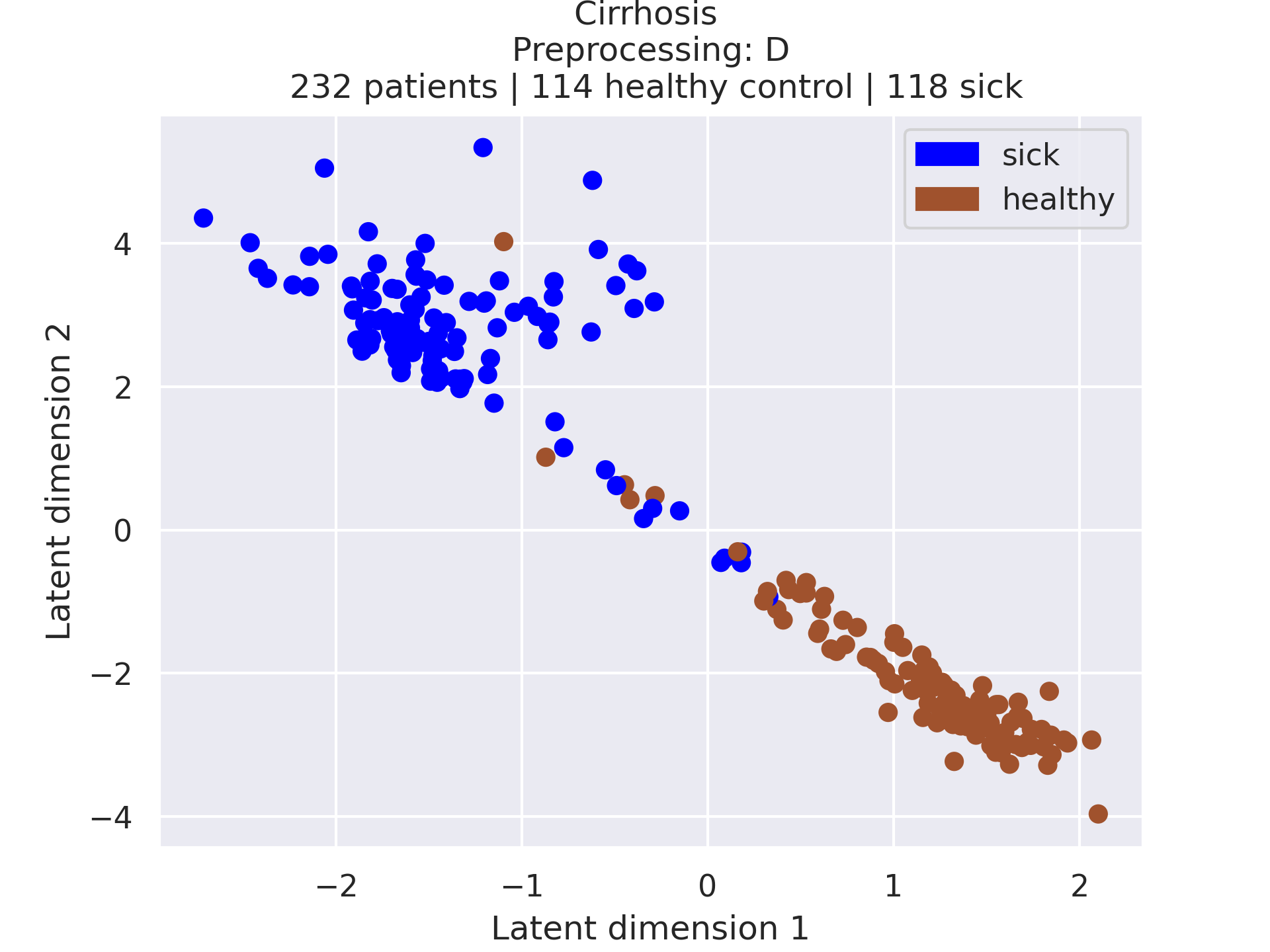

Supplement: S3 File — This file presents, for each dataset, the plots of the PCA 2D projections, as well as the plots of the mean of the MVIB 2D stochastic encodings. For the MVIB stochastic encodings z∼p(z|x)=N(μ,σ2I), the depicted points represent the mean μ. The K dimension of the latent space has been set to 2 in order to allow a 2D visualisation of the encodings. For training MVIB, the JMVIB−T objective (Eq 8) has been optimised. For MVIB, five copies of the means plots are available, as they are obtained by training the model with five different independent training-test random splits. Both the PCA and the MVIB plots have been created starting from the default datasets collection. (ZIP) [file pcbi.1010050.s008.zip › s6-file/Cirrhosis/4_embeddings.png]

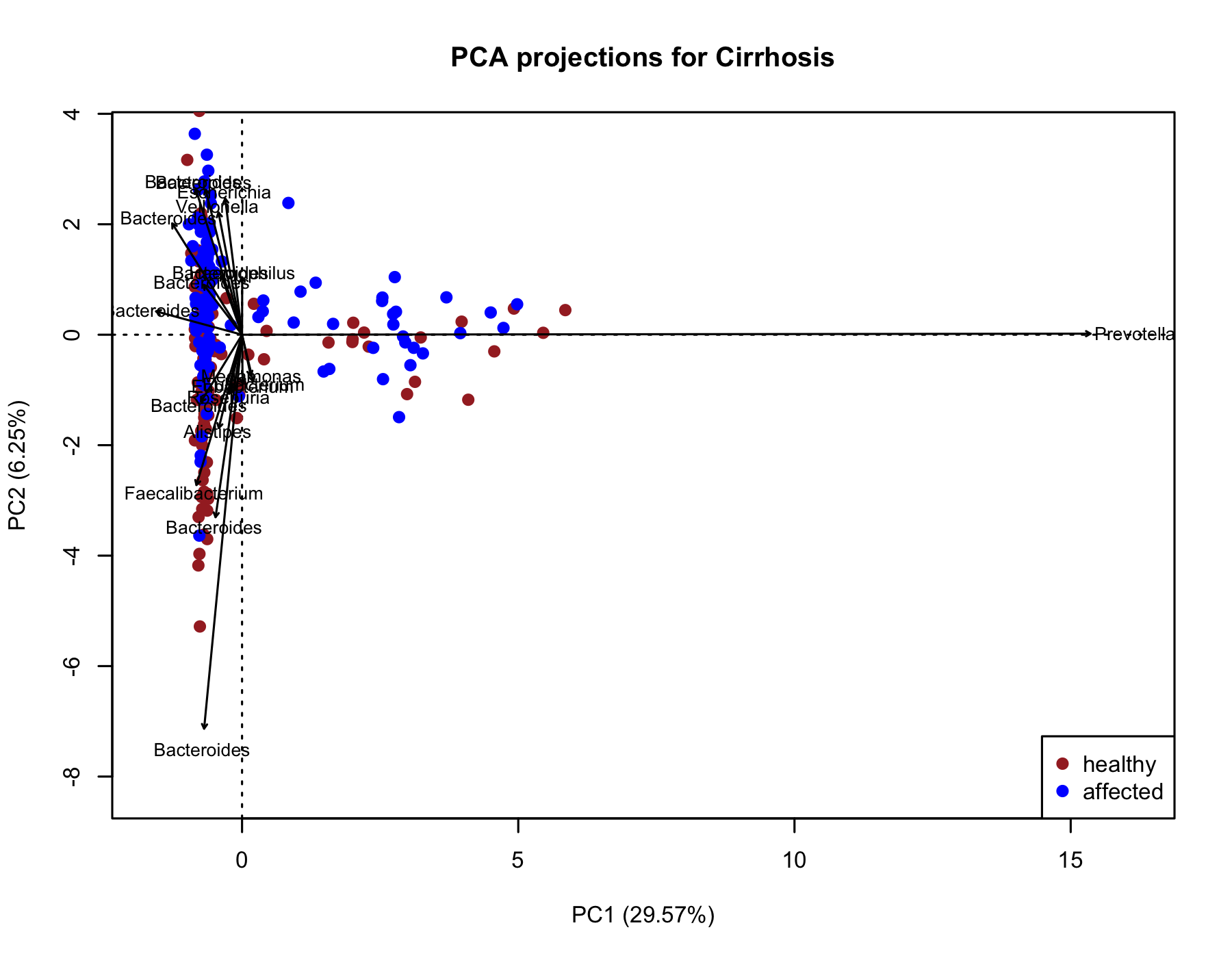

Supplement: S3 File — This file presents, for each dataset, the plots of the PCA 2D projections, as well as the plots of the mean of the MVIB 2D stochastic encodings. For the MVIB stochastic encodings z∼p(z|x)=N(μ,σ2I), the depicted points represent the mean μ. The K dimension of the latent space has been set to 2 in order to allow a 2D visualisation of the encodings. For training MVIB, the JMVIB−T objective (Eq 8) has been optimised. For MVIB, five copies of the means plots are available, as they are obtained by training the model with five different independent training-test random splits. Both the PCA and the MVIB plots have been created starting from the default datasets collection. (ZIP) [file pcbi.1010050.s008.zip › s6-file/Cirrhosis/PCA_projections.png]

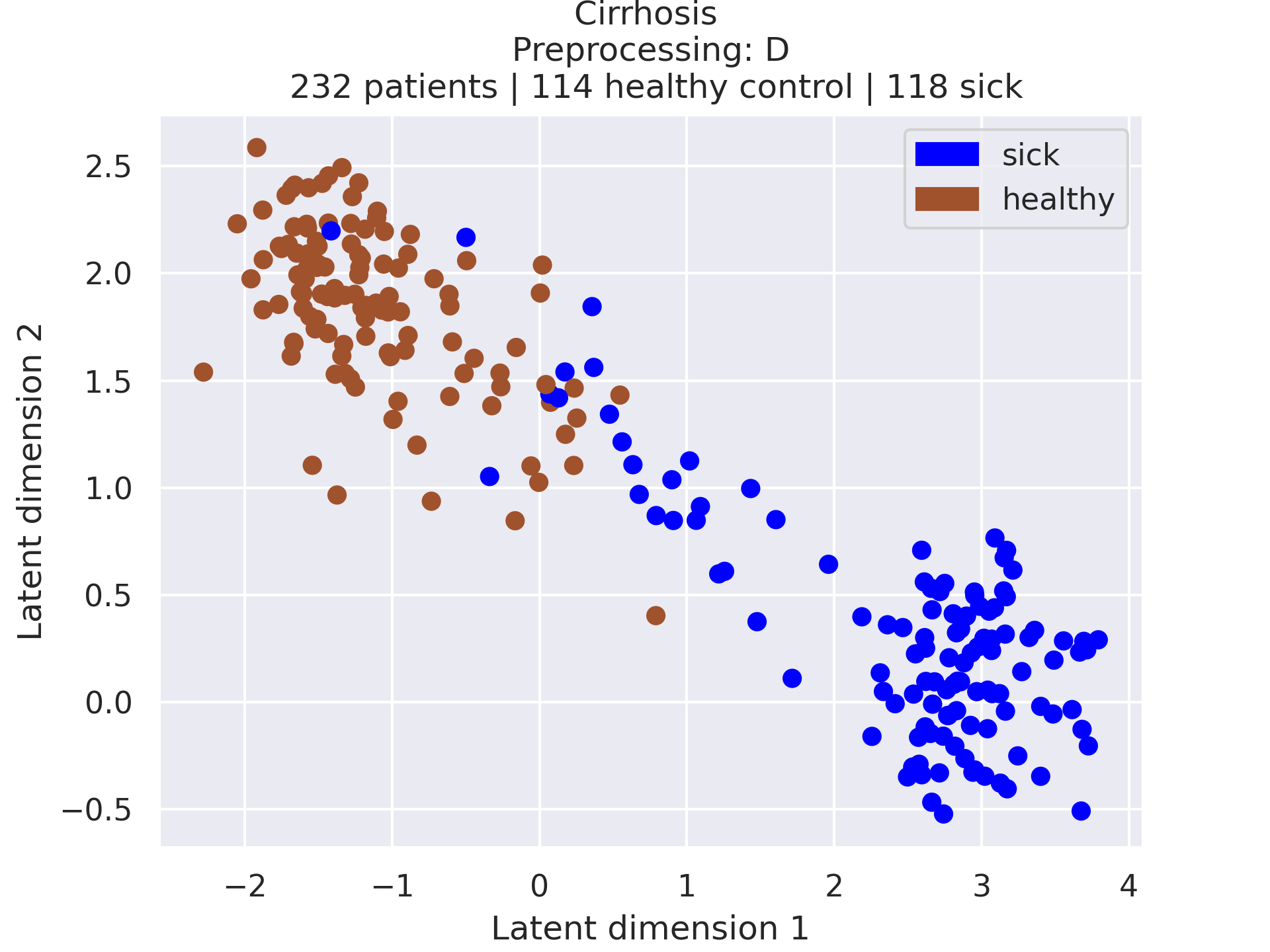

Supplement: S3 File — This file presents, for each dataset, the plots of the PCA 2D projections, as well as the plots of the mean of the MVIB 2D stochastic encodings. For the MVIB stochastic encodings z∼p(z|x)=N(μ,σ2I), the depicted points represent the mean μ. The K dimension of the latent space has been set to 2 in order to allow a 2D visualisation of the encodings. For training MVIB, the JMVIB−T objective (Eq 8) has been optimised. For MVIB, five copies of the means plots are available, as they are obtained by training the model with five different independent training-test random splits. Both the PCA and the MVIB plots have been created starting from the default datasets collection. (ZIP) [file pcbi.1010050.s008.zip › s6-file/Cirrhosis/2_embeddings.png]

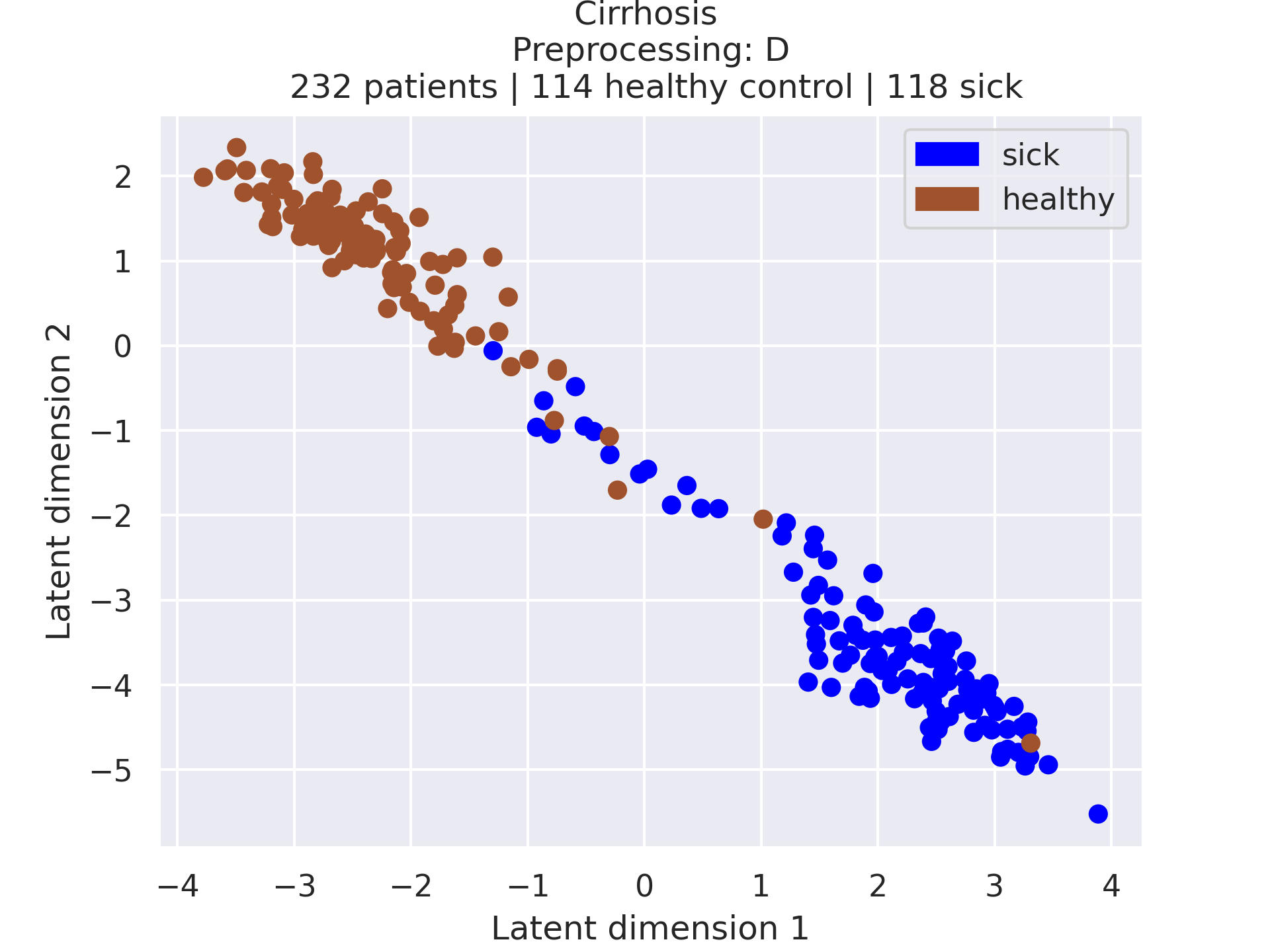

Supplement: S3 File — This file presents, for each dataset, the plots of the PCA 2D projections, as well as the plots of the mean of the MVIB 2D stochastic encodings. For the MVIB stochastic encodings z∼p(z|x)=N(μ,σ2I), the depicted points represent the mean μ. The K dimension of the latent space has been set to 2 in order to allow a 2D visualisation of the encodings. For training MVIB, the JMVIB−T objective (Eq 8) has been optimised. For MVIB, five copies of the means plots are available, as they are obtained by training the model with five different independent training-test random splits. Both the PCA and the MVIB plots have been created starting from the default datasets collection. (ZIP) [file pcbi.1010050.s008.zip › s6-file/Cirrhosis/0_embeddings.png]

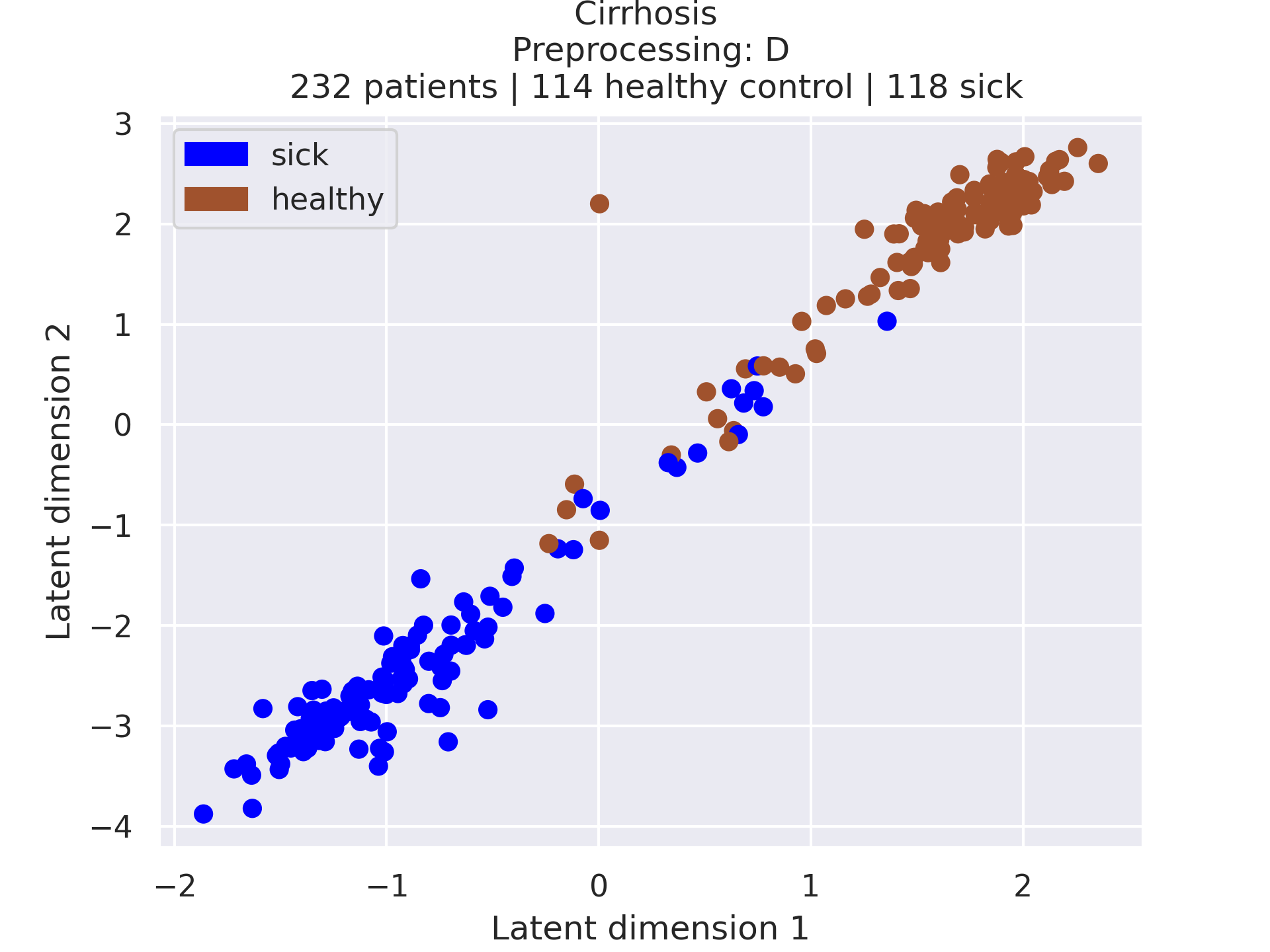

Supplement: S3 File — This file presents, for each dataset, the plots of the PCA 2D projections, as well as the plots of the mean of the MVIB 2D stochastic encodings. For the MVIB stochastic encodings z∼p(z|x)=N(μ,σ2I), the depicted points represent the mean μ. The K dimension of the latent space has been set to 2 in order to allow a 2D visualisation of the encodings. For training MVIB, the JMVIB−T objective (Eq 8) has been optimised. For MVIB, five copies of the means plots are available, as they are obtained by training the model with five different independent training-test random splits. Both the PCA and the MVIB plots have been created starting from the default datasets collection. (ZIP) [file pcbi.1010050.s008.zip › s6-file/Cirrhosis/1_embeddings.png]

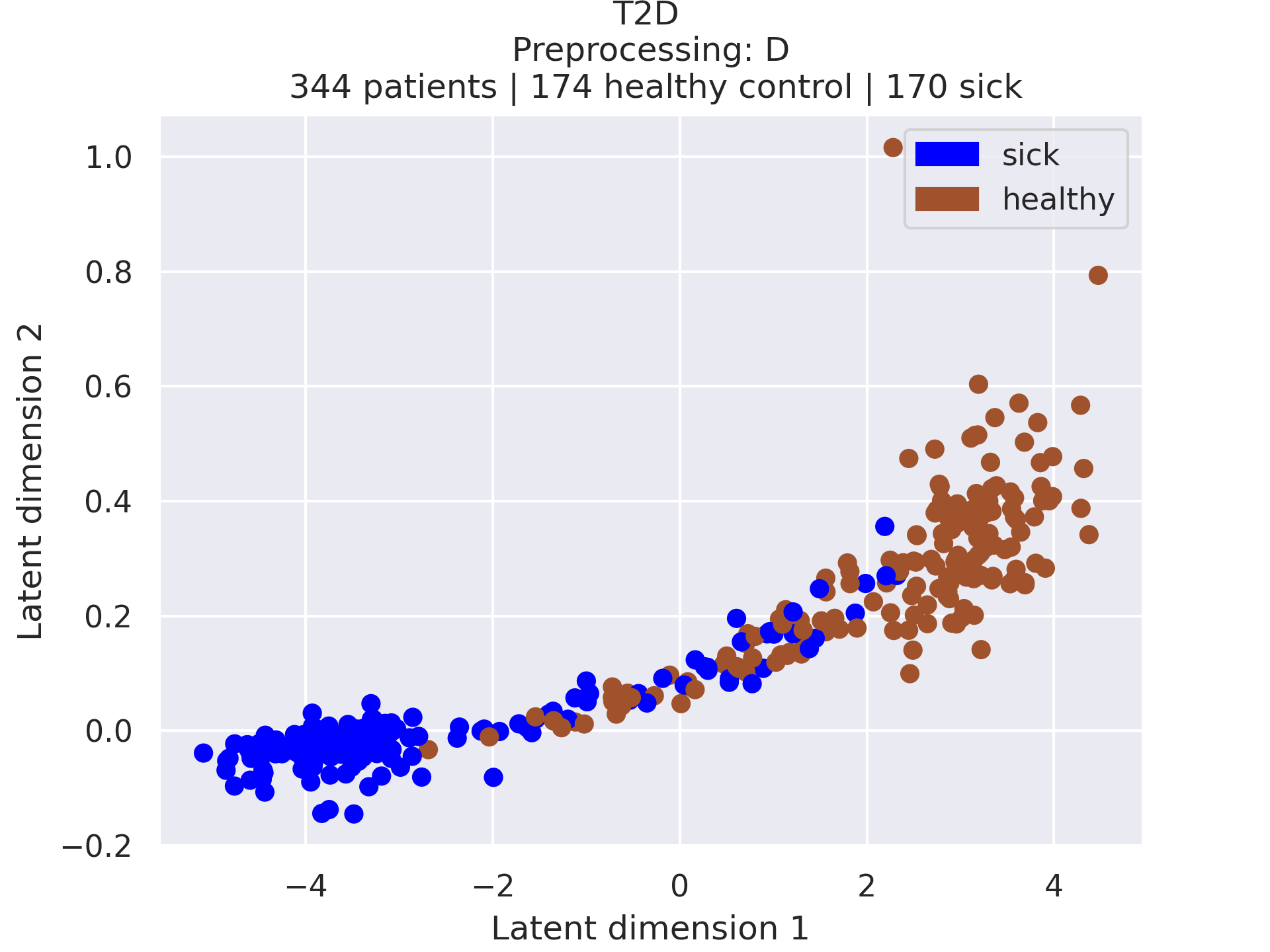

Supplement: S3 File — This file presents, for each dataset, the plots of the PCA 2D projections, as well as the plots of the mean of the MVIB 2D stochastic encodings. For the MVIB stochastic encodings z∼p(z|x)=N(μ,σ2I), the depicted points represent the mean μ. The K dimension of the latent space has been set to 2 in order to allow a 2D visualisation of the encodings. For training MVIB, the JMVIB−T objective (Eq 8) has been optimised. For MVIB, five copies of the means plots are available, as they are obtained by training the model with five different independent training-test random splits. Both the PCA and the MVIB plots have been created starting from the default datasets collection. (ZIP) [file pcbi.1010050.s008.zip › s6-file/T2D/3_embeddings.png]

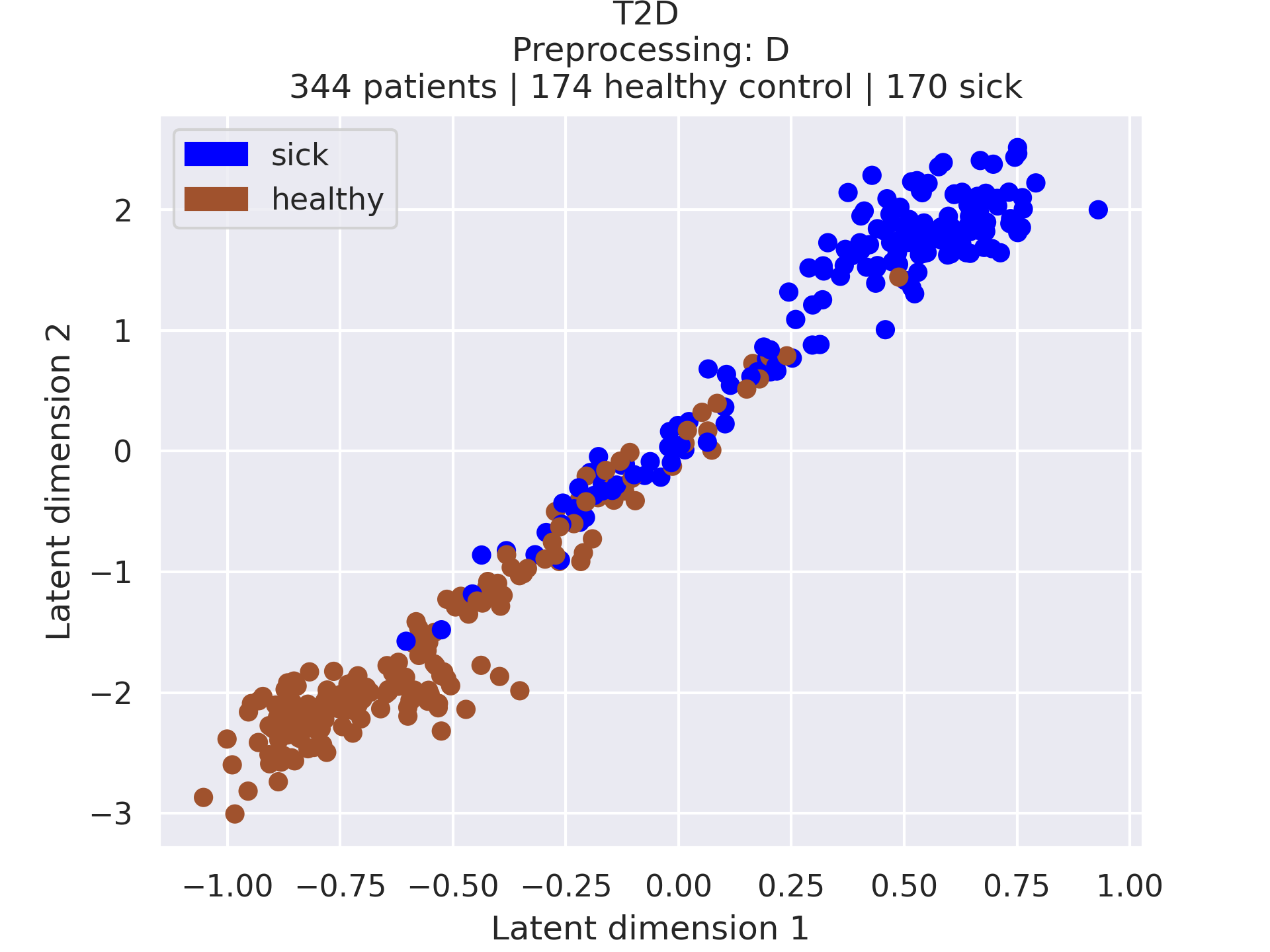

Supplement: S3 File — This file presents, for each dataset, the plots of the PCA 2D projections, as well as the plots of the mean of the MVIB 2D stochastic encodings. For the MVIB stochastic encodings z∼p(z|x)=N(μ,σ2I), the depicted points represent the mean μ. The K dimension of the latent space has been set to 2 in order to allow a 2D visualisation of the encodings. For training MVIB, the JMVIB−T objective (Eq 8) has been optimised. For MVIB, five copies of the means plots are available, as they are obtained by training the model with five different independent training-test random splits. Both the PCA and the MVIB plots have been created starting from the default datasets collection. (ZIP) [file pcbi.1010050.s008.zip › s6-file/T2D/4_embeddings.png]

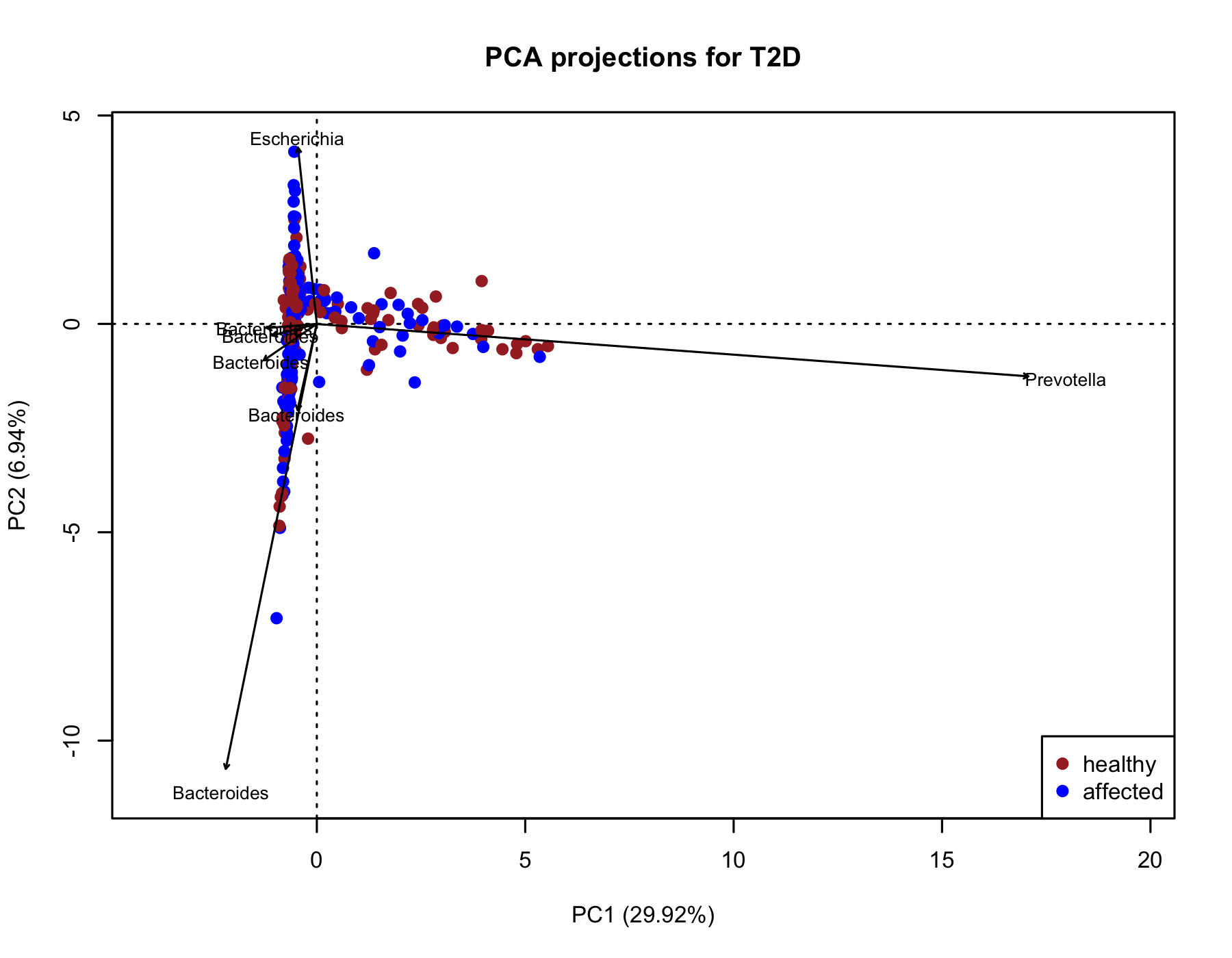

Supplement: S3 File — This file presents, for each dataset, the plots of the PCA 2D projections, as well as the plots of the mean of the MVIB 2D stochastic encodings. For the MVIB stochastic encodings z∼p(z|x)=N(μ,σ2I), the depicted points represent the mean μ. The K dimension of the latent space has been set to 2 in order to allow a 2D visualisation of the encodings. For training MVIB, the JMVIB−T objective (Eq 8) has been optimised. For MVIB, five copies of the means plots are available, as they are obtained by training the model with five different independent training-test random splits. Both the PCA and the MVIB plots have been created starting from the default datasets collection. (ZIP) [file pcbi.1010050.s008.zip › s6-file/T2D/PCA_projections.png]

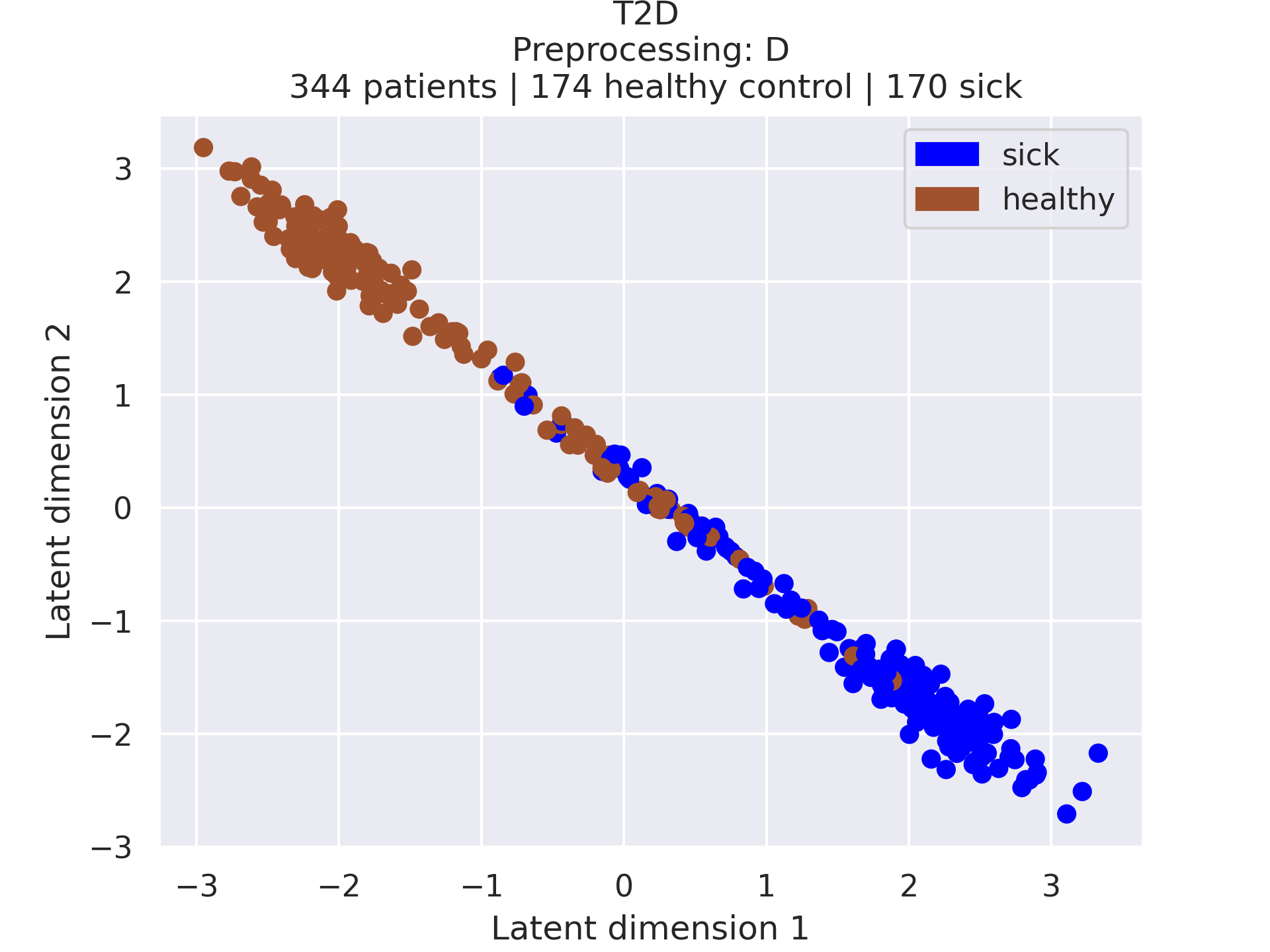

Supplement: S3 File — This file presents, for each dataset, the plots of the PCA 2D projections, as well as the plots of the mean of the MVIB 2D stochastic encodings. For the MVIB stochastic encodings z∼p(z|x)=N(μ,σ2I), the depicted points represent the mean μ. The K dimension of the latent space has been set to 2 in order to allow a 2D visualisation of the encodings. For training MVIB, the JMVIB−T objective (Eq 8) has been optimised. For MVIB, five copies of the means plots are available, as they are obtained by training the model with five different independent training-test random splits. Both the PCA and the MVIB plots have been created starting from the default datasets collection. (ZIP) [file pcbi.1010050.s008.zip › s6-file/T2D/2_embeddings.png]

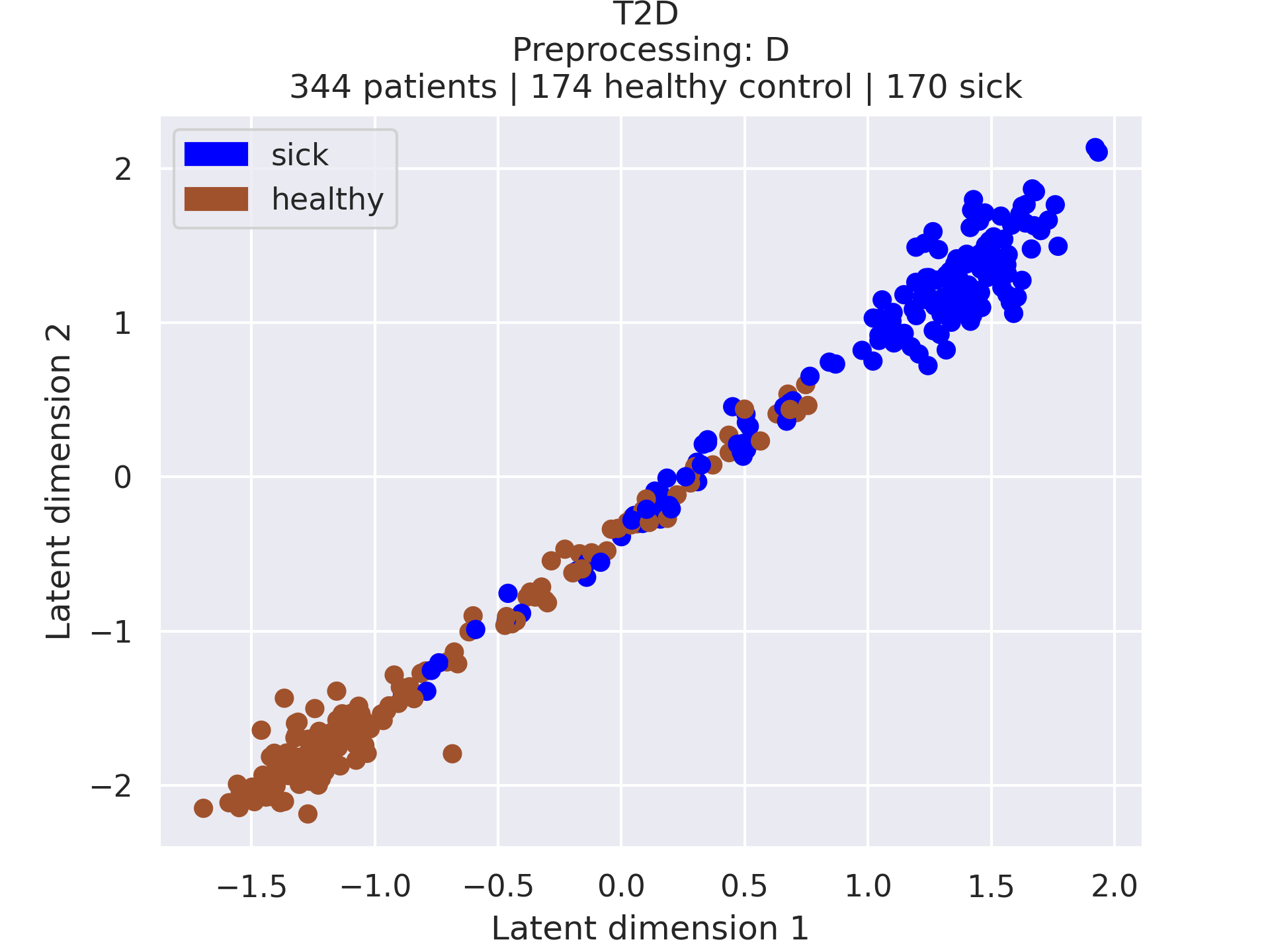

Supplement: S3 File — This file presents, for each dataset, the plots of the PCA 2D projections, as well as the plots of the mean of the MVIB 2D stochastic encodings. For the MVIB stochastic encodings z∼p(z|x)=N(μ,σ2I), the depicted points represent the mean μ. The K dimension of the latent space has been set to 2 in order to allow a 2D visualisation of the encodings. For training MVIB, the JMVIB−T objective (Eq 8) has been optimised. For MVIB, five copies of the means plots are available, as they are obtained by training the model with five different independent training-test random splits. Both the PCA and the MVIB plots have been created starting from the default datasets collection. (ZIP) [file pcbi.1010050.s008.zip › s6-file/T2D/0_embeddings.png]

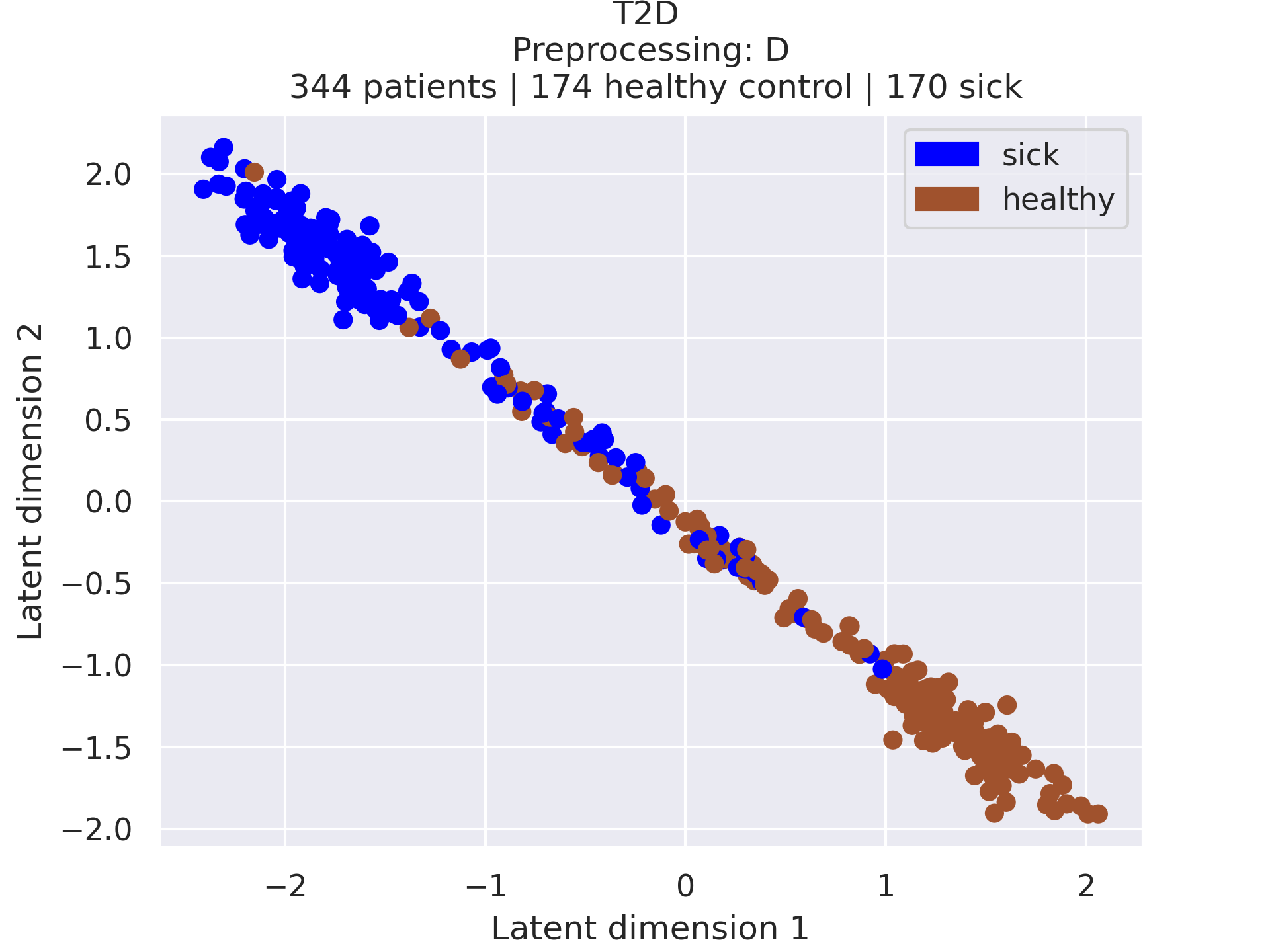

Supplement: S3 File — This file presents, for each dataset, the plots of the PCA 2D projections, as well as the plots of the mean of the MVIB 2D stochastic encodings. For the MVIB stochastic encodings z∼p(z|x)=N(μ,σ2I), the depicted points represent the mean μ. The K dimension of the latent space has been set to 2 in order to allow a 2D visualisation of the encodings. For training MVIB, the JMVIB−T objective (Eq 8) has been optimised. For MVIB, five copies of the means plots are available, as they are obtained by training the model with five different independent training-test random splits. Both the PCA and the MVIB plots have been created starting from the default datasets collection. (ZIP) [file pcbi.1010050.s008.zip › s6-file/T2D/1_embeddings.png]

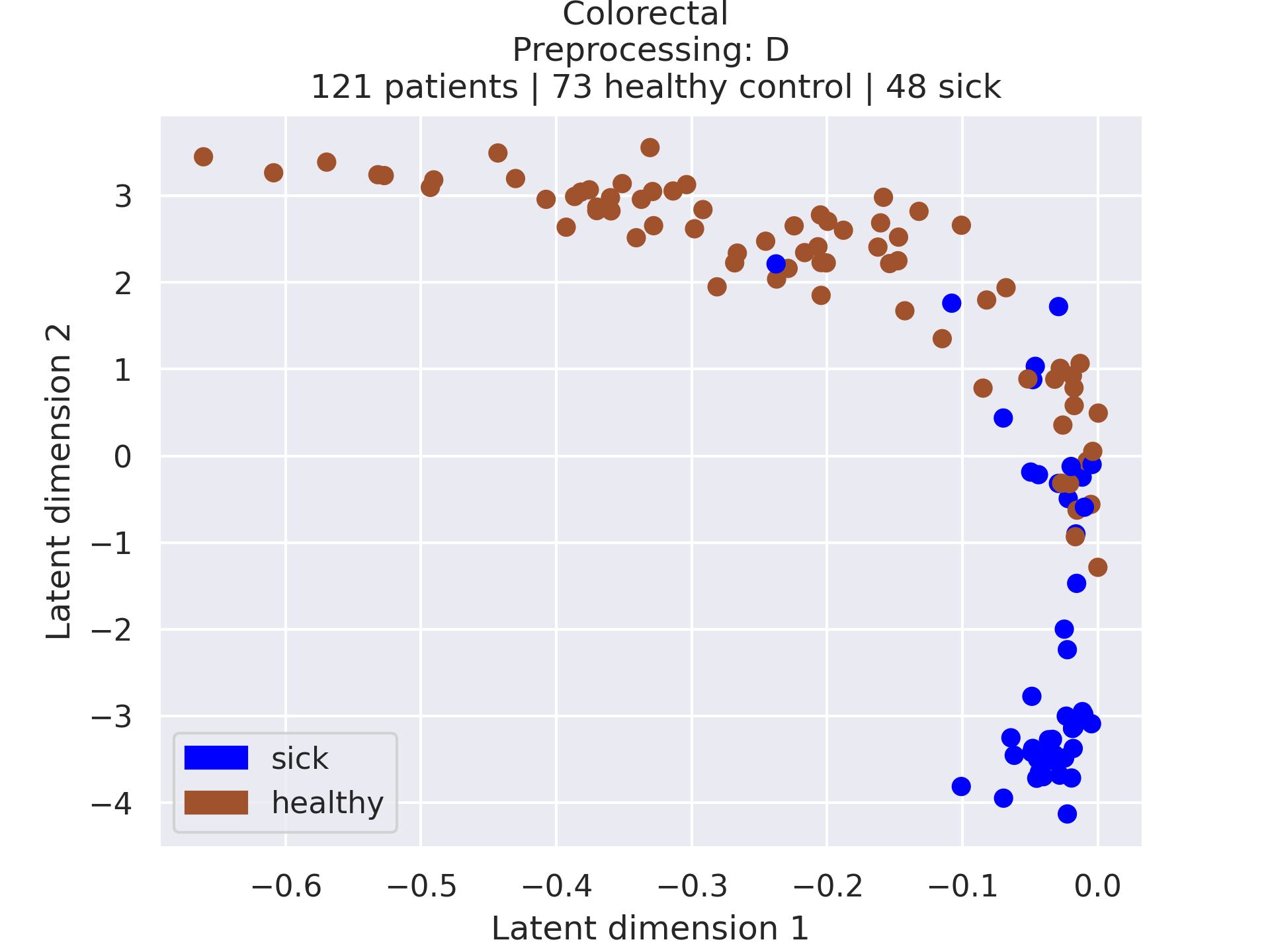

Supplement: S3 File — This file presents, for each dataset, the plots of the PCA 2D projections, as well as the plots of the mean of the MVIB 2D stochastic encodings. For the MVIB stochastic encodings z∼p(z|x)=N(μ,σ2I), the depicted points represent the mean μ. The K dimension of the latent space has been set to 2 in order to allow a 2D visualisation of the encodings. For training MVIB, the JMVIB−T objective (Eq 8) has been optimised. For MVIB, five copies of the means plots are available, as they are obtained by training the model with five different independent training-test random splits. Both the PCA and the MVIB plots have been created starting from the default datasets collection. (ZIP) [file pcbi.1010050.s008.zip › s6-file/Colorectal/3_embeddings.png]

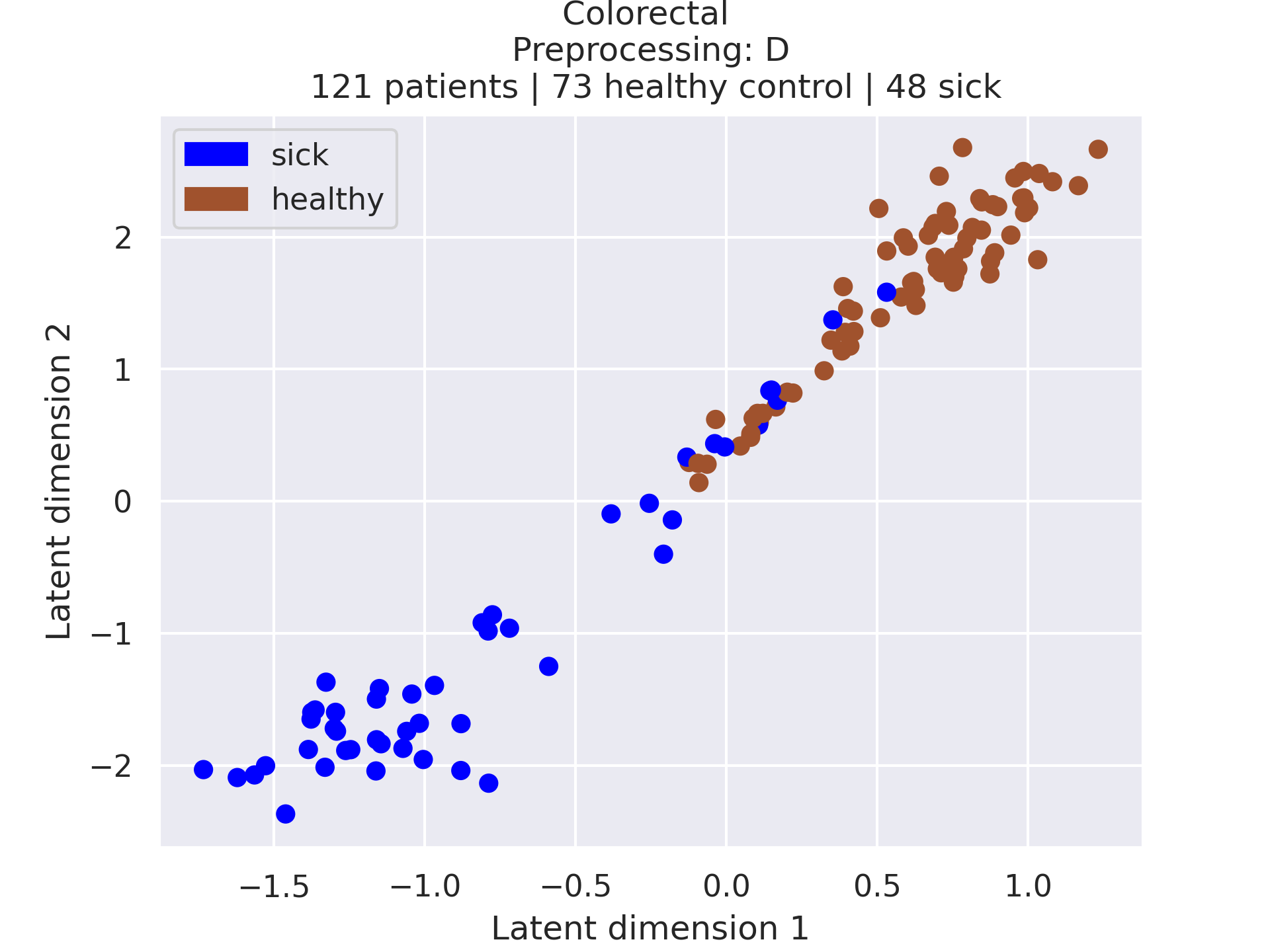

Supplement: S3 File — This file presents, for each dataset, the plots of the PCA 2D projections, as well as the plots of the mean of the MVIB 2D stochastic encodings. For the MVIB stochastic encodings z∼p(z|x)=N(μ,σ2I), the depicted points represent the mean μ. The K dimension of the latent space has been set to 2 in order to allow a 2D visualisation of the encodings. For training MVIB, the JMVIB−T objective (Eq 8) has been optimised. For MVIB, five copies of the means plots are available, as they are obtained by training the model with five different independent training-test random splits. Both the PCA and the MVIB plots have been created starting from the default datasets collection. (ZIP) [file pcbi.1010050.s008.zip › s6-file/Colorectal/4_embeddings.png]

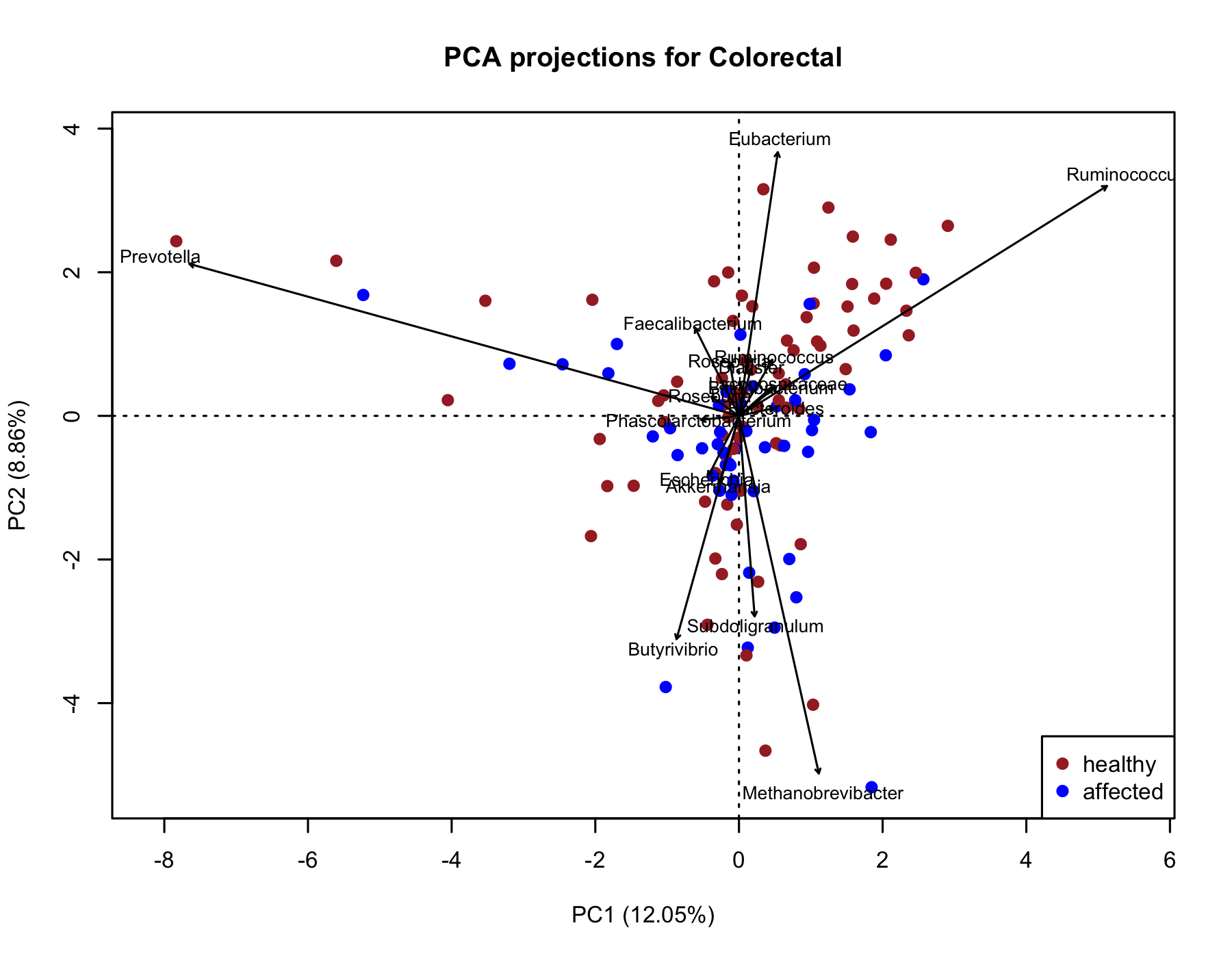

Supplement: S3 File — This file presents, for each dataset, the plots of the PCA 2D projections, as well as the plots of the mean of the MVIB 2D stochastic encodings. For the MVIB stochastic encodings z∼p(z|x)=N(μ,σ2I), the depicted points represent the mean μ. The K dimension of the latent space has been set to 2 in order to allow a 2D visualisation of the encodings. For training MVIB, the JMVIB−T objective (Eq 8) has been optimised. For MVIB, five copies of the means plots are available, as they are obtained by training the model with five different independent training-test random splits. Both the PCA and the MVIB plots have been created starting from the default datasets collection. (ZIP) [file pcbi.1010050.s008.zip › s6-file/Colorectal/PCA_projections.png]

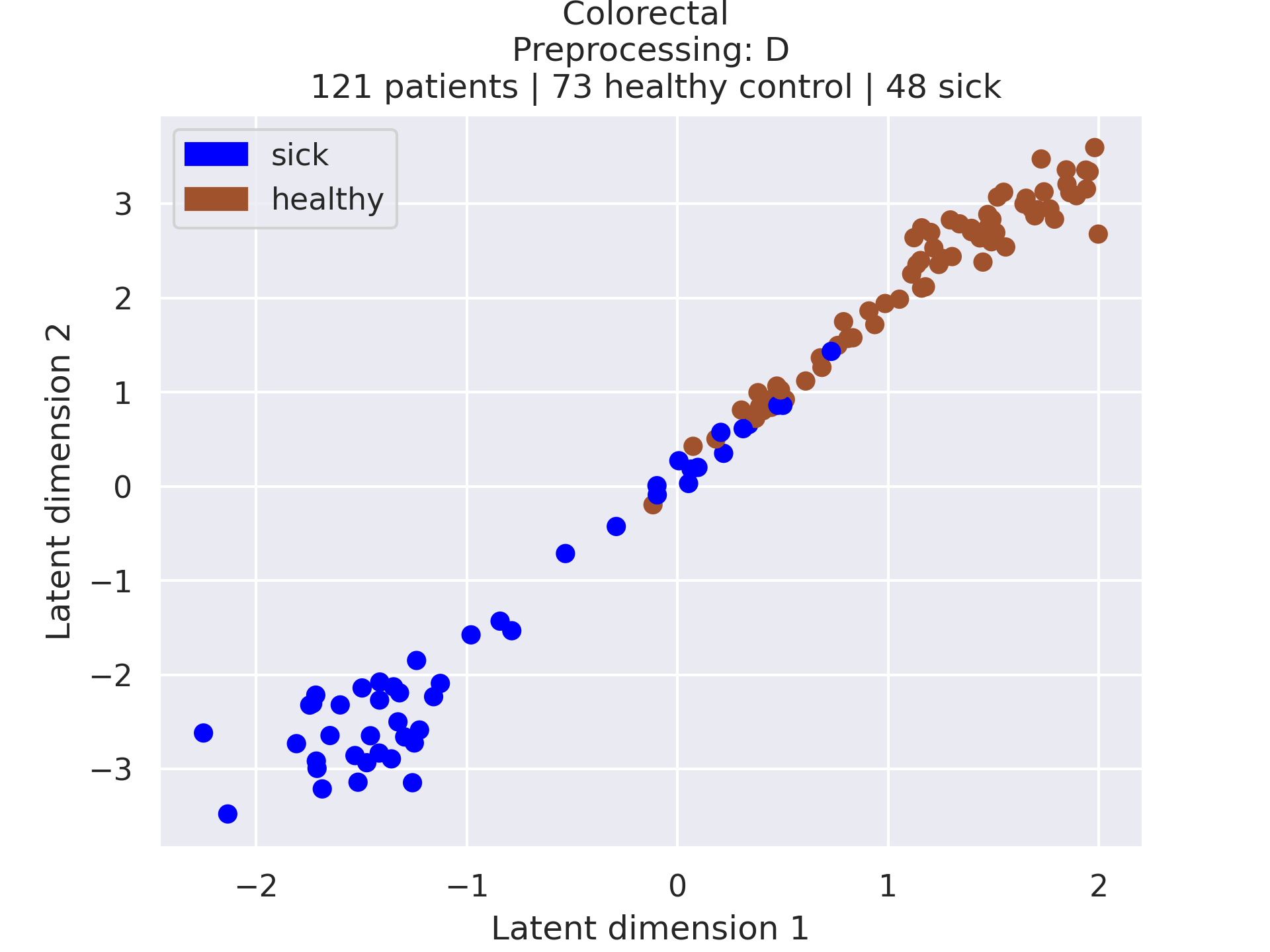

Supplement: S3 File — This file presents, for each dataset, the plots of the PCA 2D projections, as well as the plots of the mean of the MVIB 2D stochastic encodings. For the MVIB stochastic encodings z∼p(z|x)=N(μ,σ2I), the depicted points represent the mean μ. The K dimension of the latent space has been set to 2 in order to allow a 2D visualisation of the encodings. For training MVIB, the JMVIB−T objective (Eq 8) has been optimised. For MVIB, five copies of the means plots are available, as they are obtained by training the model with five different independent training-test random splits. Both the PCA and the MVIB plots have been created starting from the default datasets collection. (ZIP) [file pcbi.1010050.s008.zip › s6-file/Colorectal/2_embeddings.png]

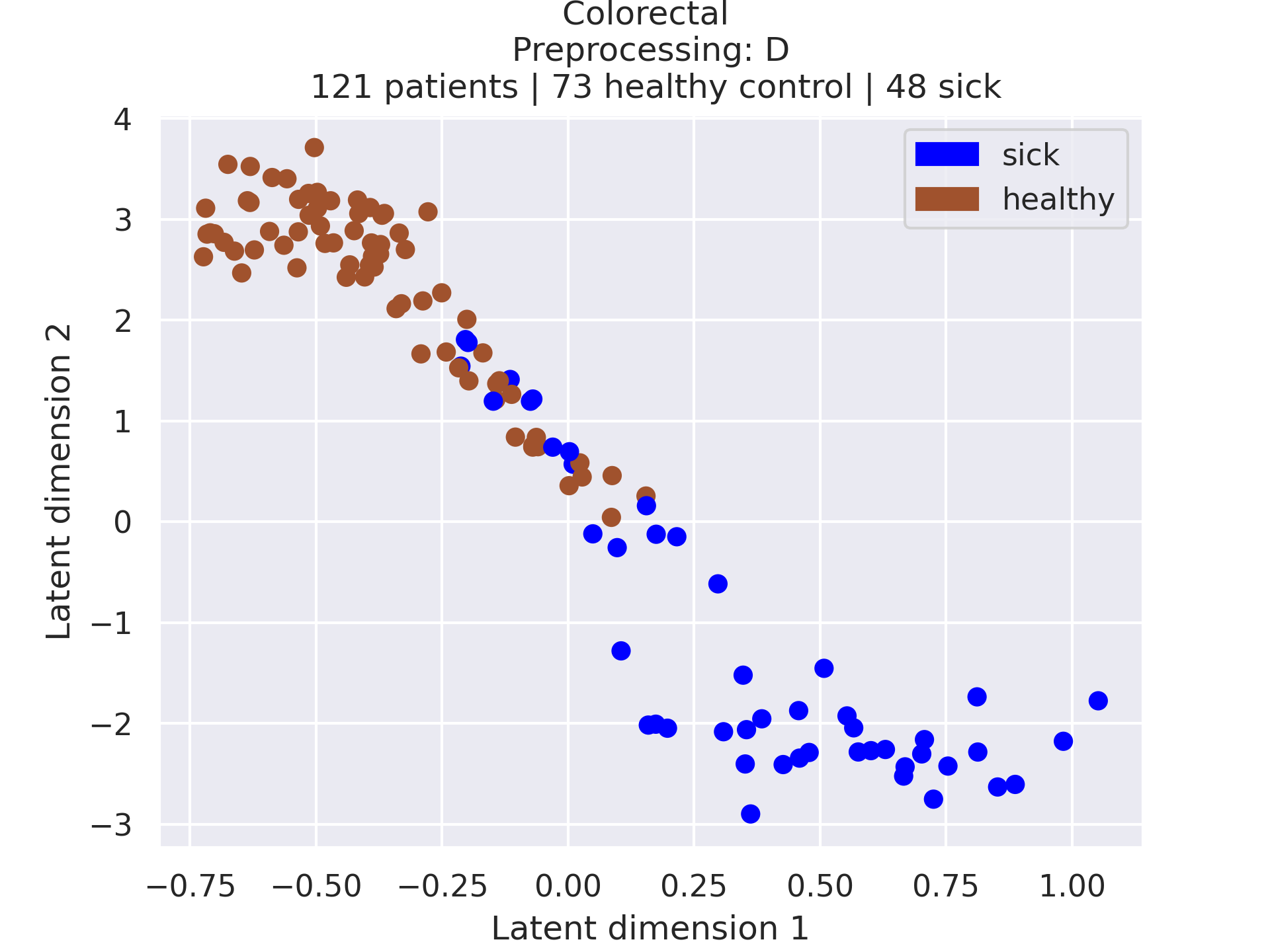

Supplement: S3 File — This file presents, for each dataset, the plots of the PCA 2D projections, as well as the plots of the mean of the MVIB 2D stochastic encodings. For the MVIB stochastic encodings z∼p(z|x)=N(μ,σ2I), the depicted points represent the mean μ. The K dimension of the latent space has been set to 2 in order to allow a 2D visualisation of the encodings. For training MVIB, the JMVIB−T objective (Eq 8) has been optimised. For MVIB, five copies of the means plots are available, as they are obtained by training the model with five different independent training-test random splits. Both the PCA and the MVIB plots have been created starting from the default datasets collection. (ZIP) [file pcbi.1010050.s008.zip › s6-file/Colorectal/0_embeddings.png]

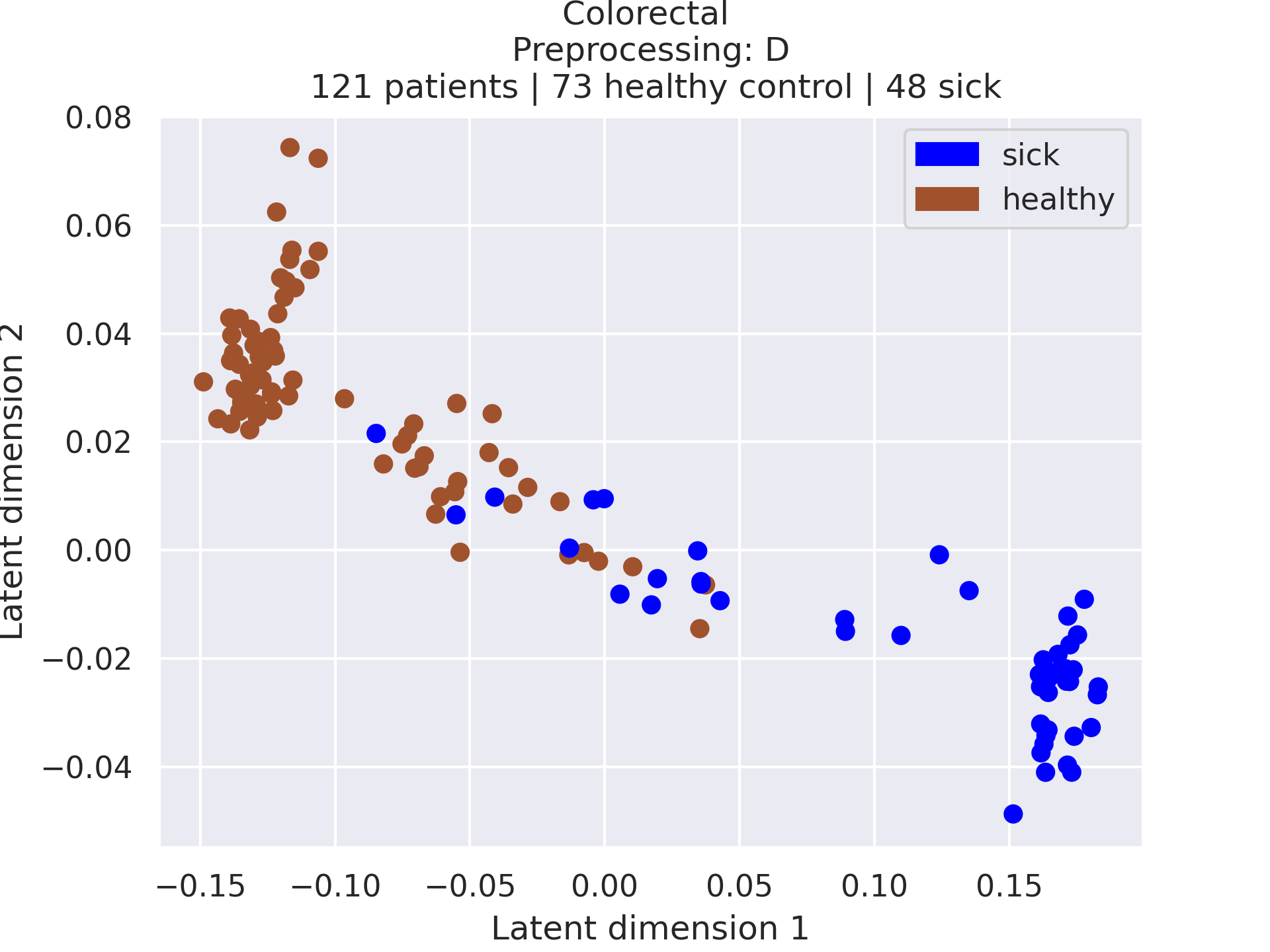

Supplement: S3 File — This file presents, for each dataset, the plots of the PCA 2D projections, as well as the plots of the mean of the MVIB 2D stochastic encodings. For the MVIB stochastic encodings z∼p(z|x)=N(μ,σ2I), the depicted points represent the mean μ. The K dimension of the latent space has been set to 2 in order to allow a 2D visualisation of the encodings. For training MVIB, the JMVIB−T objective (Eq 8) has been optimised. For MVIB, five copies of the means plots are available, as they are obtained by training the model with five different independent training-test random splits. Both the PCA and the MVIB plots have been created starting from the default datasets collection. (ZIP) [file pcbi.1010050.s008.zip › s6-file/Colorectal/1_embeddings.png]

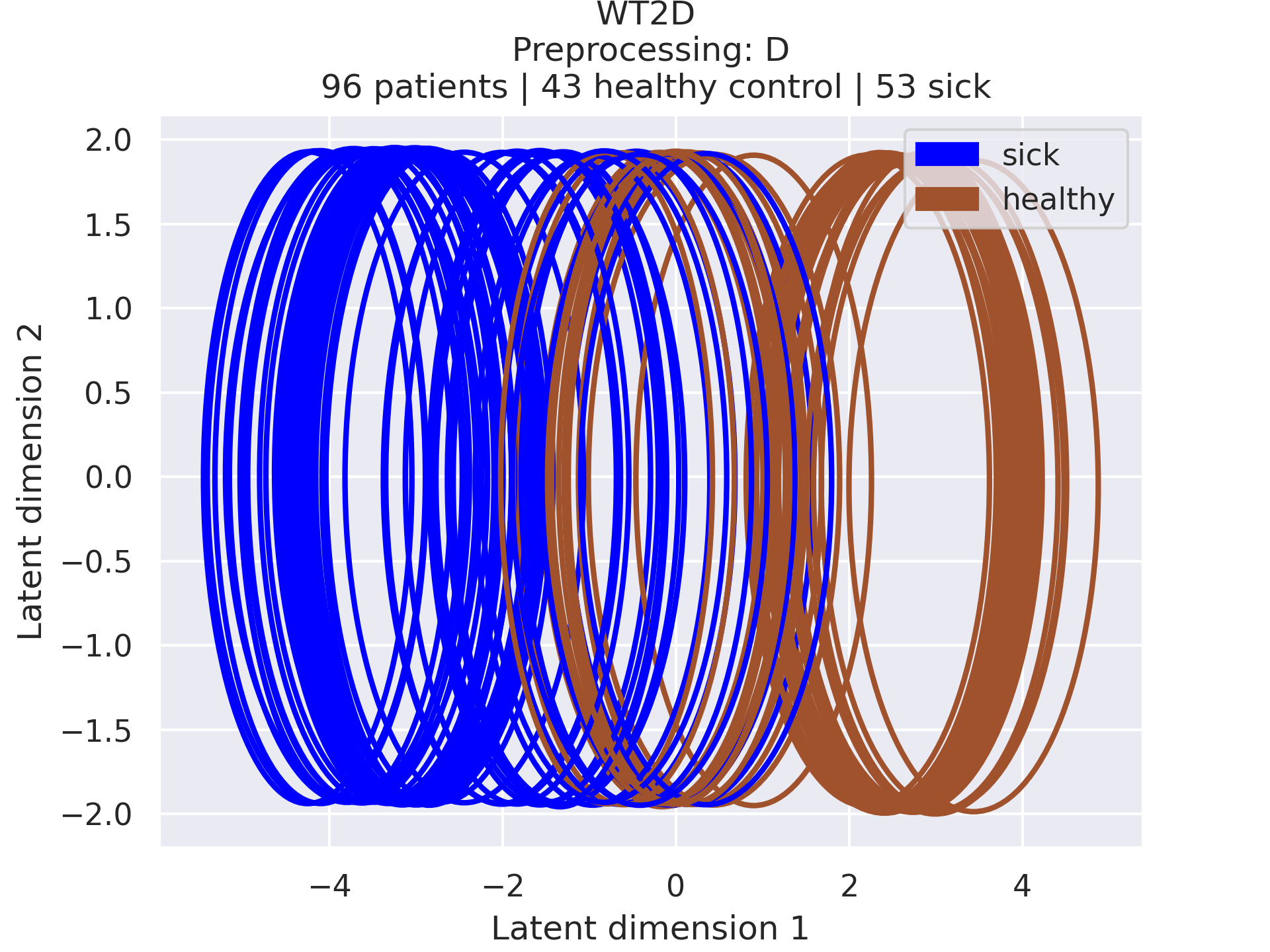

Supplement: S3 File — This file presents, for each dataset, the plots of the PCA 2D projections, as well as the plots of the mean of the MVIB 2D stochastic encodings. For the MVIB stochastic encodings z∼p(z|x)=N(μ,σ2I), the depicted points represent the mean μ. The K dimension of the latent space has been set to 2 in order to allow a 2D visualisation of the encodings. For training MVIB, the JMVIB−T objective (Eq 8) has been optimised. For MVIB, five copies of the means plots are available, as they are obtained by training the model with five different independent training-test random splits. Both the PCA and the MVIB plots have been created starting from the default datasets collection. (ZIP) [file pcbi.1010050.s008.zip › s6-file/WT2D/4_embeddings_95_confidence.png]

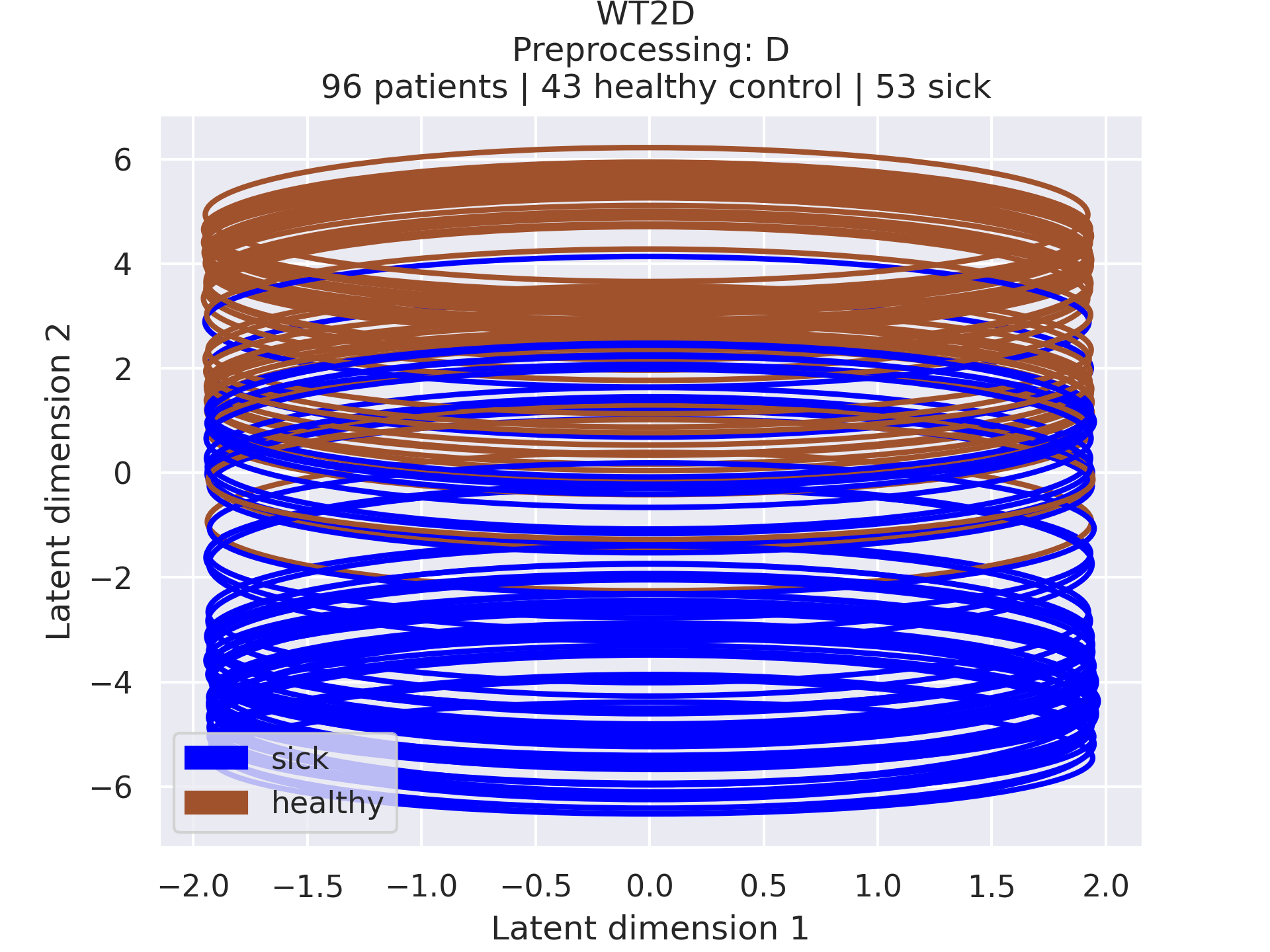

Supplement: S3 File — This file presents, for each dataset, the plots of the PCA 2D projections, as well as the plots of the mean of the MVIB 2D stochastic encodings. For the MVIB stochastic encodings z∼p(z|x)=N(μ,σ2I), the depicted points represent the mean μ. The K dimension of the latent space has been set to 2 in order to allow a 2D visualisation of the encodings. For training MVIB, the JMVIB−T objective (Eq 8) has been optimised. For MVIB, five copies of the means plots are available, as they are obtained by training the model with five different independent training-test random splits. Both the PCA and the MVIB plots have been created starting from the default datasets collection. (ZIP) [file pcbi.1010050.s008.zip › s6-file/WT2D/1_embeddings_95_confidence.png]

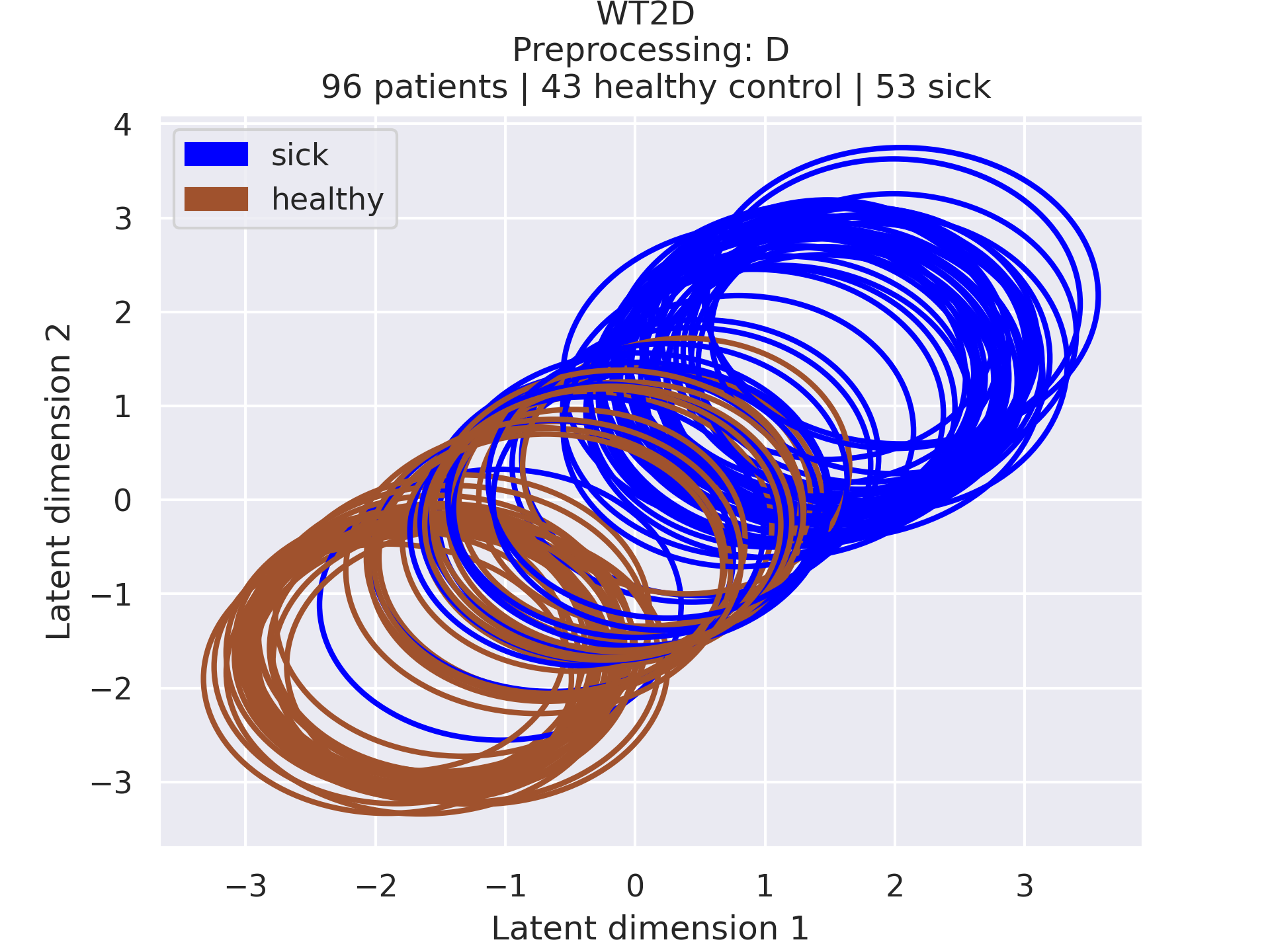

Supplement: S3 File — This file presents, for each dataset, the plots of the PCA 2D projections, as well as the plots of the mean of the MVIB 2D stochastic encodings. For the MVIB stochastic encodings z∼p(z|x)=N(μ,σ2I), the depicted points represent the mean μ. The K dimension of the latent space has been set to 2 in order to allow a 2D visualisation of the encodings. For training MVIB, the JMVIB−T objective (Eq 8) has been optimised. For MVIB, five copies of the means plots are available, as they are obtained by training the model with five different independent training-test random splits. Both the PCA and the MVIB plots have been created starting from the default datasets collection. (ZIP) [file pcbi.1010050.s008.zip › s6-file/WT2D/2_embeddings_95_confidence.png]

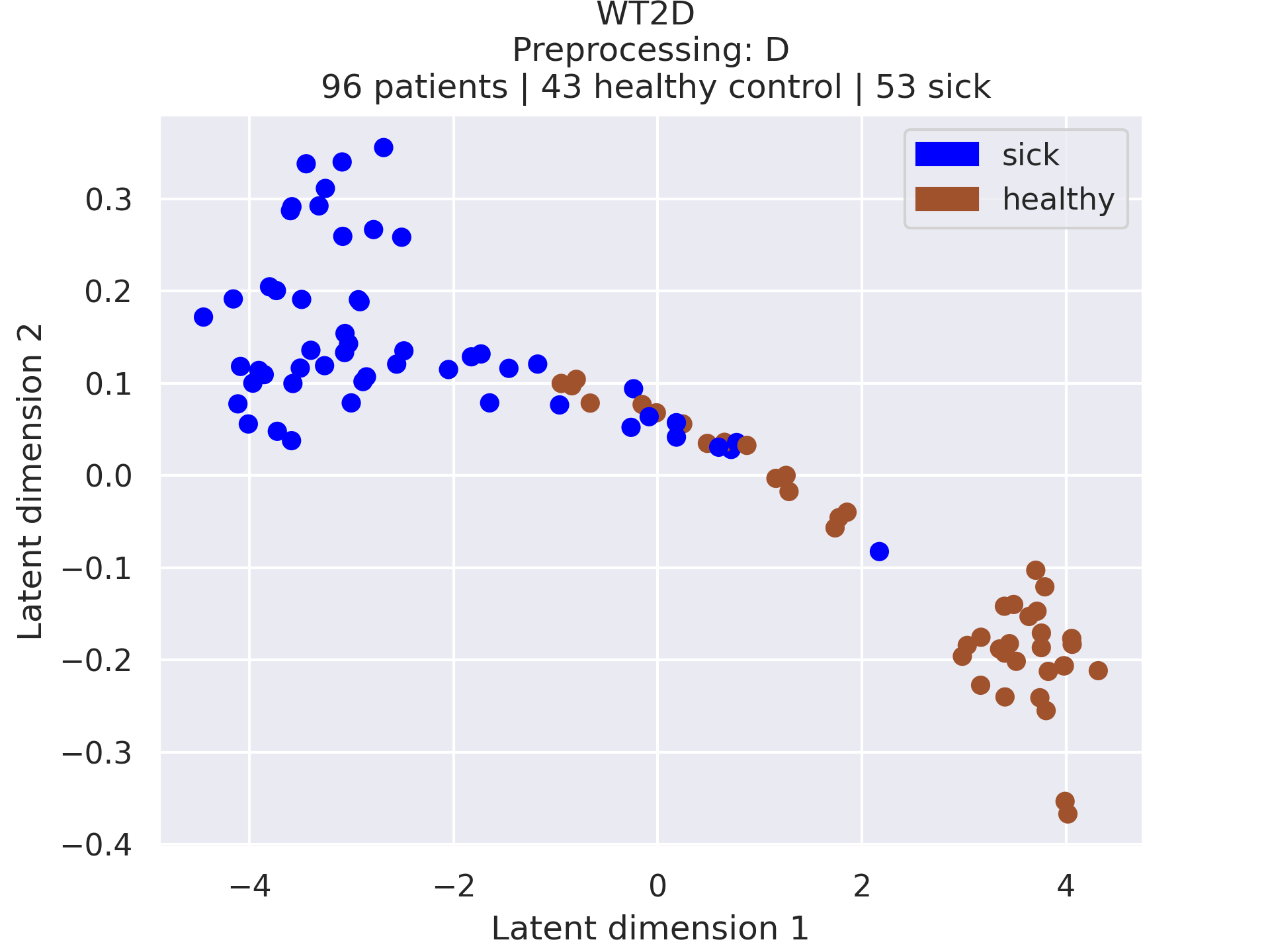

Supplement: S3 File — This file presents, for each dataset, the plots of the PCA 2D projections, as well as the plots of the mean of the MVIB 2D stochastic encodings. For the MVIB stochastic encodings z∼p(z|x)=N(μ,σ2I), the depicted points represent the mean μ. The K dimension of the latent space has been set to 2 in order to allow a 2D visualisation of the encodings. For training MVIB, the JMVIB−T objective (Eq 8) has been optimised. For MVIB, five copies of the means plots are available, as they are obtained by training the model with five different independent training-test random splits. Both the PCA and the MVIB plots have been created starting from the default datasets collection. (ZIP) [file pcbi.1010050.s008.zip › s6-file/WT2D/3_embeddings.png]

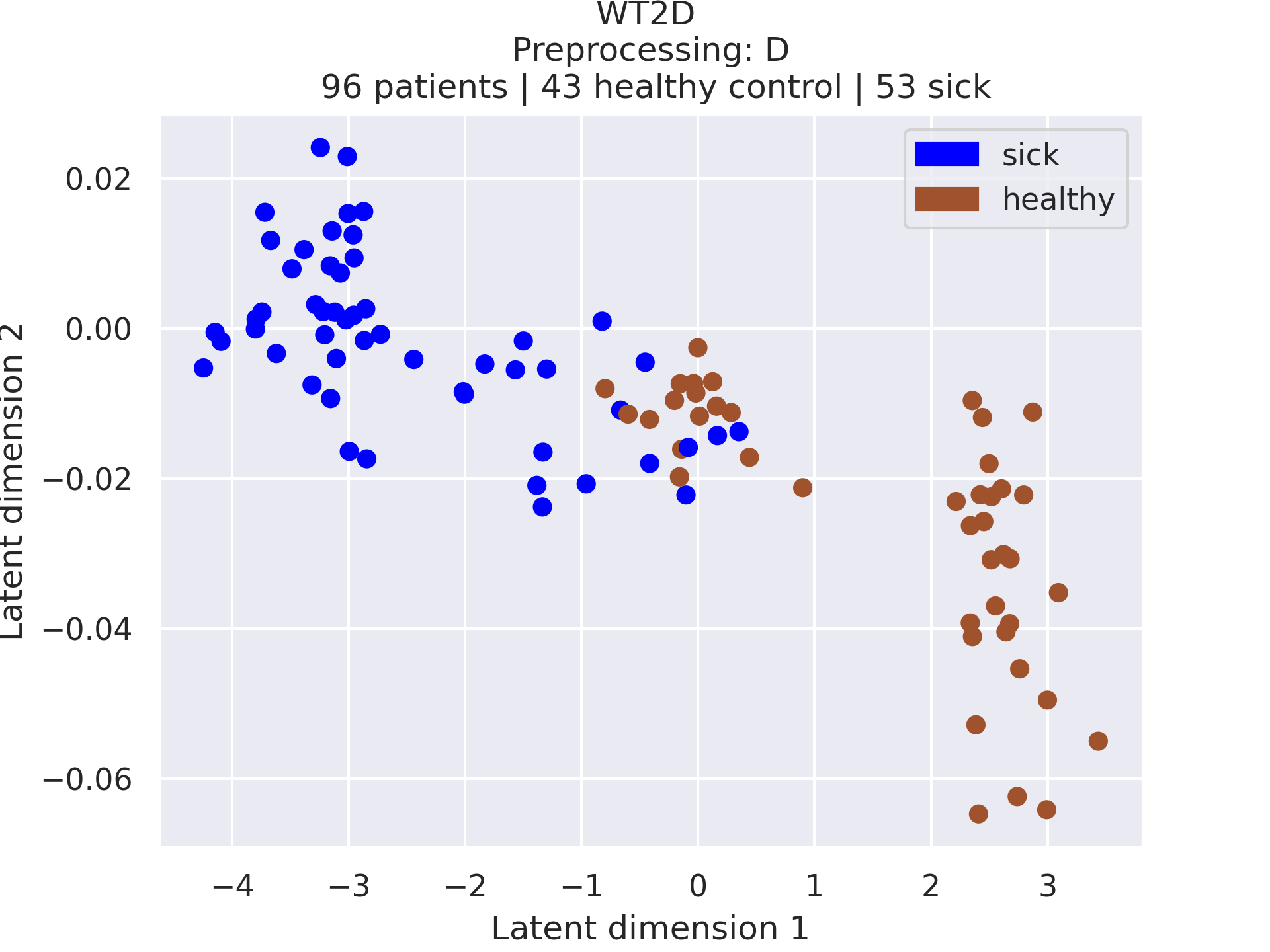

Supplement: S3 File — This file presents, for each dataset, the plots of the PCA 2D projections, as well as the plots of the mean of the MVIB 2D stochastic encodings. For the MVIB stochastic encodings z∼p(z|x)=N(μ,σ2I), the depicted points represent the mean μ. The K dimension of the latent space has been set to 2 in order to allow a 2D visualisation of the encodings. For training MVIB, the JMVIB−T objective (Eq 8) has been optimised. For MVIB, five copies of the means plots are available, as they are obtained by training the model with five different independent training-test random splits. Both the PCA and the MVIB plots have been created starting from the default datasets collection. (ZIP) [file pcbi.1010050.s008.zip › s6-file/WT2D/4_embeddings.png]

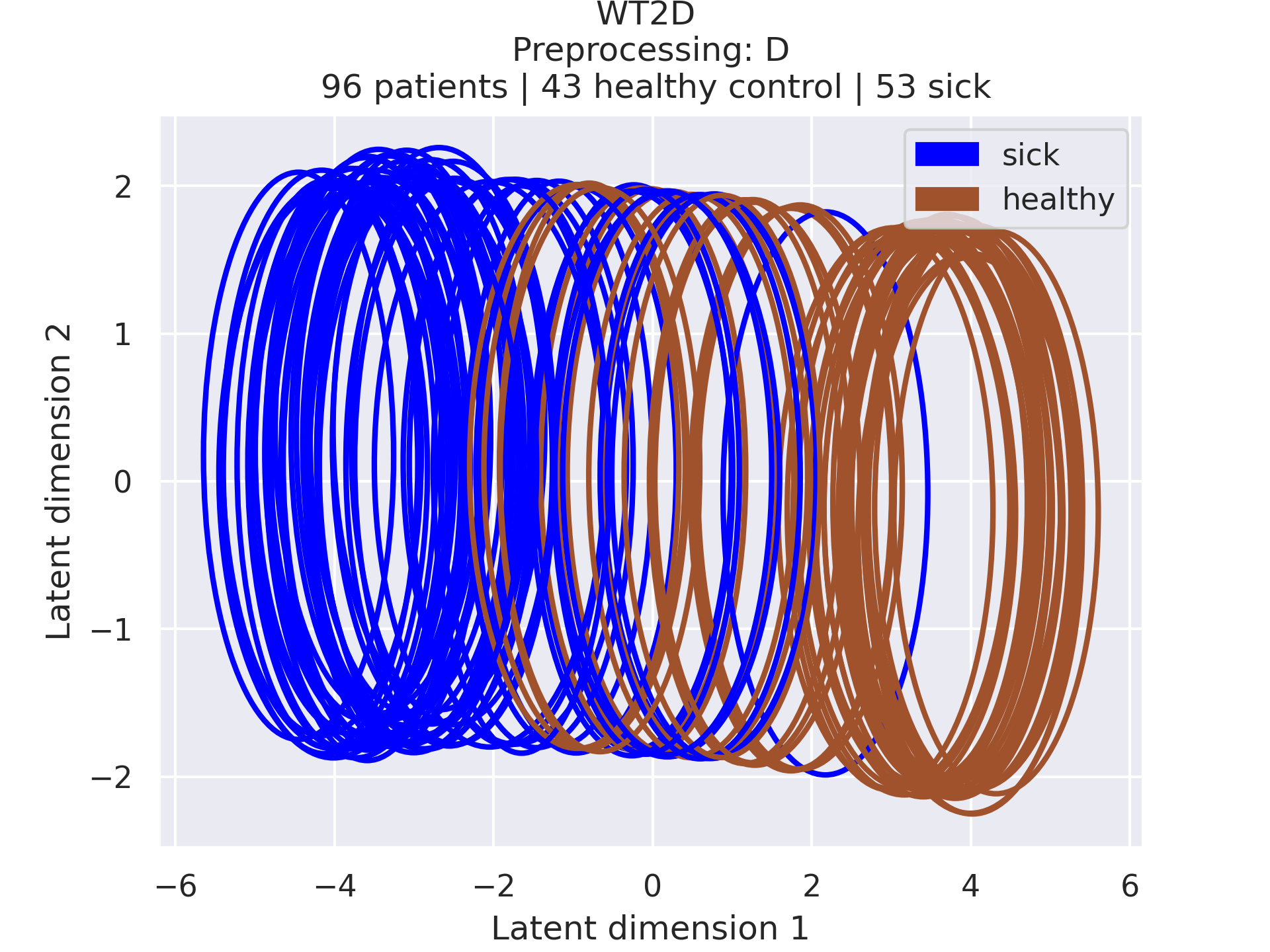

Supplement: S3 File — This file presents, for each dataset, the plots of the PCA 2D projections, as well as the plots of the mean of the MVIB 2D stochastic encodings. For the MVIB stochastic encodings z∼p(z|x)=N(μ,σ2I), the depicted points represent the mean μ. The K dimension of the latent space has been set to 2 in order to allow a 2D visualisation of the encodings. For training MVIB, the JMVIB−T objective (Eq 8) has been optimised. For MVIB, five copies of the means plots are available, as they are obtained by training the model with five different independent training-test random splits. Both the PCA and the MVIB plots have been created starting from the default datasets collection. (ZIP) [file pcbi.1010050.s008.zip › s6-file/WT2D/3_embeddings_95_confidence.png]

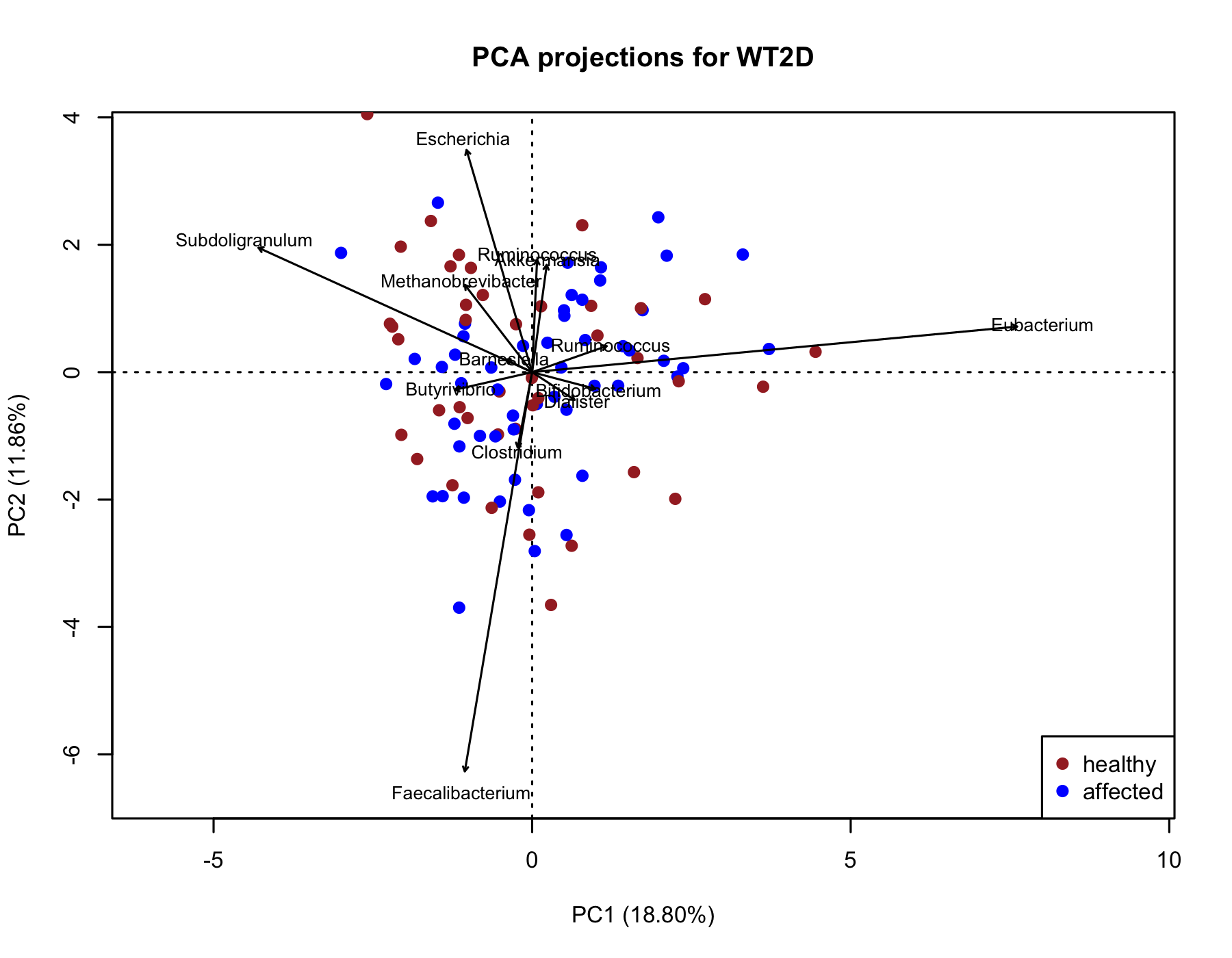

Supplement: S3 File — This file presents, for each dataset, the plots of the PCA 2D projections, as well as the plots of the mean of the MVIB 2D stochastic encodings. For the MVIB stochastic encodings z∼p(z|x)=N(μ,σ2I), the depicted points represent the mean μ. The K dimension of the latent space has been set to 2 in order to allow a 2D visualisation of the encodings. For training MVIB, the JMVIB−T objective (Eq 8) has been optimised. For MVIB, five copies of the means plots are available, as they are obtained by training the model with five different independent training-test random splits. Both the PCA and the MVIB plots have been created starting from the default datasets collection. (ZIP) [file pcbi.1010050.s008.zip › s6-file/WT2D/PCA_projections.png]

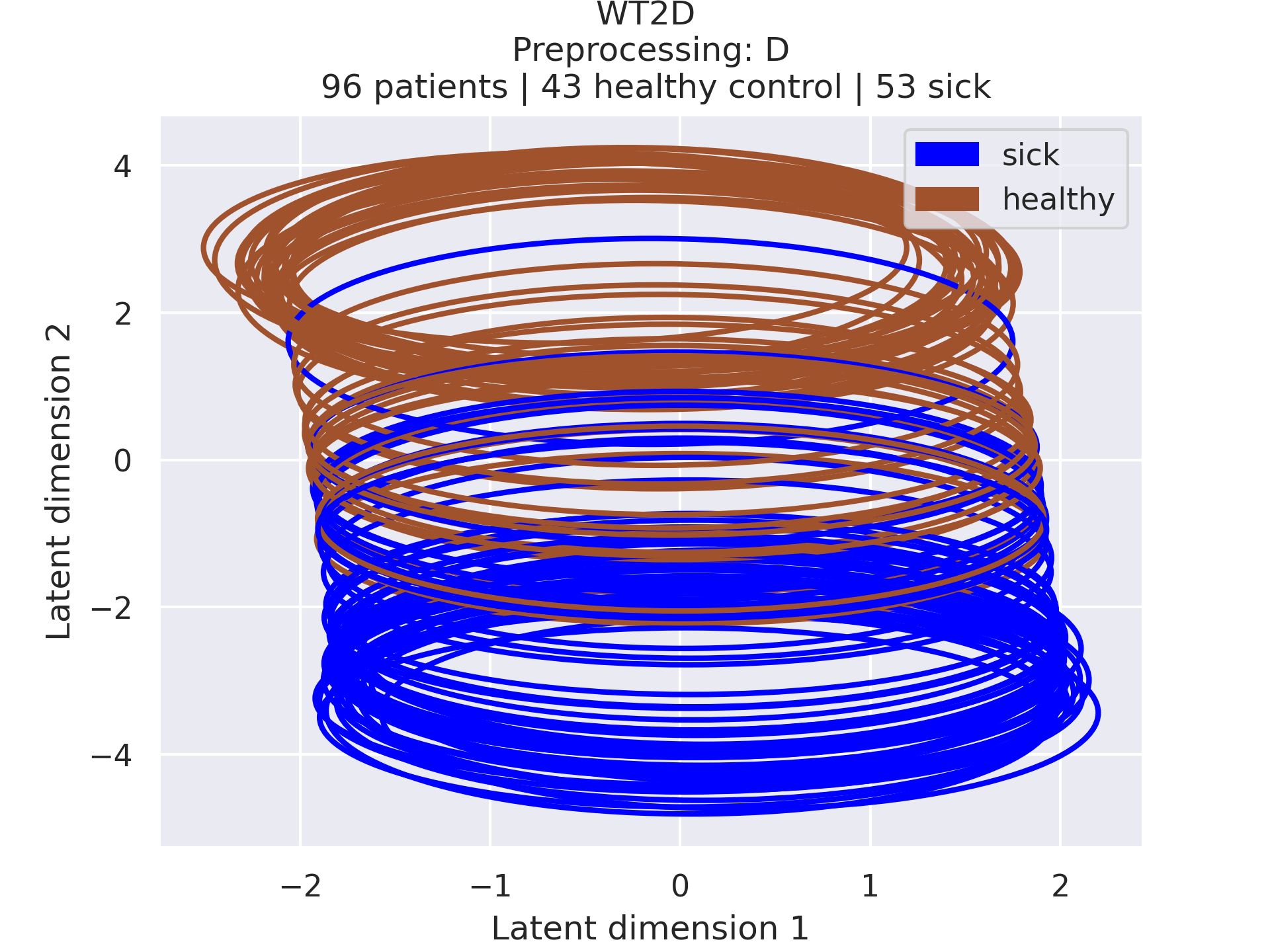

Supplement: S3 File — This file presents, for each dataset, the plots of the PCA 2D projections, as well as the plots of the mean of the MVIB 2D stochastic encodings. For the MVIB stochastic encodings z∼p(z|x)=N(μ,σ2I), the depicted points represent the mean μ. The K dimension of the latent space has been set to 2 in order to allow a 2D visualisation of the encodings. For training MVIB, the JMVIB−T objective (Eq 8) has been optimised. For MVIB, five copies of the means plots are available, as they are obtained by training the model with five different independent training-test random splits. Both the PCA and the MVIB plots have been created starting from the default datasets collection. (ZIP) [file pcbi.1010050.s008.zip › s6-file/WT2D/0_embeddings_95_confidence.png]

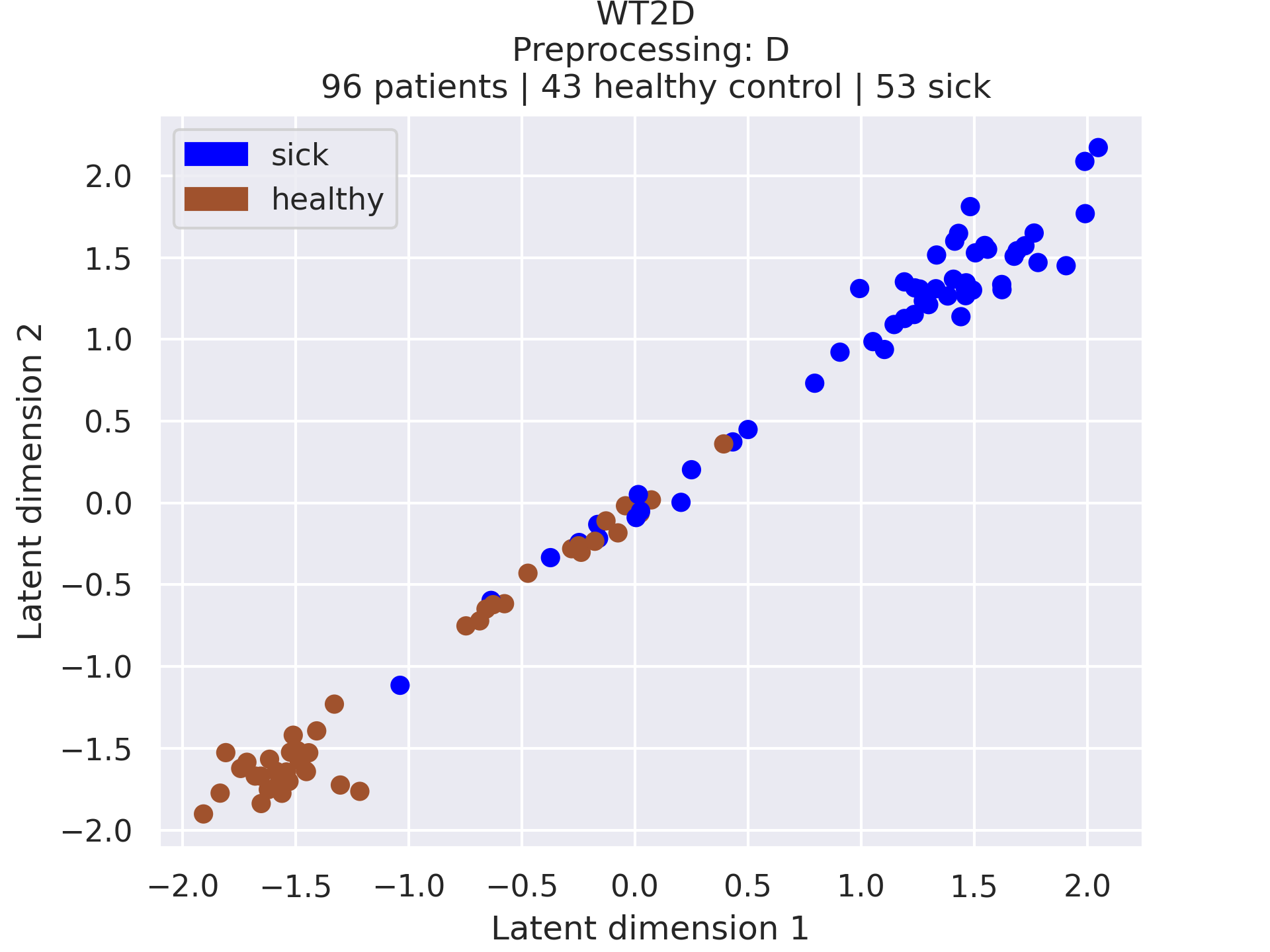

Supplement: S3 File — This file presents, for each dataset, the plots of the PCA 2D projections, as well as the plots of the mean of the MVIB 2D stochastic encodings. For the MVIB stochastic encodings z∼p(z|x)=N(μ,σ2I), the depicted points represent the mean μ. The K dimension of the latent space has been set to 2 in order to allow a 2D visualisation of the encodings. For training MVIB, the JMVIB−T objective (Eq 8) has been optimised. For MVIB, five copies of the means plots are available, as they are obtained by training the model with five different independent training-test random splits. Both the PCA and the MVIB plots have been created starting from the default datasets collection. (ZIP) [file pcbi.1010050.s008.zip › s6-file/WT2D/2_embeddings.png]

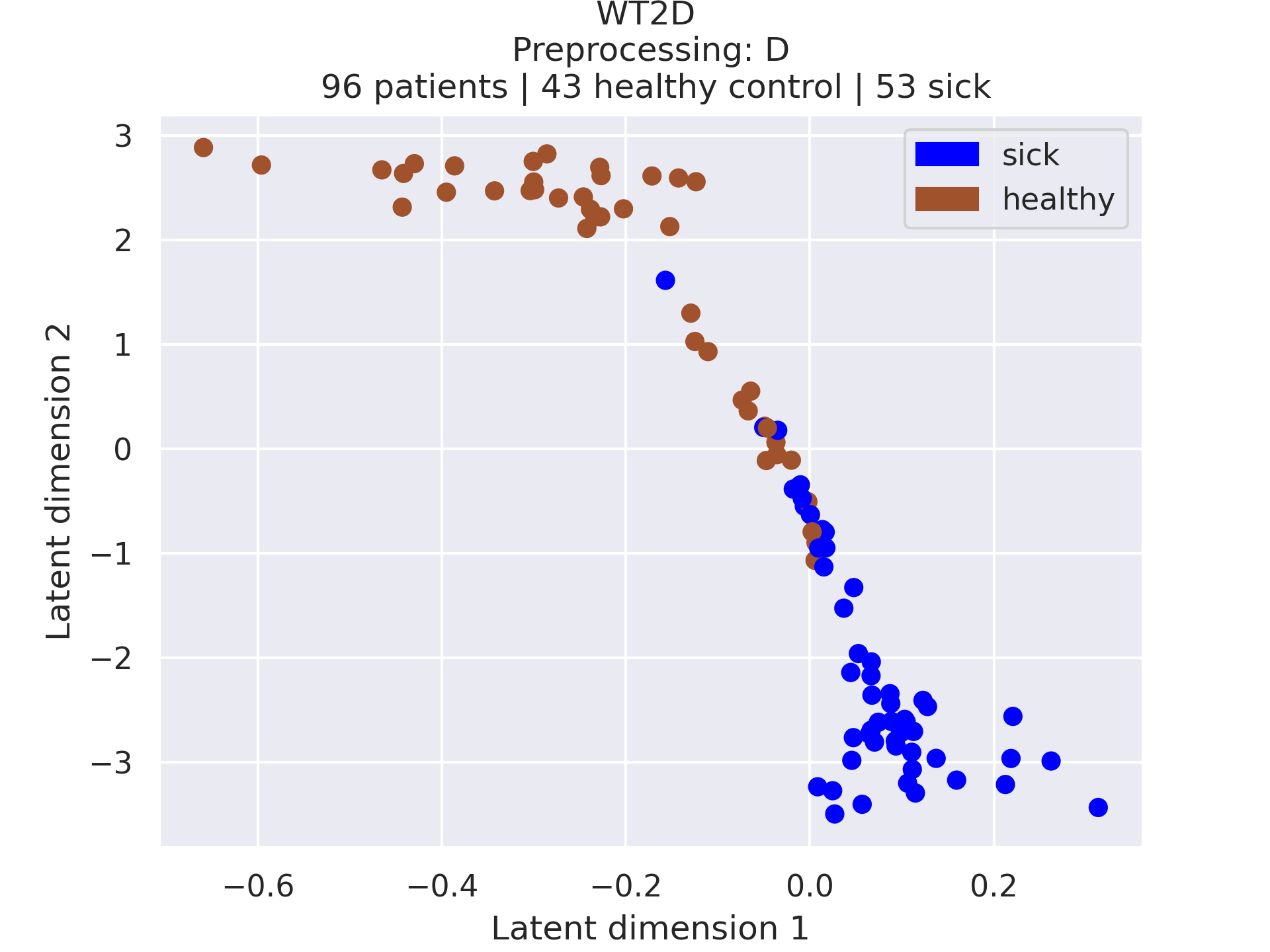

Supplement: S3 File — This file presents, for each dataset, the plots of the PCA 2D projections, as well as the plots of the mean of the MVIB 2D stochastic encodings. For the MVIB stochastic encodings z∼p(z|x)=N(μ,σ2I), the depicted points represent the mean μ. The K dimension of the latent space has been set to 2 in order to allow a 2D visualisation of the encodings. For training MVIB, the JMVIB−T objective (Eq 8) has been optimised. For MVIB, five copies of the means plots are available, as they are obtained by training the model with five different independent training-test random splits. Both the PCA and the MVIB plots have been created starting from the default datasets collection. (ZIP) [file pcbi.1010050.s008.zip › s6-file/WT2D/0_embeddings.png]

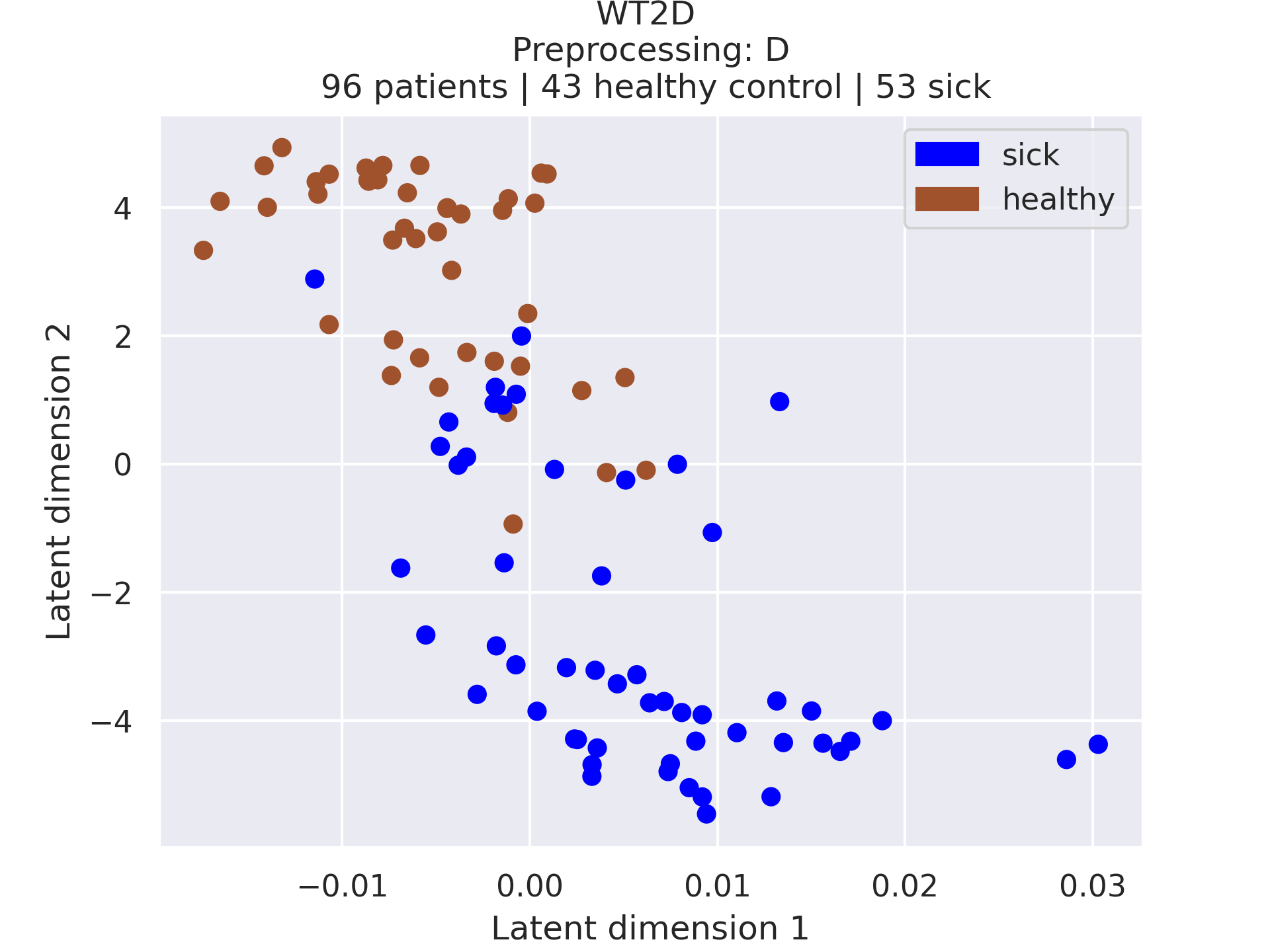

Supplement: S3 File — This file presents, for each dataset, the plots of the PCA 2D projections, as well as the plots of the mean of the MVIB 2D stochastic encodings. For the MVIB stochastic encodings z∼p(z|x)=N(μ,σ2I), the depicted points represent the mean μ. The K dimension of the latent space has been set to 2 in order to allow a 2D visualisation of the encodings. For training MVIB, the JMVIB−T objective (Eq 8) has been optimised. For MVIB, five copies of the means plots are available, as they are obtained by training the model with five different independent training-test random splits. Both the PCA and the MVIB plots have been created starting from the default datasets collection. (ZIP) [file pcbi.1010050.s008.zip › s6-file/WT2D/1_embeddings.png]

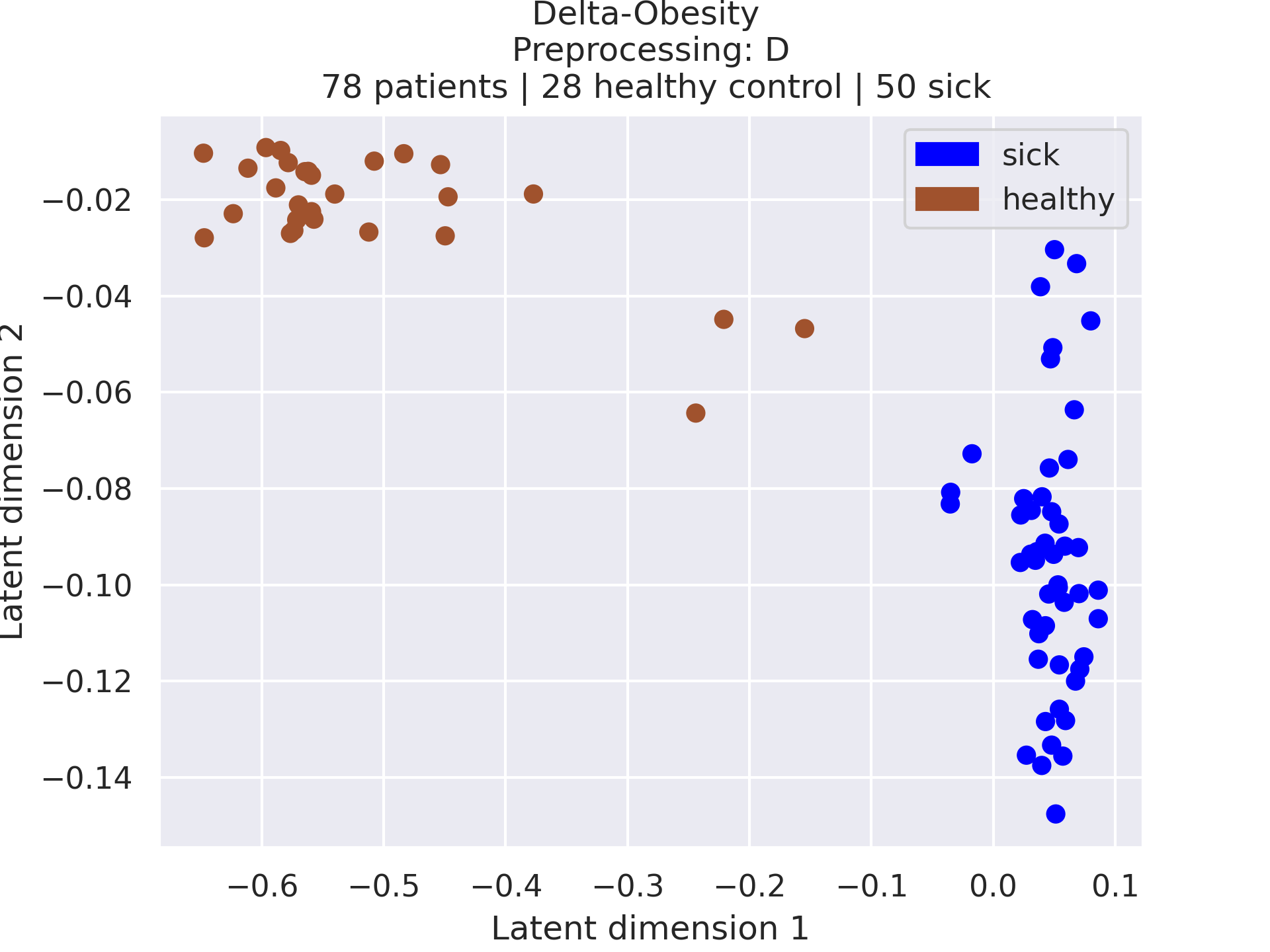

Supplement: S3 File — This file presents, for each dataset, the plots of the PCA 2D projections, as well as the plots of the mean of the MVIB 2D stochastic encodings. For the MVIB stochastic encodings z∼p(z|x)=N(μ,σ2I), the depicted points represent the mean μ. The K dimension of the latent space has been set to 2 in order to allow a 2D visualisation of the encodings. For training MVIB, the JMVIB−T objective (Eq 8) has been optimised. For MVIB, five copies of the means plots are available, as they are obtained by training the model with five different independent training-test random splits. Both the PCA and the MVIB plots have been created starting from the default datasets collection. (ZIP) [file pcbi.1010050.s008.zip › s6-file/Delta-Obesity/3_embeddings.png]

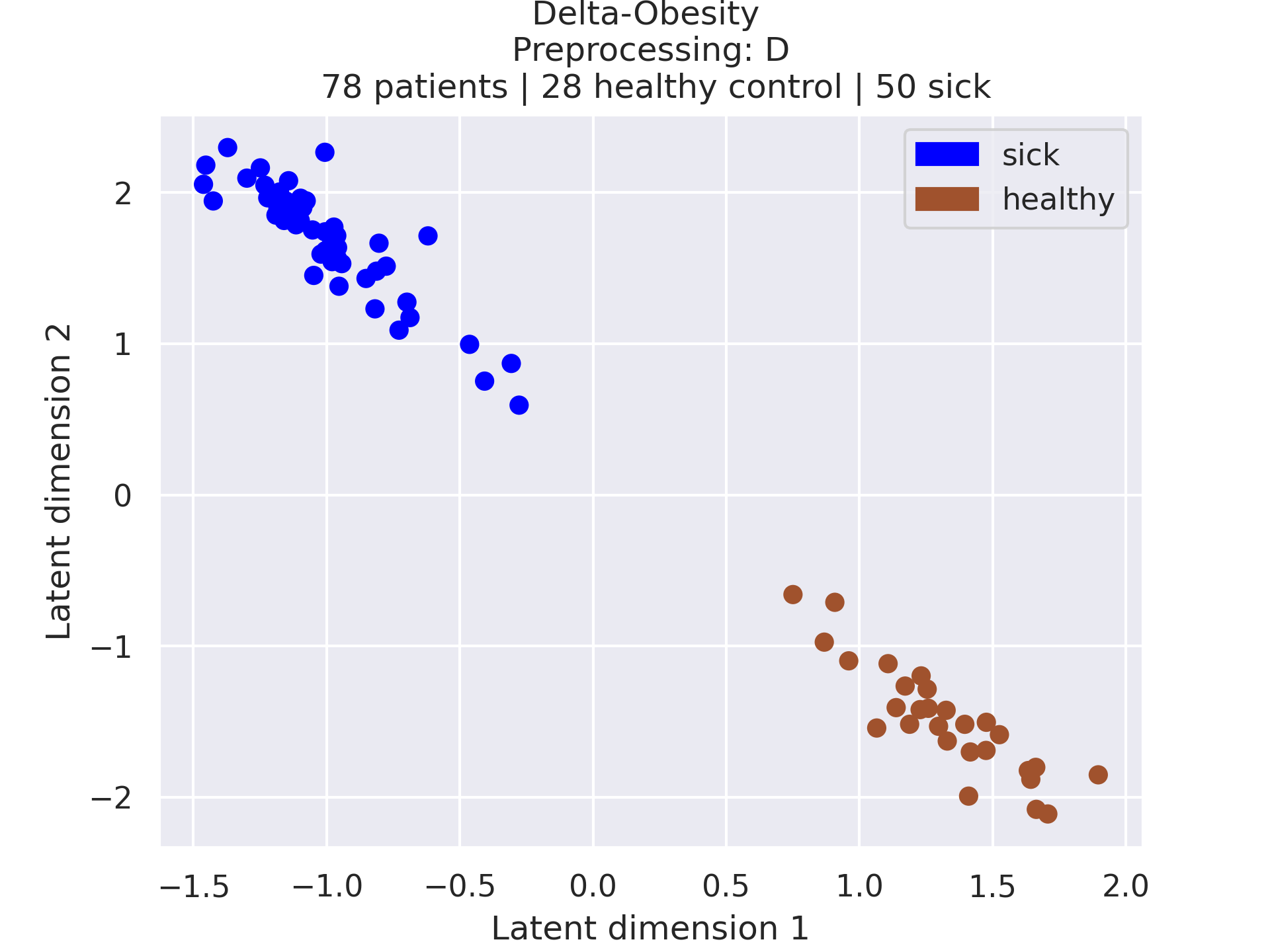

Supplement: S3 File — This file presents, for each dataset, the plots of the PCA 2D projections, as well as the plots of the mean of the MVIB 2D stochastic encodings. For the MVIB stochastic encodings z∼p(z|x)=N(μ,σ2I), the depicted points represent the mean μ. The K dimension of the latent space has been set to 2 in order to allow a 2D visualisation of the encodings. For training MVIB, the JMVIB−T objective (Eq 8) has been optimised. For MVIB, five copies of the means plots are available, as they are obtained by training the model with five different independent training-test random splits. Both the PCA and the MVIB plots have been created starting from the default datasets collection. (ZIP) [file pcbi.1010050.s008.zip › s6-file/Delta-Obesity/4_embeddings.png]

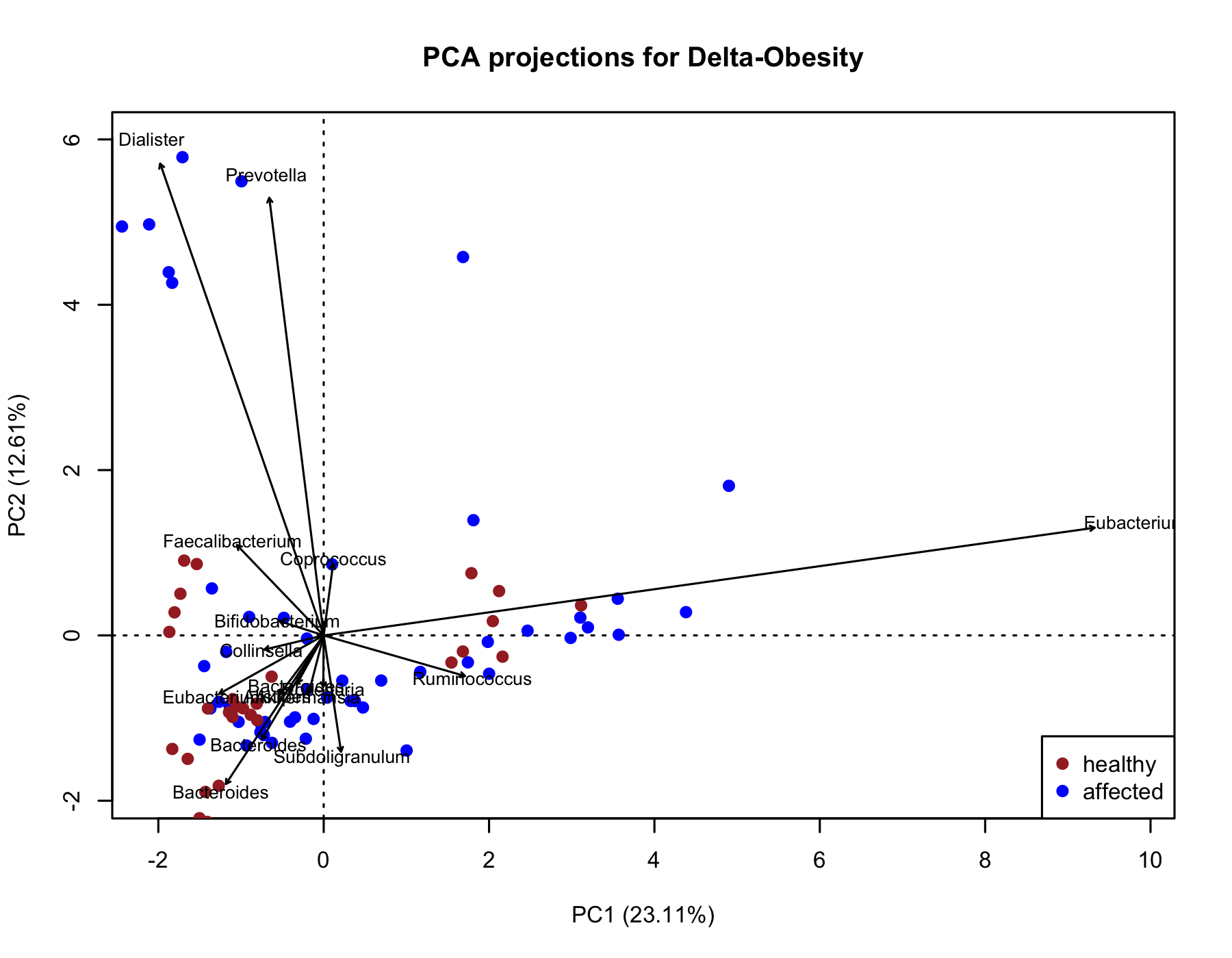

Supplement: S3 File — This file presents, for each dataset, the plots of the PCA 2D projections, as well as the plots of the mean of the MVIB 2D stochastic encodings. For the MVIB stochastic encodings z∼p(z|x)=N(μ,σ2I), the depicted points represent the mean μ. The K dimension of the latent space has been set to 2 in order to allow a 2D visualisation of the encodings. For training MVIB, the JMVIB−T objective (Eq 8) has been optimised. For MVIB, five copies of the means plots are available, as they are obtained by training the model with five different independent training-test random splits. Both the PCA and the MVIB plots have been created starting from the default datasets collection. (ZIP) [file pcbi.1010050.s008.zip › s6-file/Delta-Obesity/PCA_projections.png]

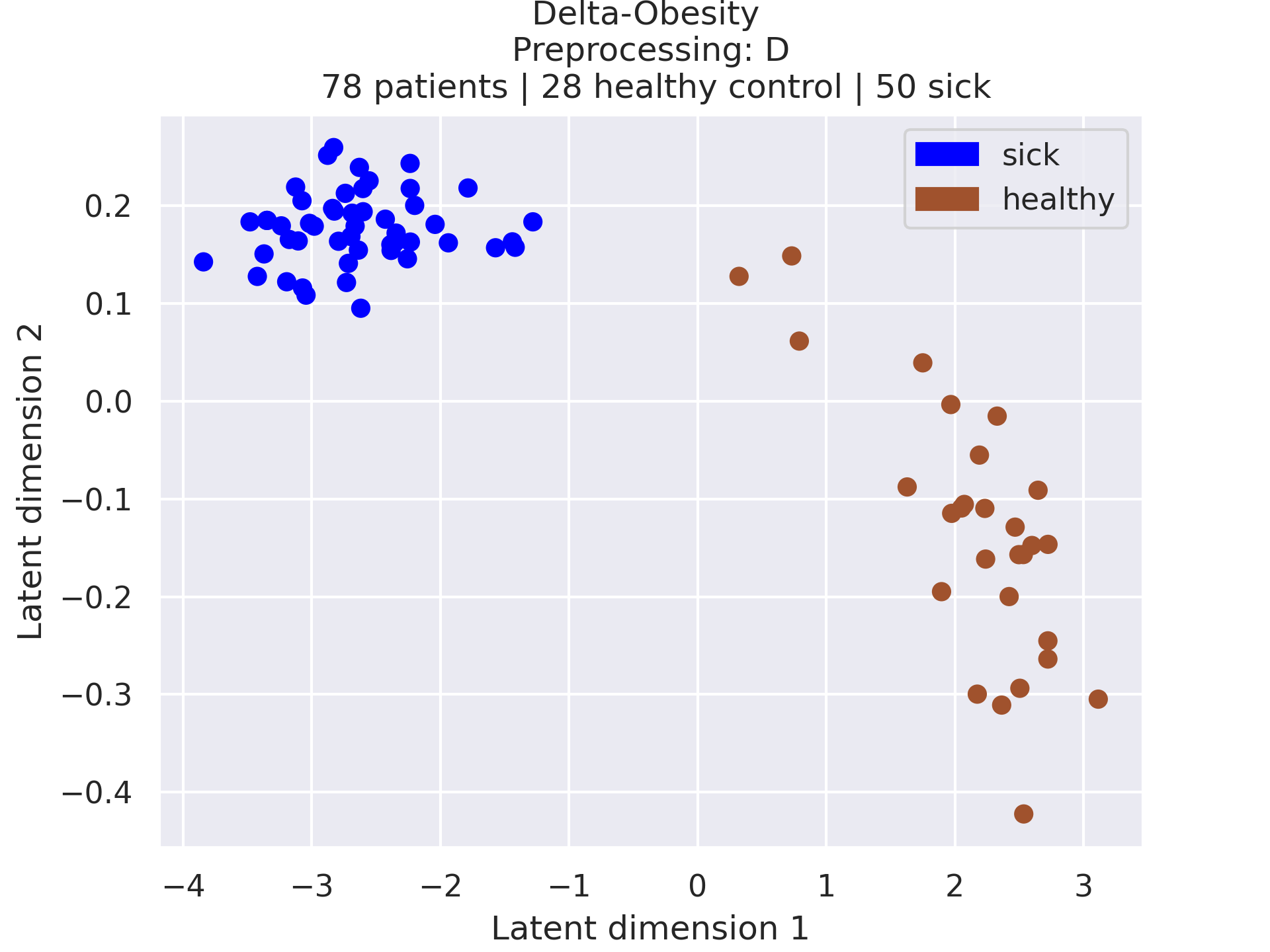

Supplement: S3 File — This file presents, for each dataset, the plots of the PCA 2D projections, as well as the plots of the mean of the MVIB 2D stochastic encodings. For the MVIB stochastic encodings z∼p(z|x)=N(μ,σ2I), the depicted points represent the mean μ. The K dimension of the latent space has been set to 2 in order to allow a 2D visualisation of the encodings. For training MVIB, the JMVIB−T objective (Eq 8) has been optimised. For MVIB, five copies of the means plots are available, as they are obtained by training the model with five different independent training-test random splits. Both the PCA and the MVIB plots have been created starting from the default datasets collection. (ZIP) [file pcbi.1010050.s008.zip › s6-file/Delta-Obesity/2_embeddings.png]

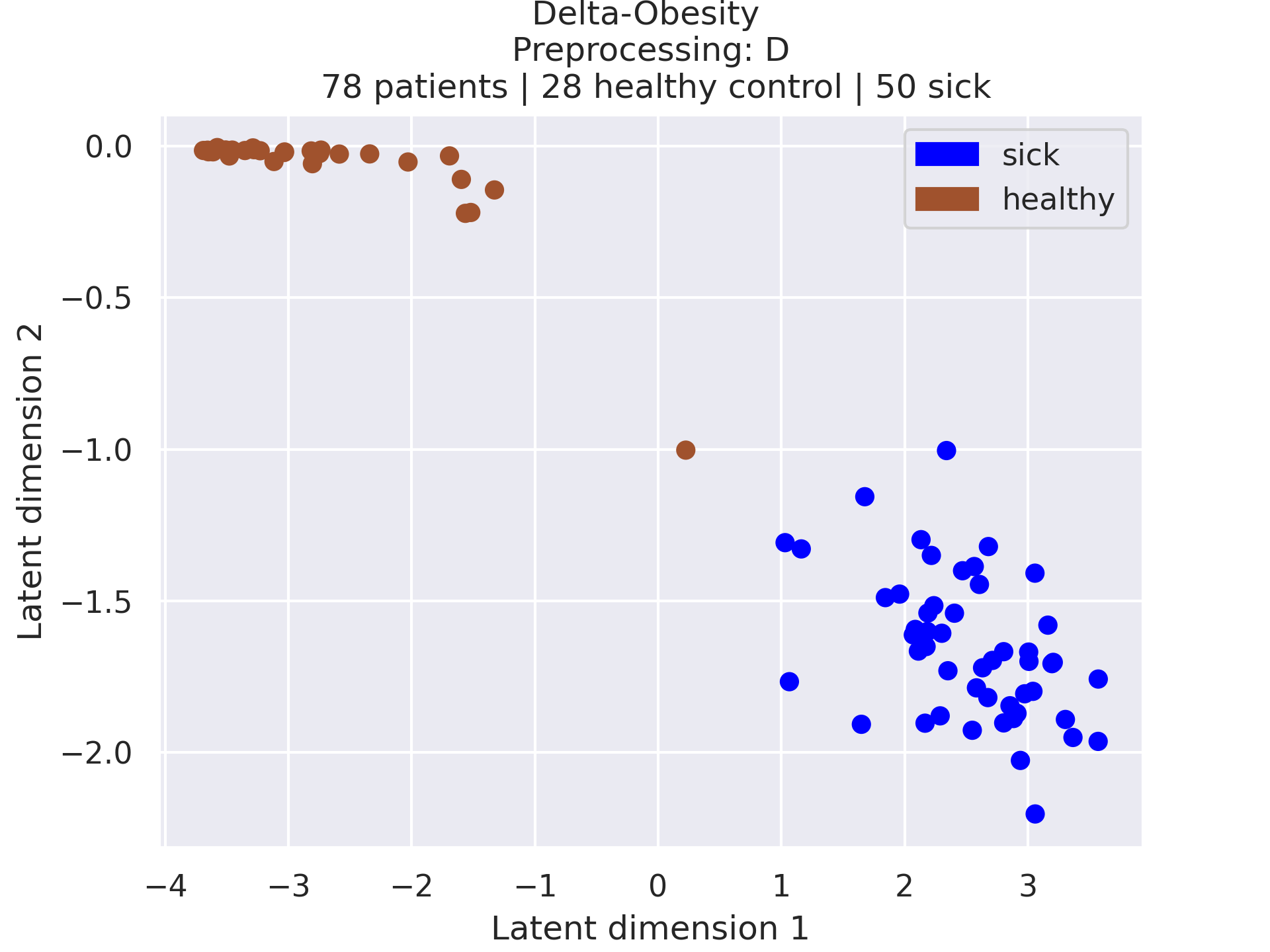

Supplement: S3 File — This file presents, for each dataset, the plots of the PCA 2D projections, as well as the plots of the mean of the MVIB 2D stochastic encodings. For the MVIB stochastic encodings z∼p(z|x)=N(μ,σ2I), the depicted points represent the mean μ. The K dimension of the latent space has been set to 2 in order to allow a 2D visualisation of the encodings. For training MVIB, the JMVIB−T objective (Eq 8) has been optimised. For MVIB, five copies of the means plots are available, as they are obtained by training the model with five different independent training-test random splits. Both the PCA and the MVIB plots have been created starting from the default datasets collection. (ZIP) [file pcbi.1010050.s008.zip › s6-file/Delta-Obesity/0_embeddings.png]

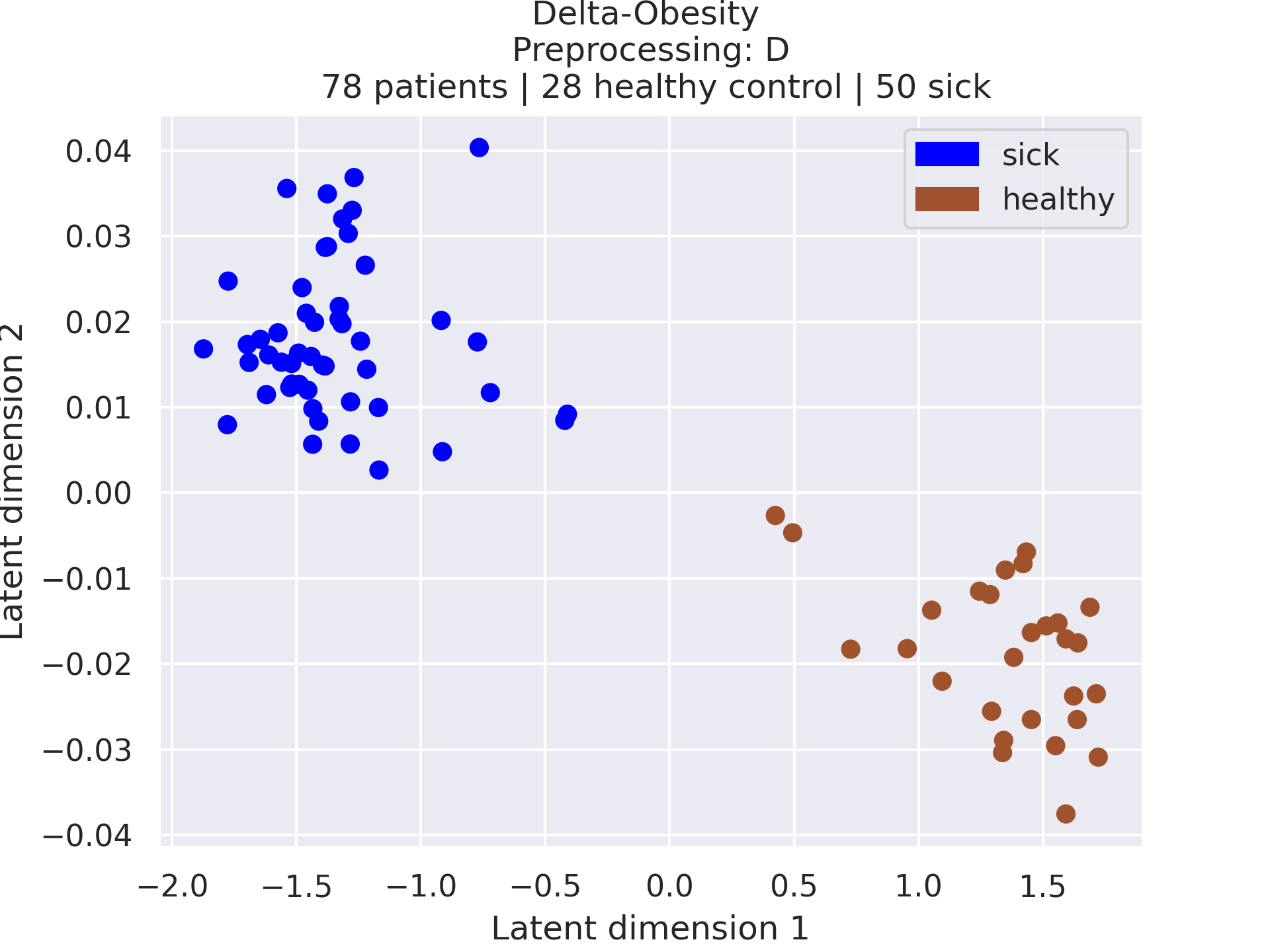

Supplement: S3 File — This file presents, for each dataset, the plots of the PCA 2D projections, as well as the plots of the mean of the MVIB 2D stochastic encodings. For the MVIB stochastic encodings z∼p(z|x)=N(μ,σ2I), the depicted points represent the mean μ. The K dimension of the latent space has been set to 2 in order to allow a 2D visualisation of the encodings. For training MVIB, the JMVIB−T objective (Eq 8) has been optimised. For MVIB, five copies of the means plots are available, as they are obtained by training the model with five different independent training-test random splits. Both the PCA and the MVIB plots have been created starting from the default datasets collection. (ZIP) [file pcbi.1010050.s008.zip › s6-file/Delta-Obesity/1_embeddings.png]

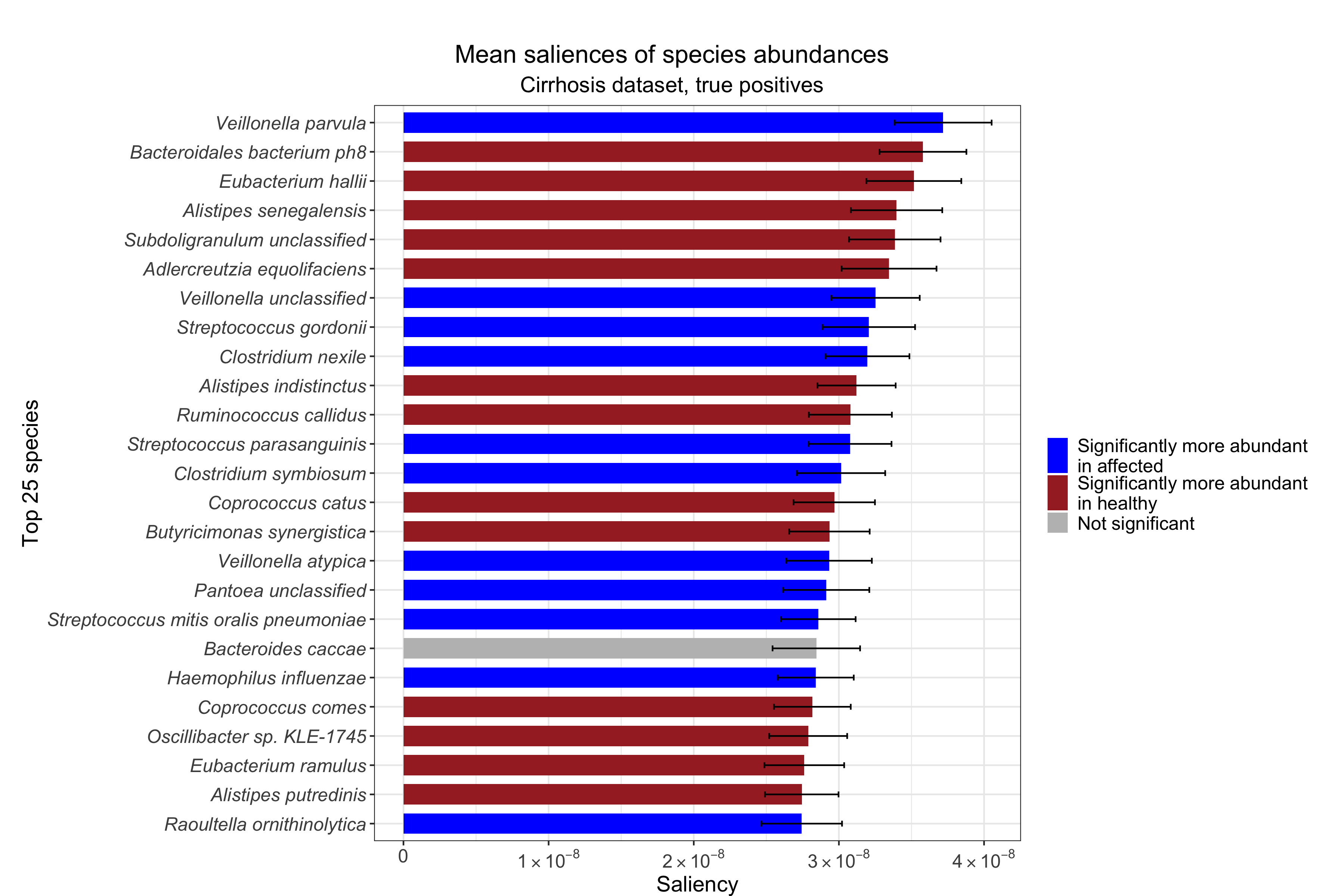

Supplement: S4 File — These files present the plots of the top 25 microbial species and strain markers for all datasets considered in this work, analogous to what Fig 5A depicts for the species from the Colorectal-EMBL dataset. Additionally, the scripts used to create the plots are included. (ZIP) [file pcbi.1010050.s009.zip › s8-file/Cirrhosis/abundance_errbarplot_TP_saliences_no_rescale_pval-0.1.png]

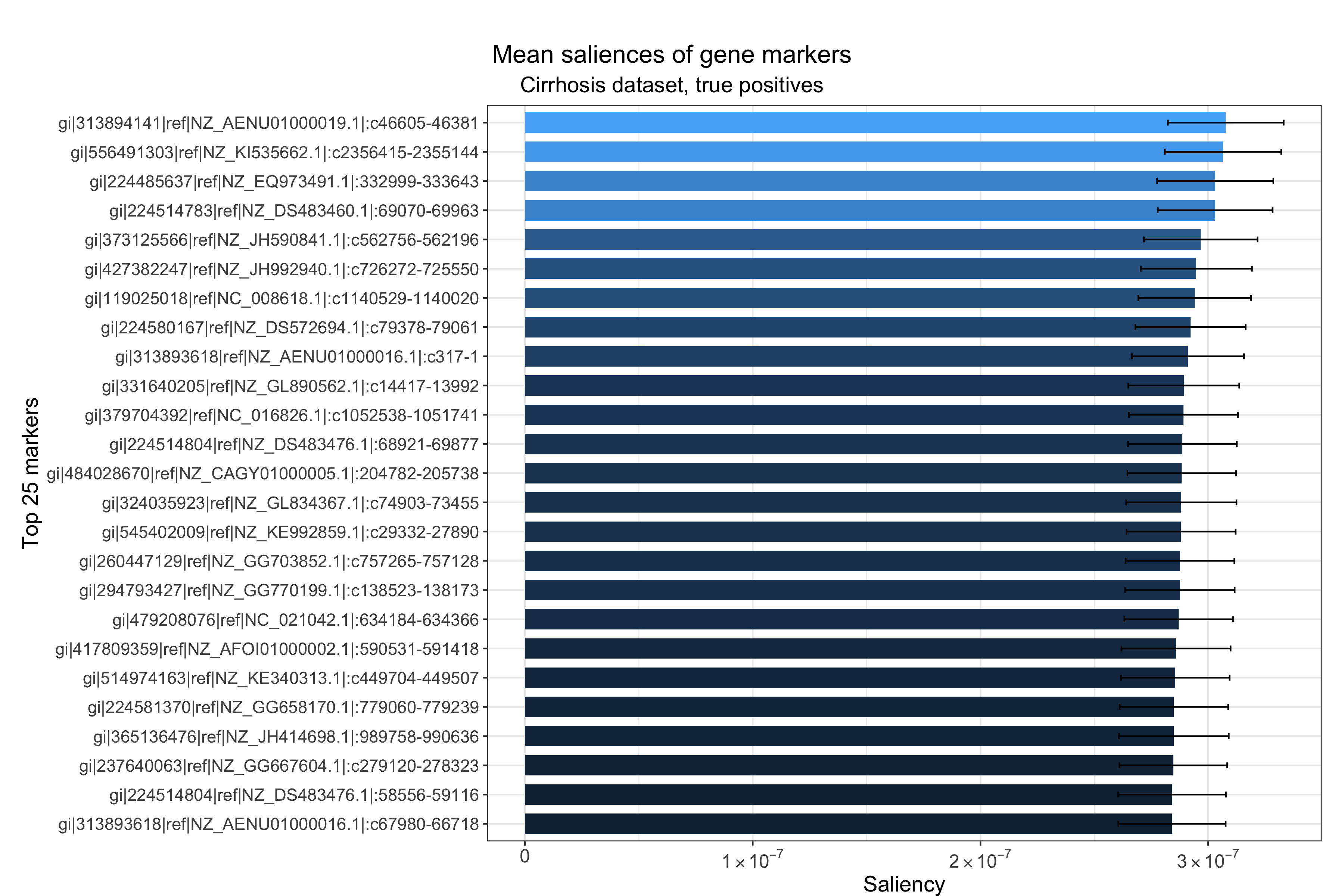

Supplement: S4 File — These files present the plots of the top 25 microbial species and strain markers for all datasets considered in this work, analogous to what Fig 5A depicts for the species from the Colorectal-EMBL dataset. Additionally, the scripts used to create the plots are included. (ZIP) [file pcbi.1010050.s009.zip › s8-file/Cirrhosis/markers_errbarplot_TP_saliences_no_rescale.png]

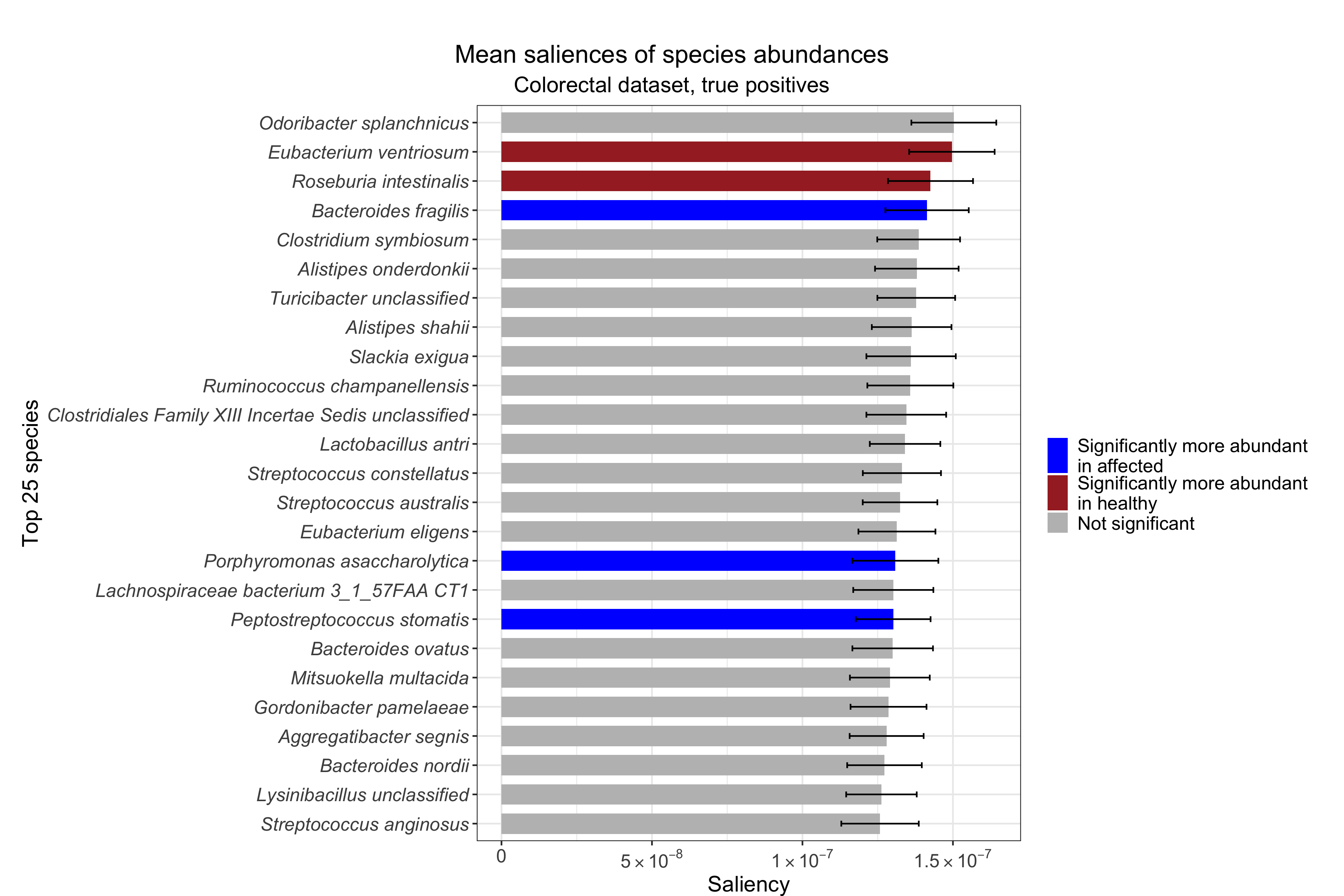

Supplement: S4 File — These files present the plots of the top 25 microbial species and strain markers for all datasets considered in this work, analogous to what Fig 5A depicts for the species from the Colorectal-EMBL dataset. Additionally, the scripts used to create the plots are included. (ZIP) [file pcbi.1010050.s009.zip › s8-file/Colorectal/abundance_errbarplot_TP_saliences_no_rescale_pval-0.1.png]

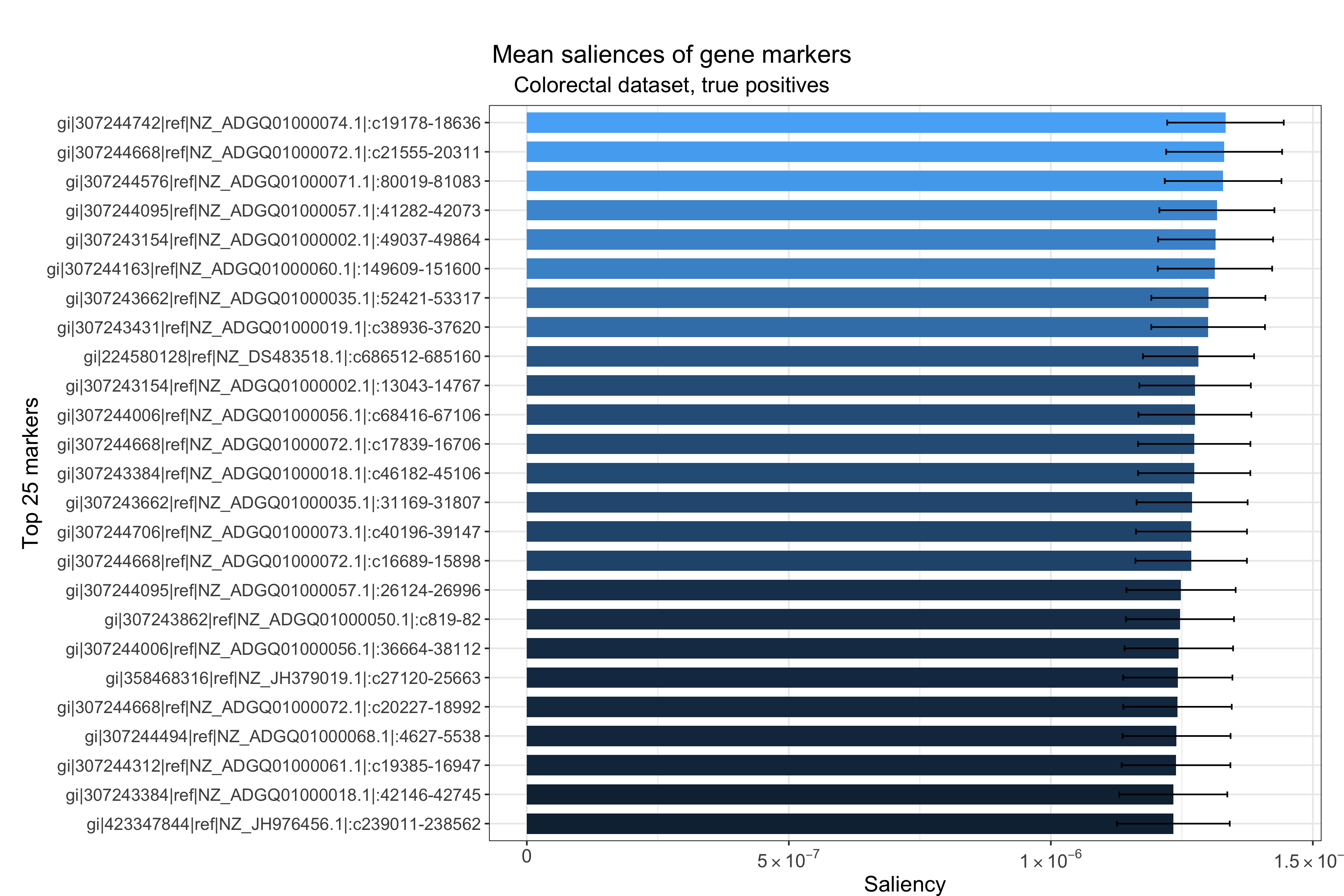

Supplement: S4 File — These files present the plots of the top 25 microbial species and strain markers for all datasets considered in this work, analogous to what Fig 5A depicts for the species from the Colorectal-EMBL dataset. Additionally, the scripts used to create the plots are included. (ZIP) [file pcbi.1010050.s009.zip › s8-file/Colorectal/markers_errbarplot_TP_saliences_no_rescale.png]

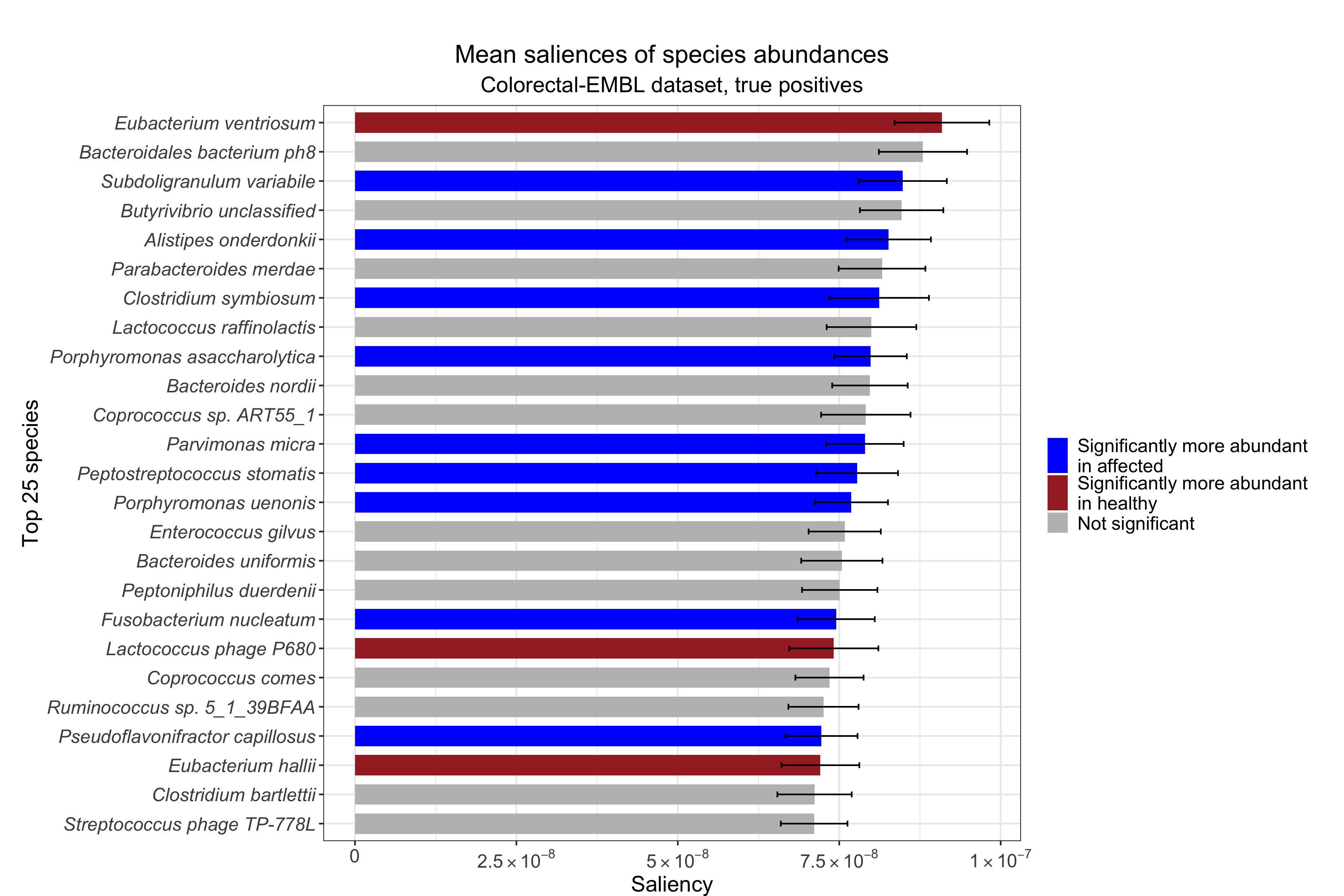

Supplement: S4 File — These files present the plots of the top 25 microbial species and strain markers for all datasets considered in this work, analogous to what Fig 5A depicts for the species from the Colorectal-EMBL dataset. Additionally, the scripts used to create the plots are included. (ZIP) [file pcbi.1010050.s009.zip › s8-file/Colorectal-EMBL/abundance_errbarplot_TP_saliences_no_rescale_pval-0.1.png]

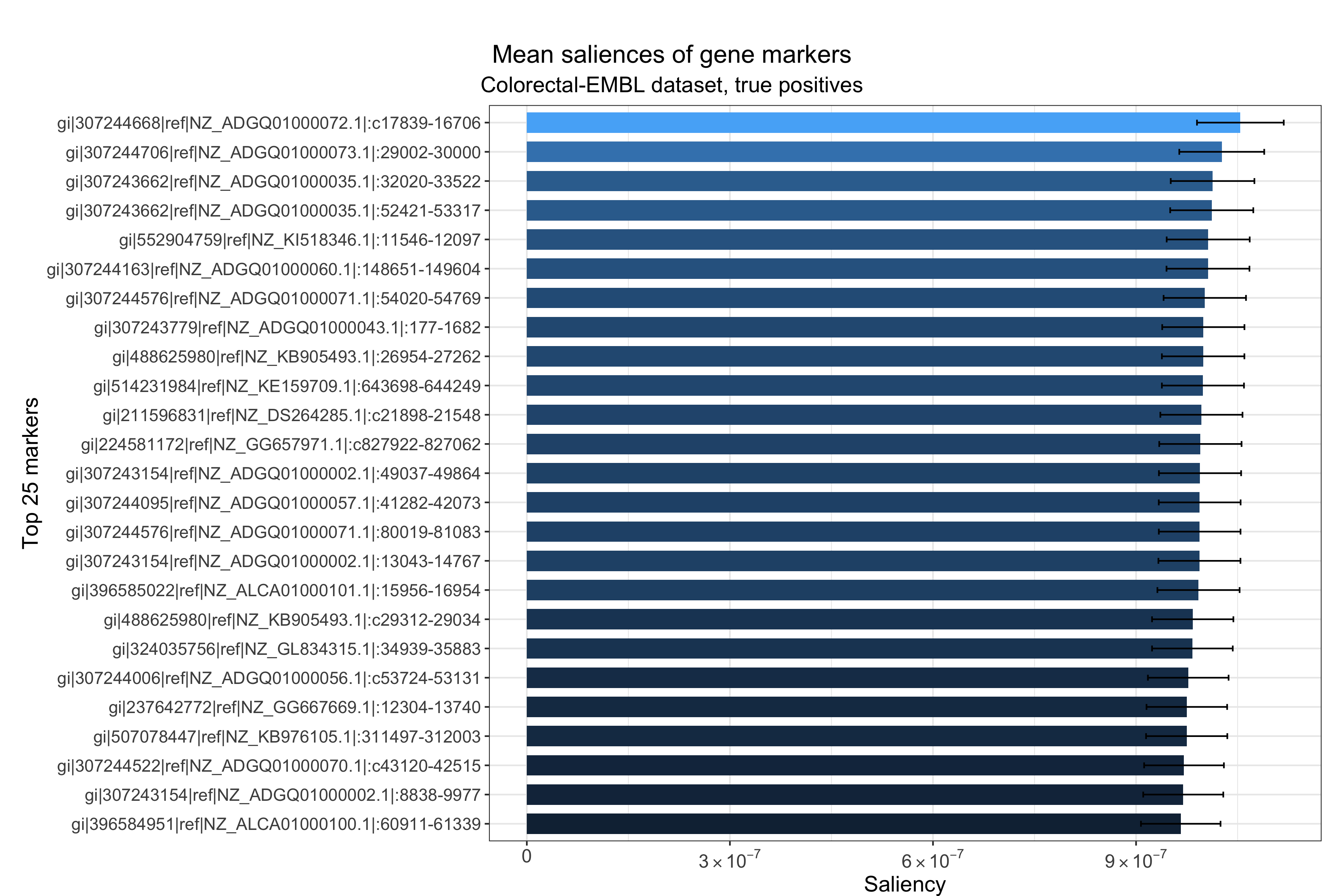

Supplement: S4 File — These files present the plots of the top 25 microbial species and strain markers for all datasets considered in this work, analogous to what Fig 5A depicts for the species from the Colorectal-EMBL dataset. Additionally, the scripts used to create the plots are included. (ZIP) [file pcbi.1010050.s009.zip › s8-file/Colorectal-EMBL/markers_errbarplot_TP_saliences_no_rescale.png]

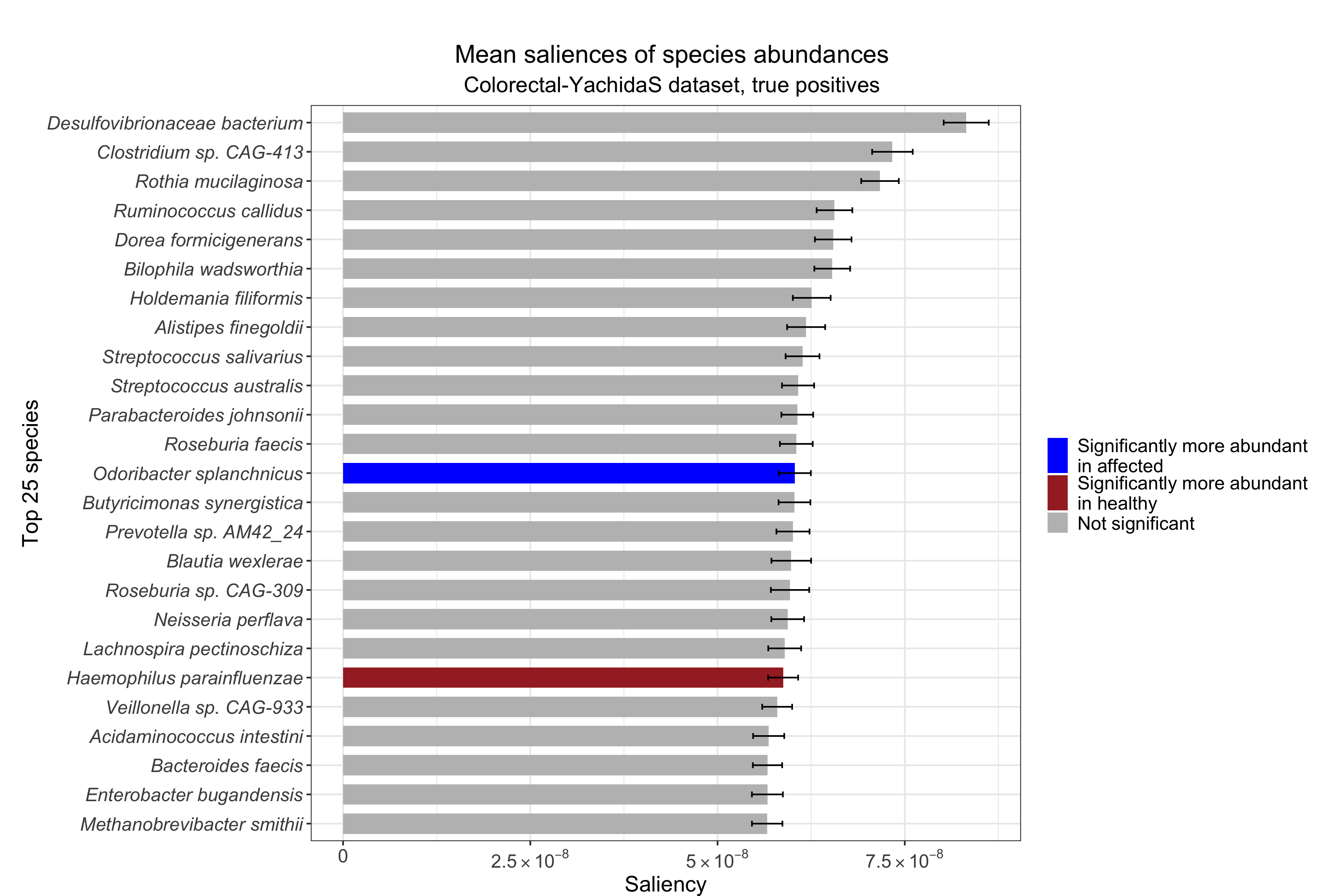

Supplement: S4 File — These files present the plots of the top 25 microbial species and strain markers for all datasets considered in this work, analogous to what Fig 5A depicts for the species from the Colorectal-EMBL dataset. Additionally, the scripts used to create the plots are included. (ZIP) [file pcbi.1010050.s009.zip › s8-file/Colorectal-YachidaS/abundance_errbarplot_TP_saliences_no_rescale_pval-0.1.png]

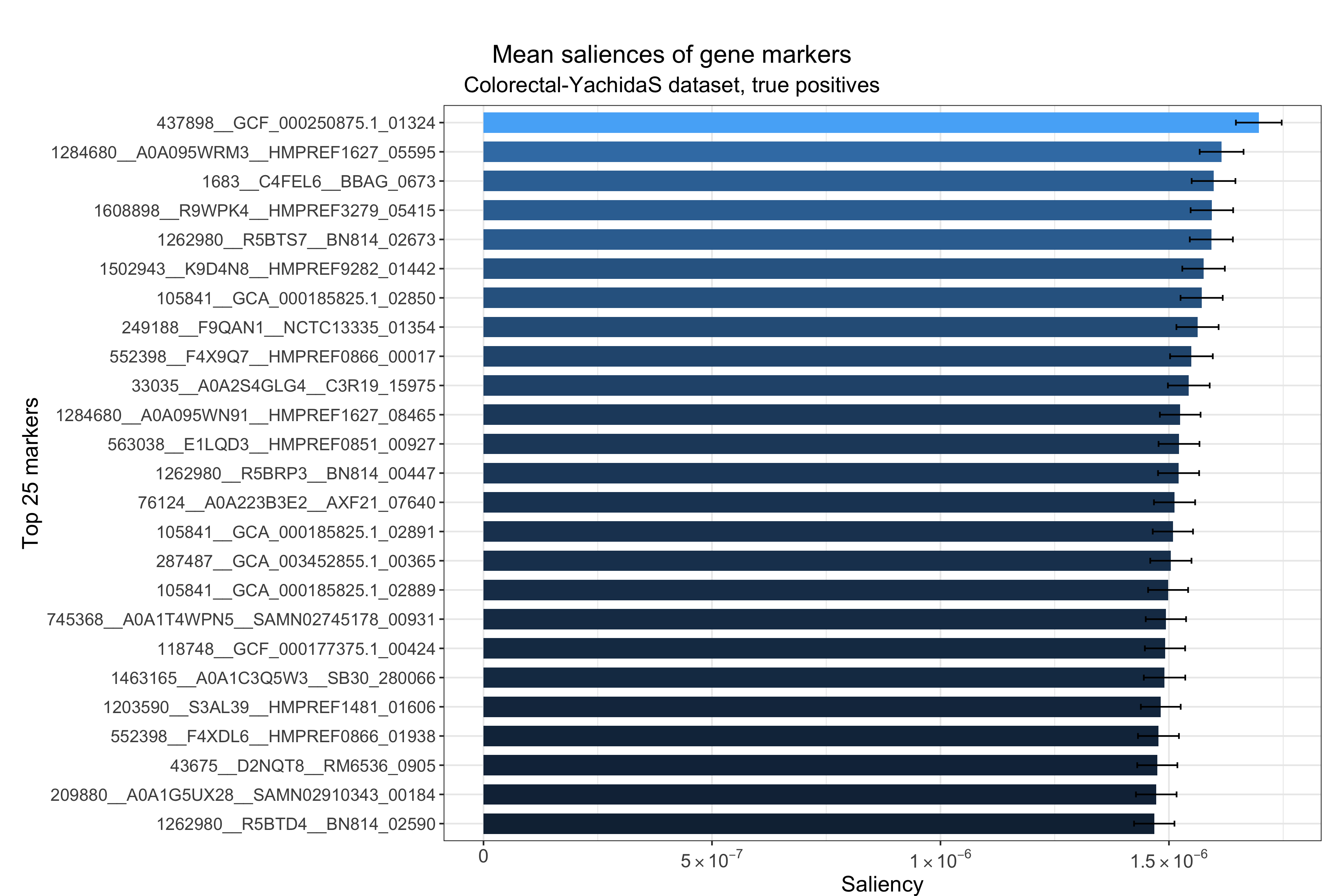

Supplement: S4 File — These files present the plots of the top 25 microbial species and strain markers for all datasets considered in this work, analogous to what Fig 5A depicts for the species from the Colorectal-EMBL dataset. Additionally, the scripts used to create the plots are included. (ZIP) [file pcbi.1010050.s009.zip › s8-file/Colorectal-YachidaS/markers_errbarplot_TP_saliences_no_rescale.png]

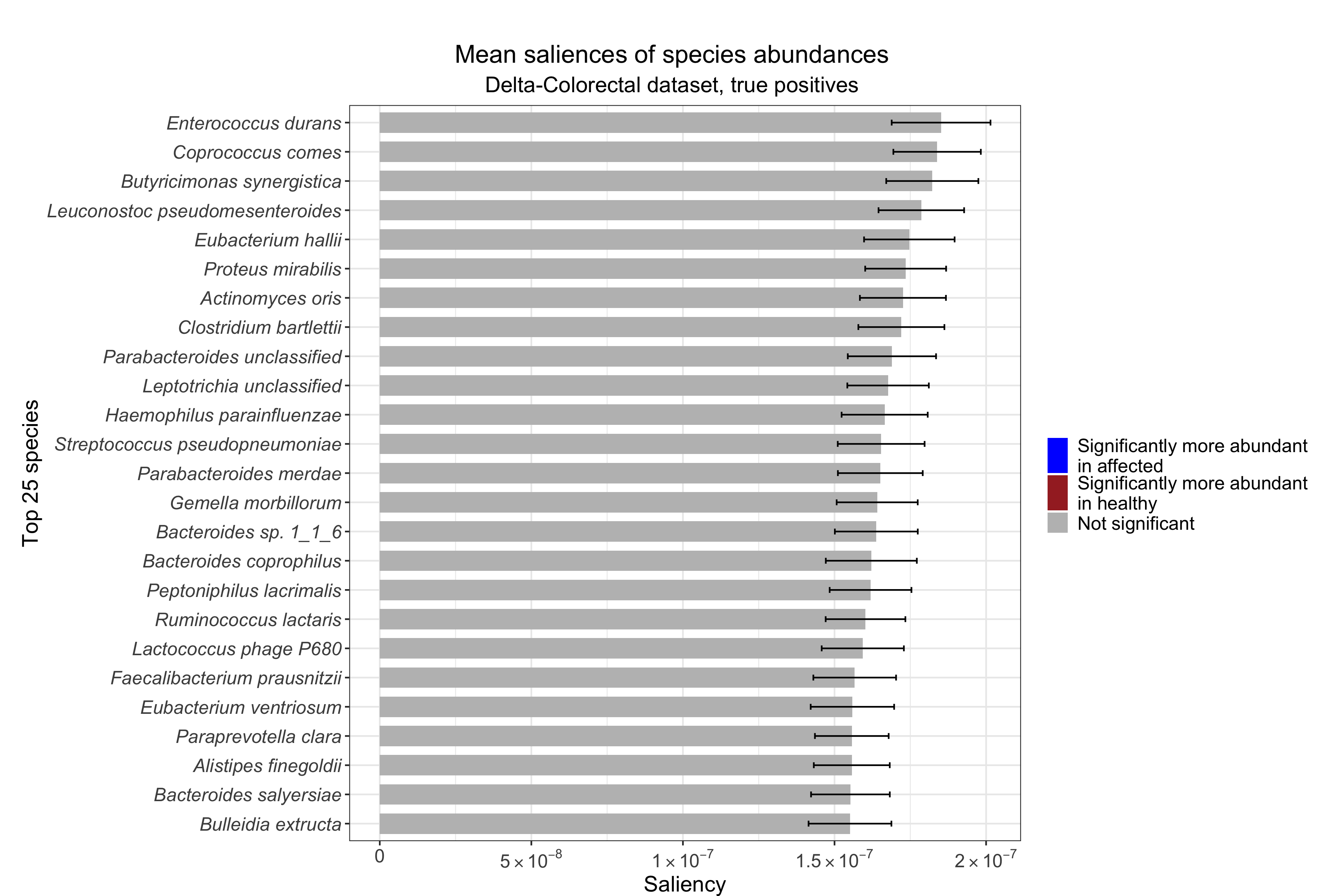

Supplement: S4 File — These files present the plots of the top 25 microbial species and strain markers for all datasets considered in this work, analogous to what Fig 5A depicts for the species from the Colorectal-EMBL dataset. Additionally, the scripts used to create the plots are included. (ZIP) [file pcbi.1010050.s009.zip › s8-file/Delta-Colorectal/abundance_errbarplot_TP_saliences_no_rescale_pval-0.1.png]

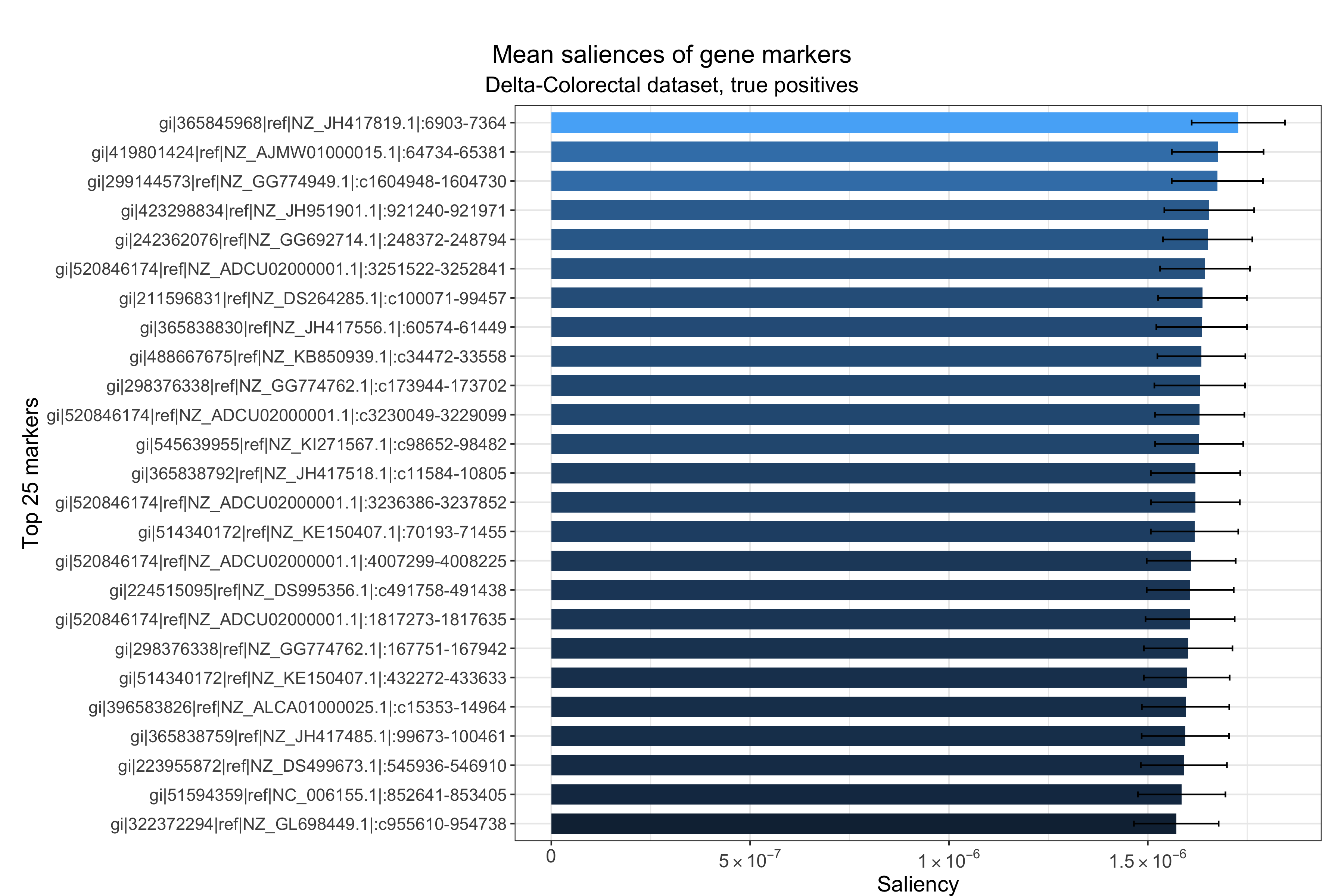

Supplement: S4 File — These files present the plots of the top 25 microbial species and strain markers for all datasets considered in this work, analogous to what Fig 5A depicts for the species from the Colorectal-EMBL dataset. Additionally, the scripts used to create the plots are included. (ZIP) [file pcbi.1010050.s009.zip › s8-file/Delta-Colorectal/markers_errbarplot_TP_saliences_no_rescale.png]

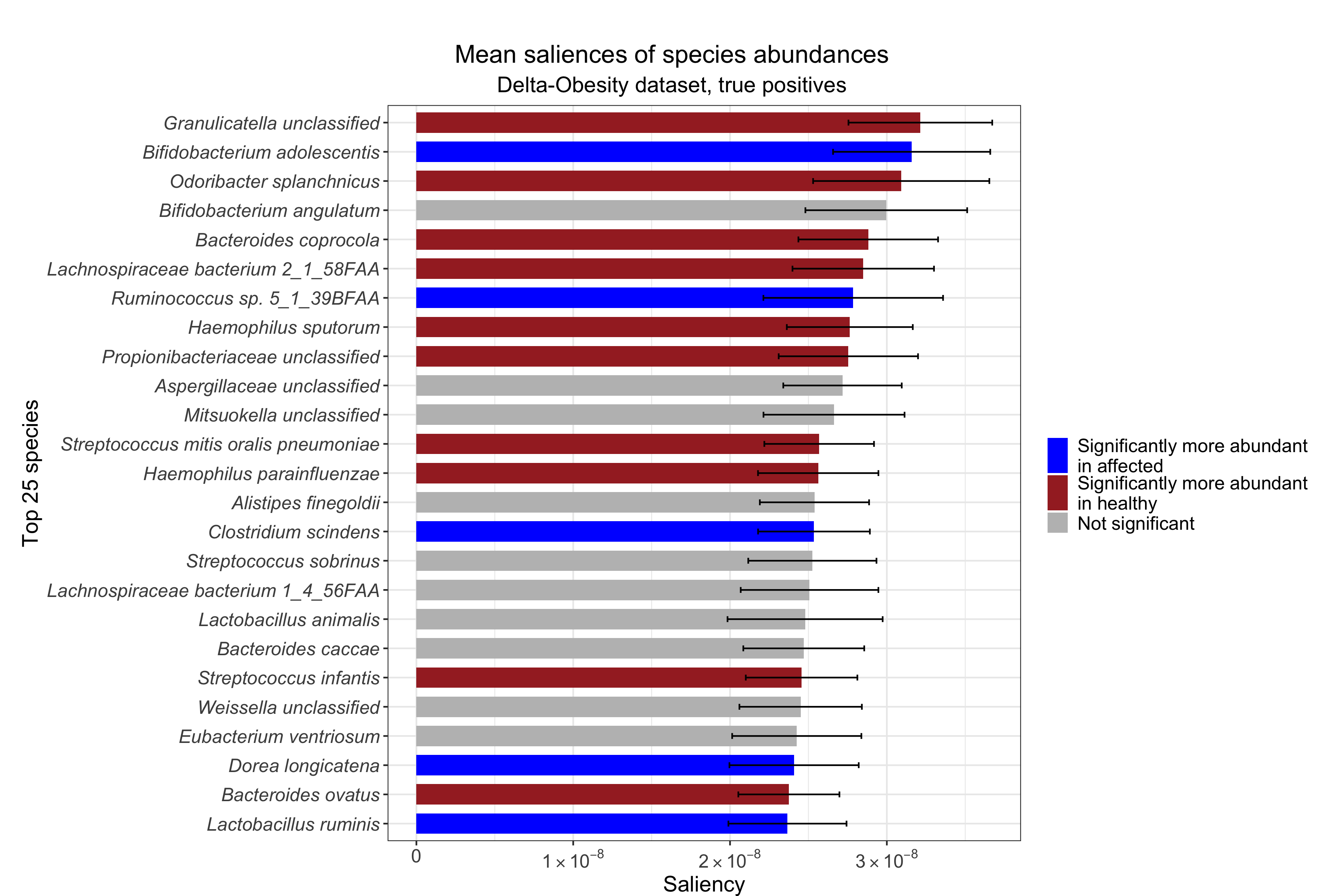

Supplement: S4 File — These files present the plots of the top 25 microbial species and strain markers for all datasets considered in this work, analogous to what Fig 5A depicts for the species from the Colorectal-EMBL dataset. Additionally, the scripts used to create the plots are included. (ZIP) [file pcbi.1010050.s009.zip › s8-file/Delta-Obesity/abundance_errbarplot_TP_saliences_no_rescale_pval-0.1.png]

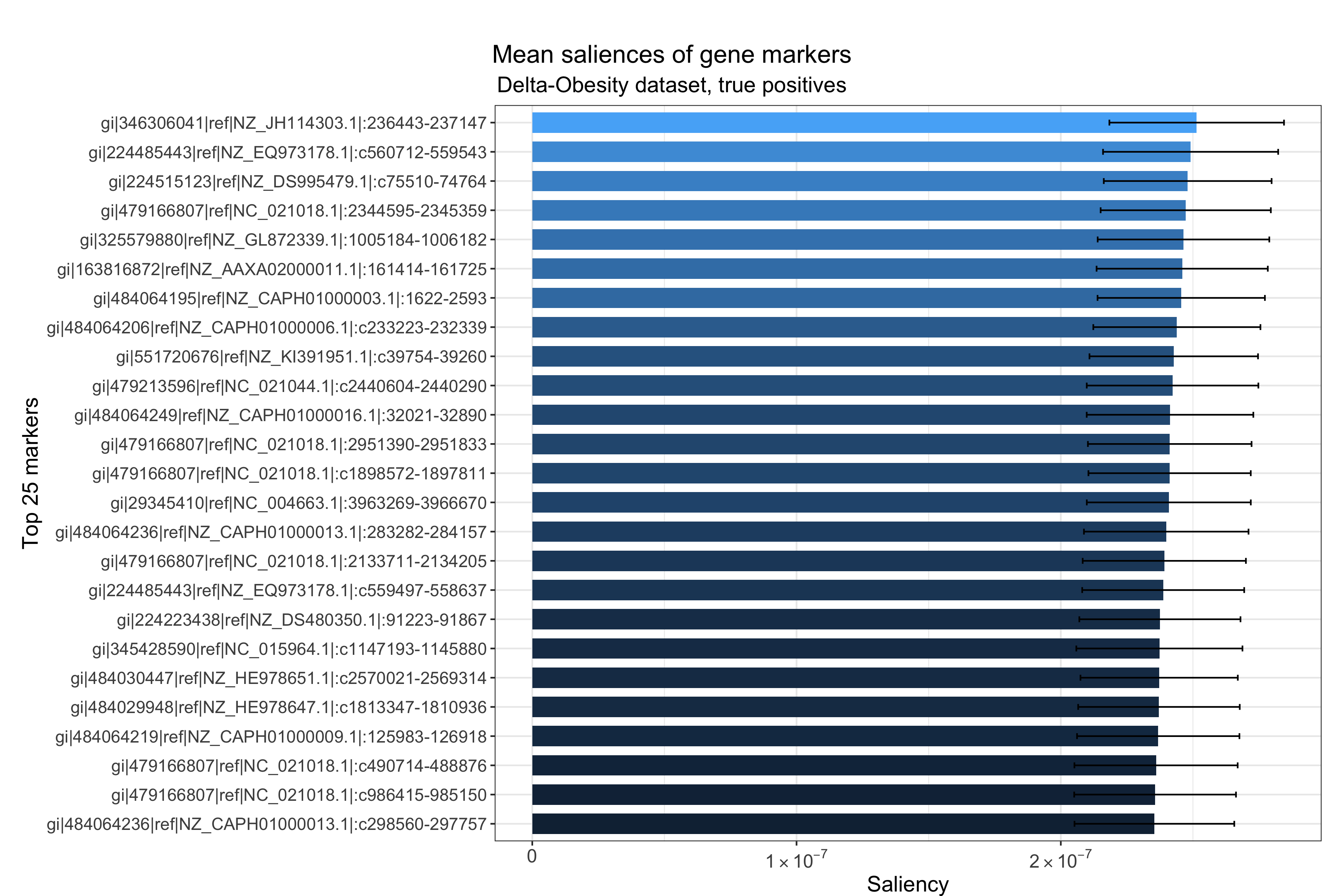

Supplement: S4 File — These files present the plots of the top 25 microbial species and strain markers for all datasets considered in this work, analogous to what Fig 5A depicts for the species from the Colorectal-EMBL dataset. Additionally, the scripts used to create the plots are included. (ZIP) [file pcbi.1010050.s009.zip › s8-file/Delta-Obesity/markers_errbarplot_TP_saliences_no_rescale.png]

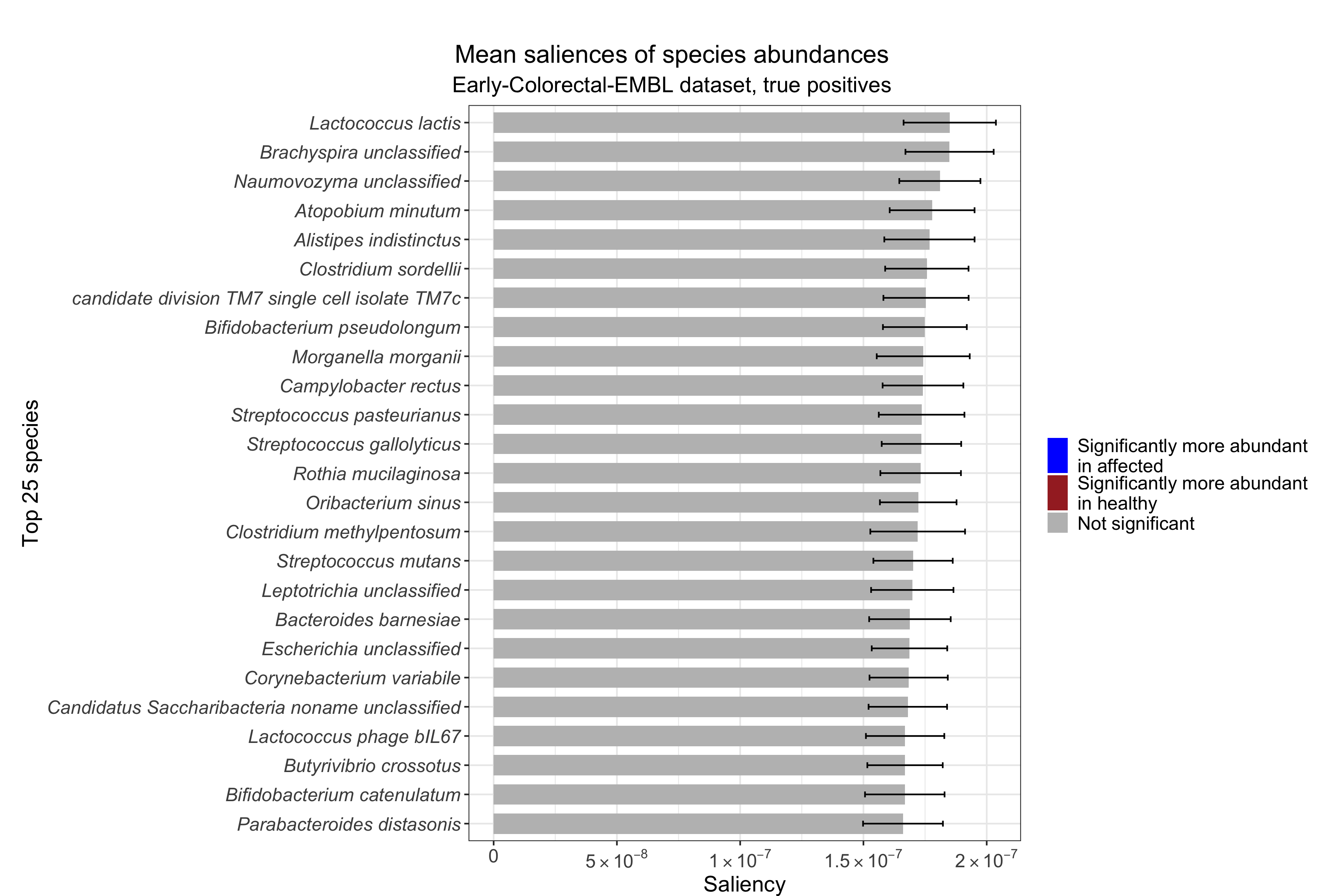

Supplement: S4 File — These files present the plots of the top 25 microbial species and strain markers for all datasets considered in this work, analogous to what Fig 5A depicts for the species from the Colorectal-EMBL dataset. Additionally, the scripts used to create the plots are included. (ZIP) [file pcbi.1010050.s009.zip › s8-file/Early-Colorectal-EMBL/abundance_errbarplot_TP_saliences_no_rescale_pval-0.1.png]

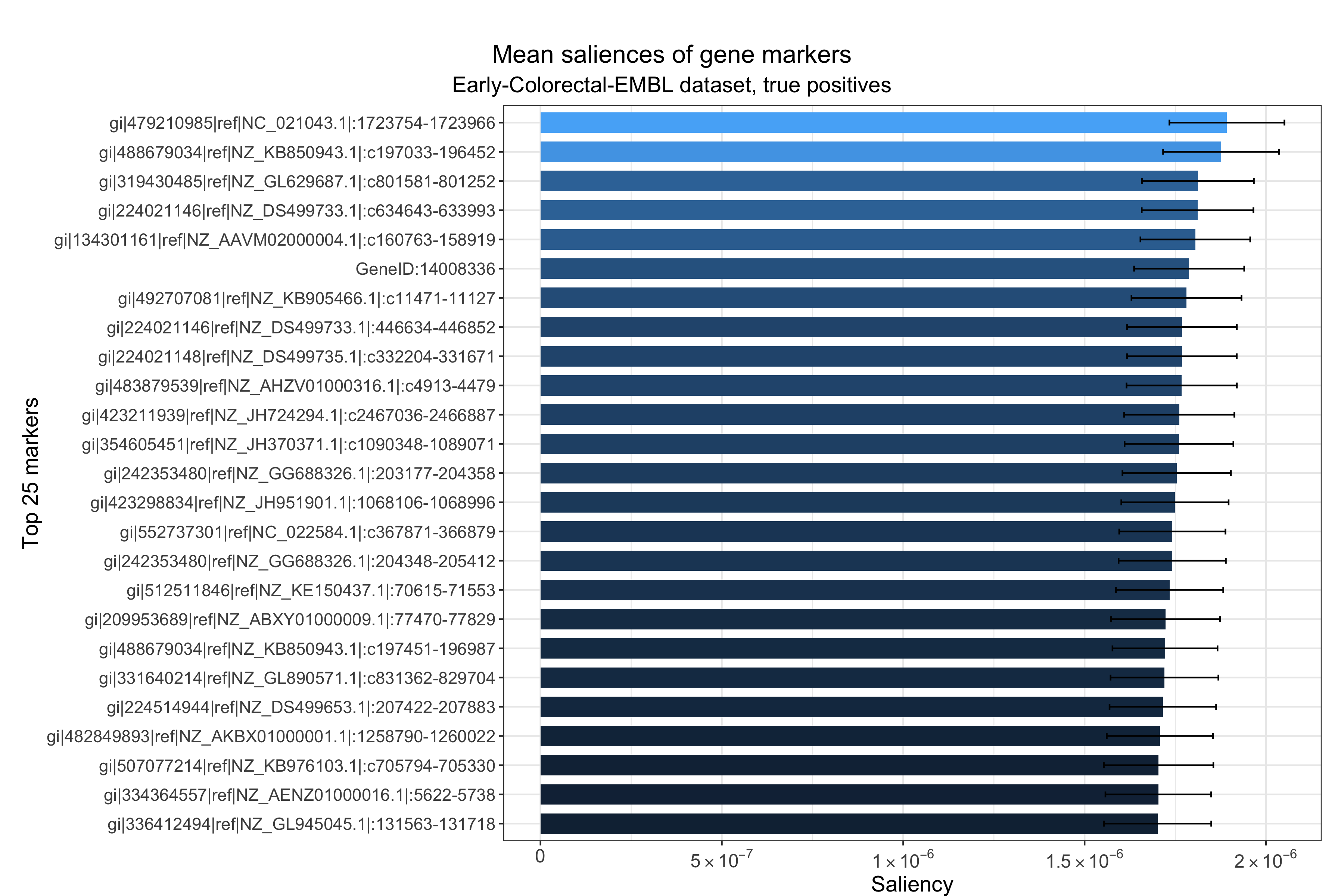

Supplement: S4 File — These files present the plots of the top 25 microbial species and strain markers for all datasets considered in this work, analogous to what Fig 5A depicts for the species from the Colorectal-EMBL dataset. Additionally, the scripts used to create the plots are included. (ZIP) [file pcbi.1010050.s009.zip › s8-file/Early-Colorectal-EMBL/markers_errbarplot_TP_saliences_no_rescale.png]

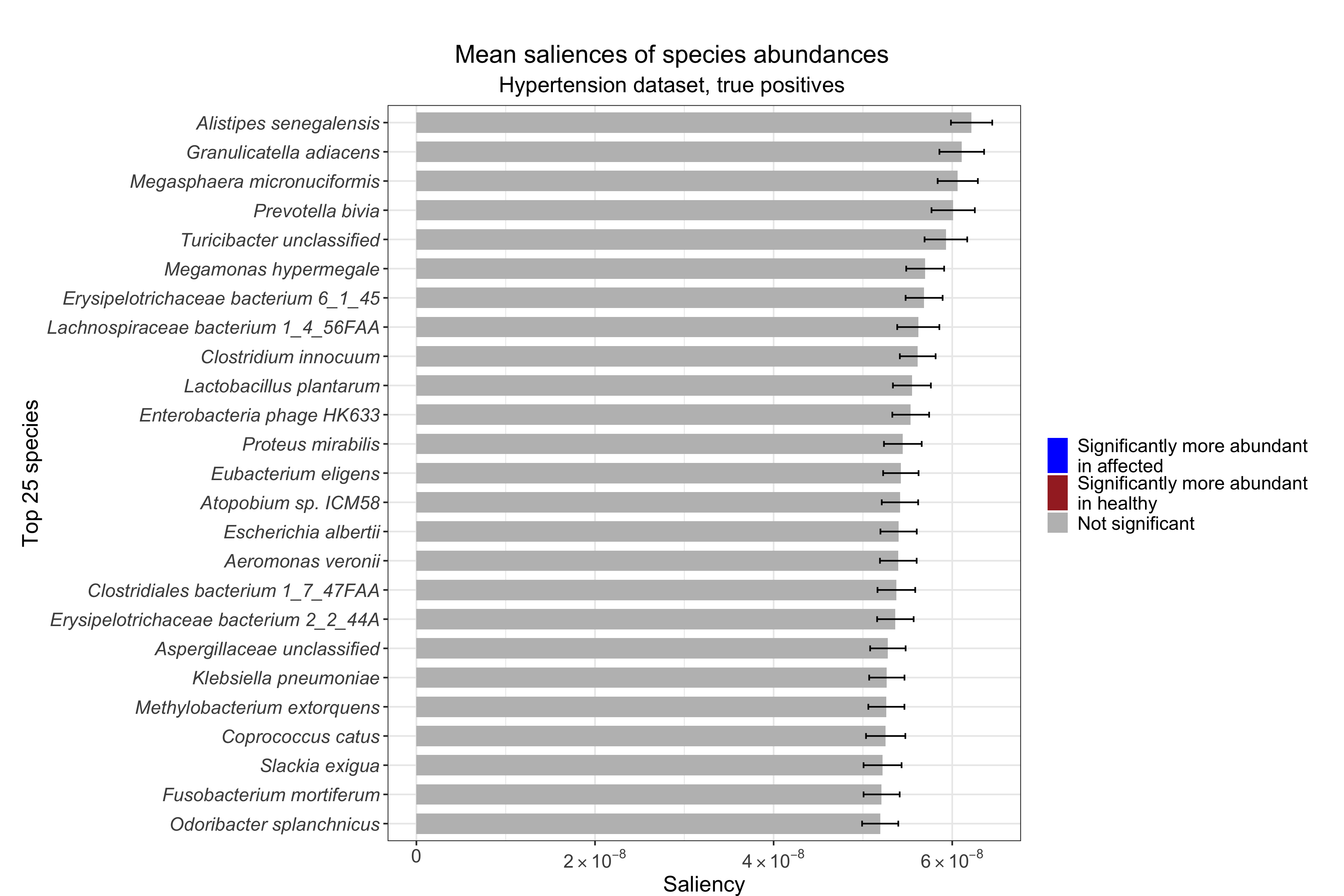

Supplement: S4 File — These files present the plots of the top 25 microbial species and strain markers for all datasets considered in this work, analogous to what Fig 5A depicts for the species from the Colorectal-EMBL dataset. Additionally, the scripts used to create the plots are included. (ZIP) [file pcbi.1010050.s009.zip › s8-file/Hypertension/abundance_errbarplot_TP_saliences_no_rescale_pval-0.1.png]

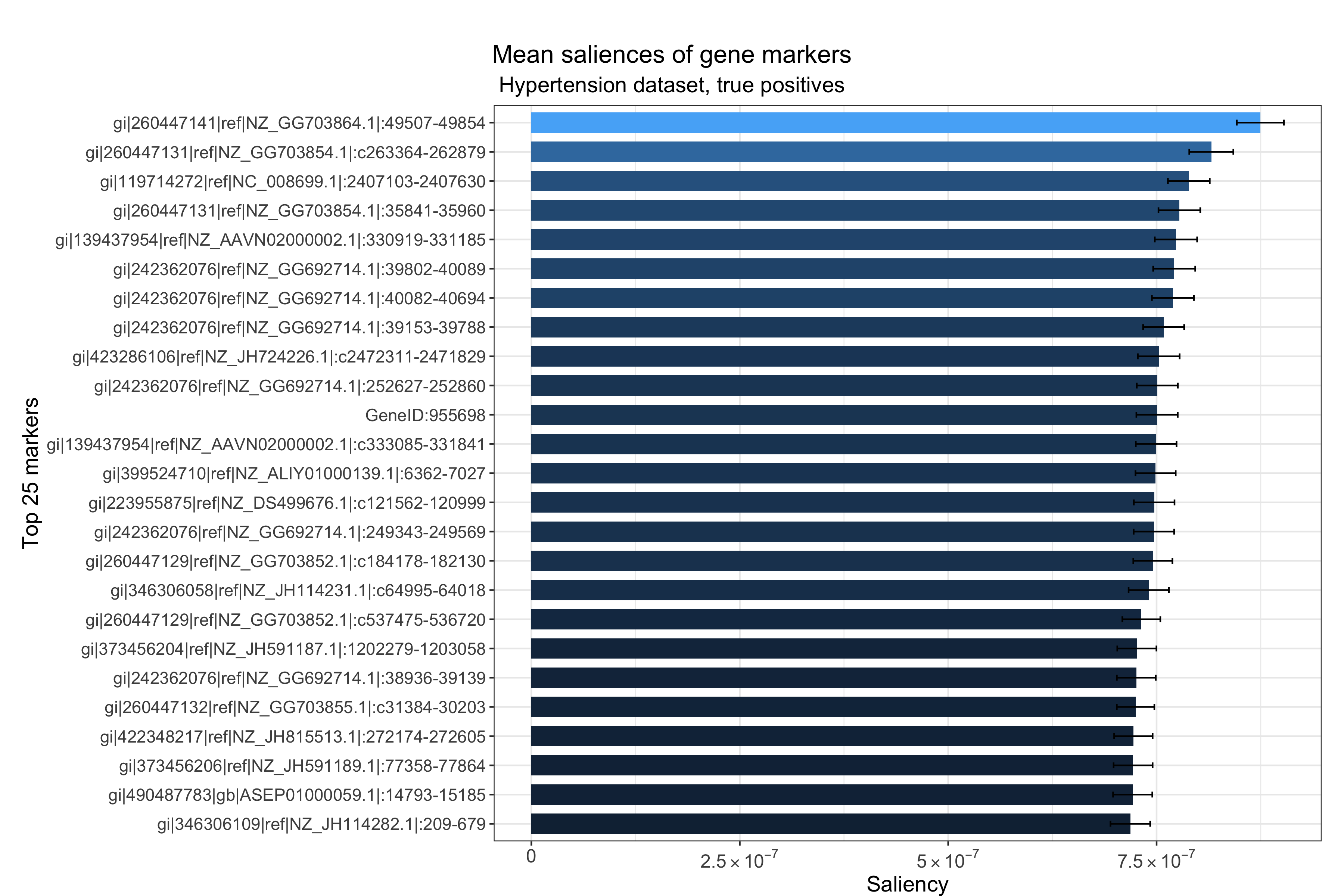

Supplement: S4 File — These files present the plots of the top 25 microbial species and strain markers for all datasets considered in this work, analogous to what Fig 5A depicts for the species from the Colorectal-EMBL dataset. Additionally, the scripts used to create the plots are included. (ZIP) [file pcbi.1010050.s009.zip › s8-file/Hypertension/markers_errbarplot_TP_saliences_no_rescale.png]

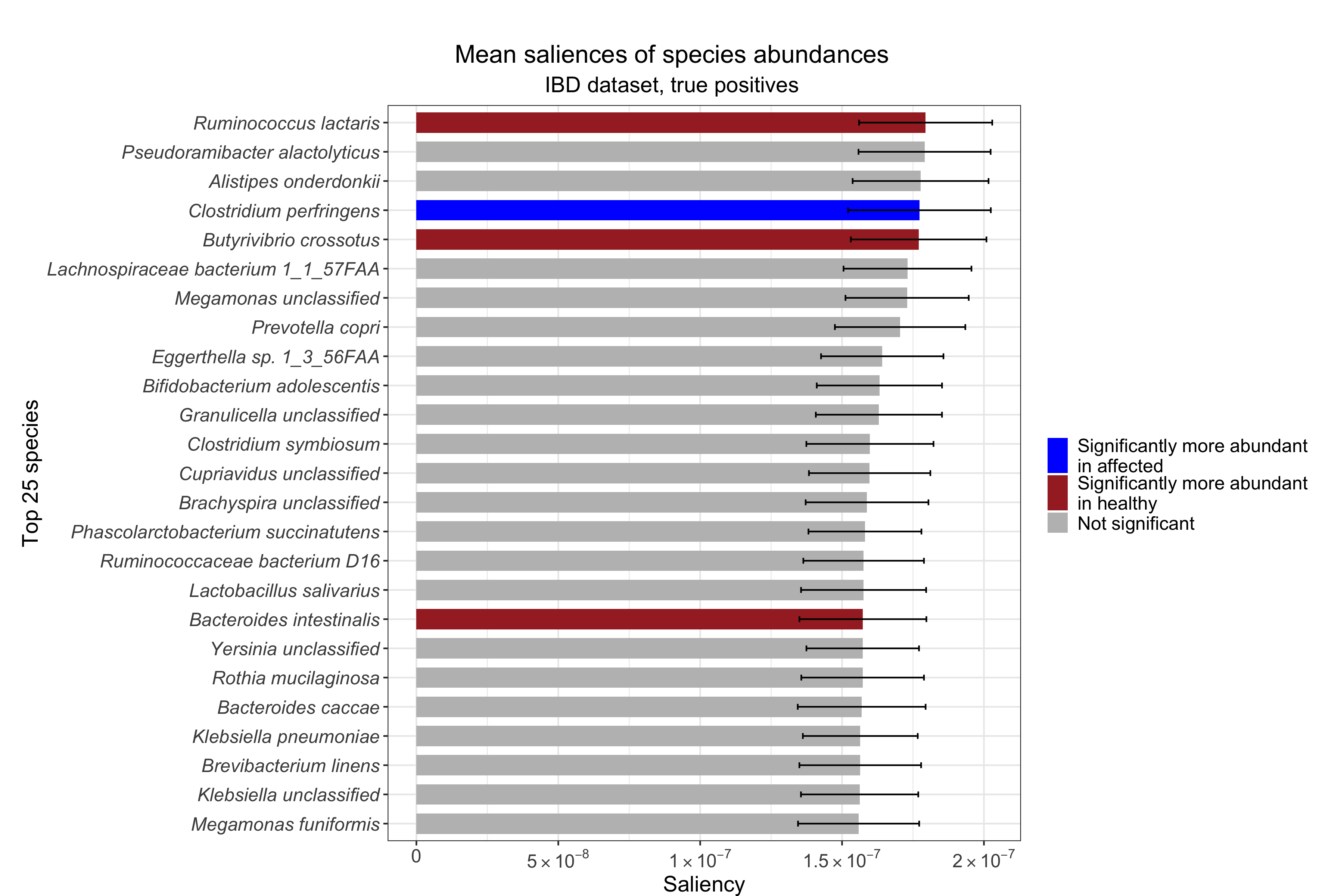

Supplement: S4 File — These files present the plots of the top 25 microbial species and strain markers for all datasets considered in this work, analogous to what Fig 5A depicts for the species from the Colorectal-EMBL dataset. Additionally, the scripts used to create the plots are included. (ZIP) [file pcbi.1010050.s009.zip › s8-file/IBD/abundance_errbarplot_TP_saliences_no_rescale_pval-0.1.png]
