# Supplementary figures and images for: Microbiome-based disease prediction with multimodal variational information bottlenecks (part 2 of 4)
Source: PLoS Comput Biol. 2022 Apr 11;18(4):e1010050. doi: 10.1371/journal.pcbi.1010050 (PMC9022840; doi:10.1371/journal.pcbi.1010050)

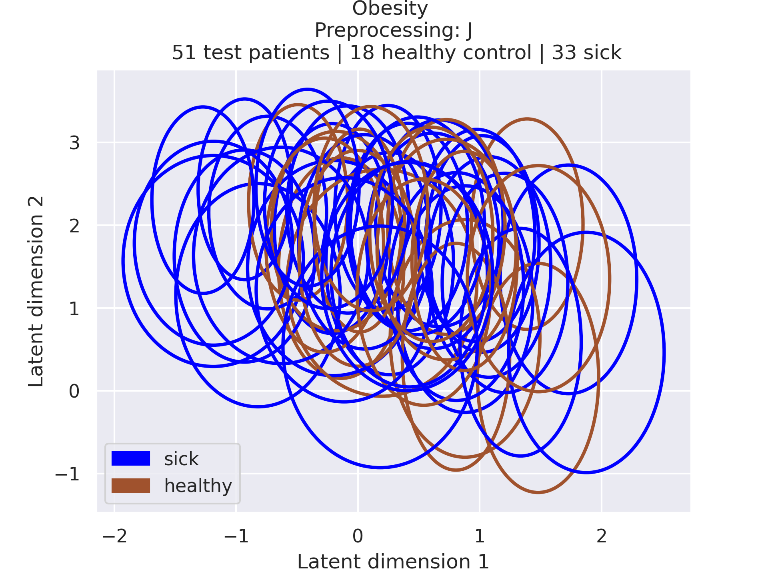

Supplement: S2 File — For all datasets considered in this work, this file presents plots of the 2D MVIB stochastic encodings analogous to Fig 3. The depicted curves are the 95% confidence intervals of the samples’ stochastic encodings z∼p(z|x)=N(μ,σ2I); the points are their means μ. The displayed encodings consist only in the test samples obtained from random training-test splits (i.e. the 20% of the dataset not used for training). The K dimension of the latent space has been set to 2 in order to allow a 2D visualisation. Plots derived from both the optimisation of the JMVIB−T objective (Eq 8) and the optimisation of the JMVIB objective (Eq 5) are included. Five copies of all plots are available, as they are obtained by training the model with five different independent training-test random splits. (ZIP) [file pcbi.1010050.s007.zip › s5-file/bce-and-triplet/J/Obesity/4_embeddings_95_confidence.png]

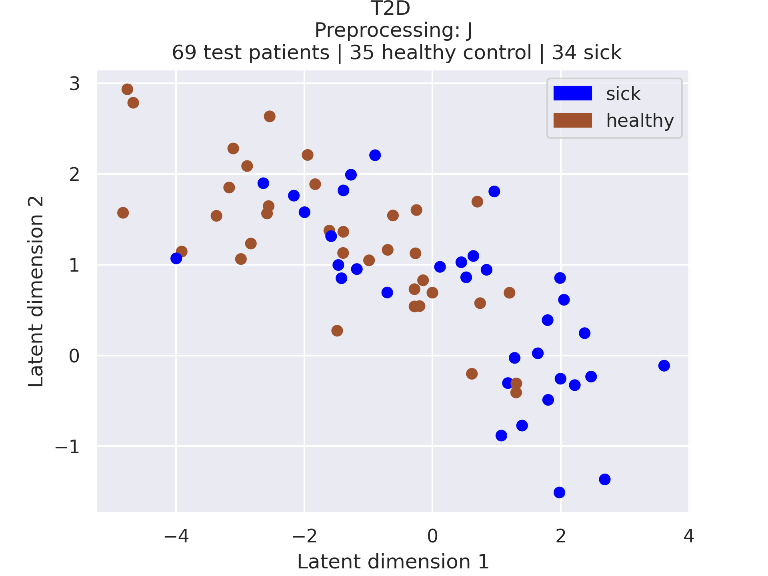

Supplement: S2 File — For all datasets considered in this work, this file presents plots of the 2D MVIB stochastic encodings analogous to Fig 3. The depicted curves are the 95% confidence intervals of the samples’ stochastic encodings z∼p(z|x)=N(μ,σ2I); the points are their means μ. The displayed encodings consist only in the test samples obtained from random training-test splits (i.e. the 20% of the dataset not used for training). The K dimension of the latent space has been set to 2 in order to allow a 2D visualisation. Plots derived from both the optimisation of the JMVIB−T objective (Eq 8) and the optimisation of the JMVIB objective (Eq 5) are included. Five copies of all plots are available, as they are obtained by training the model with five different independent training-test random splits. (ZIP) [file pcbi.1010050.s007.zip › s5-file/bce-and-triplet/J/T2D/0_embeddings.png]

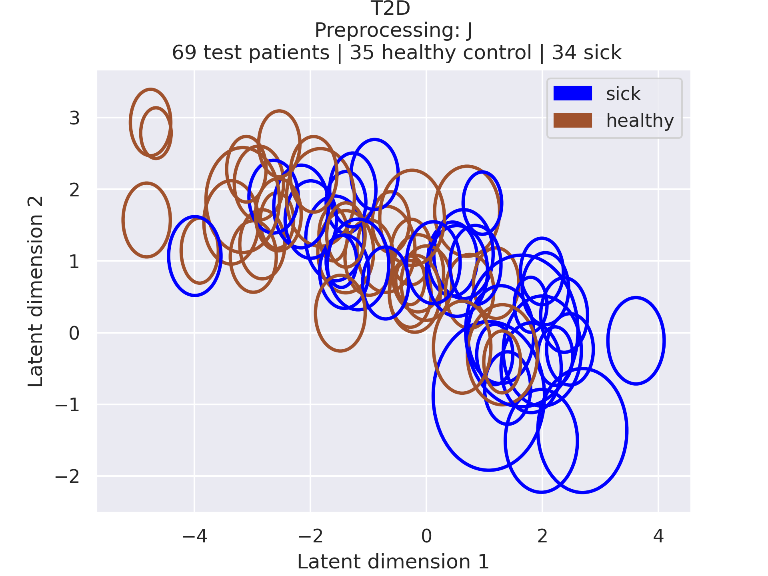

Supplement: S2 File — For all datasets considered in this work, this file presents plots of the 2D MVIB stochastic encodings analogous to Fig 3. The depicted curves are the 95% confidence intervals of the samples’ stochastic encodings z∼p(z|x)=N(μ,σ2I); the points are their means μ. The displayed encodings consist only in the test samples obtained from random training-test splits (i.e. the 20% of the dataset not used for training). The K dimension of the latent space has been set to 2 in order to allow a 2D visualisation. Plots derived from both the optimisation of the JMVIB−T objective (Eq 8) and the optimisation of the JMVIB objective (Eq 5) are included. Five copies of all plots are available, as they are obtained by training the model with five different independent training-test random splits. (ZIP) [file pcbi.1010050.s007.zip › s5-file/bce-and-triplet/J/T2D/0_embeddings_95_confidence.png]

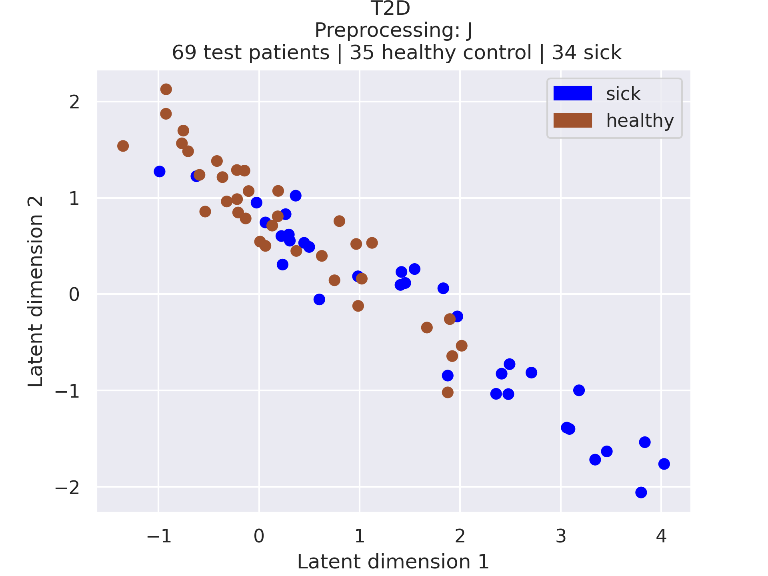

Supplement: S2 File — For all datasets considered in this work, this file presents plots of the 2D MVIB stochastic encodings analogous to Fig 3. The depicted curves are the 95% confidence intervals of the samples’ stochastic encodings z∼p(z|x)=N(μ,σ2I); the points are their means μ. The displayed encodings consist only in the test samples obtained from random training-test splits (i.e. the 20% of the dataset not used for training). The K dimension of the latent space has been set to 2 in order to allow a 2D visualisation. Plots derived from both the optimisation of the JMVIB−T objective (Eq 8) and the optimisation of the JMVIB objective (Eq 5) are included. Five copies of all plots are available, as they are obtained by training the model with five different independent training-test random splits. (ZIP) [file pcbi.1010050.s007.zip › s5-file/bce-and-triplet/J/T2D/1_embeddings.png]

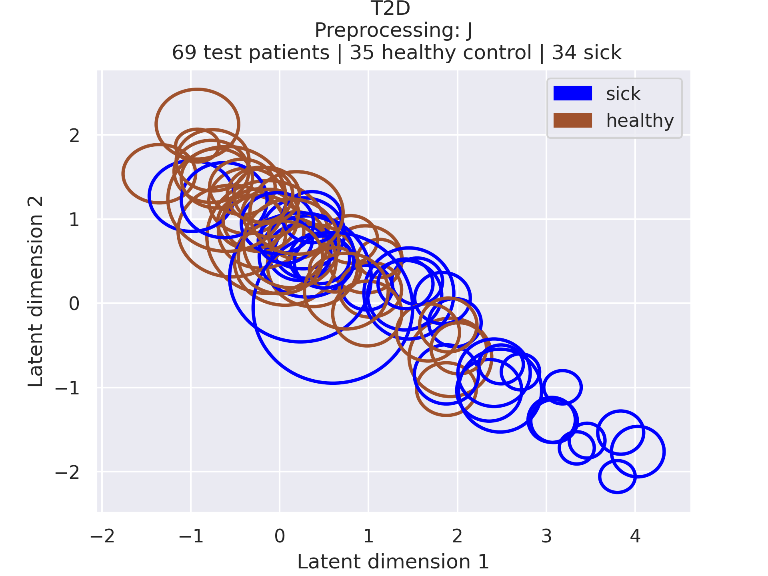

Supplement: S2 File — For all datasets considered in this work, this file presents plots of the 2D MVIB stochastic encodings analogous to Fig 3. The depicted curves are the 95% confidence intervals of the samples’ stochastic encodings z∼p(z|x)=N(μ,σ2I); the points are their means μ. The displayed encodings consist only in the test samples obtained from random training-test splits (i.e. the 20% of the dataset not used for training). The K dimension of the latent space has been set to 2 in order to allow a 2D visualisation. Plots derived from both the optimisation of the JMVIB−T objective (Eq 8) and the optimisation of the JMVIB objective (Eq 5) are included. Five copies of all plots are available, as they are obtained by training the model with five different independent training-test random splits. (ZIP) [file pcbi.1010050.s007.zip › s5-file/bce-and-triplet/J/T2D/1_embeddings_95_confidence.png]

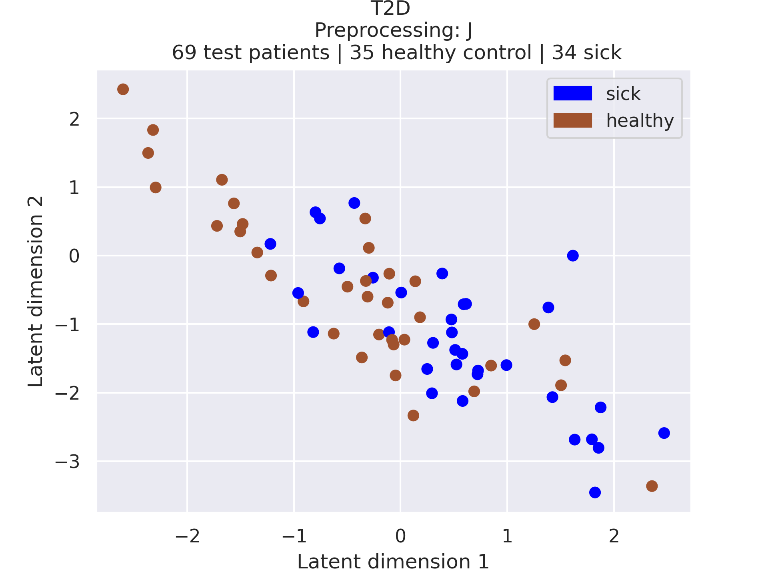

Supplement: S2 File — For all datasets considered in this work, this file presents plots of the 2D MVIB stochastic encodings analogous to Fig 3. The depicted curves are the 95% confidence intervals of the samples’ stochastic encodings z∼p(z|x)=N(μ,σ2I); the points are their means μ. The displayed encodings consist only in the test samples obtained from random training-test splits (i.e. the 20% of the dataset not used for training). The K dimension of the latent space has been set to 2 in order to allow a 2D visualisation. Plots derived from both the optimisation of the JMVIB−T objective (Eq 8) and the optimisation of the JMVIB objective (Eq 5) are included. Five copies of all plots are available, as they are obtained by training the model with five different independent training-test random splits. (ZIP) [file pcbi.1010050.s007.zip › s5-file/bce-and-triplet/J/T2D/2_embeddings.png]

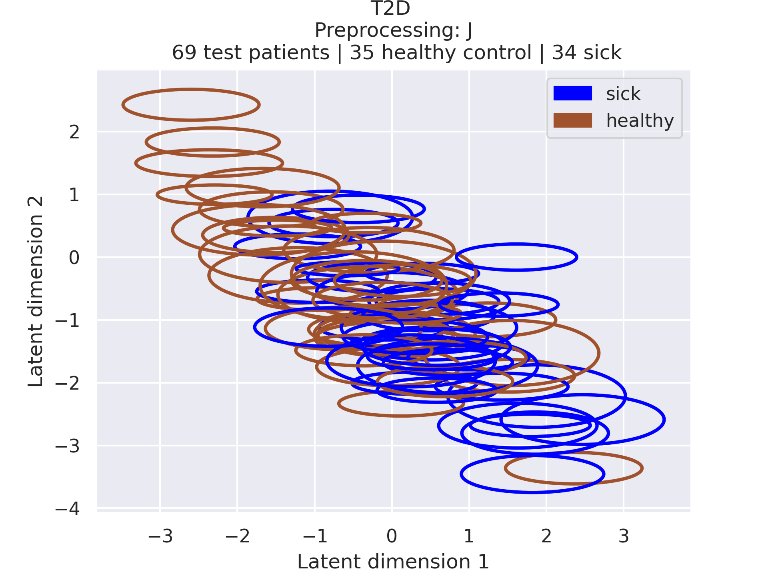

Supplement: S2 File — For all datasets considered in this work, this file presents plots of the 2D MVIB stochastic encodings analogous to Fig 3. The depicted curves are the 95% confidence intervals of the samples’ stochastic encodings z∼p(z|x)=N(μ,σ2I); the points are their means μ. The displayed encodings consist only in the test samples obtained from random training-test splits (i.e. the 20% of the dataset not used for training). The K dimension of the latent space has been set to 2 in order to allow a 2D visualisation. Plots derived from both the optimisation of the JMVIB−T objective (Eq 8) and the optimisation of the JMVIB objective (Eq 5) are included. Five copies of all plots are available, as they are obtained by training the model with five different independent training-test random splits. (ZIP) [file pcbi.1010050.s007.zip › s5-file/bce-and-triplet/J/T2D/2_embeddings_95_confidence.png]

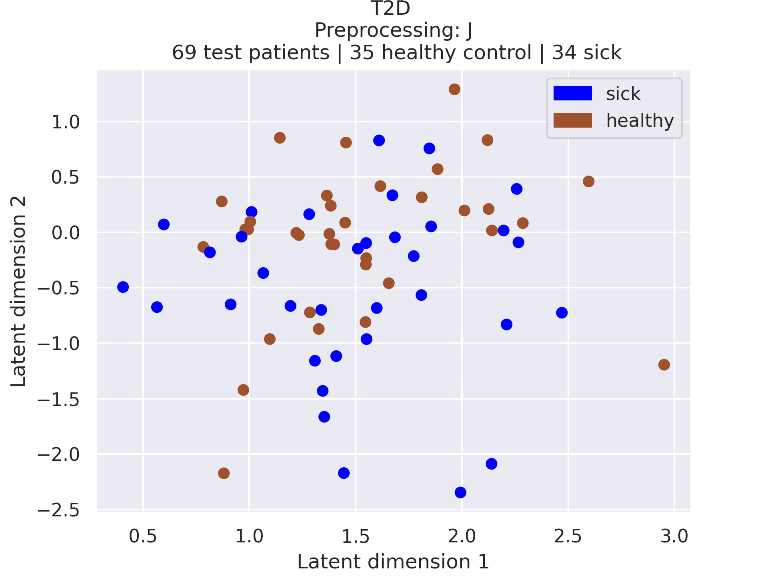

Supplement: S2 File — For all datasets considered in this work, this file presents plots of the 2D MVIB stochastic encodings analogous to Fig 3. The depicted curves are the 95% confidence intervals of the samples’ stochastic encodings z∼p(z|x)=N(μ,σ2I); the points are their means μ. The displayed encodings consist only in the test samples obtained from random training-test splits (i.e. the 20% of the dataset not used for training). The K dimension of the latent space has been set to 2 in order to allow a 2D visualisation. Plots derived from both the optimisation of the JMVIB−T objective (Eq 8) and the optimisation of the JMVIB objective (Eq 5) are included. Five copies of all plots are available, as they are obtained by training the model with five different independent training-test random splits. (ZIP) [file pcbi.1010050.s007.zip › s5-file/bce-and-triplet/J/T2D/3_embeddings.png]

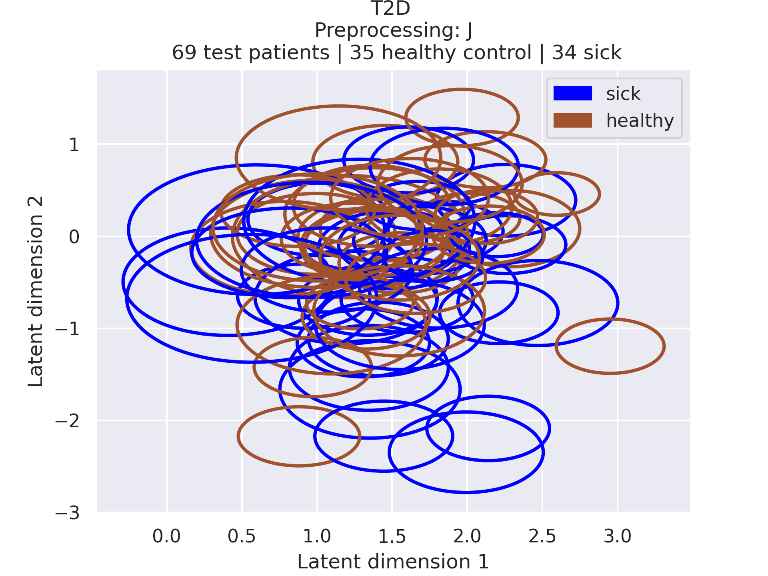

Supplement: S2 File — For all datasets considered in this work, this file presents plots of the 2D MVIB stochastic encodings analogous to Fig 3. The depicted curves are the 95% confidence intervals of the samples’ stochastic encodings z∼p(z|x)=N(μ,σ2I); the points are their means μ. The displayed encodings consist only in the test samples obtained from random training-test splits (i.e. the 20% of the dataset not used for training). The K dimension of the latent space has been set to 2 in order to allow a 2D visualisation. Plots derived from both the optimisation of the JMVIB−T objective (Eq 8) and the optimisation of the JMVIB objective (Eq 5) are included. Five copies of all plots are available, as they are obtained by training the model with five different independent training-test random splits. (ZIP) [file pcbi.1010050.s007.zip › s5-file/bce-and-triplet/J/T2D/3_embeddings_95_confidence.png]

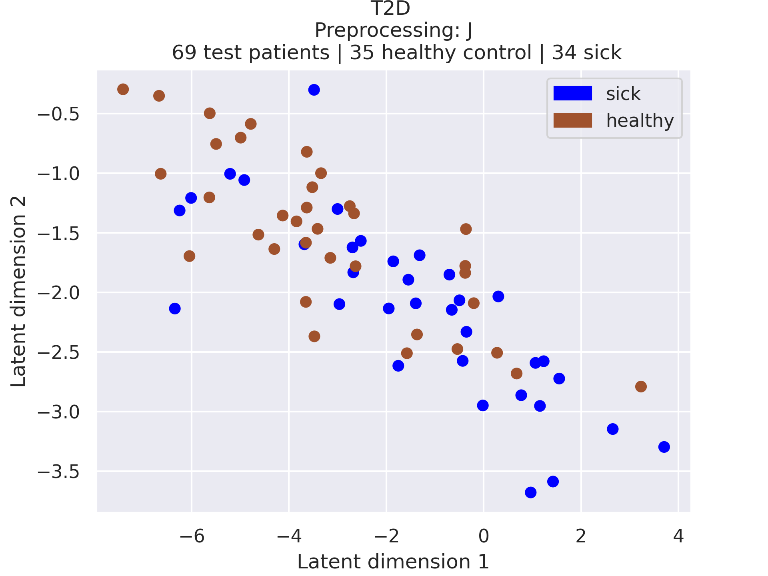

Supplement: S2 File — For all datasets considered in this work, this file presents plots of the 2D MVIB stochastic encodings analogous to Fig 3. The depicted curves are the 95% confidence intervals of the samples’ stochastic encodings z∼p(z|x)=N(μ,σ2I); the points are their means μ. The displayed encodings consist only in the test samples obtained from random training-test splits (i.e. the 20% of the dataset not used for training). The K dimension of the latent space has been set to 2 in order to allow a 2D visualisation. Plots derived from both the optimisation of the JMVIB−T objective (Eq 8) and the optimisation of the JMVIB objective (Eq 5) are included. Five copies of all plots are available, as they are obtained by training the model with five different independent training-test random splits. (ZIP) [file pcbi.1010050.s007.zip › s5-file/bce-and-triplet/J/T2D/4_embeddings.png]

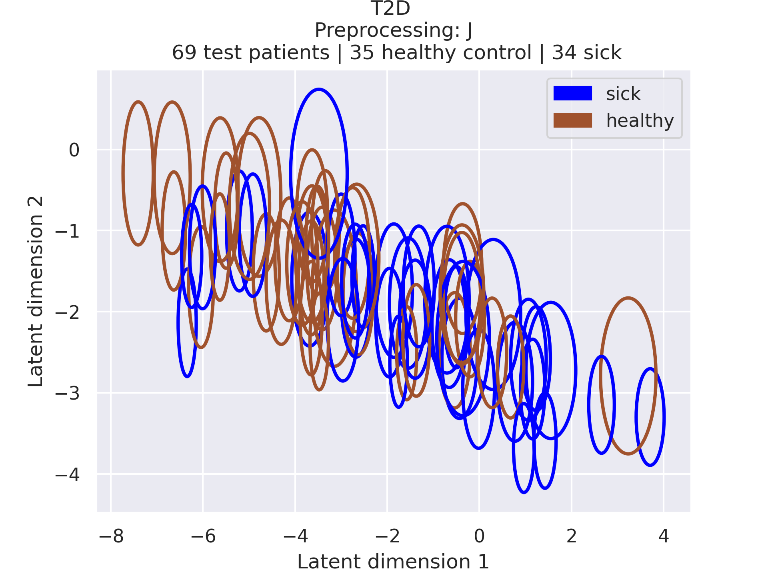

Supplement: S2 File — For all datasets considered in this work, this file presents plots of the 2D MVIB stochastic encodings analogous to Fig 3. The depicted curves are the 95% confidence intervals of the samples’ stochastic encodings z∼p(z|x)=N(μ,σ2I); the points are their means μ. The displayed encodings consist only in the test samples obtained from random training-test splits (i.e. the 20% of the dataset not used for training). The K dimension of the latent space has been set to 2 in order to allow a 2D visualisation. Plots derived from both the optimisation of the JMVIB−T objective (Eq 8) and the optimisation of the JMVIB objective (Eq 5) are included. Five copies of all plots are available, as they are obtained by training the model with five different independent training-test random splits. (ZIP) [file pcbi.1010050.s007.zip › s5-file/bce-and-triplet/J/T2D/4_embeddings_95_confidence.png]

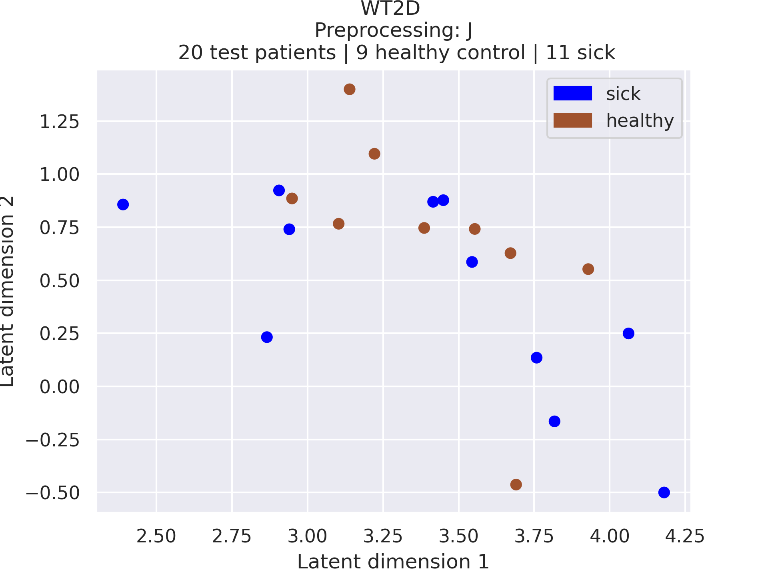

Supplement: S2 File — For all datasets considered in this work, this file presents plots of the 2D MVIB stochastic encodings analogous to Fig 3. The depicted curves are the 95% confidence intervals of the samples’ stochastic encodings z∼p(z|x)=N(μ,σ2I); the points are their means μ. The displayed encodings consist only in the test samples obtained from random training-test splits (i.e. the 20% of the dataset not used for training). The K dimension of the latent space has been set to 2 in order to allow a 2D visualisation. Plots derived from both the optimisation of the JMVIB−T objective (Eq 8) and the optimisation of the JMVIB objective (Eq 5) are included. Five copies of all plots are available, as they are obtained by training the model with five different independent training-test random splits. (ZIP) [file pcbi.1010050.s007.zip › s5-file/bce-and-triplet/J/WT2D/0_embeddings.png]

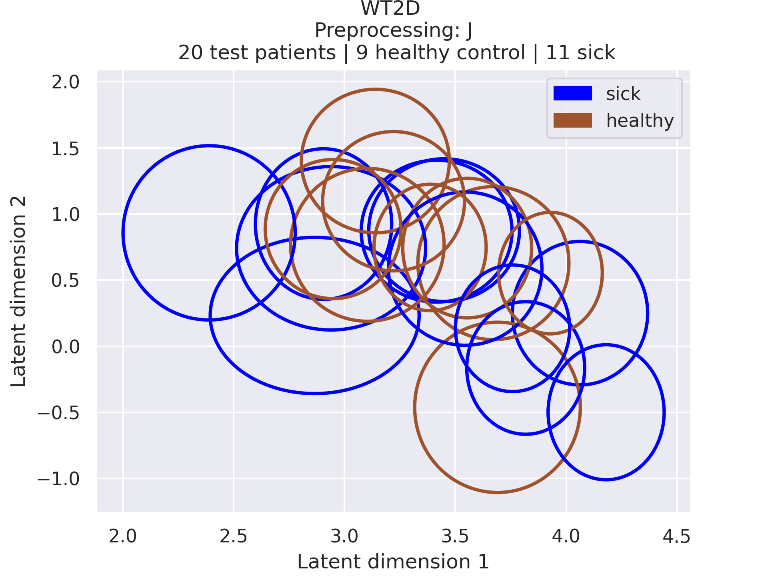

Supplement: S2 File — For all datasets considered in this work, this file presents plots of the 2D MVIB stochastic encodings analogous to Fig 3. The depicted curves are the 95% confidence intervals of the samples’ stochastic encodings z∼p(z|x)=N(μ,σ2I); the points are their means μ. The displayed encodings consist only in the test samples obtained from random training-test splits (i.e. the 20% of the dataset not used for training). The K dimension of the latent space has been set to 2 in order to allow a 2D visualisation. Plots derived from both the optimisation of the JMVIB−T objective (Eq 8) and the optimisation of the JMVIB objective (Eq 5) are included. Five copies of all plots are available, as they are obtained by training the model with five different independent training-test random splits. (ZIP) [file pcbi.1010050.s007.zip › s5-file/bce-and-triplet/J/WT2D/0_embeddings_95_confidence.png]

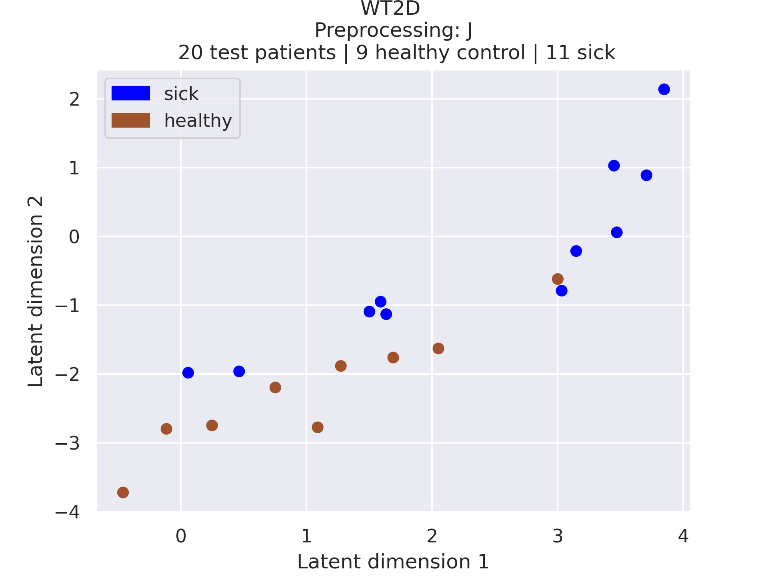

Supplement: S2 File — For all datasets considered in this work, this file presents plots of the 2D MVIB stochastic encodings analogous to Fig 3. The depicted curves are the 95% confidence intervals of the samples’ stochastic encodings z∼p(z|x)=N(μ,σ2I); the points are their means μ. The displayed encodings consist only in the test samples obtained from random training-test splits (i.e. the 20% of the dataset not used for training). The K dimension of the latent space has been set to 2 in order to allow a 2D visualisation. Plots derived from both the optimisation of the JMVIB−T objective (Eq 8) and the optimisation of the JMVIB objective (Eq 5) are included. Five copies of all plots are available, as they are obtained by training the model with five different independent training-test random splits. (ZIP) [file pcbi.1010050.s007.zip › s5-file/bce-and-triplet/J/WT2D/1_embeddings.png]

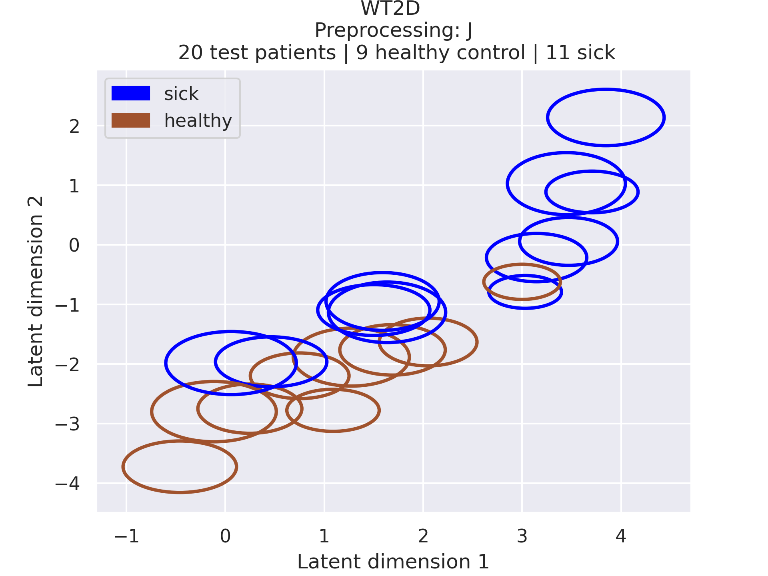

Supplement: S2 File — For all datasets considered in this work, this file presents plots of the 2D MVIB stochastic encodings analogous to Fig 3. The depicted curves are the 95% confidence intervals of the samples’ stochastic encodings z∼p(z|x)=N(μ,σ2I); the points are their means μ. The displayed encodings consist only in the test samples obtained from random training-test splits (i.e. the 20% of the dataset not used for training). The K dimension of the latent space has been set to 2 in order to allow a 2D visualisation. Plots derived from both the optimisation of the JMVIB−T objective (Eq 8) and the optimisation of the JMVIB objective (Eq 5) are included. Five copies of all plots are available, as they are obtained by training the model with five different independent training-test random splits. (ZIP) [file pcbi.1010050.s007.zip › s5-file/bce-and-triplet/J/WT2D/1_embeddings_95_confidence.png]

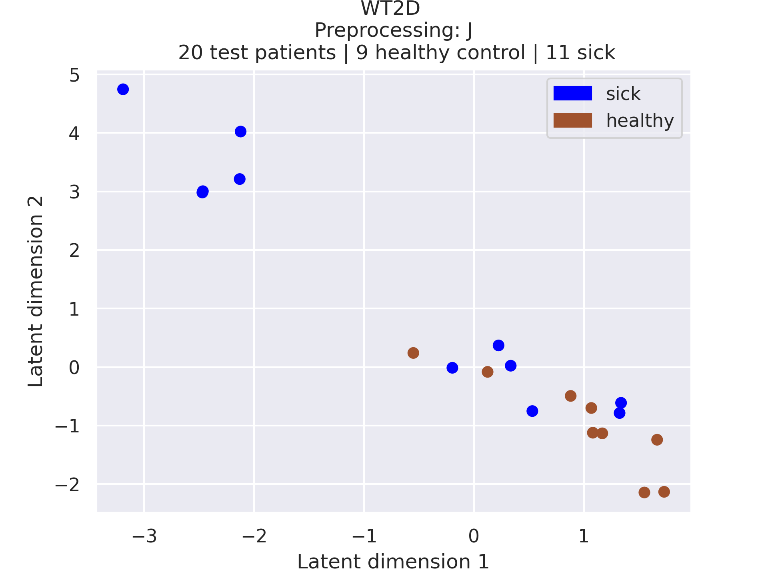

Supplement: S2 File — For all datasets considered in this work, this file presents plots of the 2D MVIB stochastic encodings analogous to Fig 3. The depicted curves are the 95% confidence intervals of the samples’ stochastic encodings z∼p(z|x)=N(μ,σ2I); the points are their means μ. The displayed encodings consist only in the test samples obtained from random training-test splits (i.e. the 20% of the dataset not used for training). The K dimension of the latent space has been set to 2 in order to allow a 2D visualisation. Plots derived from both the optimisation of the JMVIB−T objective (Eq 8) and the optimisation of the JMVIB objective (Eq 5) are included. Five copies of all plots are available, as they are obtained by training the model with five different independent training-test random splits. (ZIP) [file pcbi.1010050.s007.zip › s5-file/bce-and-triplet/J/WT2D/2_embeddings.png]

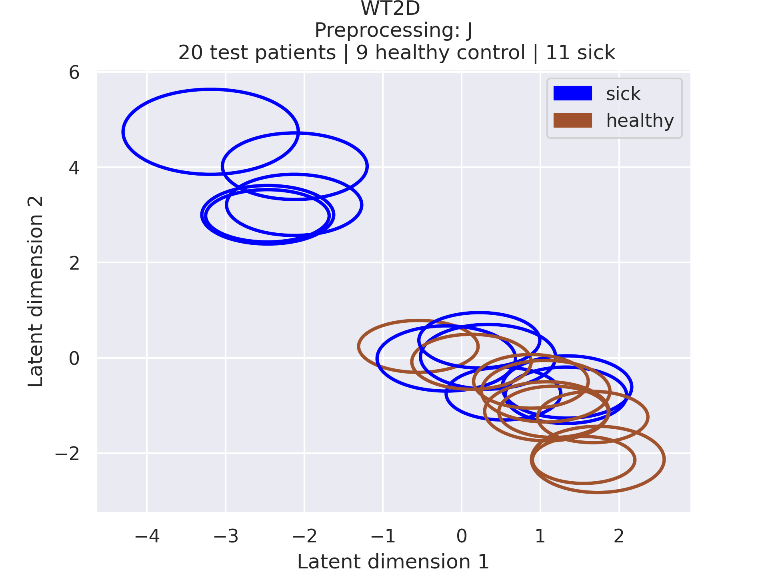

Supplement: S2 File — For all datasets considered in this work, this file presents plots of the 2D MVIB stochastic encodings analogous to Fig 3. The depicted curves are the 95% confidence intervals of the samples’ stochastic encodings z∼p(z|x)=N(μ,σ2I); the points are their means μ. The displayed encodings consist only in the test samples obtained from random training-test splits (i.e. the 20% of the dataset not used for training). The K dimension of the latent space has been set to 2 in order to allow a 2D visualisation. Plots derived from both the optimisation of the JMVIB−T objective (Eq 8) and the optimisation of the JMVIB objective (Eq 5) are included. Five copies of all plots are available, as they are obtained by training the model with five different independent training-test random splits. (ZIP) [file pcbi.1010050.s007.zip › s5-file/bce-and-triplet/J/WT2D/2_embeddings_95_confidence.png]

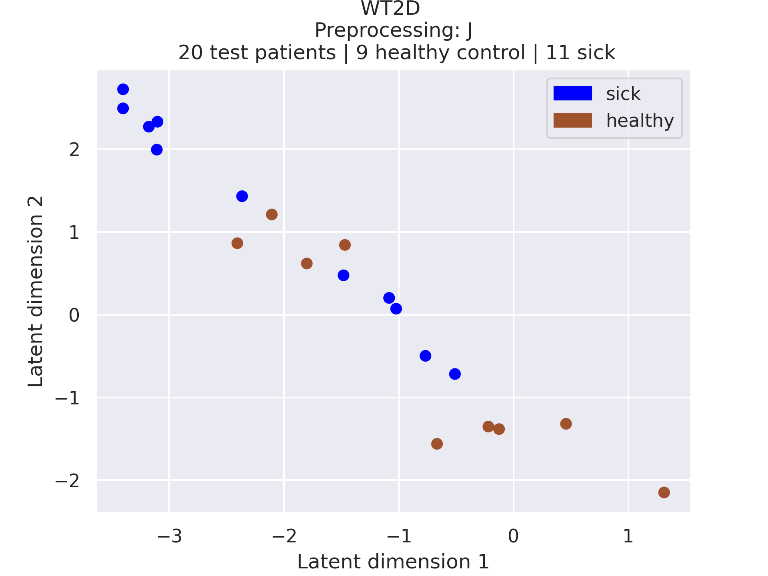

Supplement: S2 File — For all datasets considered in this work, this file presents plots of the 2D MVIB stochastic encodings analogous to Fig 3. The depicted curves are the 95% confidence intervals of the samples’ stochastic encodings z∼p(z|x)=N(μ,σ2I); the points are their means μ. The displayed encodings consist only in the test samples obtained from random training-test splits (i.e. the 20% of the dataset not used for training). The K dimension of the latent space has been set to 2 in order to allow a 2D visualisation. Plots derived from both the optimisation of the JMVIB−T objective (Eq 8) and the optimisation of the JMVIB objective (Eq 5) are included. Five copies of all plots are available, as they are obtained by training the model with five different independent training-test random splits. (ZIP) [file pcbi.1010050.s007.zip › s5-file/bce-and-triplet/J/WT2D/3_embeddings.png]

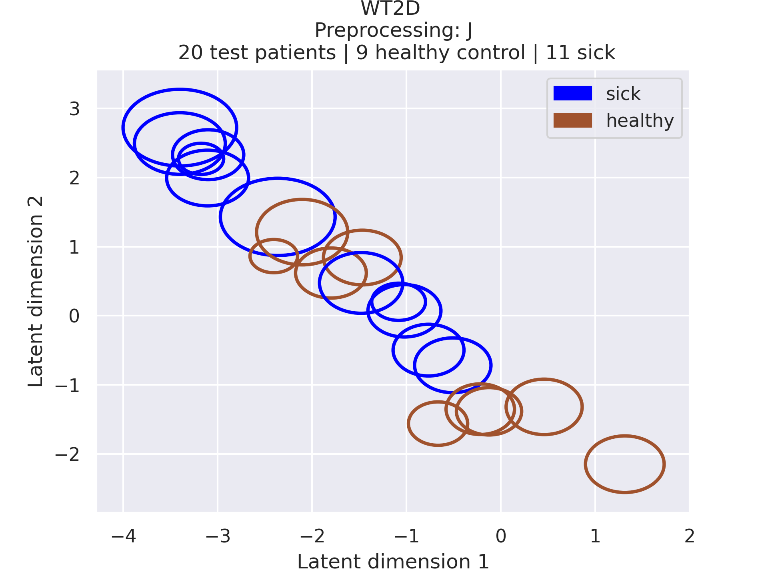

Supplement: S2 File — For all datasets considered in this work, this file presents plots of the 2D MVIB stochastic encodings analogous to Fig 3. The depicted curves are the 95% confidence intervals of the samples’ stochastic encodings z∼p(z|x)=N(μ,σ2I); the points are their means μ. The displayed encodings consist only in the test samples obtained from random training-test splits (i.e. the 20% of the dataset not used for training). The K dimension of the latent space has been set to 2 in order to allow a 2D visualisation. Plots derived from both the optimisation of the JMVIB−T objective (Eq 8) and the optimisation of the JMVIB objective (Eq 5) are included. Five copies of all plots are available, as they are obtained by training the model with five different independent training-test random splits. (ZIP) [file pcbi.1010050.s007.zip › s5-file/bce-and-triplet/J/WT2D/3_embeddings_95_confidence.png]

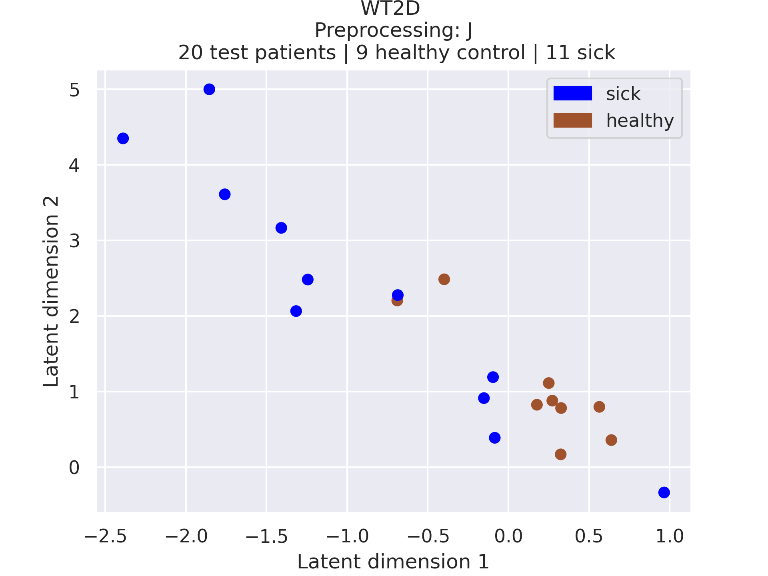

Supplement: S2 File — For all datasets considered in this work, this file presents plots of the 2D MVIB stochastic encodings analogous to Fig 3. The depicted curves are the 95% confidence intervals of the samples’ stochastic encodings z∼p(z|x)=N(μ,σ2I); the points are their means μ. The displayed encodings consist only in the test samples obtained from random training-test splits (i.e. the 20% of the dataset not used for training). The K dimension of the latent space has been set to 2 in order to allow a 2D visualisation. Plots derived from both the optimisation of the JMVIB−T objective (Eq 8) and the optimisation of the JMVIB objective (Eq 5) are included. Five copies of all plots are available, as they are obtained by training the model with five different independent training-test random splits. (ZIP) [file pcbi.1010050.s007.zip › s5-file/bce-and-triplet/J/WT2D/4_embeddings.png]

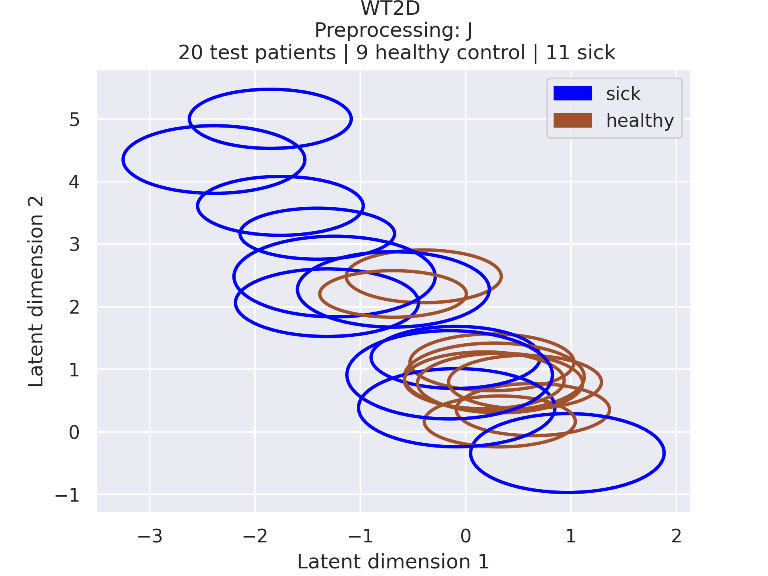

Supplement: S2 File — For all datasets considered in this work, this file presents plots of the 2D MVIB stochastic encodings analogous to Fig 3. The depicted curves are the 95% confidence intervals of the samples’ stochastic encodings z∼p(z|x)=N(μ,σ2I); the points are their means μ. The displayed encodings consist only in the test samples obtained from random training-test splits (i.e. the 20% of the dataset not used for training). The K dimension of the latent space has been set to 2 in order to allow a 2D visualisation. Plots derived from both the optimisation of the JMVIB−T objective (Eq 8) and the optimisation of the JMVIB objective (Eq 5) are included. Five copies of all plots are available, as they are obtained by training the model with five different independent training-test random splits. (ZIP) [file pcbi.1010050.s007.zip › s5-file/bce-and-triplet/J/WT2D/4_embeddings_95_confidence.png]

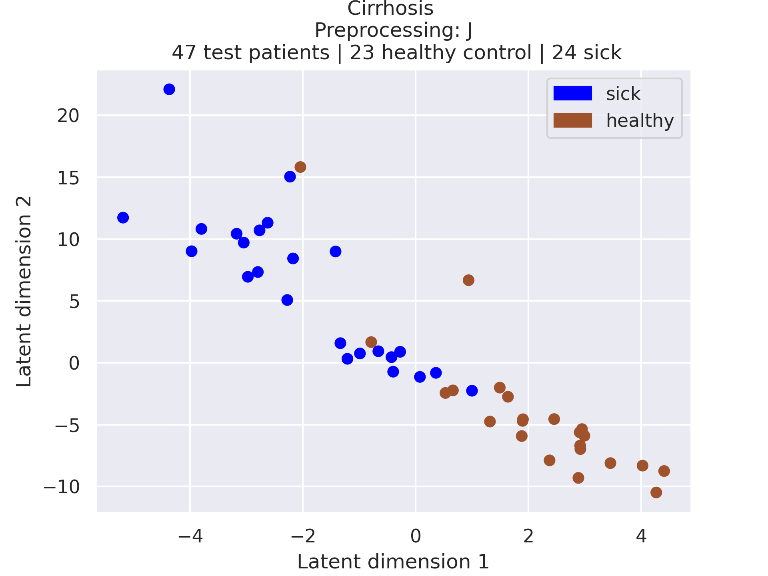

Supplement: S2 File — For all datasets considered in this work, this file presents plots of the 2D MVIB stochastic encodings analogous to Fig 3. The depicted curves are the 95% confidence intervals of the samples’ stochastic encodings z∼p(z|x)=N(μ,σ2I); the points are their means μ. The displayed encodings consist only in the test samples obtained from random training-test splits (i.e. the 20% of the dataset not used for training). The K dimension of the latent space has been set to 2 in order to allow a 2D visualisation. Plots derived from both the optimisation of the JMVIB−T objective (Eq 8) and the optimisation of the JMVIB objective (Eq 5) are included. Five copies of all plots are available, as they are obtained by training the model with five different independent training-test random splits. (ZIP) [file pcbi.1010050.s007.zip › s5-file/bce-only/J/Cirrhosis/0_embeddings.png]

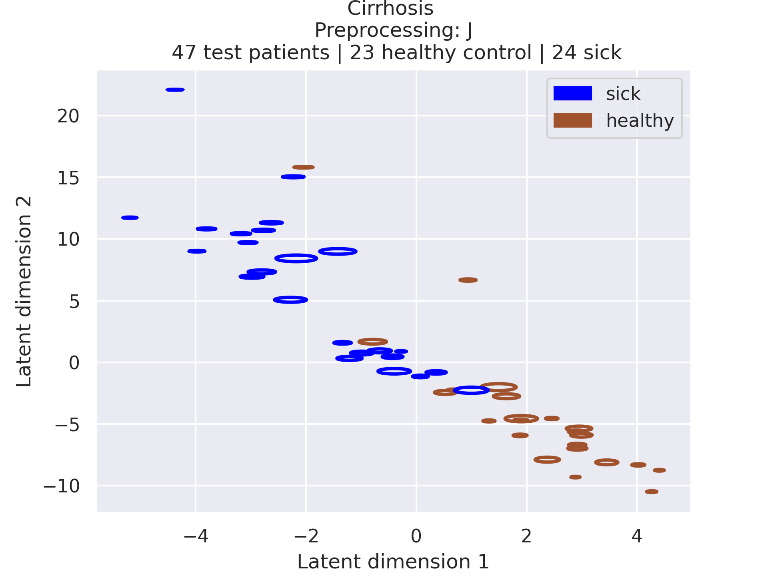

Supplement: S2 File — For all datasets considered in this work, this file presents plots of the 2D MVIB stochastic encodings analogous to Fig 3. The depicted curves are the 95% confidence intervals of the samples’ stochastic encodings z∼p(z|x)=N(μ,σ2I); the points are their means μ. The displayed encodings consist only in the test samples obtained from random training-test splits (i.e. the 20% of the dataset not used for training). The K dimension of the latent space has been set to 2 in order to allow a 2D visualisation. Plots derived from both the optimisation of the JMVIB−T objective (Eq 8) and the optimisation of the JMVIB objective (Eq 5) are included. Five copies of all plots are available, as they are obtained by training the model with five different independent training-test random splits. (ZIP) [file pcbi.1010050.s007.zip › s5-file/bce-only/J/Cirrhosis/0_embeddings_95_confidence.png]

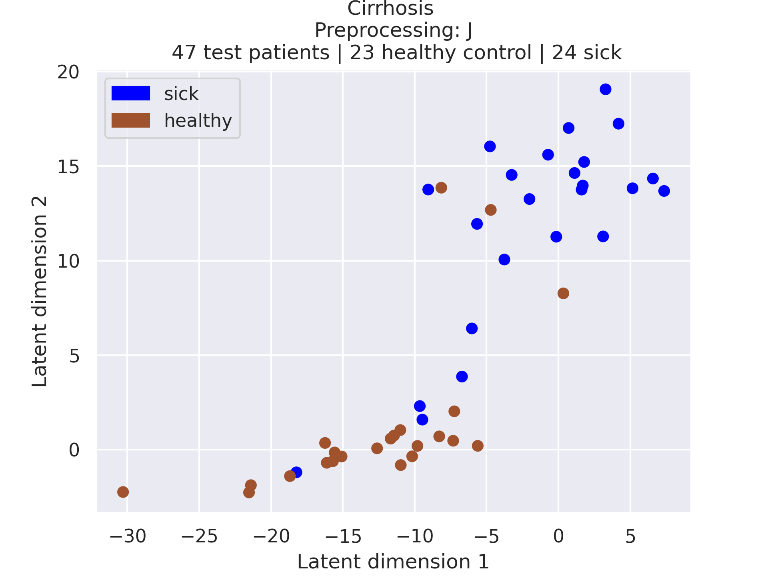

Supplement: S2 File — For all datasets considered in this work, this file presents plots of the 2D MVIB stochastic encodings analogous to Fig 3. The depicted curves are the 95% confidence intervals of the samples’ stochastic encodings z∼p(z|x)=N(μ,σ2I); the points are their means μ. The displayed encodings consist only in the test samples obtained from random training-test splits (i.e. the 20% of the dataset not used for training). The K dimension of the latent space has been set to 2 in order to allow a 2D visualisation. Plots derived from both the optimisation of the JMVIB−T objective (Eq 8) and the optimisation of the JMVIB objective (Eq 5) are included. Five copies of all plots are available, as they are obtained by training the model with five different independent training-test random splits. (ZIP) [file pcbi.1010050.s007.zip › s5-file/bce-only/J/Cirrhosis/1_embeddings.png]

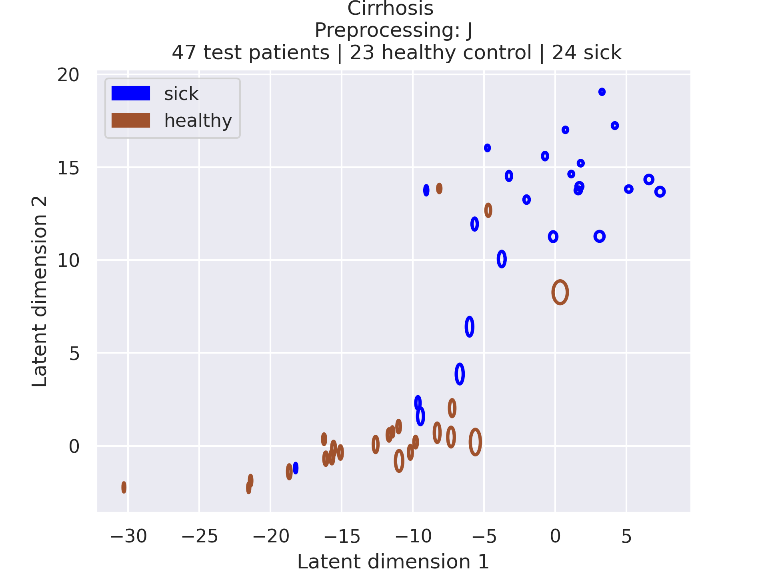

Supplement: S2 File — For all datasets considered in this work, this file presents plots of the 2D MVIB stochastic encodings analogous to Fig 3. The depicted curves are the 95% confidence intervals of the samples’ stochastic encodings z∼p(z|x)=N(μ,σ2I); the points are their means μ. The displayed encodings consist only in the test samples obtained from random training-test splits (i.e. the 20% of the dataset not used for training). The K dimension of the latent space has been set to 2 in order to allow a 2D visualisation. Plots derived from both the optimisation of the JMVIB−T objective (Eq 8) and the optimisation of the JMVIB objective (Eq 5) are included. Five copies of all plots are available, as they are obtained by training the model with five different independent training-test random splits. (ZIP) [file pcbi.1010050.s007.zip › s5-file/bce-only/J/Cirrhosis/1_embeddings_95_confidence.png]

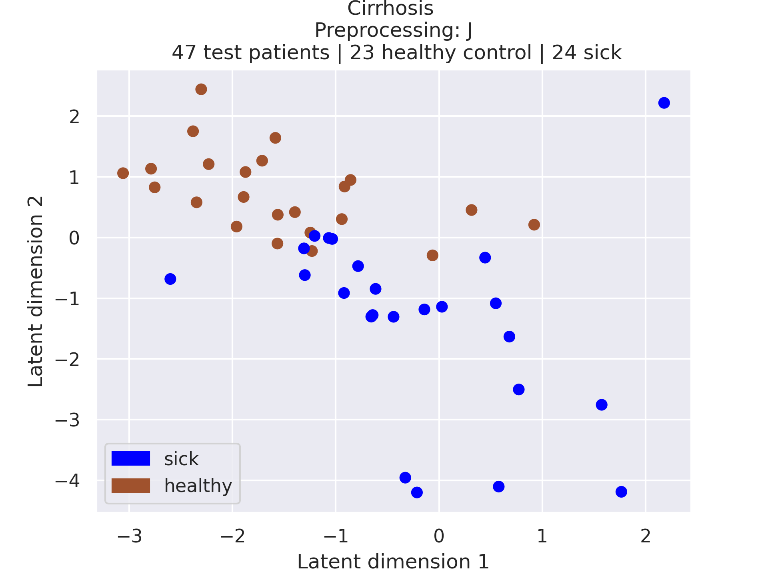

Supplement: S2 File — For all datasets considered in this work, this file presents plots of the 2D MVIB stochastic encodings analogous to Fig 3. The depicted curves are the 95% confidence intervals of the samples’ stochastic encodings z∼p(z|x)=N(μ,σ2I); the points are their means μ. The displayed encodings consist only in the test samples obtained from random training-test splits (i.e. the 20% of the dataset not used for training). The K dimension of the latent space has been set to 2 in order to allow a 2D visualisation. Plots derived from both the optimisation of the JMVIB−T objective (Eq 8) and the optimisation of the JMVIB objective (Eq 5) are included. Five copies of all plots are available, as they are obtained by training the model with five different independent training-test random splits. (ZIP) [file pcbi.1010050.s007.zip › s5-file/bce-only/J/Cirrhosis/2_embeddings.png]

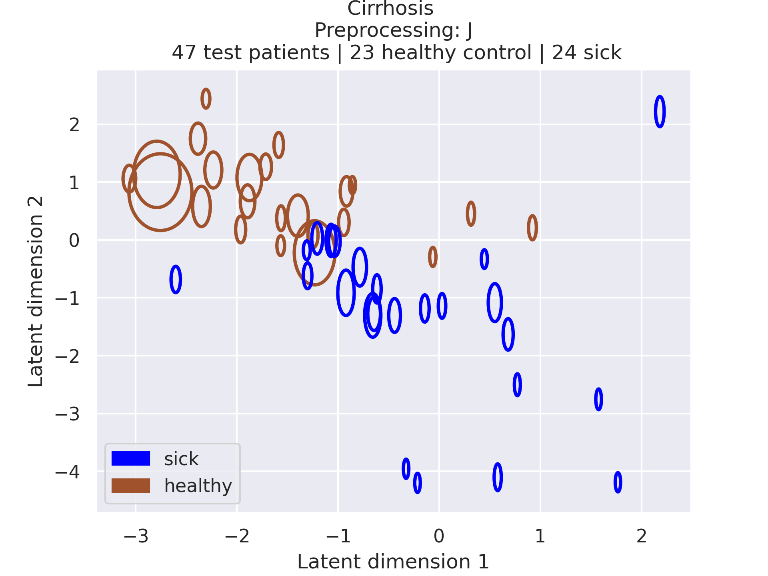

Supplement: S2 File — For all datasets considered in this work, this file presents plots of the 2D MVIB stochastic encodings analogous to Fig 3. The depicted curves are the 95% confidence intervals of the samples’ stochastic encodings z∼p(z|x)=N(μ,σ2I); the points are their means μ. The displayed encodings consist only in the test samples obtained from random training-test splits (i.e. the 20% of the dataset not used for training). The K dimension of the latent space has been set to 2 in order to allow a 2D visualisation. Plots derived from both the optimisation of the JMVIB−T objective (Eq 8) and the optimisation of the JMVIB objective (Eq 5) are included. Five copies of all plots are available, as they are obtained by training the model with five different independent training-test random splits. (ZIP) [file pcbi.1010050.s007.zip › s5-file/bce-only/J/Cirrhosis/2_embeddings_95_confidence.png]

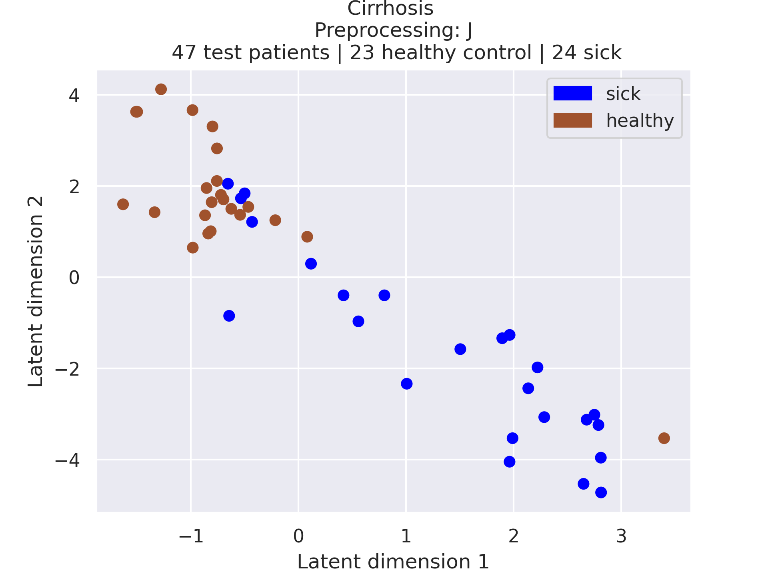

Supplement: S2 File — For all datasets considered in this work, this file presents plots of the 2D MVIB stochastic encodings analogous to Fig 3. The depicted curves are the 95% confidence intervals of the samples’ stochastic encodings z∼p(z|x)=N(μ,σ2I); the points are their means μ. The displayed encodings consist only in the test samples obtained from random training-test splits (i.e. the 20% of the dataset not used for training). The K dimension of the latent space has been set to 2 in order to allow a 2D visualisation. Plots derived from both the optimisation of the JMVIB−T objective (Eq 8) and the optimisation of the JMVIB objective (Eq 5) are included. Five copies of all plots are available, as they are obtained by training the model with five different independent training-test random splits. (ZIP) [file pcbi.1010050.s007.zip › s5-file/bce-only/J/Cirrhosis/3_embeddings.png]

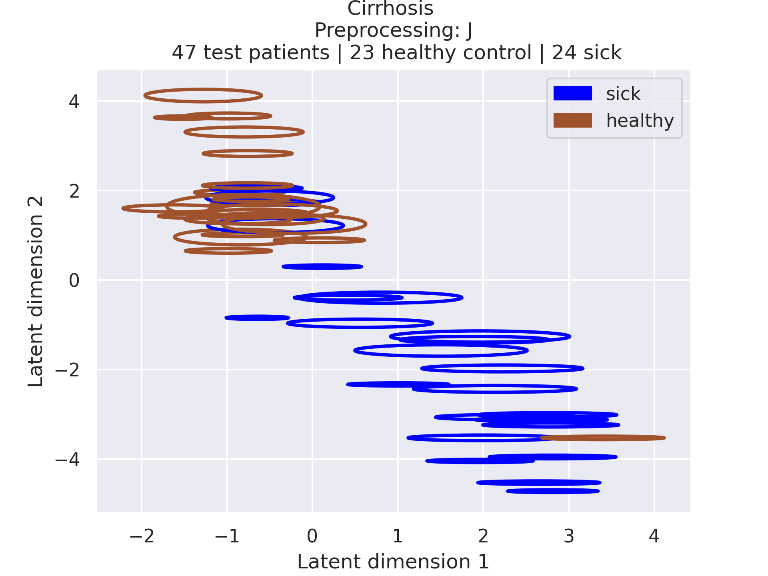

Supplement: S2 File — For all datasets considered in this work, this file presents plots of the 2D MVIB stochastic encodings analogous to Fig 3. The depicted curves are the 95% confidence intervals of the samples’ stochastic encodings z∼p(z|x)=N(μ,σ2I); the points are their means μ. The displayed encodings consist only in the test samples obtained from random training-test splits (i.e. the 20% of the dataset not used for training). The K dimension of the latent space has been set to 2 in order to allow a 2D visualisation. Plots derived from both the optimisation of the JMVIB−T objective (Eq 8) and the optimisation of the JMVIB objective (Eq 5) are included. Five copies of all plots are available, as they are obtained by training the model with five different independent training-test random splits. (ZIP) [file pcbi.1010050.s007.zip › s5-file/bce-only/J/Cirrhosis/3_embeddings_95_confidence.png]

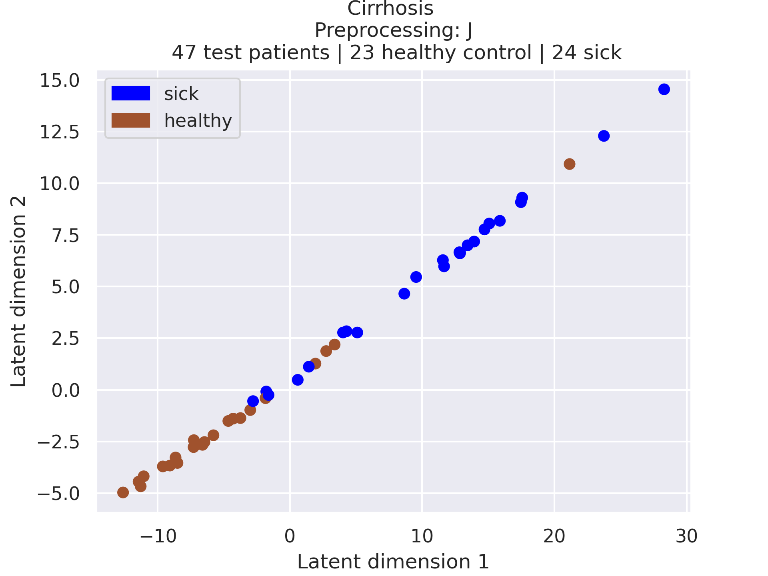

Supplement: S2 File — For all datasets considered in this work, this file presents plots of the 2D MVIB stochastic encodings analogous to Fig 3. The depicted curves are the 95% confidence intervals of the samples’ stochastic encodings z∼p(z|x)=N(μ,σ2I); the points are their means μ. The displayed encodings consist only in the test samples obtained from random training-test splits (i.e. the 20% of the dataset not used for training). The K dimension of the latent space has been set to 2 in order to allow a 2D visualisation. Plots derived from both the optimisation of the JMVIB−T objective (Eq 8) and the optimisation of the JMVIB objective (Eq 5) are included. Five copies of all plots are available, as they are obtained by training the model with five different independent training-test random splits. (ZIP) [file pcbi.1010050.s007.zip › s5-file/bce-only/J/Cirrhosis/4_embeddings.png]

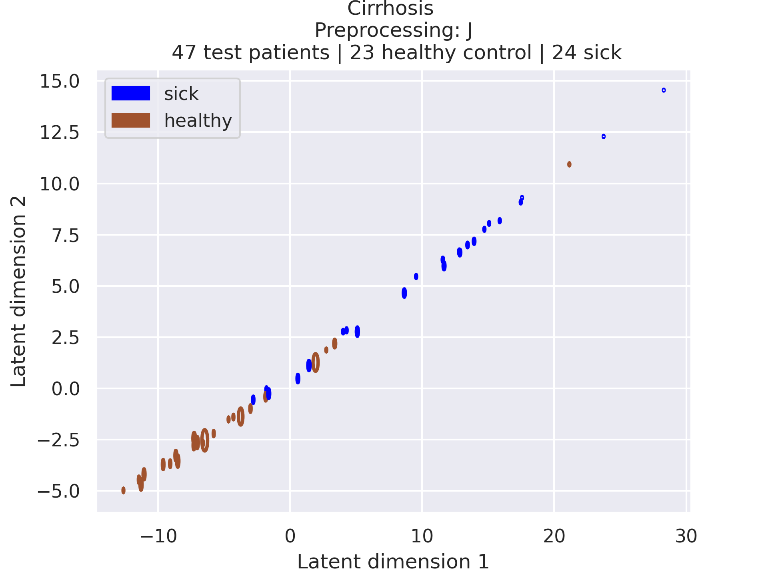

Supplement: S2 File — For all datasets considered in this work, this file presents plots of the 2D MVIB stochastic encodings analogous to Fig 3. The depicted curves are the 95% confidence intervals of the samples’ stochastic encodings z∼p(z|x)=N(μ,σ2I); the points are their means μ. The displayed encodings consist only in the test samples obtained from random training-test splits (i.e. the 20% of the dataset not used for training). The K dimension of the latent space has been set to 2 in order to allow a 2D visualisation. Plots derived from both the optimisation of the JMVIB−T objective (Eq 8) and the optimisation of the JMVIB objective (Eq 5) are included. Five copies of all plots are available, as they are obtained by training the model with five different independent training-test random splits. (ZIP) [file pcbi.1010050.s007.zip › s5-file/bce-only/J/Cirrhosis/4_embeddings_95_confidence.png]

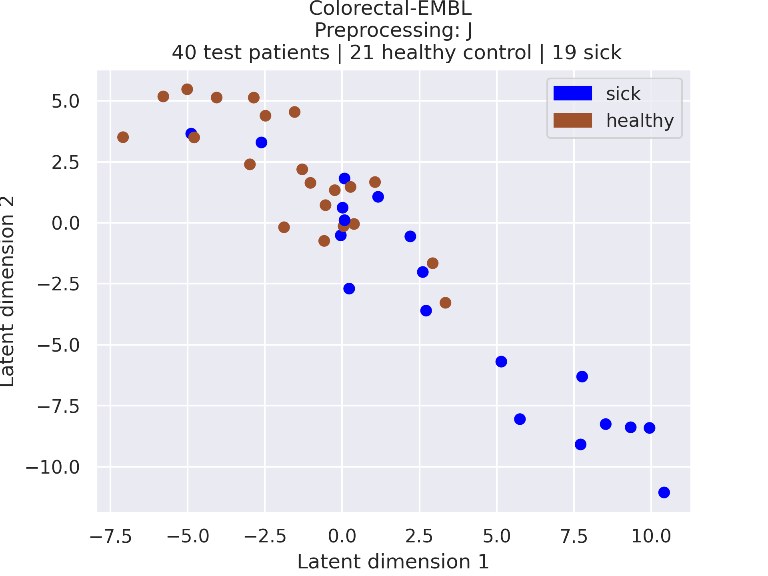

Supplement: S2 File — For all datasets considered in this work, this file presents plots of the 2D MVIB stochastic encodings analogous to Fig 3. The depicted curves are the 95% confidence intervals of the samples’ stochastic encodings z∼p(z|x)=N(μ,σ2I); the points are their means μ. The displayed encodings consist only in the test samples obtained from random training-test splits (i.e. the 20% of the dataset not used for training). The K dimension of the latent space has been set to 2 in order to allow a 2D visualisation. Plots derived from both the optimisation of the JMVIB−T objective (Eq 8) and the optimisation of the JMVIB objective (Eq 5) are included. Five copies of all plots are available, as they are obtained by training the model with five different independent training-test random splits. (ZIP) [file pcbi.1010050.s007.zip › s5-file/bce-only/J/Colorectal-EMBL/0_embeddings.png]

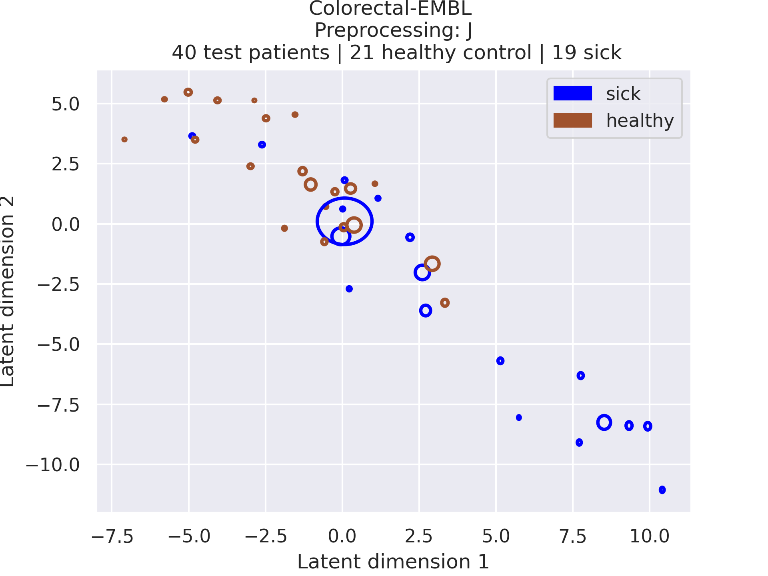

Supplement: S2 File — For all datasets considered in this work, this file presents plots of the 2D MVIB stochastic encodings analogous to Fig 3. The depicted curves are the 95% confidence intervals of the samples’ stochastic encodings z∼p(z|x)=N(μ,σ2I); the points are their means μ. The displayed encodings consist only in the test samples obtained from random training-test splits (i.e. the 20% of the dataset not used for training). The K dimension of the latent space has been set to 2 in order to allow a 2D visualisation. Plots derived from both the optimisation of the JMVIB−T objective (Eq 8) and the optimisation of the JMVIB objective (Eq 5) are included. Five copies of all plots are available, as they are obtained by training the model with five different independent training-test random splits. (ZIP) [file pcbi.1010050.s007.zip › s5-file/bce-only/J/Colorectal-EMBL/0_embeddings_95_confidence.png]

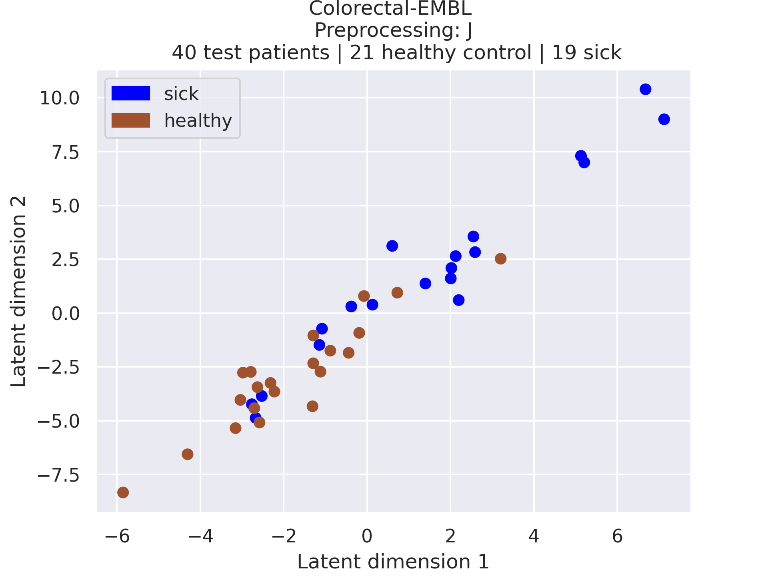

Supplement: S2 File — For all datasets considered in this work, this file presents plots of the 2D MVIB stochastic encodings analogous to Fig 3. The depicted curves are the 95% confidence intervals of the samples’ stochastic encodings z∼p(z|x)=N(μ,σ2I); the points are their means μ. The displayed encodings consist only in the test samples obtained from random training-test splits (i.e. the 20% of the dataset not used for training). The K dimension of the latent space has been set to 2 in order to allow a 2D visualisation. Plots derived from both the optimisation of the JMVIB−T objective (Eq 8) and the optimisation of the JMVIB objective (Eq 5) are included. Five copies of all plots are available, as they are obtained by training the model with five different independent training-test random splits. (ZIP) [file pcbi.1010050.s007.zip › s5-file/bce-only/J/Colorectal-EMBL/1_embeddings.png]

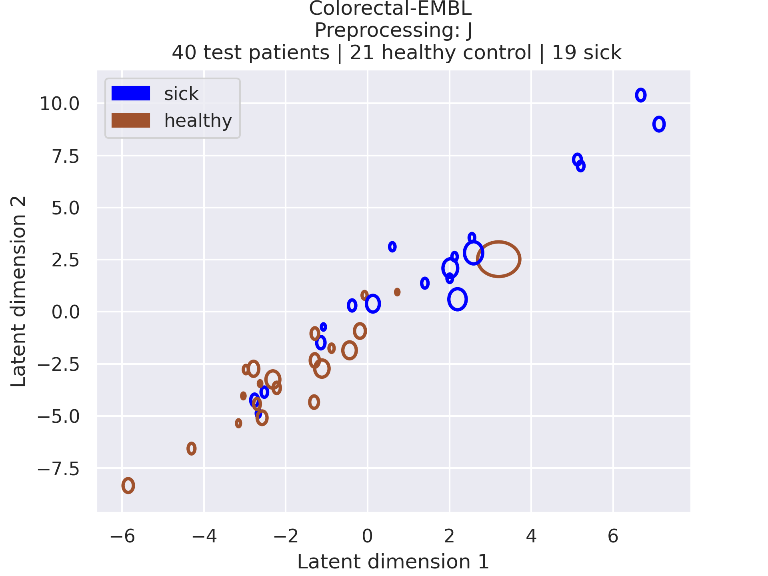

Supplement: S2 File — For all datasets considered in this work, this file presents plots of the 2D MVIB stochastic encodings analogous to Fig 3. The depicted curves are the 95% confidence intervals of the samples’ stochastic encodings z∼p(z|x)=N(μ,σ2I); the points are their means μ. The displayed encodings consist only in the test samples obtained from random training-test splits (i.e. the 20% of the dataset not used for training). The K dimension of the latent space has been set to 2 in order to allow a 2D visualisation. Plots derived from both the optimisation of the JMVIB−T objective (Eq 8) and the optimisation of the JMVIB objective (Eq 5) are included. Five copies of all plots are available, as they are obtained by training the model with five different independent training-test random splits. (ZIP) [file pcbi.1010050.s007.zip › s5-file/bce-only/J/Colorectal-EMBL/1_embeddings_95_confidence.png]

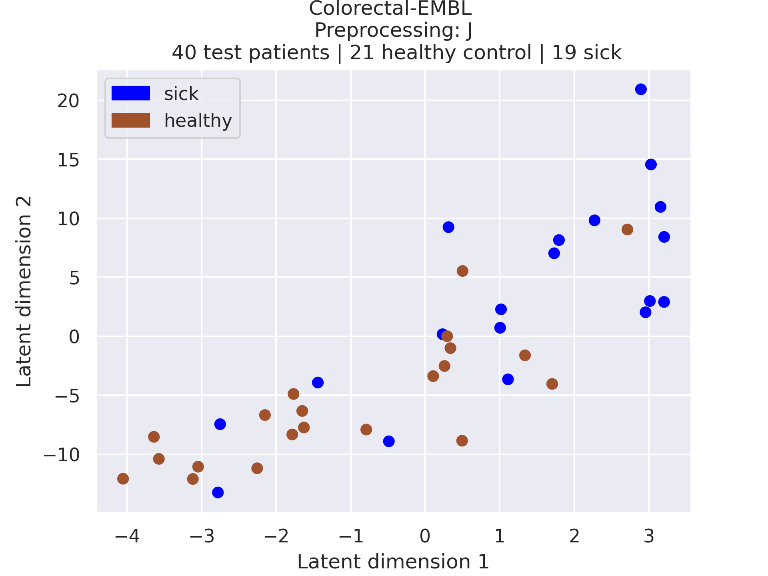

Supplement: S2 File — For all datasets considered in this work, this file presents plots of the 2D MVIB stochastic encodings analogous to Fig 3. The depicted curves are the 95% confidence intervals of the samples’ stochastic encodings z∼p(z|x)=N(μ,σ2I); the points are their means μ. The displayed encodings consist only in the test samples obtained from random training-test splits (i.e. the 20% of the dataset not used for training). The K dimension of the latent space has been set to 2 in order to allow a 2D visualisation. Plots derived from both the optimisation of the JMVIB−T objective (Eq 8) and the optimisation of the JMVIB objective (Eq 5) are included. Five copies of all plots are available, as they are obtained by training the model with five different independent training-test random splits. (ZIP) [file pcbi.1010050.s007.zip › s5-file/bce-only/J/Colorectal-EMBL/2_embeddings.png]

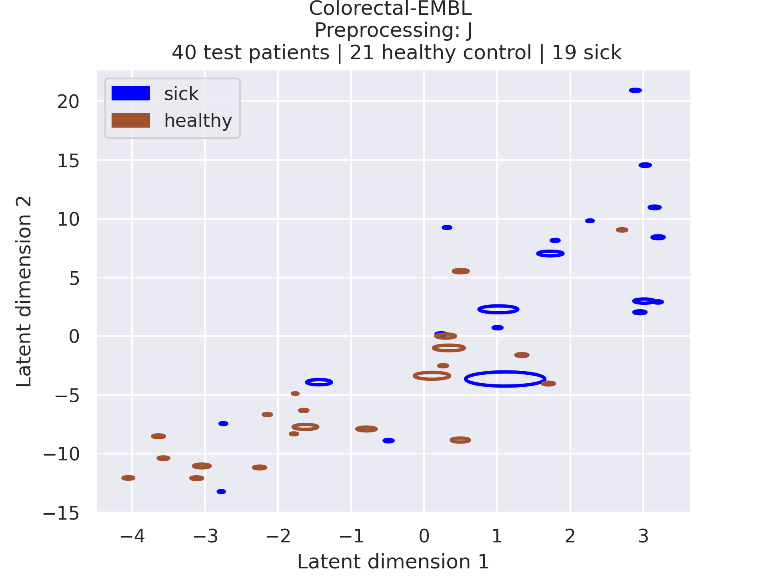

Supplement: S2 File — For all datasets considered in this work, this file presents plots of the 2D MVIB stochastic encodings analogous to Fig 3. The depicted curves are the 95% confidence intervals of the samples’ stochastic encodings z∼p(z|x)=N(μ,σ2I); the points are their means μ. The displayed encodings consist only in the test samples obtained from random training-test splits (i.e. the 20% of the dataset not used for training). The K dimension of the latent space has been set to 2 in order to allow a 2D visualisation. Plots derived from both the optimisation of the JMVIB−T objective (Eq 8) and the optimisation of the JMVIB objective (Eq 5) are included. Five copies of all plots are available, as they are obtained by training the model with five different independent training-test random splits. (ZIP) [file pcbi.1010050.s007.zip › s5-file/bce-only/J/Colorectal-EMBL/2_embeddings_95_confidence.png]

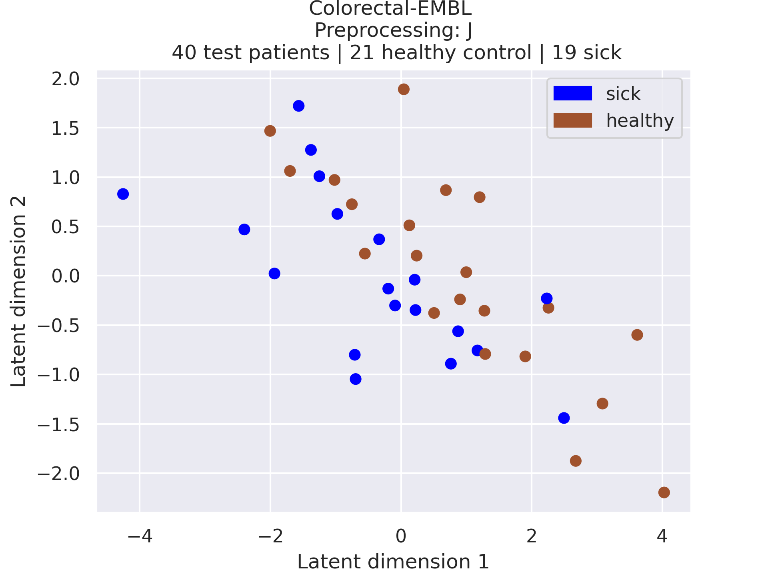

Supplement: S2 File — For all datasets considered in this work, this file presents plots of the 2D MVIB stochastic encodings analogous to Fig 3. The depicted curves are the 95% confidence intervals of the samples’ stochastic encodings z∼p(z|x)=N(μ,σ2I); the points are their means μ. The displayed encodings consist only in the test samples obtained from random training-test splits (i.e. the 20% of the dataset not used for training). The K dimension of the latent space has been set to 2 in order to allow a 2D visualisation. Plots derived from both the optimisation of the JMVIB−T objective (Eq 8) and the optimisation of the JMVIB objective (Eq 5) are included. Five copies of all plots are available, as they are obtained by training the model with five different independent training-test random splits. (ZIP) [file pcbi.1010050.s007.zip › s5-file/bce-only/J/Colorectal-EMBL/3_embeddings.png]

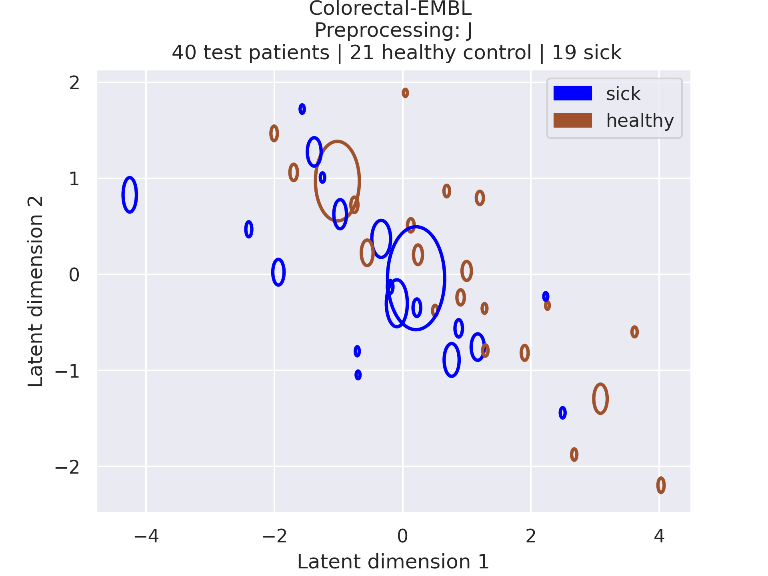

Supplement: S2 File — For all datasets considered in this work, this file presents plots of the 2D MVIB stochastic encodings analogous to Fig 3. The depicted curves are the 95% confidence intervals of the samples’ stochastic encodings z∼p(z|x)=N(μ,σ2I); the points are their means μ. The displayed encodings consist only in the test samples obtained from random training-test splits (i.e. the 20% of the dataset not used for training). The K dimension of the latent space has been set to 2 in order to allow a 2D visualisation. Plots derived from both the optimisation of the JMVIB−T objective (Eq 8) and the optimisation of the JMVIB objective (Eq 5) are included. Five copies of all plots are available, as they are obtained by training the model with five different independent training-test random splits. (ZIP) [file pcbi.1010050.s007.zip › s5-file/bce-only/J/Colorectal-EMBL/3_embeddings_95_confidence.png]

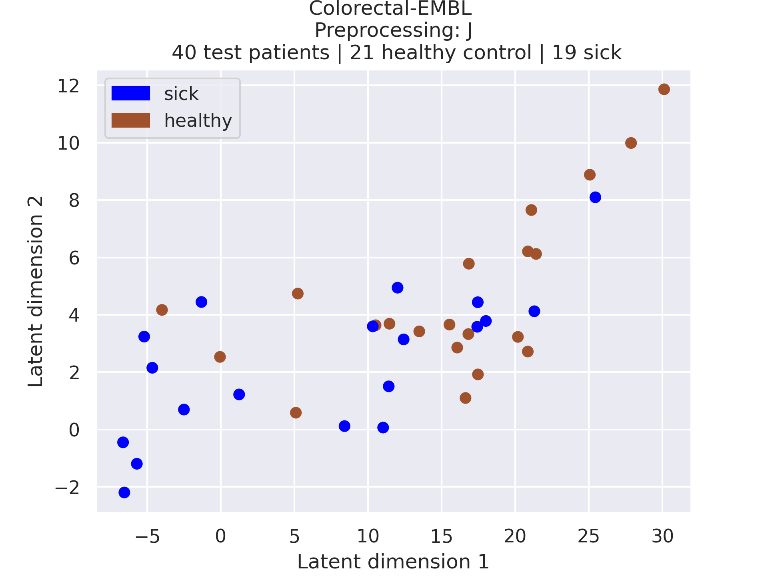

Supplement: S2 File — For all datasets considered in this work, this file presents plots of the 2D MVIB stochastic encodings analogous to Fig 3. The depicted curves are the 95% confidence intervals of the samples’ stochastic encodings z∼p(z|x)=N(μ,σ2I); the points are their means μ. The displayed encodings consist only in the test samples obtained from random training-test splits (i.e. the 20% of the dataset not used for training). The K dimension of the latent space has been set to 2 in order to allow a 2D visualisation. Plots derived from both the optimisation of the JMVIB−T objective (Eq 8) and the optimisation of the JMVIB objective (Eq 5) are included. Five copies of all plots are available, as they are obtained by training the model with five different independent training-test random splits. (ZIP) [file pcbi.1010050.s007.zip › s5-file/bce-only/J/Colorectal-EMBL/4_embeddings.png]

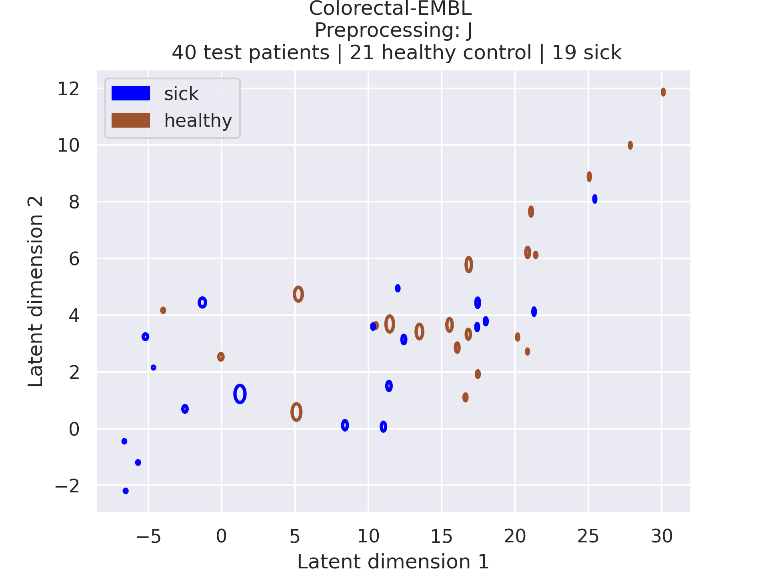

Supplement: S2 File — For all datasets considered in this work, this file presents plots of the 2D MVIB stochastic encodings analogous to Fig 3. The depicted curves are the 95% confidence intervals of the samples’ stochastic encodings z∼p(z|x)=N(μ,σ2I); the points are their means μ. The displayed encodings consist only in the test samples obtained from random training-test splits (i.e. the 20% of the dataset not used for training). The K dimension of the latent space has been set to 2 in order to allow a 2D visualisation. Plots derived from both the optimisation of the JMVIB−T objective (Eq 8) and the optimisation of the JMVIB objective (Eq 5) are included. Five copies of all plots are available, as they are obtained by training the model with five different independent training-test random splits. (ZIP) [file pcbi.1010050.s007.zip › s5-file/bce-only/J/Colorectal-EMBL/4_embeddings_95_confidence.png]

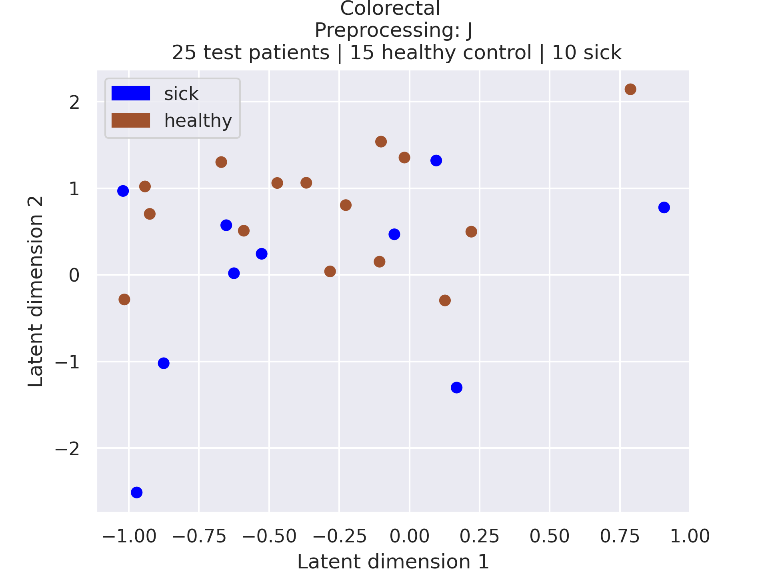

Supplement: S2 File — For all datasets considered in this work, this file presents plots of the 2D MVIB stochastic encodings analogous to Fig 3. The depicted curves are the 95% confidence intervals of the samples’ stochastic encodings z∼p(z|x)=N(μ,σ2I); the points are their means μ. The displayed encodings consist only in the test samples obtained from random training-test splits (i.e. the 20% of the dataset not used for training). The K dimension of the latent space has been set to 2 in order to allow a 2D visualisation. Plots derived from both the optimisation of the JMVIB−T objective (Eq 8) and the optimisation of the JMVIB objective (Eq 5) are included. Five copies of all plots are available, as they are obtained by training the model with five different independent training-test random splits. (ZIP) [file pcbi.1010050.s007.zip › s5-file/bce-only/J/Colorectal/0_embeddings.png]

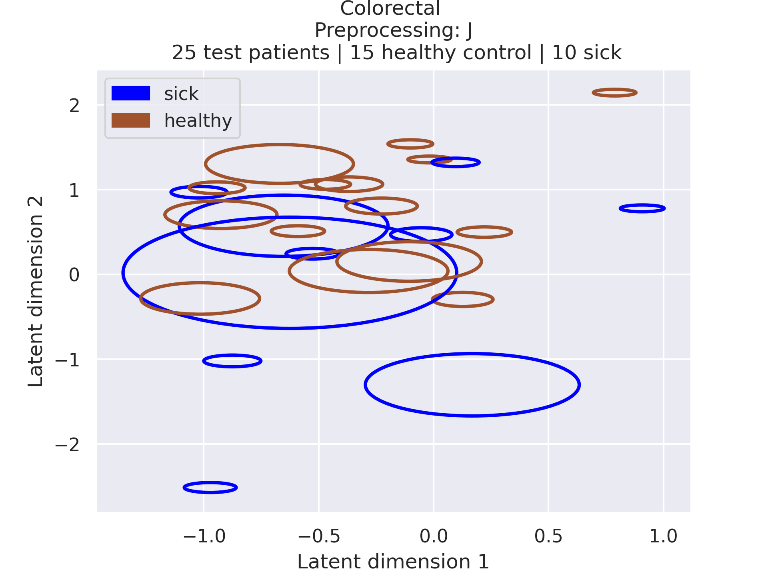

Supplement: S2 File — For all datasets considered in this work, this file presents plots of the 2D MVIB stochastic encodings analogous to Fig 3. The depicted curves are the 95% confidence intervals of the samples’ stochastic encodings z∼p(z|x)=N(μ,σ2I); the points are their means μ. The displayed encodings consist only in the test samples obtained from random training-test splits (i.e. the 20% of the dataset not used for training). The K dimension of the latent space has been set to 2 in order to allow a 2D visualisation. Plots derived from both the optimisation of the JMVIB−T objective (Eq 8) and the optimisation of the JMVIB objective (Eq 5) are included. Five copies of all plots are available, as they are obtained by training the model with five different independent training-test random splits. (ZIP) [file pcbi.1010050.s007.zip › s5-file/bce-only/J/Colorectal/0_embeddings_95_confidence.png]

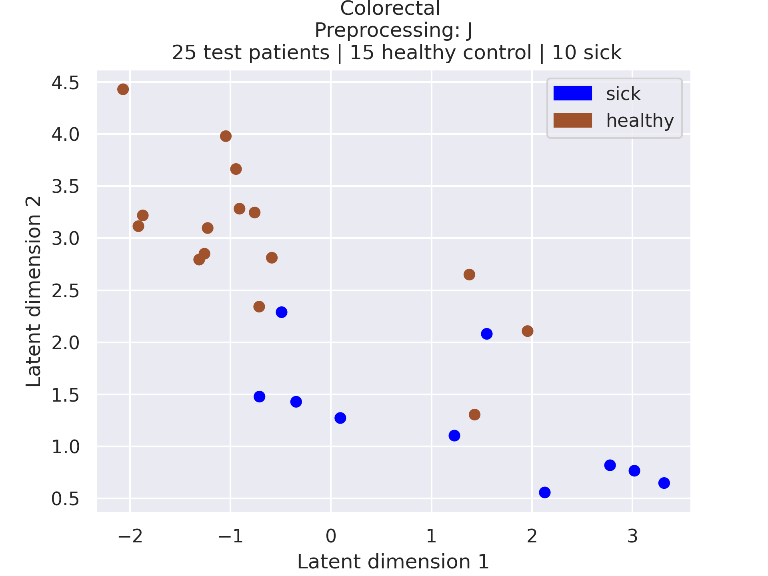

Supplement: S2 File — For all datasets considered in this work, this file presents plots of the 2D MVIB stochastic encodings analogous to Fig 3. The depicted curves are the 95% confidence intervals of the samples’ stochastic encodings z∼p(z|x)=N(μ,σ2I); the points are their means μ. The displayed encodings consist only in the test samples obtained from random training-test splits (i.e. the 20% of the dataset not used for training). The K dimension of the latent space has been set to 2 in order to allow a 2D visualisation. Plots derived from both the optimisation of the JMVIB−T objective (Eq 8) and the optimisation of the JMVIB objective (Eq 5) are included. Five copies of all plots are available, as they are obtained by training the model with five different independent training-test random splits. (ZIP) [file pcbi.1010050.s007.zip › s5-file/bce-only/J/Colorectal/1_embeddings.png]

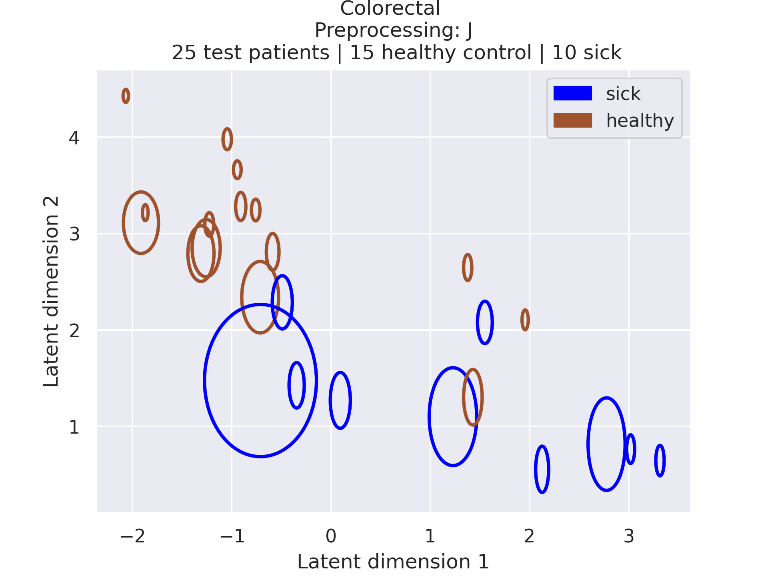

Supplement: S2 File — For all datasets considered in this work, this file presents plots of the 2D MVIB stochastic encodings analogous to Fig 3. The depicted curves are the 95% confidence intervals of the samples’ stochastic encodings z∼p(z|x)=N(μ,σ2I); the points are their means μ. The displayed encodings consist only in the test samples obtained from random training-test splits (i.e. the 20% of the dataset not used for training). The K dimension of the latent space has been set to 2 in order to allow a 2D visualisation. Plots derived from both the optimisation of the JMVIB−T objective (Eq 8) and the optimisation of the JMVIB objective (Eq 5) are included. Five copies of all plots are available, as they are obtained by training the model with five different independent training-test random splits. (ZIP) [file pcbi.1010050.s007.zip › s5-file/bce-only/J/Colorectal/1_embeddings_95_confidence.png]

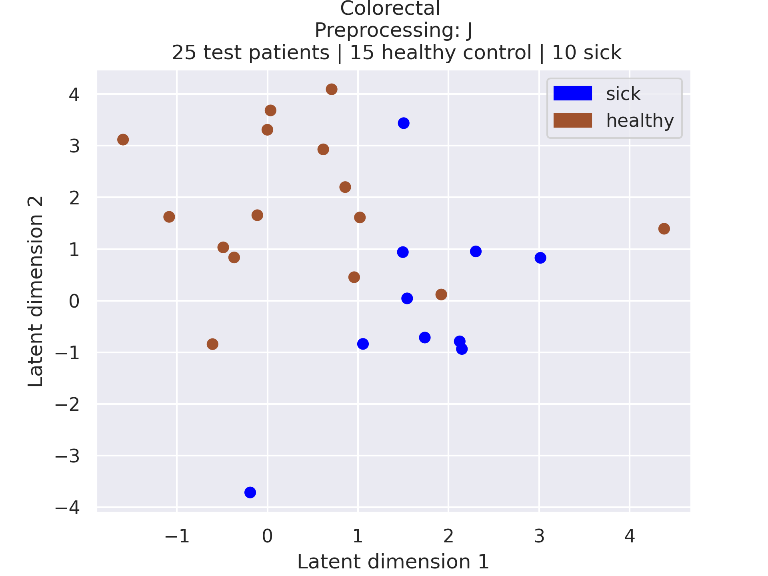

Supplement: S2 File — For all datasets considered in this work, this file presents plots of the 2D MVIB stochastic encodings analogous to Fig 3. The depicted curves are the 95% confidence intervals of the samples’ stochastic encodings z∼p(z|x)=N(μ,σ2I); the points are their means μ. The displayed encodings consist only in the test samples obtained from random training-test splits (i.e. the 20% of the dataset not used for training). The K dimension of the latent space has been set to 2 in order to allow a 2D visualisation. Plots derived from both the optimisation of the JMVIB−T objective (Eq 8) and the optimisation of the JMVIB objective (Eq 5) are included. Five copies of all plots are available, as they are obtained by training the model with five different independent training-test random splits. (ZIP) [file pcbi.1010050.s007.zip › s5-file/bce-only/J/Colorectal/2_embeddings.png]

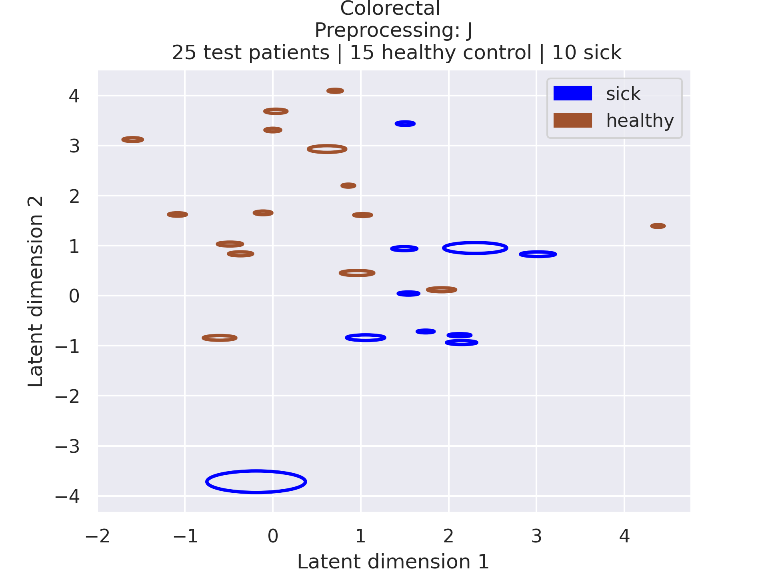

Supplement: S2 File — For all datasets considered in this work, this file presents plots of the 2D MVIB stochastic encodings analogous to Fig 3. The depicted curves are the 95% confidence intervals of the samples’ stochastic encodings z∼p(z|x)=N(μ,σ2I); the points are their means μ. The displayed encodings consist only in the test samples obtained from random training-test splits (i.e. the 20% of the dataset not used for training). The K dimension of the latent space has been set to 2 in order to allow a 2D visualisation. Plots derived from both the optimisation of the JMVIB−T objective (Eq 8) and the optimisation of the JMVIB objective (Eq 5) are included. Five copies of all plots are available, as they are obtained by training the model with five different independent training-test random splits. (ZIP) [file pcbi.1010050.s007.zip › s5-file/bce-only/J/Colorectal/2_embeddings_95_confidence.png]

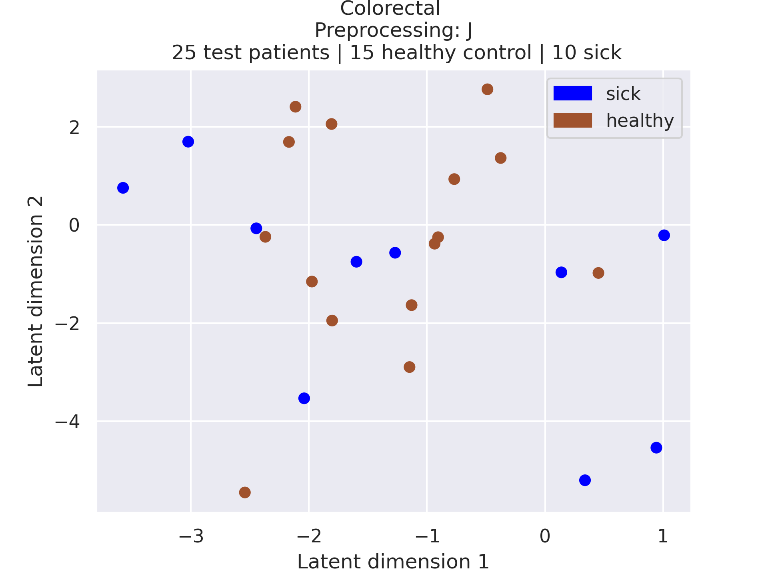

Supplement: S2 File — For all datasets considered in this work, this file presents plots of the 2D MVIB stochastic encodings analogous to Fig 3. The depicted curves are the 95% confidence intervals of the samples’ stochastic encodings z∼p(z|x)=N(μ,σ2I); the points are their means μ. The displayed encodings consist only in the test samples obtained from random training-test splits (i.e. the 20% of the dataset not used for training). The K dimension of the latent space has been set to 2 in order to allow a 2D visualisation. Plots derived from both the optimisation of the JMVIB−T objective (Eq 8) and the optimisation of the JMVIB objective (Eq 5) are included. Five copies of all plots are available, as they are obtained by training the model with five different independent training-test random splits. (ZIP) [file pcbi.1010050.s007.zip › s5-file/bce-only/J/Colorectal/3_embeddings.png]

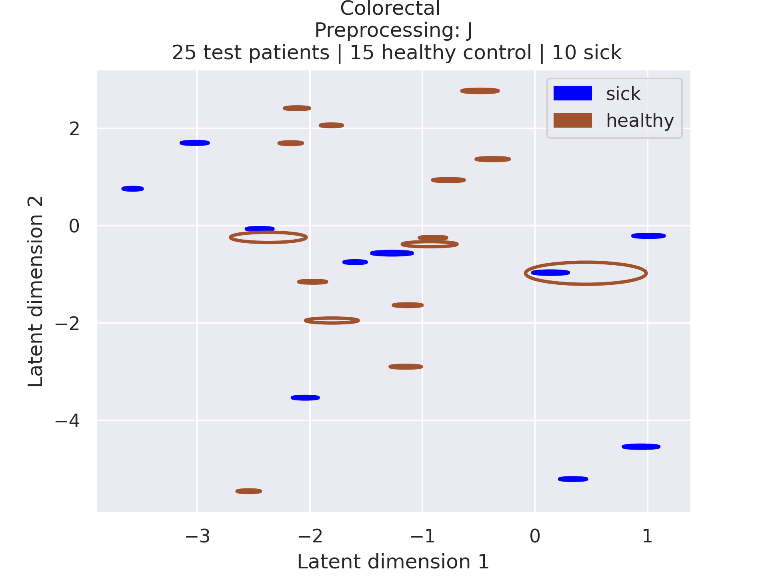

Supplement: S2 File — For all datasets considered in this work, this file presents plots of the 2D MVIB stochastic encodings analogous to Fig 3. The depicted curves are the 95% confidence intervals of the samples’ stochastic encodings z∼p(z|x)=N(μ,σ2I); the points are their means μ. The displayed encodings consist only in the test samples obtained from random training-test splits (i.e. the 20% of the dataset not used for training). The K dimension of the latent space has been set to 2 in order to allow a 2D visualisation. Plots derived from both the optimisation of the JMVIB−T objective (Eq 8) and the optimisation of the JMVIB objective (Eq 5) are included. Five copies of all plots are available, as they are obtained by training the model with five different independent training-test random splits. (ZIP) [file pcbi.1010050.s007.zip › s5-file/bce-only/J/Colorectal/3_embeddings_95_confidence.png]

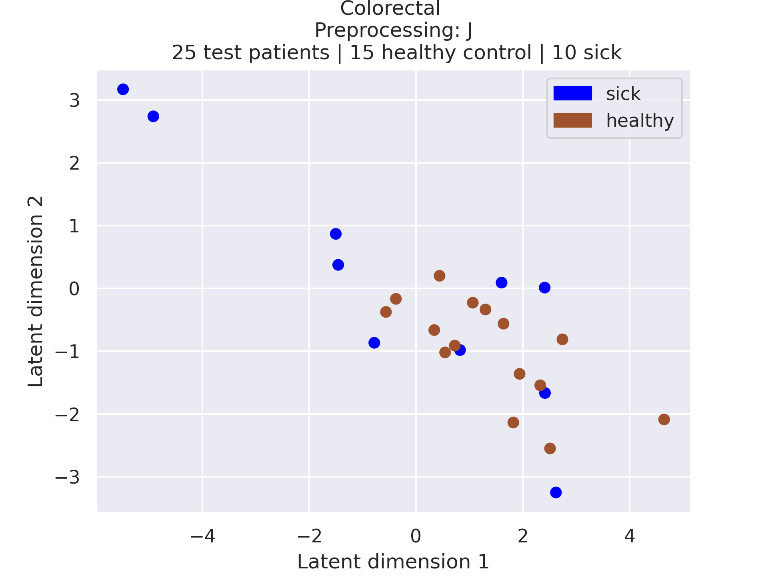

Supplement: S2 File — For all datasets considered in this work, this file presents plots of the 2D MVIB stochastic encodings analogous to Fig 3. The depicted curves are the 95% confidence intervals of the samples’ stochastic encodings z∼p(z|x)=N(μ,σ2I); the points are their means μ. The displayed encodings consist only in the test samples obtained from random training-test splits (i.e. the 20% of the dataset not used for training). The K dimension of the latent space has been set to 2 in order to allow a 2D visualisation. Plots derived from both the optimisation of the JMVIB−T objective (Eq 8) and the optimisation of the JMVIB objective (Eq 5) are included. Five copies of all plots are available, as they are obtained by training the model with five different independent training-test random splits. (ZIP) [file pcbi.1010050.s007.zip › s5-file/bce-only/J/Colorectal/4_embeddings.png]

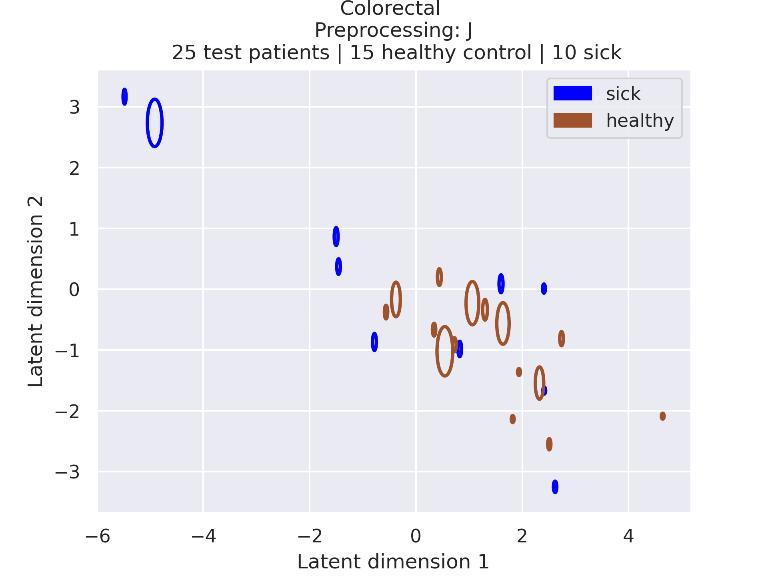

Supplement: S2 File — For all datasets considered in this work, this file presents plots of the 2D MVIB stochastic encodings analogous to Fig 3. The depicted curves are the 95% confidence intervals of the samples’ stochastic encodings z∼p(z|x)=N(μ,σ2I); the points are their means μ. The displayed encodings consist only in the test samples obtained from random training-test splits (i.e. the 20% of the dataset not used for training). The K dimension of the latent space has been set to 2 in order to allow a 2D visualisation. Plots derived from both the optimisation of the JMVIB−T objective (Eq 8) and the optimisation of the JMVIB objective (Eq 5) are included. Five copies of all plots are available, as they are obtained by training the model with five different independent training-test random splits. (ZIP) [file pcbi.1010050.s007.zip › s5-file/bce-only/J/Colorectal/4_embeddings_95_confidence.png]

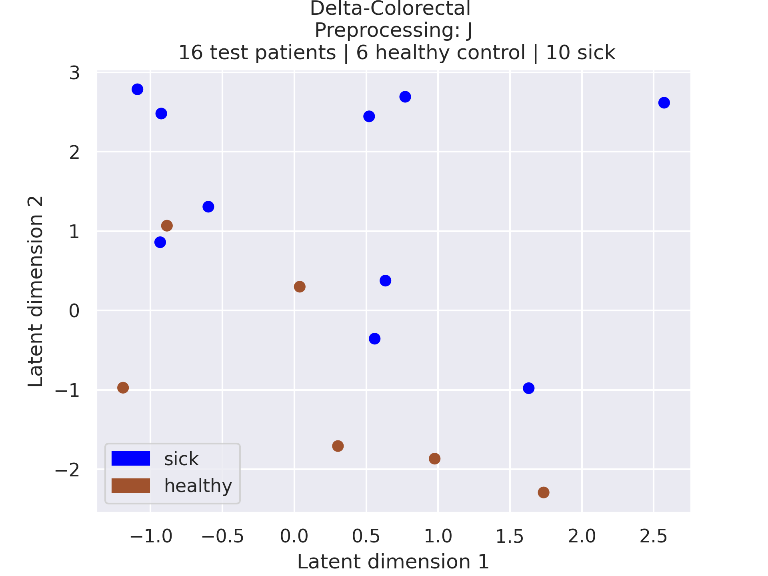

Supplement: S2 File — For all datasets considered in this work, this file presents plots of the 2D MVIB stochastic encodings analogous to Fig 3. The depicted curves are the 95% confidence intervals of the samples’ stochastic encodings z∼p(z|x)=N(μ,σ2I); the points are their means μ. The displayed encodings consist only in the test samples obtained from random training-test splits (i.e. the 20% of the dataset not used for training). The K dimension of the latent space has been set to 2 in order to allow a 2D visualisation. Plots derived from both the optimisation of the JMVIB−T objective (Eq 8) and the optimisation of the JMVIB objective (Eq 5) are included. Five copies of all plots are available, as they are obtained by training the model with five different independent training-test random splits. (ZIP) [file pcbi.1010050.s007.zip › s5-file/bce-only/J/Delta-Colorectal/0_embeddings.png]

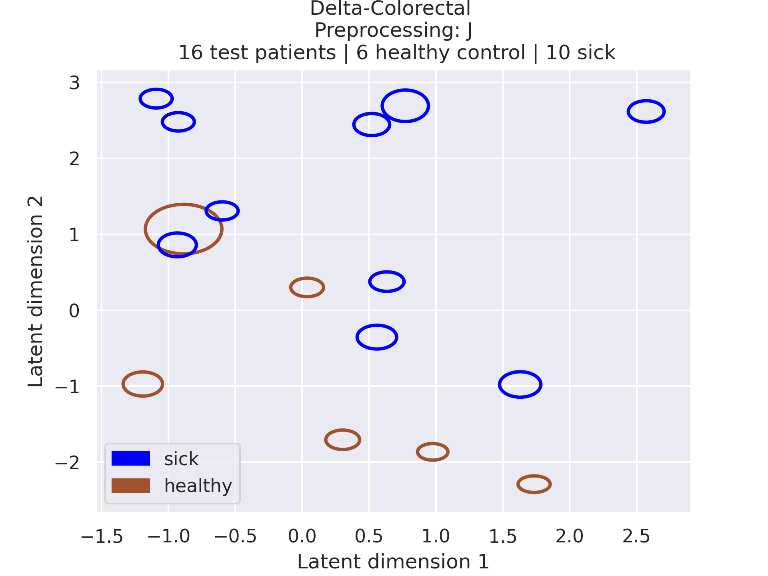

Supplement: S2 File — For all datasets considered in this work, this file presents plots of the 2D MVIB stochastic encodings analogous to Fig 3. The depicted curves are the 95% confidence intervals of the samples’ stochastic encodings z∼p(z|x)=N(μ,σ2I); the points are their means μ. The displayed encodings consist only in the test samples obtained from random training-test splits (i.e. the 20% of the dataset not used for training). The K dimension of the latent space has been set to 2 in order to allow a 2D visualisation. Plots derived from both the optimisation of the JMVIB−T objective (Eq 8) and the optimisation of the JMVIB objective (Eq 5) are included. Five copies of all plots are available, as they are obtained by training the model with five different independent training-test random splits. (ZIP) [file pcbi.1010050.s007.zip › s5-file/bce-only/J/Delta-Colorectal/0_embeddings_95_confidence.png]

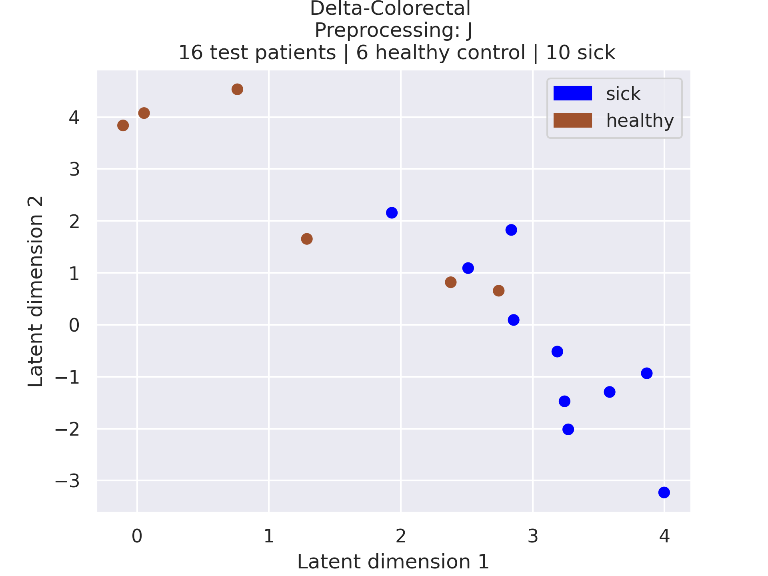

Supplement: S2 File — For all datasets considered in this work, this file presents plots of the 2D MVIB stochastic encodings analogous to Fig 3. The depicted curves are the 95% confidence intervals of the samples’ stochastic encodings z∼p(z|x)=N(μ,σ2I); the points are their means μ. The displayed encodings consist only in the test samples obtained from random training-test splits (i.e. the 20% of the dataset not used for training). The K dimension of the latent space has been set to 2 in order to allow a 2D visualisation. Plots derived from both the optimisation of the JMVIB−T objective (Eq 8) and the optimisation of the JMVIB objective (Eq 5) are included. Five copies of all plots are available, as they are obtained by training the model with five different independent training-test random splits. (ZIP) [file pcbi.1010050.s007.zip › s5-file/bce-only/J/Delta-Colorectal/1_embeddings.png]

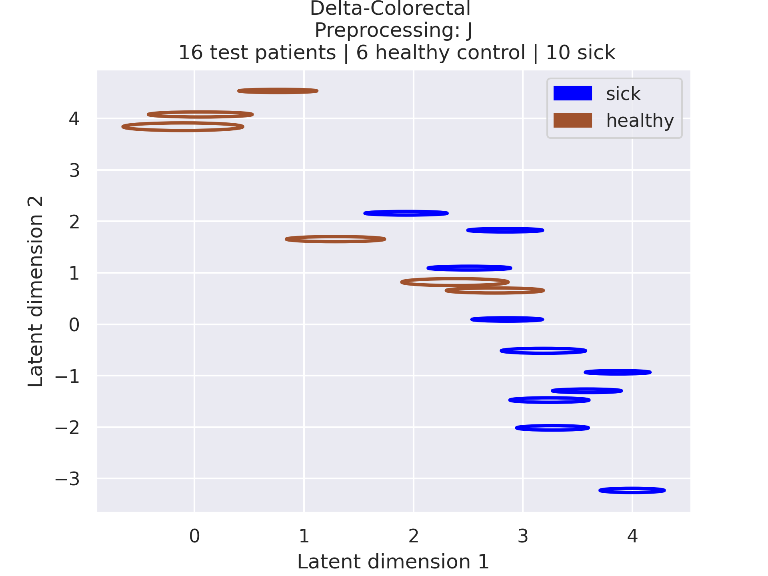

Supplement: S2 File — For all datasets considered in this work, this file presents plots of the 2D MVIB stochastic encodings analogous to Fig 3. The depicted curves are the 95% confidence intervals of the samples’ stochastic encodings z∼p(z|x)=N(μ,σ2I); the points are their means μ. The displayed encodings consist only in the test samples obtained from random training-test splits (i.e. the 20% of the dataset not used for training). The K dimension of the latent space has been set to 2 in order to allow a 2D visualisation. Plots derived from both the optimisation of the JMVIB−T objective (Eq 8) and the optimisation of the JMVIB objective (Eq 5) are included. Five copies of all plots are available, as they are obtained by training the model with five different independent training-test random splits. (ZIP) [file pcbi.1010050.s007.zip › s5-file/bce-only/J/Delta-Colorectal/1_embeddings_95_confidence.png]

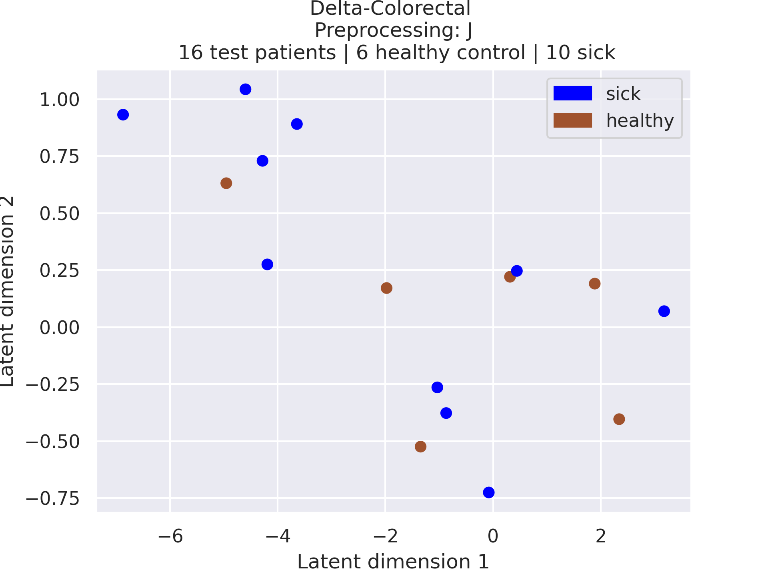

Supplement: S2 File — For all datasets considered in this work, this file presents plots of the 2D MVIB stochastic encodings analogous to Fig 3. The depicted curves are the 95% confidence intervals of the samples’ stochastic encodings z∼p(z|x)=N(μ,σ2I); the points are their means μ. The displayed encodings consist only in the test samples obtained from random training-test splits (i.e. the 20% of the dataset not used for training). The K dimension of the latent space has been set to 2 in order to allow a 2D visualisation. Plots derived from both the optimisation of the JMVIB−T objective (Eq 8) and the optimisation of the JMVIB objective (Eq 5) are included. Five copies of all plots are available, as they are obtained by training the model with five different independent training-test random splits. (ZIP) [file pcbi.1010050.s007.zip › s5-file/bce-only/J/Delta-Colorectal/2_embeddings.png]

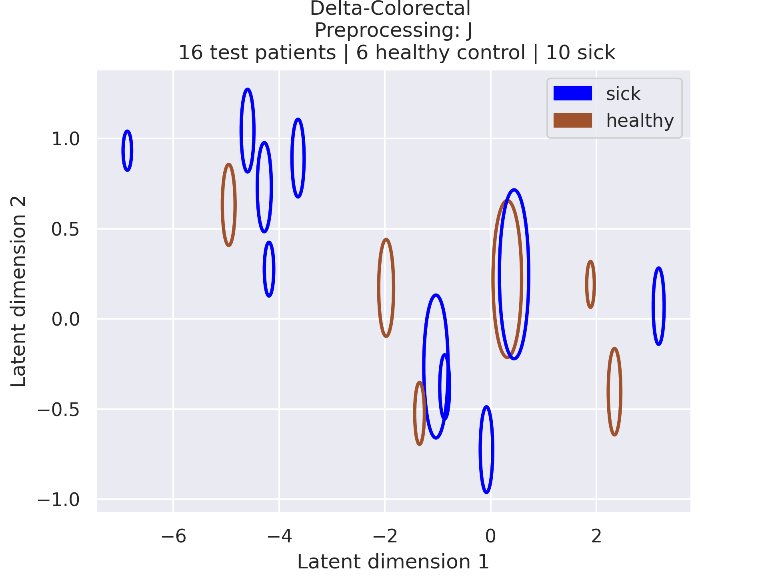

Supplement: S2 File — For all datasets considered in this work, this file presents plots of the 2D MVIB stochastic encodings analogous to Fig 3. The depicted curves are the 95% confidence intervals of the samples’ stochastic encodings z∼p(z|x)=N(μ,σ2I); the points are their means μ. The displayed encodings consist only in the test samples obtained from random training-test splits (i.e. the 20% of the dataset not used for training). The K dimension of the latent space has been set to 2 in order to allow a 2D visualisation. Plots derived from both the optimisation of the JMVIB−T objective (Eq 8) and the optimisation of the JMVIB objective (Eq 5) are included. Five copies of all plots are available, as they are obtained by training the model with five different independent training-test random splits. (ZIP) [file pcbi.1010050.s007.zip › s5-file/bce-only/J/Delta-Colorectal/2_embeddings_95_confidence.png]

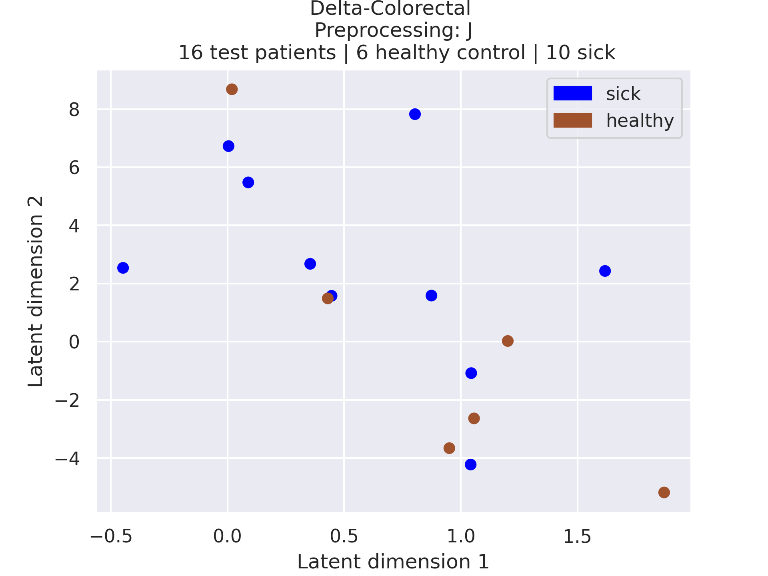

Supplement: S2 File — For all datasets considered in this work, this file presents plots of the 2D MVIB stochastic encodings analogous to Fig 3. The depicted curves are the 95% confidence intervals of the samples’ stochastic encodings z∼p(z|x)=N(μ,σ2I); the points are their means μ. The displayed encodings consist only in the test samples obtained from random training-test splits (i.e. the 20% of the dataset not used for training). The K dimension of the latent space has been set to 2 in order to allow a 2D visualisation. Plots derived from both the optimisation of the JMVIB−T objective (Eq 8) and the optimisation of the JMVIB objective (Eq 5) are included. Five copies of all plots are available, as they are obtained by training the model with five different independent training-test random splits. (ZIP) [file pcbi.1010050.s007.zip › s5-file/bce-only/J/Delta-Colorectal/3_embeddings.png]

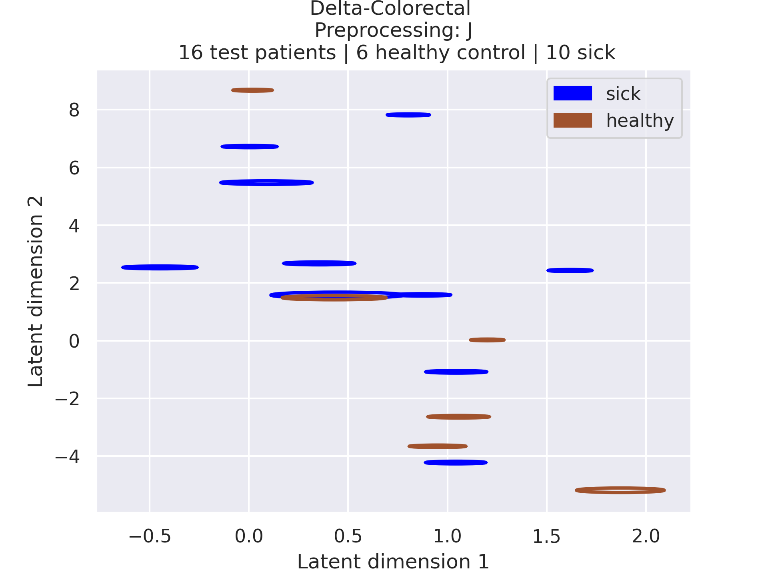

Supplement: S2 File — For all datasets considered in this work, this file presents plots of the 2D MVIB stochastic encodings analogous to Fig 3. The depicted curves are the 95% confidence intervals of the samples’ stochastic encodings z∼p(z|x)=N(μ,σ2I); the points are their means μ. The displayed encodings consist only in the test samples obtained from random training-test splits (i.e. the 20% of the dataset not used for training). The K dimension of the latent space has been set to 2 in order to allow a 2D visualisation. Plots derived from both the optimisation of the JMVIB−T objective (Eq 8) and the optimisation of the JMVIB objective (Eq 5) are included. Five copies of all plots are available, as they are obtained by training the model with five different independent training-test random splits. (ZIP) [file pcbi.1010050.s007.zip › s5-file/bce-only/J/Delta-Colorectal/3_embeddings_95_confidence.png]

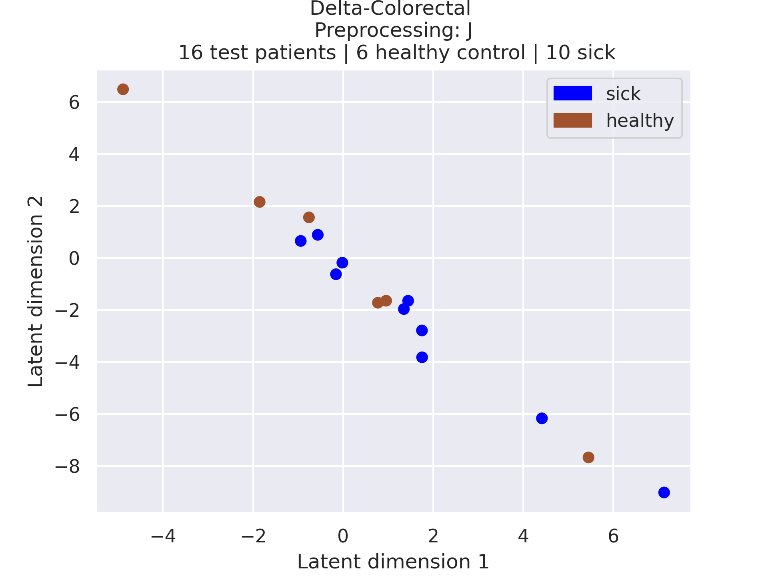

Supplement: S2 File — For all datasets considered in this work, this file presents plots of the 2D MVIB stochastic encodings analogous to Fig 3. The depicted curves are the 95% confidence intervals of the samples’ stochastic encodings z∼p(z|x)=N(μ,σ2I); the points are their means μ. The displayed encodings consist only in the test samples obtained from random training-test splits (i.e. the 20% of the dataset not used for training). The K dimension of the latent space has been set to 2 in order to allow a 2D visualisation. Plots derived from both the optimisation of the JMVIB−T objective (Eq 8) and the optimisation of the JMVIB objective (Eq 5) are included. Five copies of all plots are available, as they are obtained by training the model with five different independent training-test random splits. (ZIP) [file pcbi.1010050.s007.zip › s5-file/bce-only/J/Delta-Colorectal/4_embeddings.png]

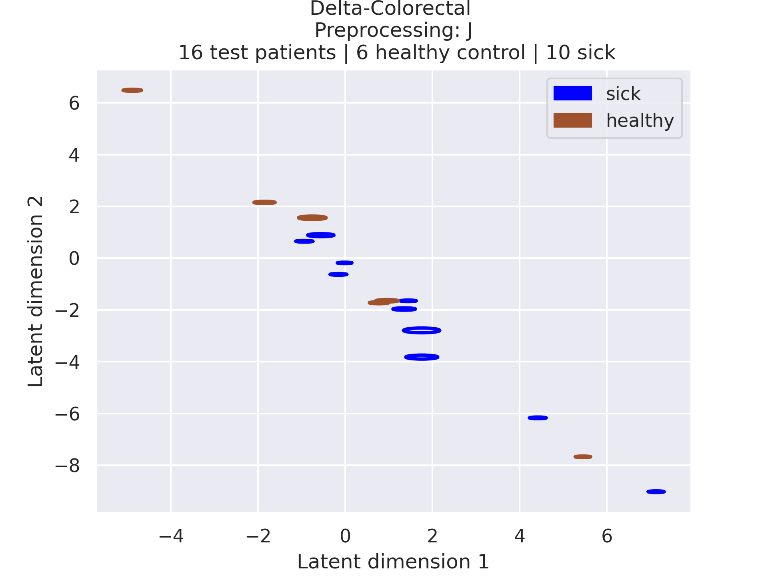

Supplement: S2 File — For all datasets considered in this work, this file presents plots of the 2D MVIB stochastic encodings analogous to Fig 3. The depicted curves are the 95% confidence intervals of the samples’ stochastic encodings z∼p(z|x)=N(μ,σ2I); the points are their means μ. The displayed encodings consist only in the test samples obtained from random training-test splits (i.e. the 20% of the dataset not used for training). The K dimension of the latent space has been set to 2 in order to allow a 2D visualisation. Plots derived from both the optimisation of the JMVIB−T objective (Eq 8) and the optimisation of the JMVIB objective (Eq 5) are included. Five copies of all plots are available, as they are obtained by training the model with five different independent training-test random splits. (ZIP) [file pcbi.1010050.s007.zip › s5-file/bce-only/J/Delta-Colorectal/4_embeddings_95_confidence.png]

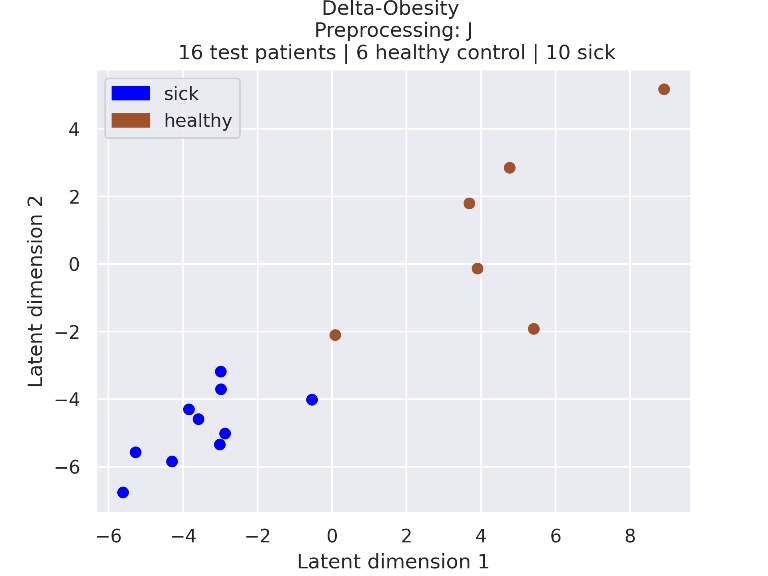

Supplement: S2 File — For all datasets considered in this work, this file presents plots of the 2D MVIB stochastic encodings analogous to Fig 3. The depicted curves are the 95% confidence intervals of the samples’ stochastic encodings z∼p(z|x)=N(μ,σ2I); the points are their means μ. The displayed encodings consist only in the test samples obtained from random training-test splits (i.e. the 20% of the dataset not used for training). The K dimension of the latent space has been set to 2 in order to allow a 2D visualisation. Plots derived from both the optimisation of the JMVIB−T objective (Eq 8) and the optimisation of the JMVIB objective (Eq 5) are included. Five copies of all plots are available, as they are obtained by training the model with five different independent training-test random splits. (ZIP) [file pcbi.1010050.s007.zip › s5-file/bce-only/J/Delta-Obesity/0_embeddings.png]

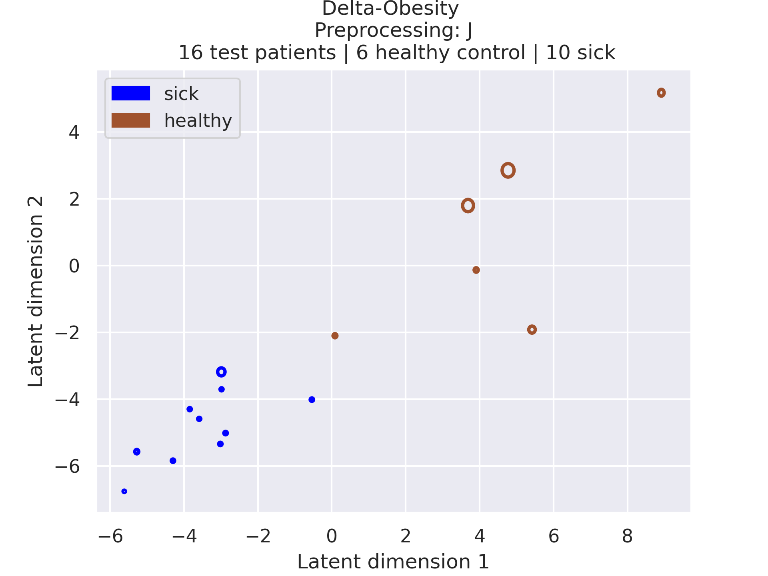

Supplement: S2 File — For all datasets considered in this work, this file presents plots of the 2D MVIB stochastic encodings analogous to Fig 3. The depicted curves are the 95% confidence intervals of the samples’ stochastic encodings z∼p(z|x)=N(μ,σ2I); the points are their means μ. The displayed encodings consist only in the test samples obtained from random training-test splits (i.e. the 20% of the dataset not used for training). The K dimension of the latent space has been set to 2 in order to allow a 2D visualisation. Plots derived from both the optimisation of the JMVIB−T objective (Eq 8) and the optimisation of the JMVIB objective (Eq 5) are included. Five copies of all plots are available, as they are obtained by training the model with five different independent training-test random splits. (ZIP) [file pcbi.1010050.s007.zip › s5-file/bce-only/J/Delta-Obesity/0_embeddings_95_confidence.png]

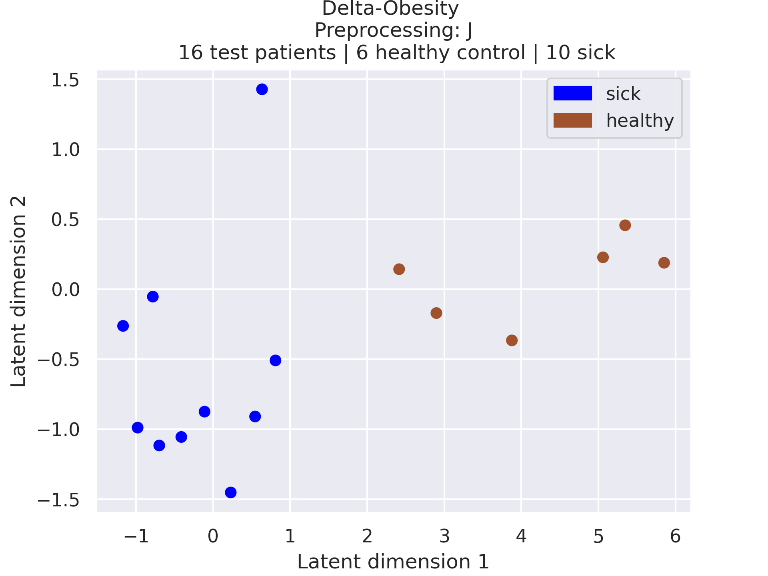

Supplement: S2 File — For all datasets considered in this work, this file presents plots of the 2D MVIB stochastic encodings analogous to Fig 3. The depicted curves are the 95% confidence intervals of the samples’ stochastic encodings z∼p(z|x)=N(μ,σ2I); the points are their means μ. The displayed encodings consist only in the test samples obtained from random training-test splits (i.e. the 20% of the dataset not used for training). The K dimension of the latent space has been set to 2 in order to allow a 2D visualisation. Plots derived from both the optimisation of the JMVIB−T objective (Eq 8) and the optimisation of the JMVIB objective (Eq 5) are included. Five copies of all plots are available, as they are obtained by training the model with five different independent training-test random splits. (ZIP) [file pcbi.1010050.s007.zip › s5-file/bce-only/J/Delta-Obesity/1_embeddings.png]

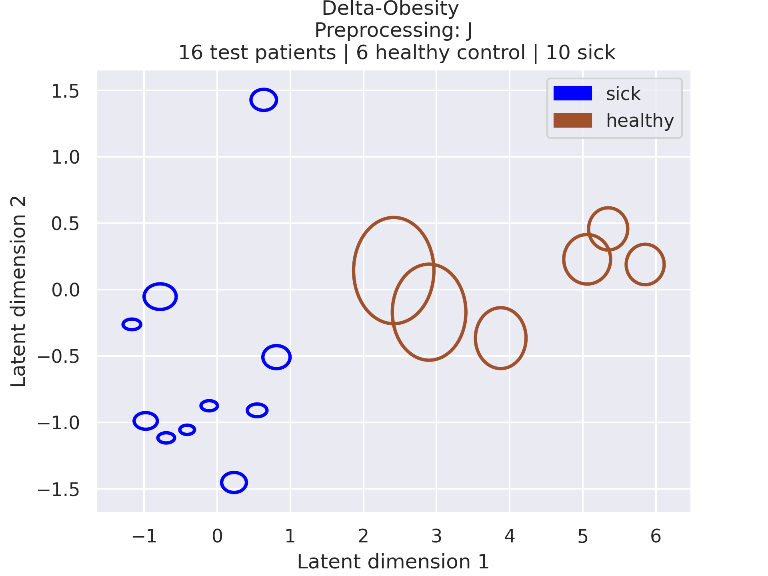

Supplement: S2 File — For all datasets considered in this work, this file presents plots of the 2D MVIB stochastic encodings analogous to Fig 3. The depicted curves are the 95% confidence intervals of the samples’ stochastic encodings z∼p(z|x)=N(μ,σ2I); the points are their means μ. The displayed encodings consist only in the test samples obtained from random training-test splits (i.e. the 20% of the dataset not used for training). The K dimension of the latent space has been set to 2 in order to allow a 2D visualisation. Plots derived from both the optimisation of the JMVIB−T objective (Eq 8) and the optimisation of the JMVIB objective (Eq 5) are included. Five copies of all plots are available, as they are obtained by training the model with five different independent training-test random splits. (ZIP) [file pcbi.1010050.s007.zip › s5-file/bce-only/J/Delta-Obesity/1_embeddings_95_confidence.png]

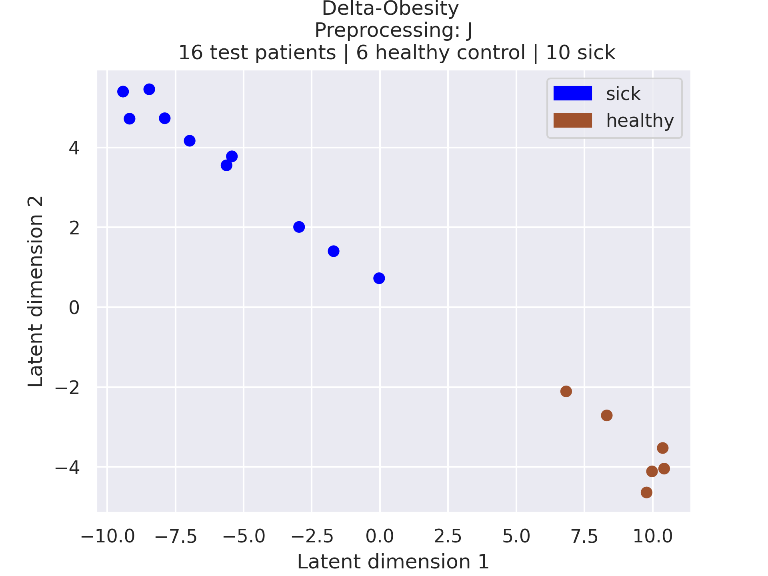

Supplement: S2 File — For all datasets considered in this work, this file presents plots of the 2D MVIB stochastic encodings analogous to Fig 3. The depicted curves are the 95% confidence intervals of the samples’ stochastic encodings z∼p(z|x)=N(μ,σ2I); the points are their means μ. The displayed encodings consist only in the test samples obtained from random training-test splits (i.e. the 20% of the dataset not used for training). The K dimension of the latent space has been set to 2 in order to allow a 2D visualisation. Plots derived from both the optimisation of the JMVIB−T objective (Eq 8) and the optimisation of the JMVIB objective (Eq 5) are included. Five copies of all plots are available, as they are obtained by training the model with five different independent training-test random splits. (ZIP) [file pcbi.1010050.s007.zip › s5-file/bce-only/J/Delta-Obesity/2_embeddings.png]

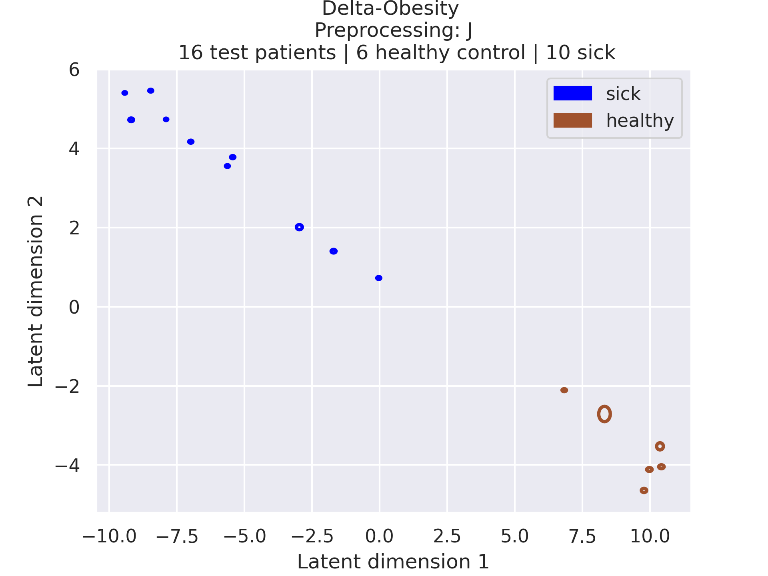

Supplement: S2 File — For all datasets considered in this work, this file presents plots of the 2D MVIB stochastic encodings analogous to Fig 3. The depicted curves are the 95% confidence intervals of the samples’ stochastic encodings z∼p(z|x)=N(μ,σ2I); the points are their means μ. The displayed encodings consist only in the test samples obtained from random training-test splits (i.e. the 20% of the dataset not used for training). The K dimension of the latent space has been set to 2 in order to allow a 2D visualisation. Plots derived from both the optimisation of the JMVIB−T objective (Eq 8) and the optimisation of the JMVIB objective (Eq 5) are included. Five copies of all plots are available, as they are obtained by training the model with five different independent training-test random splits. (ZIP) [file pcbi.1010050.s007.zip › s5-file/bce-only/J/Delta-Obesity/2_embeddings_95_confidence.png]

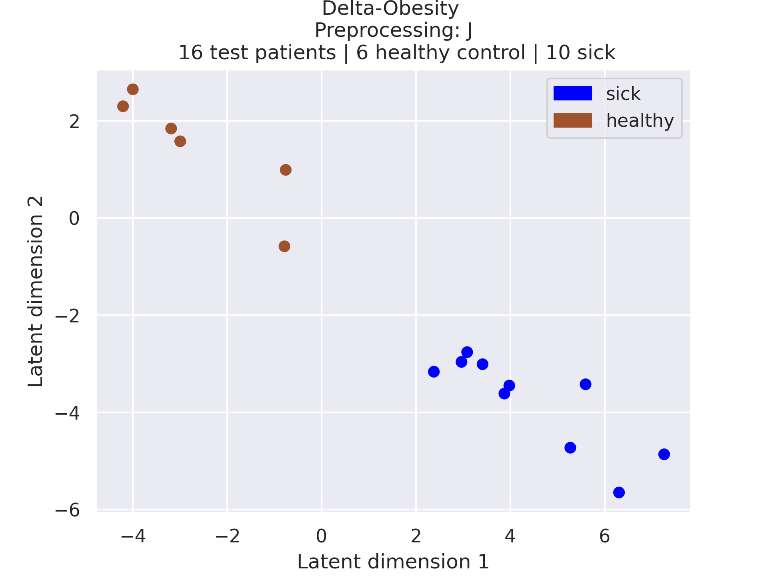

Supplement: S2 File — For all datasets considered in this work, this file presents plots of the 2D MVIB stochastic encodings analogous to Fig 3. The depicted curves are the 95% confidence intervals of the samples’ stochastic encodings z∼p(z|x)=N(μ,σ2I); the points are their means μ. The displayed encodings consist only in the test samples obtained from random training-test splits (i.e. the 20% of the dataset not used for training). The K dimension of the latent space has been set to 2 in order to allow a 2D visualisation. Plots derived from both the optimisation of the JMVIB−T objective (Eq 8) and the optimisation of the JMVIB objective (Eq 5) are included. Five copies of all plots are available, as they are obtained by training the model with five different independent training-test random splits. (ZIP) [file pcbi.1010050.s007.zip › s5-file/bce-only/J/Delta-Obesity/3_embeddings.png]

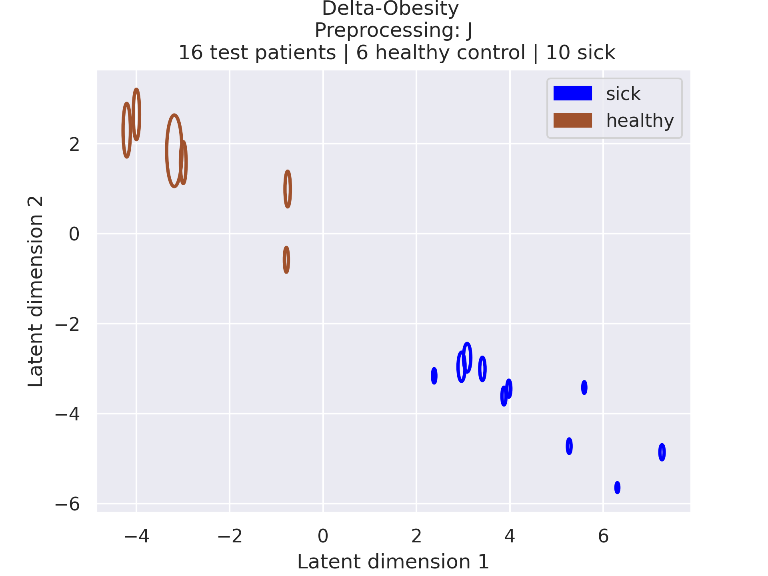

Supplement: S2 File — For all datasets considered in this work, this file presents plots of the 2D MVIB stochastic encodings analogous to Fig 3. The depicted curves are the 95% confidence intervals of the samples’ stochastic encodings z∼p(z|x)=N(μ,σ2I); the points are their means μ. The displayed encodings consist only in the test samples obtained from random training-test splits (i.e. the 20% of the dataset not used for training). The K dimension of the latent space has been set to 2 in order to allow a 2D visualisation. Plots derived from both the optimisation of the JMVIB−T objective (Eq 8) and the optimisation of the JMVIB objective (Eq 5) are included. Five copies of all plots are available, as they are obtained by training the model with five different independent training-test random splits. (ZIP) [file pcbi.1010050.s007.zip › s5-file/bce-only/J/Delta-Obesity/3_embeddings_95_confidence.png]

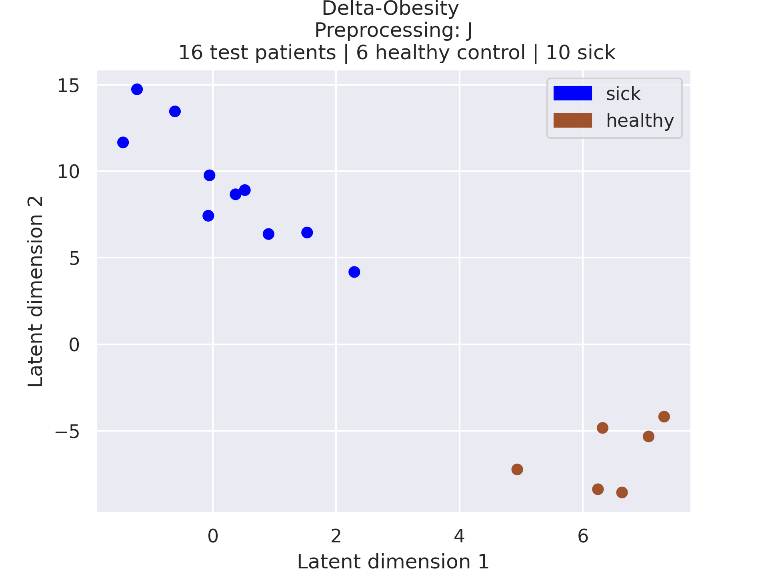

Supplement: S2 File — For all datasets considered in this work, this file presents plots of the 2D MVIB stochastic encodings analogous to Fig 3. The depicted curves are the 95% confidence intervals of the samples’ stochastic encodings z∼p(z|x)=N(μ,σ2I); the points are their means μ. The displayed encodings consist only in the test samples obtained from random training-test splits (i.e. the 20% of the dataset not used for training). The K dimension of the latent space has been set to 2 in order to allow a 2D visualisation. Plots derived from both the optimisation of the JMVIB−T objective (Eq 8) and the optimisation of the JMVIB objective (Eq 5) are included. Five copies of all plots are available, as they are obtained by training the model with five different independent training-test random splits. (ZIP) [file pcbi.1010050.s007.zip › s5-file/bce-only/J/Delta-Obesity/4_embeddings.png]

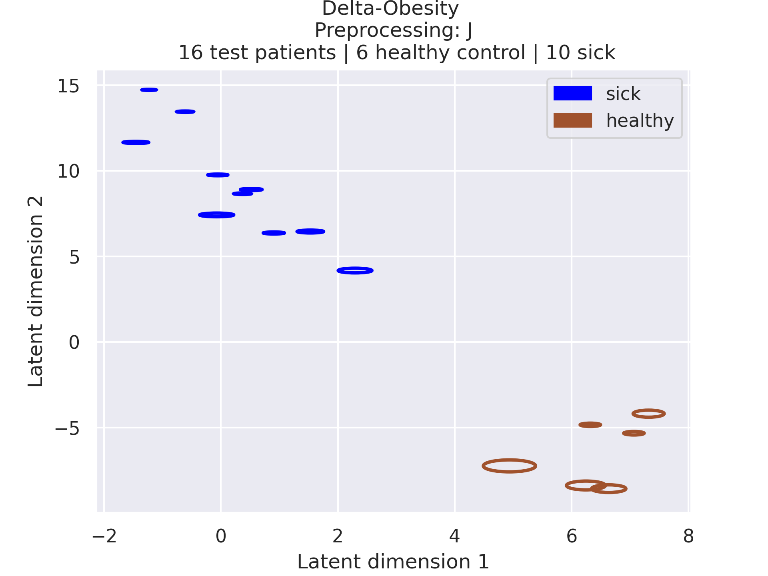

Supplement: S2 File — For all datasets considered in this work, this file presents plots of the 2D MVIB stochastic encodings analogous to Fig 3. The depicted curves are the 95% confidence intervals of the samples’ stochastic encodings z∼p(z|x)=N(μ,σ2I); the points are their means μ. The displayed encodings consist only in the test samples obtained from random training-test splits (i.e. the 20% of the dataset not used for training). The K dimension of the latent space has been set to 2 in order to allow a 2D visualisation. Plots derived from both the optimisation of the JMVIB−T objective (Eq 8) and the optimisation of the JMVIB objective (Eq 5) are included. Five copies of all plots are available, as they are obtained by training the model with five different independent training-test random splits. (ZIP) [file pcbi.1010050.s007.zip › s5-file/bce-only/J/Delta-Obesity/4_embeddings_95_confidence.png]

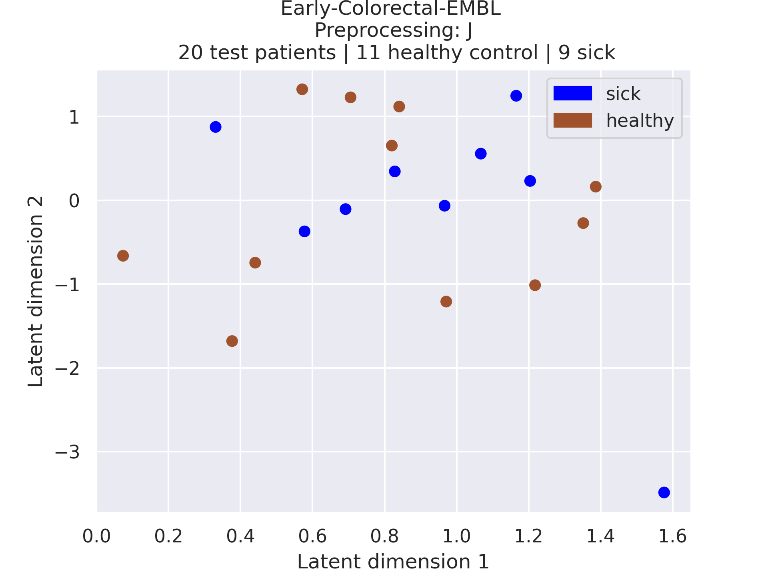

Supplement: S2 File — For all datasets considered in this work, this file presents plots of the 2D MVIB stochastic encodings analogous to Fig 3. The depicted curves are the 95% confidence intervals of the samples’ stochastic encodings z∼p(z|x)=N(μ,σ2I); the points are their means μ. The displayed encodings consist only in the test samples obtained from random training-test splits (i.e. the 20% of the dataset not used for training). The K dimension of the latent space has been set to 2 in order to allow a 2D visualisation. Plots derived from both the optimisation of the JMVIB−T objective (Eq 8) and the optimisation of the JMVIB objective (Eq 5) are included. Five copies of all plots are available, as they are obtained by training the model with five different independent training-test random splits. (ZIP) [file pcbi.1010050.s007.zip › s5-file/bce-only/J/Early-Colorectal-EMBL/0_embeddings.png]

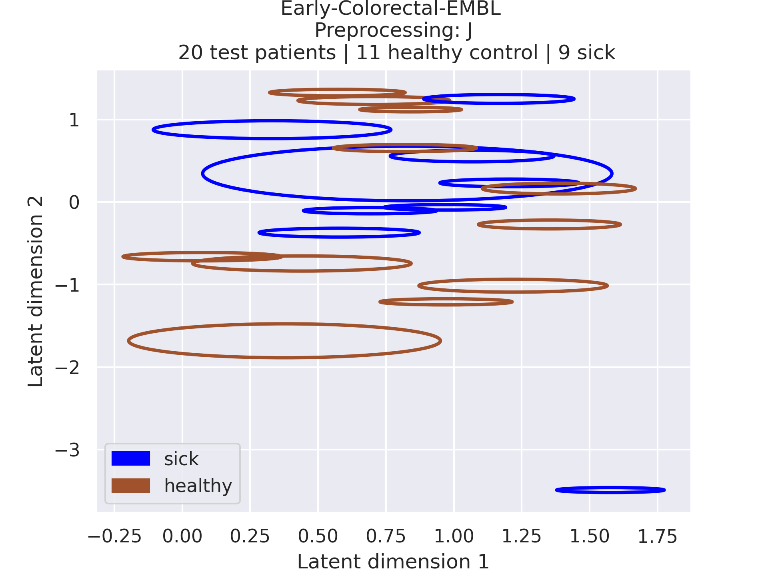

Supplement: S2 File — For all datasets considered in this work, this file presents plots of the 2D MVIB stochastic encodings analogous to Fig 3. The depicted curves are the 95% confidence intervals of the samples’ stochastic encodings z∼p(z|x)=N(μ,σ2I); the points are their means μ. The displayed encodings consist only in the test samples obtained from random training-test splits (i.e. the 20% of the dataset not used for training). The K dimension of the latent space has been set to 2 in order to allow a 2D visualisation. Plots derived from both the optimisation of the JMVIB−T objective (Eq 8) and the optimisation of the JMVIB objective (Eq 5) are included. Five copies of all plots are available, as they are obtained by training the model with five different independent training-test random splits. (ZIP) [file pcbi.1010050.s007.zip › s5-file/bce-only/J/Early-Colorectal-EMBL/0_embeddings_95_confidence.png]

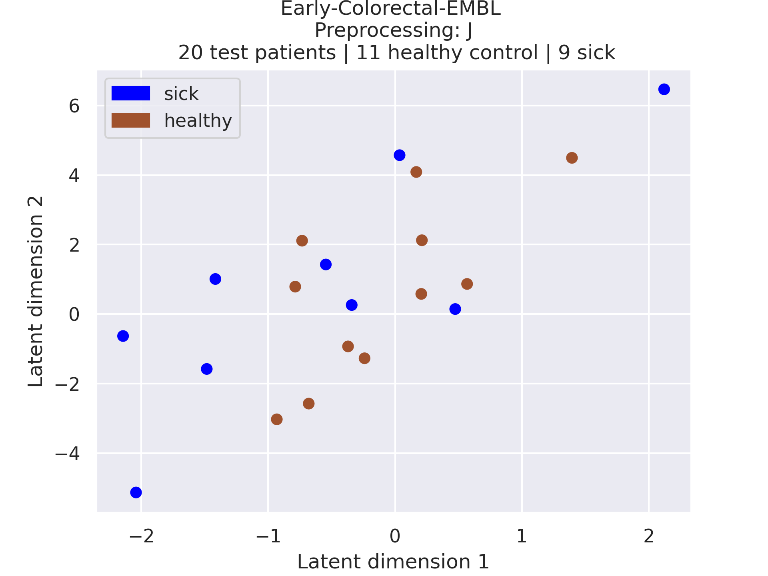

Supplement: S2 File — For all datasets considered in this work, this file presents plots of the 2D MVIB stochastic encodings analogous to Fig 3. The depicted curves are the 95% confidence intervals of the samples’ stochastic encodings z∼p(z|x)=N(μ,σ2I); the points are their means μ. The displayed encodings consist only in the test samples obtained from random training-test splits (i.e. the 20% of the dataset not used for training). The K dimension of the latent space has been set to 2 in order to allow a 2D visualisation. Plots derived from both the optimisation of the JMVIB−T objective (Eq 8) and the optimisation of the JMVIB objective (Eq 5) are included. Five copies of all plots are available, as they are obtained by training the model with five different independent training-test random splits. (ZIP) [file pcbi.1010050.s007.zip › s5-file/bce-only/J/Early-Colorectal-EMBL/1_embeddings.png]

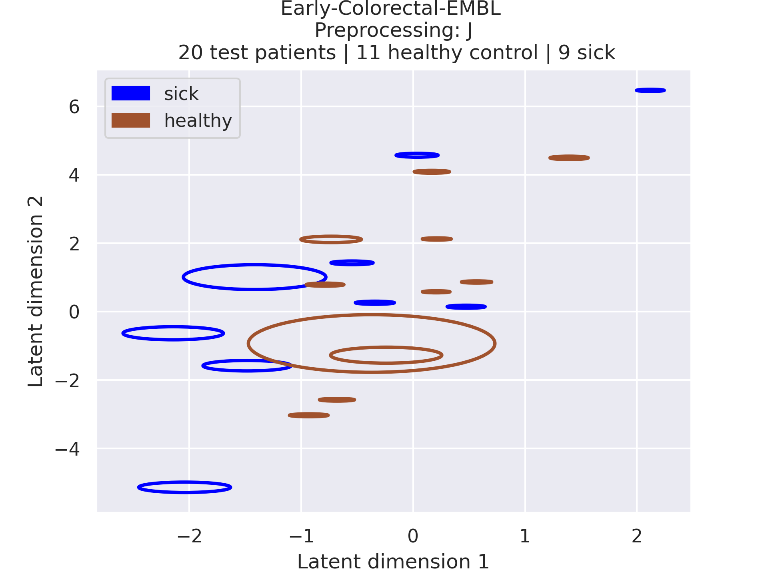

Supplement: S2 File — For all datasets considered in this work, this file presents plots of the 2D MVIB stochastic encodings analogous to Fig 3. The depicted curves are the 95% confidence intervals of the samples’ stochastic encodings z∼p(z|x)=N(μ,σ2I); the points are their means μ. The displayed encodings consist only in the test samples obtained from random training-test splits (i.e. the 20% of the dataset not used for training). The K dimension of the latent space has been set to 2 in order to allow a 2D visualisation. Plots derived from both the optimisation of the JMVIB−T objective (Eq 8) and the optimisation of the JMVIB objective (Eq 5) are included. Five copies of all plots are available, as they are obtained by training the model with five different independent training-test random splits. (ZIP) [file pcbi.1010050.s007.zip › s5-file/bce-only/J/Early-Colorectal-EMBL/1_embeddings_95_confidence.png]

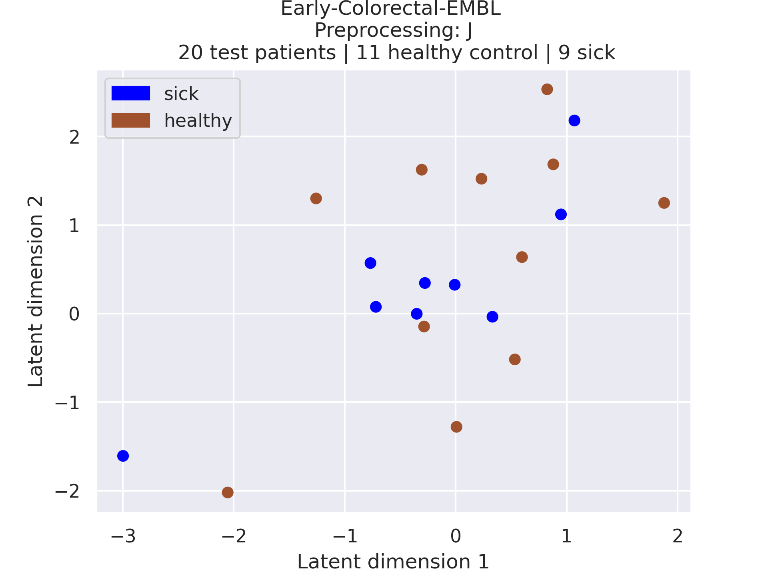

Supplement: S2 File — For all datasets considered in this work, this file presents plots of the 2D MVIB stochastic encodings analogous to Fig 3. The depicted curves are the 95% confidence intervals of the samples’ stochastic encodings z∼p(z|x)=N(μ,σ2I); the points are their means μ. The displayed encodings consist only in the test samples obtained from random training-test splits (i.e. the 20% of the dataset not used for training). The K dimension of the latent space has been set to 2 in order to allow a 2D visualisation. Plots derived from both the optimisation of the JMVIB−T objective (Eq 8) and the optimisation of the JMVIB objective (Eq 5) are included. Five copies of all plots are available, as they are obtained by training the model with five different independent training-test random splits. (ZIP) [file pcbi.1010050.s007.zip › s5-file/bce-only/J/Early-Colorectal-EMBL/2_embeddings.png]

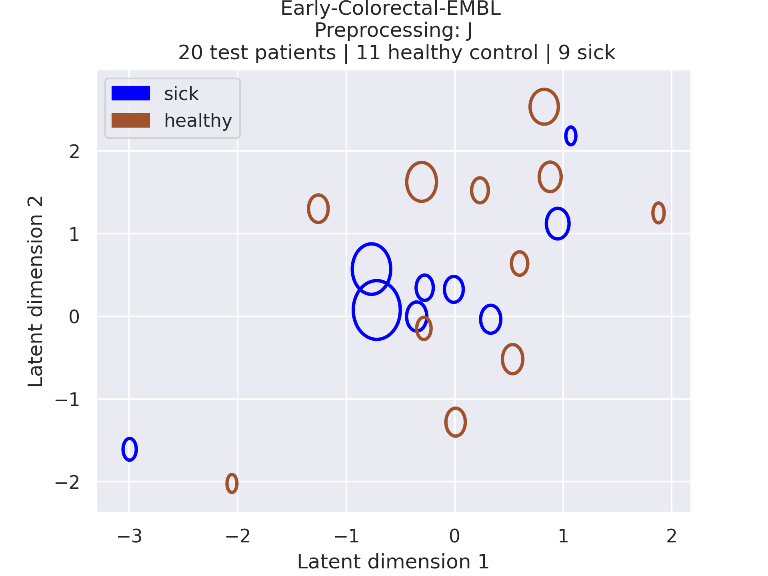

Supplement: S2 File — For all datasets considered in this work, this file presents plots of the 2D MVIB stochastic encodings analogous to Fig 3. The depicted curves are the 95% confidence intervals of the samples’ stochastic encodings z∼p(z|x)=N(μ,σ2I); the points are their means μ. The displayed encodings consist only in the test samples obtained from random training-test splits (i.e. the 20% of the dataset not used for training). The K dimension of the latent space has been set to 2 in order to allow a 2D visualisation. Plots derived from both the optimisation of the JMVIB−T objective (Eq 8) and the optimisation of the JMVIB objective (Eq 5) are included. Five copies of all plots are available, as they are obtained by training the model with five different independent training-test random splits. (ZIP) [file pcbi.1010050.s007.zip › s5-file/bce-only/J/Early-Colorectal-EMBL/2_embeddings_95_confidence.png]

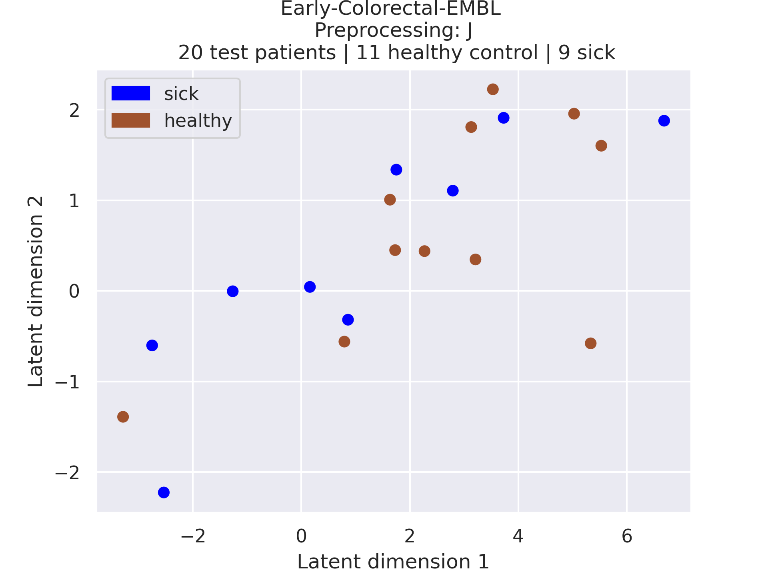

Supplement: S2 File — For all datasets considered in this work, this file presents plots of the 2D MVIB stochastic encodings analogous to Fig 3. The depicted curves are the 95% confidence intervals of the samples’ stochastic encodings z∼p(z|x)=N(μ,σ2I); the points are their means μ. The displayed encodings consist only in the test samples obtained from random training-test splits (i.e. the 20% of the dataset not used for training). The K dimension of the latent space has been set to 2 in order to allow a 2D visualisation. Plots derived from both the optimisation of the JMVIB−T objective (Eq 8) and the optimisation of the JMVIB objective (Eq 5) are included. Five copies of all plots are available, as they are obtained by training the model with five different independent training-test random splits. (ZIP) [file pcbi.1010050.s007.zip › s5-file/bce-only/J/Early-Colorectal-EMBL/3_embeddings.png]

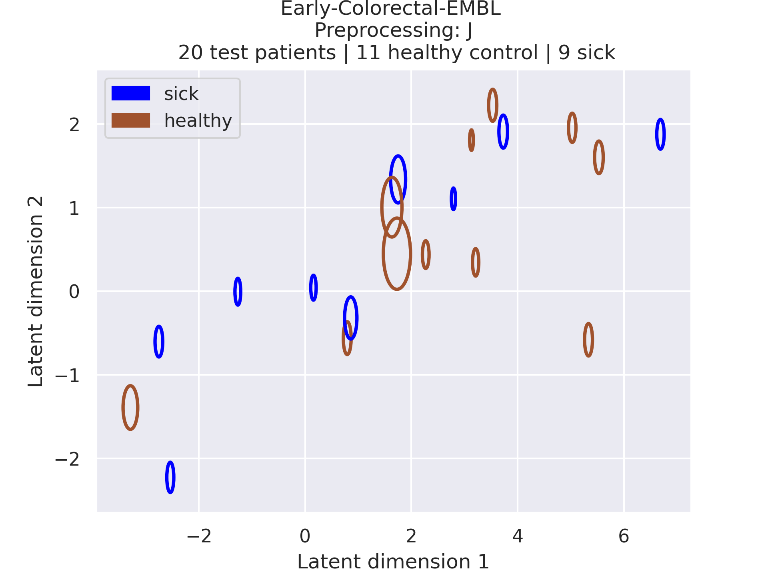

Supplement: S2 File — For all datasets considered in this work, this file presents plots of the 2D MVIB stochastic encodings analogous to Fig 3. The depicted curves are the 95% confidence intervals of the samples’ stochastic encodings z∼p(z|x)=N(μ,σ2I); the points are their means μ. The displayed encodings consist only in the test samples obtained from random training-test splits (i.e. the 20% of the dataset not used for training). The K dimension of the latent space has been set to 2 in order to allow a 2D visualisation. Plots derived from both the optimisation of the JMVIB−T objective (Eq 8) and the optimisation of the JMVIB objective (Eq 5) are included. Five copies of all plots are available, as they are obtained by training the model with five different independent training-test random splits. (ZIP) [file pcbi.1010050.s007.zip › s5-file/bce-only/J/Early-Colorectal-EMBL/3_embeddings_95_confidence.png]

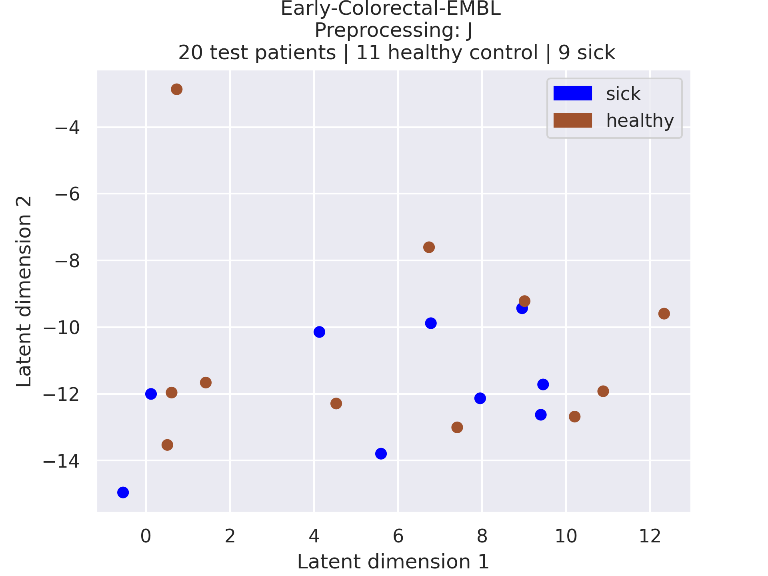

Supplement: S2 File — For all datasets considered in this work, this file presents plots of the 2D MVIB stochastic encodings analogous to Fig 3. The depicted curves are the 95% confidence intervals of the samples’ stochastic encodings z∼p(z|x)=N(μ,σ2I); the points are their means μ. The displayed encodings consist only in the test samples obtained from random training-test splits (i.e. the 20% of the dataset not used for training). The K dimension of the latent space has been set to 2 in order to allow a 2D visualisation. Plots derived from both the optimisation of the JMVIB−T objective (Eq 8) and the optimisation of the JMVIB objective (Eq 5) are included. Five copies of all plots are available, as they are obtained by training the model with five different independent training-test random splits. (ZIP) [file pcbi.1010050.s007.zip › s5-file/bce-only/J/Early-Colorectal-EMBL/4_embeddings.png]

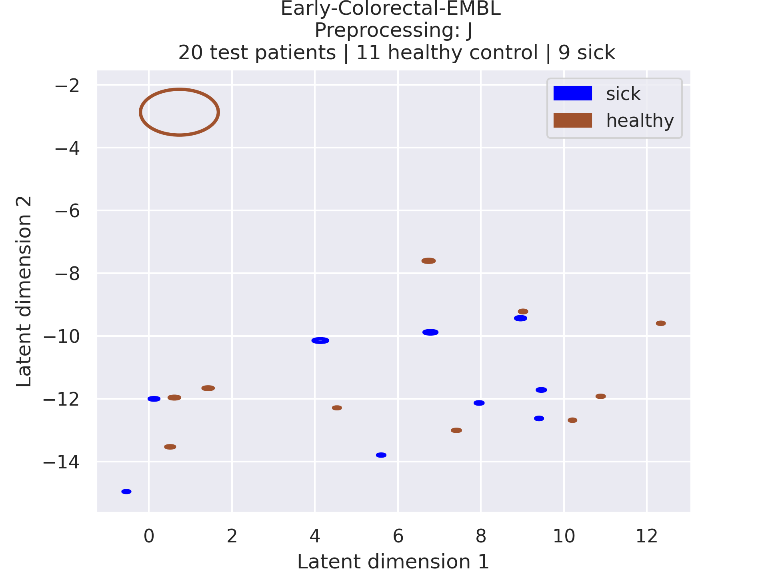

Supplement: S2 File — For all datasets considered in this work, this file presents plots of the 2D MVIB stochastic encodings analogous to Fig 3. The depicted curves are the 95% confidence intervals of the samples’ stochastic encodings z∼p(z|x)=N(μ,σ2I); the points are their means μ. The displayed encodings consist only in the test samples obtained from random training-test splits (i.e. the 20% of the dataset not used for training). The K dimension of the latent space has been set to 2 in order to allow a 2D visualisation. Plots derived from both the optimisation of the JMVIB−T objective (Eq 8) and the optimisation of the JMVIB objective (Eq 5) are included. Five copies of all plots are available, as they are obtained by training the model with five different independent training-test random splits. (ZIP) [file pcbi.1010050.s007.zip › s5-file/bce-only/J/Early-Colorectal-EMBL/4_embeddings_95_confidence.png]

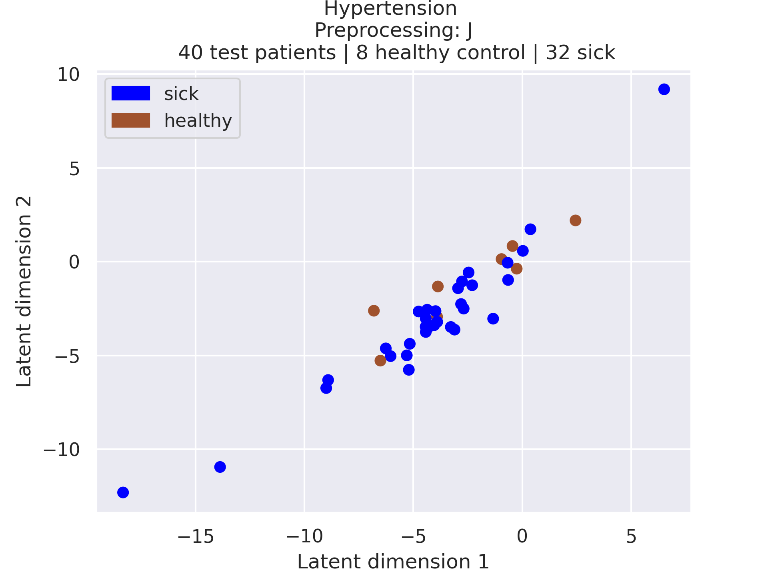

Supplement: S2 File — For all datasets considered in this work, this file presents plots of the 2D MVIB stochastic encodings analogous to Fig 3. The depicted curves are the 95% confidence intervals of the samples’ stochastic encodings z∼p(z|x)=N(μ,σ2I); the points are their means μ. The displayed encodings consist only in the test samples obtained from random training-test splits (i.e. the 20% of the dataset not used for training). The K dimension of the latent space has been set to 2 in order to allow a 2D visualisation. Plots derived from both the optimisation of the JMVIB−T objective (Eq 8) and the optimisation of the JMVIB objective (Eq 5) are included. Five copies of all plots are available, as they are obtained by training the model with five different independent training-test random splits. (ZIP) [file pcbi.1010050.s007.zip › s5-file/bce-only/J/Hypertension/0_embeddings.png]

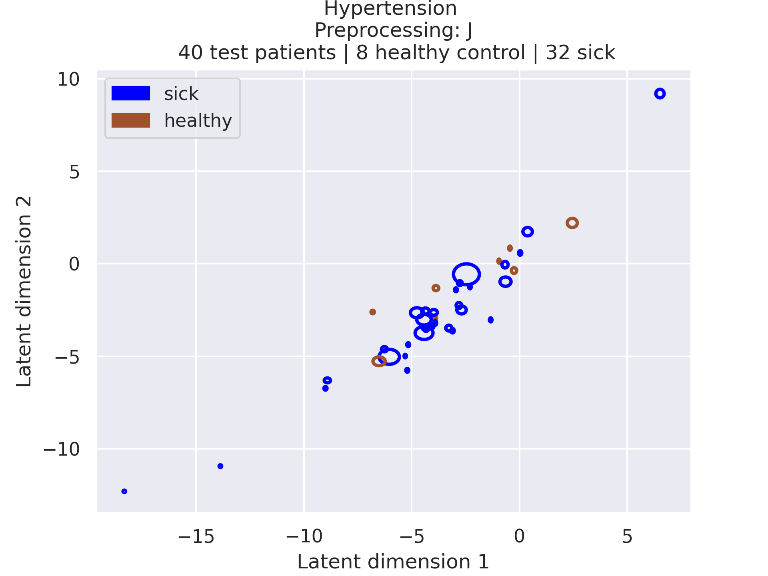

Supplement: S2 File — For all datasets considered in this work, this file presents plots of the 2D MVIB stochastic encodings analogous to Fig 3. The depicted curves are the 95% confidence intervals of the samples’ stochastic encodings z∼p(z|x)=N(μ,σ2I); the points are their means μ. The displayed encodings consist only in the test samples obtained from random training-test splits (i.e. the 20% of the dataset not used for training). The K dimension of the latent space has been set to 2 in order to allow a 2D visualisation. Plots derived from both the optimisation of the JMVIB−T objective (Eq 8) and the optimisation of the JMVIB objective (Eq 5) are included. Five copies of all plots are available, as they are obtained by training the model with five different independent training-test random splits. (ZIP) [file pcbi.1010050.s007.zip › s5-file/bce-only/J/Hypertension/0_embeddings_95_confidence.png]

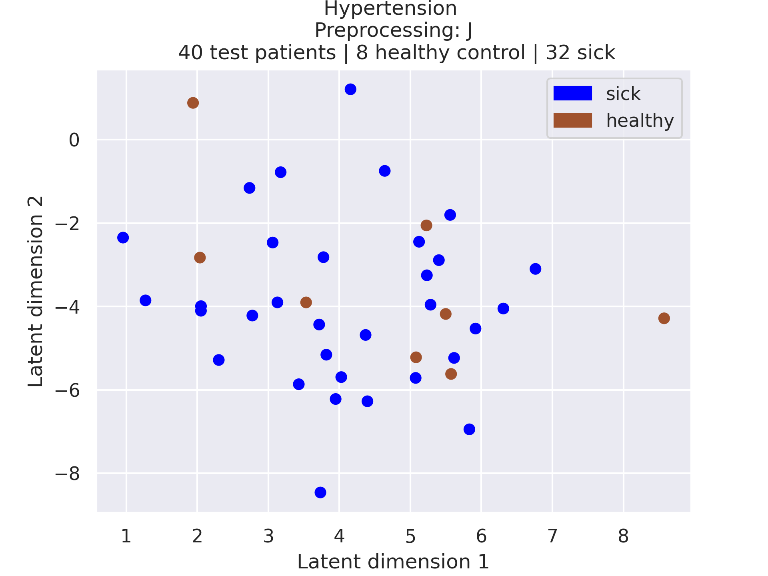

Supplement: S2 File — For all datasets considered in this work, this file presents plots of the 2D MVIB stochastic encodings analogous to Fig 3. The depicted curves are the 95% confidence intervals of the samples’ stochastic encodings z∼p(z|x)=N(μ,σ2I); the points are their means μ. The displayed encodings consist only in the test samples obtained from random training-test splits (i.e. the 20% of the dataset not used for training). The K dimension of the latent space has been set to 2 in order to allow a 2D visualisation. Plots derived from both the optimisation of the JMVIB−T objective (Eq 8) and the optimisation of the JMVIB objective (Eq 5) are included. Five copies of all plots are available, as they are obtained by training the model with five different independent training-test random splits. (ZIP) [file pcbi.1010050.s007.zip › s5-file/bce-only/J/Hypertension/1_embeddings.png]

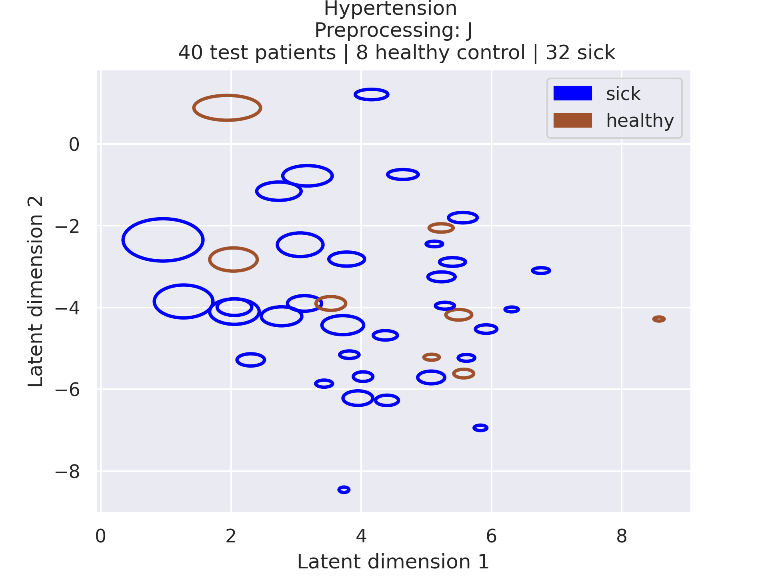

Supplement: S2 File — For all datasets considered in this work, this file presents plots of the 2D MVIB stochastic encodings analogous to Fig 3. The depicted curves are the 95% confidence intervals of the samples’ stochastic encodings z∼p(z|x)=N(μ,σ2I); the points are their means μ. The displayed encodings consist only in the test samples obtained from random training-test splits (i.e. the 20% of the dataset not used for training). The K dimension of the latent space has been set to 2 in order to allow a 2D visualisation. Plots derived from both the optimisation of the JMVIB−T objective (Eq 8) and the optimisation of the JMVIB objective (Eq 5) are included. Five copies of all plots are available, as they are obtained by training the model with five different independent training-test random splits. (ZIP) [file pcbi.1010050.s007.zip › s5-file/bce-only/J/Hypertension/1_embeddings_95_confidence.png]

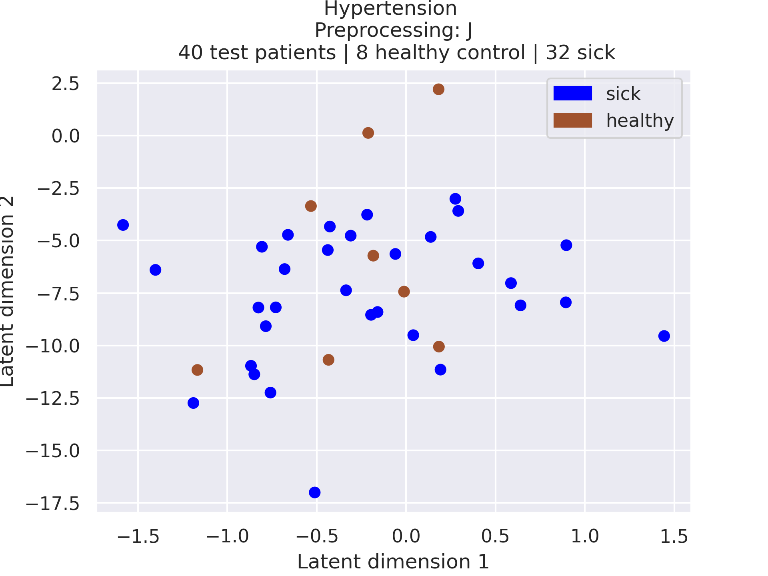

Supplement: S2 File — For all datasets considered in this work, this file presents plots of the 2D MVIB stochastic encodings analogous to Fig 3. The depicted curves are the 95% confidence intervals of the samples’ stochastic encodings z∼p(z|x)=N(μ,σ2I); the points are their means μ. The displayed encodings consist only in the test samples obtained from random training-test splits (i.e. the 20% of the dataset not used for training). The K dimension of the latent space has been set to 2 in order to allow a 2D visualisation. Plots derived from both the optimisation of the JMVIB−T objective (Eq 8) and the optimisation of the JMVIB objective (Eq 5) are included. Five copies of all plots are available, as they are obtained by training the model with five different independent training-test random splits. (ZIP) [file pcbi.1010050.s007.zip › s5-file/bce-only/J/Hypertension/2_embeddings.png]

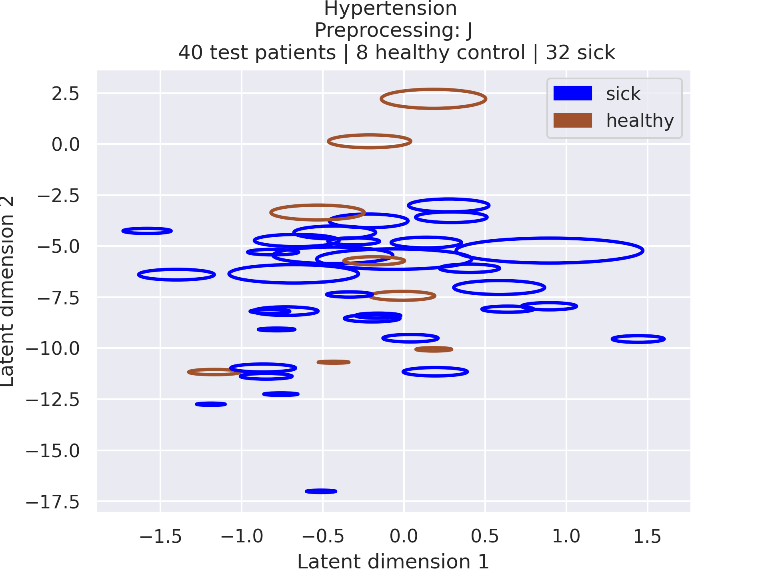

Supplement: S2 File — For all datasets considered in this work, this file presents plots of the 2D MVIB stochastic encodings analogous to Fig 3. The depicted curves are the 95% confidence intervals of the samples’ stochastic encodings z∼p(z|x)=N(μ,σ2I); the points are their means μ. The displayed encodings consist only in the test samples obtained from random training-test splits (i.e. the 20% of the dataset not used for training). The K dimension of the latent space has been set to 2 in order to allow a 2D visualisation. Plots derived from both the optimisation of the JMVIB−T objective (Eq 8) and the optimisation of the JMVIB objective (Eq 5) are included. Five copies of all plots are available, as they are obtained by training the model with five different independent training-test random splits. (ZIP) [file pcbi.1010050.s007.zip › s5-file/bce-only/J/Hypertension/2_embeddings_95_confidence.png]

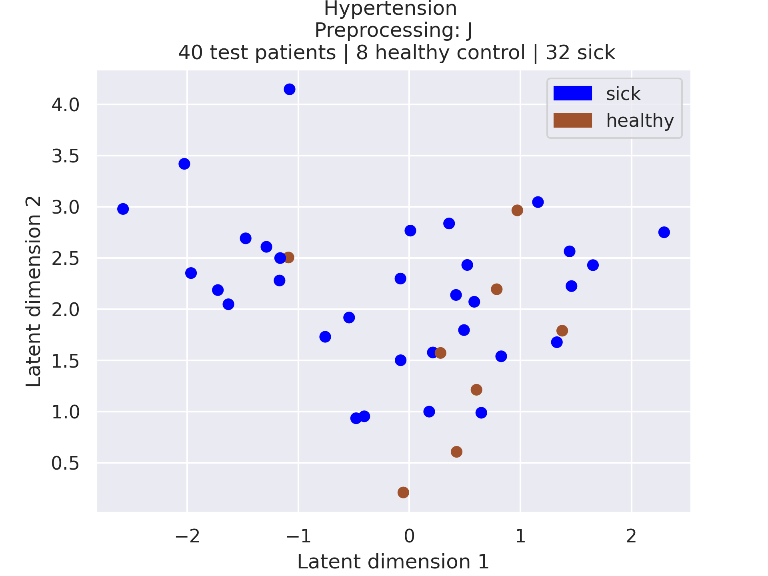

Supplement: S2 File — For all datasets considered in this work, this file presents plots of the 2D MVIB stochastic encodings analogous to Fig 3. The depicted curves are the 95% confidence intervals of the samples’ stochastic encodings z∼p(z|x)=N(μ,σ2I); the points are their means μ. The displayed encodings consist only in the test samples obtained from random training-test splits (i.e. the 20% of the dataset not used for training). The K dimension of the latent space has been set to 2 in order to allow a 2D visualisation. Plots derived from both the optimisation of the JMVIB−T objective (Eq 8) and the optimisation of the JMVIB objective (Eq 5) are included. Five copies of all plots are available, as they are obtained by training the model with five different independent training-test random splits. (ZIP) [file pcbi.1010050.s007.zip › s5-file/bce-only/J/Hypertension/3_embeddings.png]

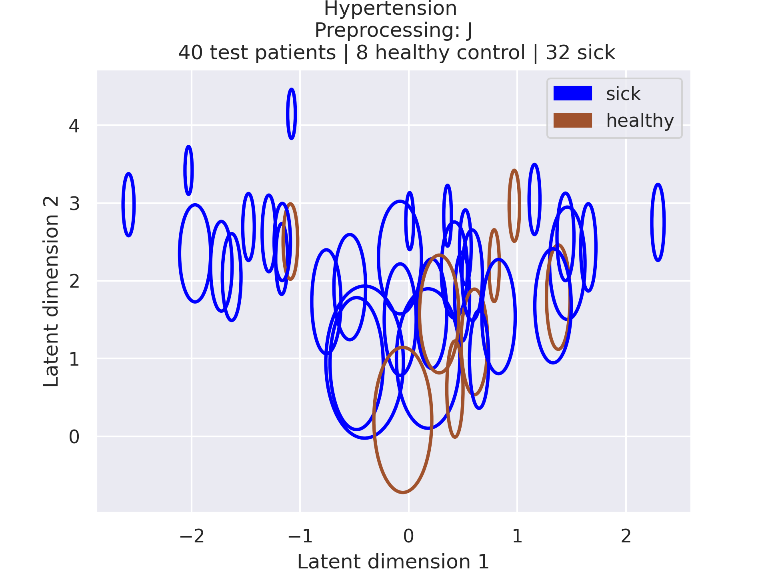

Supplement: S2 File — For all datasets considered in this work, this file presents plots of the 2D MVIB stochastic encodings analogous to Fig 3. The depicted curves are the 95% confidence intervals of the samples’ stochastic encodings z∼p(z|x)=N(μ,σ2I); the points are their means μ. The displayed encodings consist only in the test samples obtained from random training-test splits (i.e. the 20% of the dataset not used for training). The K dimension of the latent space has been set to 2 in order to allow a 2D visualisation. Plots derived from both the optimisation of the JMVIB−T objective (Eq 8) and the optimisation of the JMVIB objective (Eq 5) are included. Five copies of all plots are available, as they are obtained by training the model with five different independent training-test random splits. (ZIP) [file pcbi.1010050.s007.zip › s5-file/bce-only/J/Hypertension/3_embeddings_95_confidence.png]

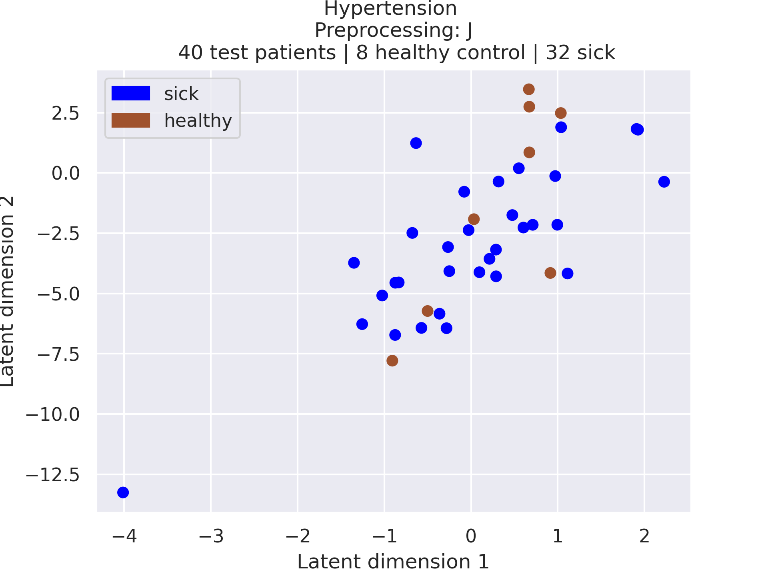

Supplement: S2 File — For all datasets considered in this work, this file presents plots of the 2D MVIB stochastic encodings analogous to Fig 3. The depicted curves are the 95% confidence intervals of the samples’ stochastic encodings z∼p(z|x)=N(μ,σ2I); the points are their means μ. The displayed encodings consist only in the test samples obtained from random training-test splits (i.e. the 20% of the dataset not used for training). The K dimension of the latent space has been set to 2 in order to allow a 2D visualisation. Plots derived from both the optimisation of the JMVIB−T objective (Eq 8) and the optimisation of the JMVIB objective (Eq 5) are included. Five copies of all plots are available, as they are obtained by training the model with five different independent training-test random splits. (ZIP) [file pcbi.1010050.s007.zip › s5-file/bce-only/J/Hypertension/4_embeddings.png]

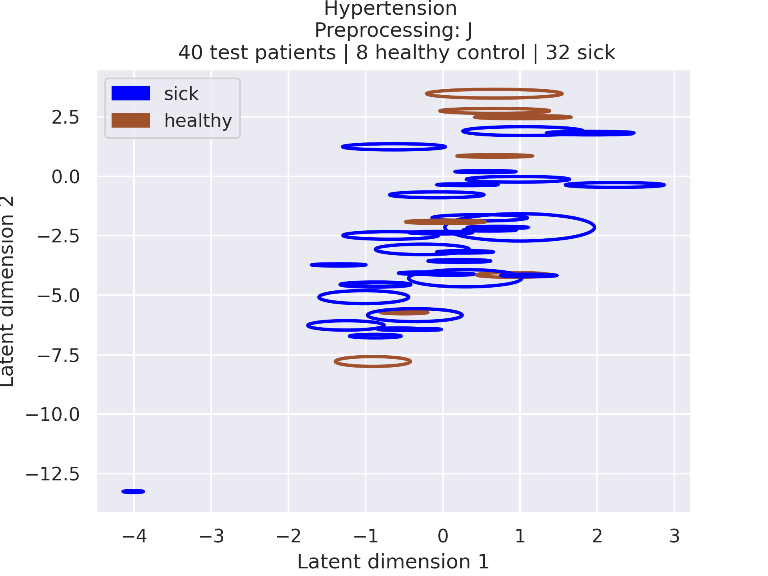

Supplement: S2 File — For all datasets considered in this work, this file presents plots of the 2D MVIB stochastic encodings analogous to Fig 3. The depicted curves are the 95% confidence intervals of the samples’ stochastic encodings z∼p(z|x)=N(μ,σ2I); the points are their means μ. The displayed encodings consist only in the test samples obtained from random training-test splits (i.e. the 20% of the dataset not used for training). The K dimension of the latent space has been set to 2 in order to allow a 2D visualisation. Plots derived from both the optimisation of the JMVIB−T objective (Eq 8) and the optimisation of the JMVIB objective (Eq 5) are included. Five copies of all plots are available, as they are obtained by training the model with five different independent training-test random splits. (ZIP) [file pcbi.1010050.s007.zip › s5-file/bce-only/J/Hypertension/4_embeddings_95_confidence.png]

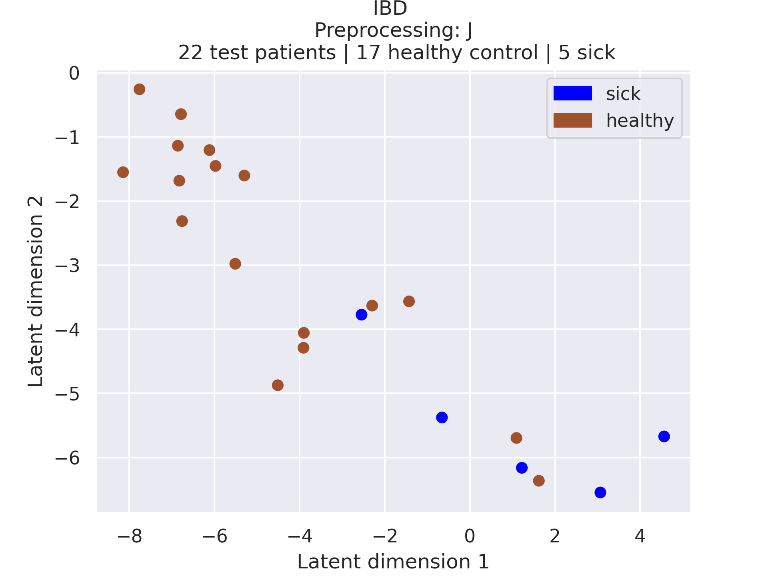

Supplement: S2 File — For all datasets considered in this work, this file presents plots of the 2D MVIB stochastic encodings analogous to Fig 3. The depicted curves are the 95% confidence intervals of the samples’ stochastic encodings z∼p(z|x)=N(μ,σ2I); the points are their means μ. The displayed encodings consist only in the test samples obtained from random training-test splits (i.e. the 20% of the dataset not used for training). The K dimension of the latent space has been set to 2 in order to allow a 2D visualisation. Plots derived from both the optimisation of the JMVIB−T objective (Eq 8) and the optimisation of the JMVIB objective (Eq 5) are included. Five copies of all plots are available, as they are obtained by training the model with five different independent training-test random splits. (ZIP) [file pcbi.1010050.s007.zip › s5-file/bce-only/J/IBD/0_embeddings.png]

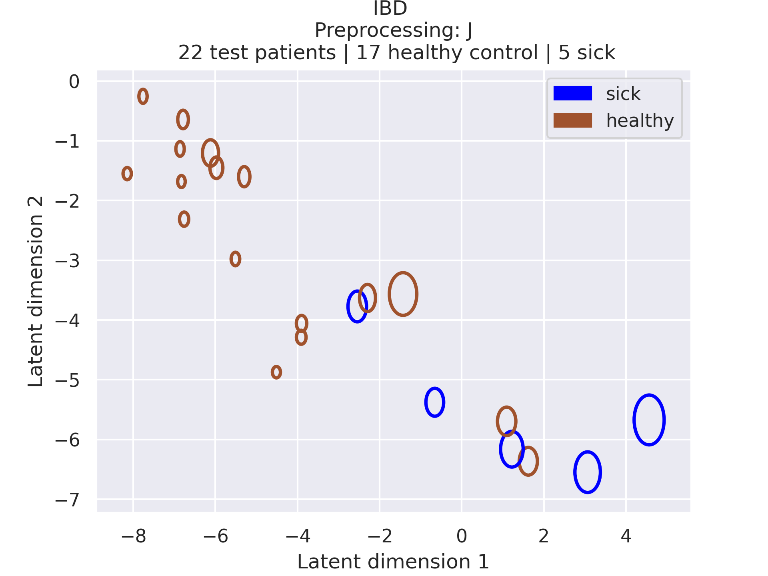

Supplement: S2 File — For all datasets considered in this work, this file presents plots of the 2D MVIB stochastic encodings analogous to Fig 3. The depicted curves are the 95% confidence intervals of the samples’ stochastic encodings z∼p(z|x)=N(μ,σ2I); the points are their means μ. The displayed encodings consist only in the test samples obtained from random training-test splits (i.e. the 20% of the dataset not used for training). The K dimension of the latent space has been set to 2 in order to allow a 2D visualisation. Plots derived from both the optimisation of the JMVIB−T objective (Eq 8) and the optimisation of the JMVIB objective (Eq 5) are included. Five copies of all plots are available, as they are obtained by training the model with five different independent training-test random splits. (ZIP) [file pcbi.1010050.s007.zip › s5-file/bce-only/J/IBD/0_embeddings_95_confidence.png]

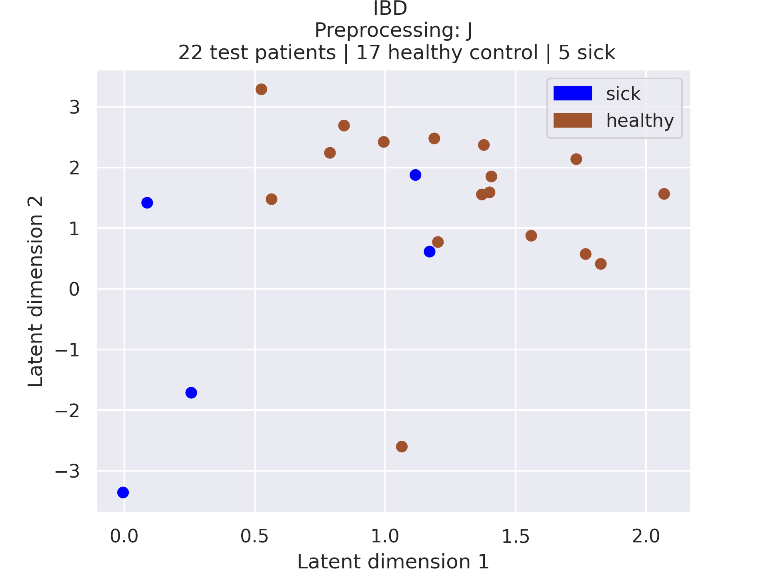

Supplement: S2 File — For all datasets considered in this work, this file presents plots of the 2D MVIB stochastic encodings analogous to Fig 3. The depicted curves are the 95% confidence intervals of the samples’ stochastic encodings z∼p(z|x)=N(μ,σ2I); the points are their means μ. The displayed encodings consist only in the test samples obtained from random training-test splits (i.e. the 20% of the dataset not used for training). The K dimension of the latent space has been set to 2 in order to allow a 2D visualisation. Plots derived from both the optimisation of the JMVIB−T objective (Eq 8) and the optimisation of the JMVIB objective (Eq 5) are included. Five copies of all plots are available, as they are obtained by training the model with five different independent training-test random splits. (ZIP) [file pcbi.1010050.s007.zip › s5-file/bce-only/J/IBD/1_embeddings.png]

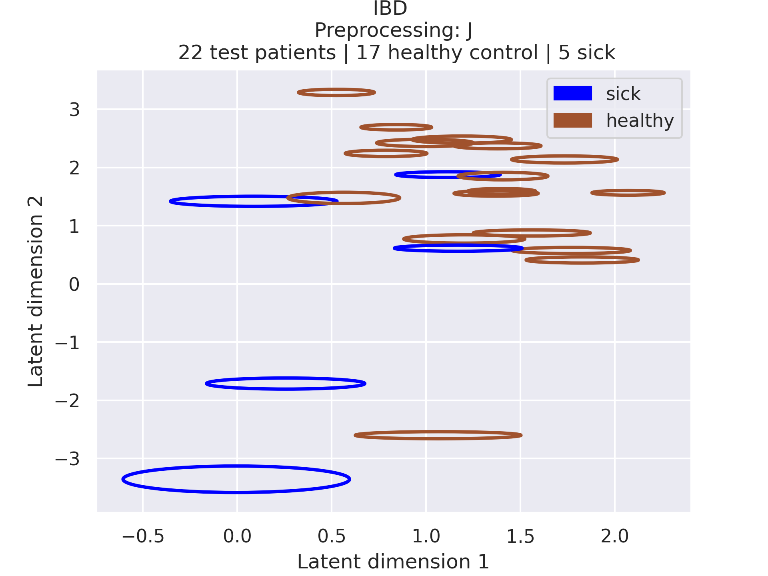

Supplement: S2 File — For all datasets considered in this work, this file presents plots of the 2D MVIB stochastic encodings analogous to Fig 3. The depicted curves are the 95% confidence intervals of the samples’ stochastic encodings z∼p(z|x)=N(μ,σ2I); the points are their means μ. The displayed encodings consist only in the test samples obtained from random training-test splits (i.e. the 20% of the dataset not used for training). The K dimension of the latent space has been set to 2 in order to allow a 2D visualisation. Plots derived from both the optimisation of the JMVIB−T objective (Eq 8) and the optimisation of the JMVIB objective (Eq 5) are included. Five copies of all plots are available, as they are obtained by training the model with five different independent training-test random splits. (ZIP) [file pcbi.1010050.s007.zip › s5-file/bce-only/J/IBD/1_embeddings_95_confidence.png]

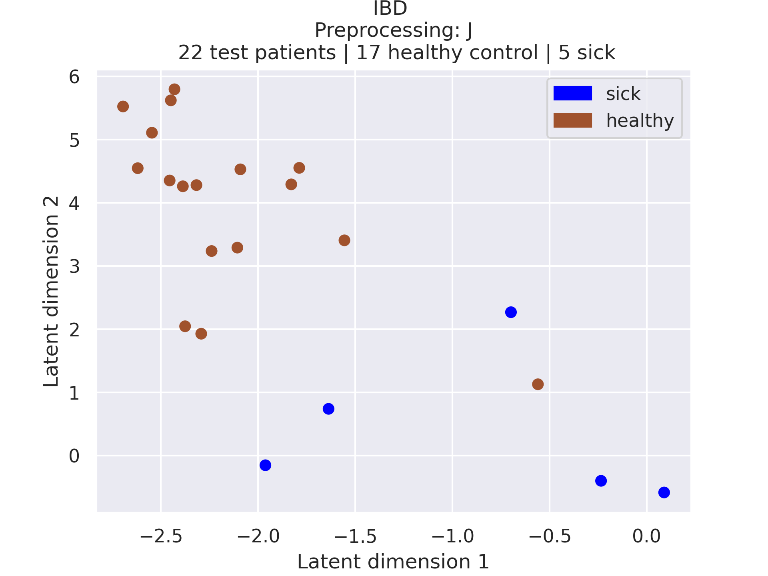

Supplement: S2 File — For all datasets considered in this work, this file presents plots of the 2D MVIB stochastic encodings analogous to Fig 3. The depicted curves are the 95% confidence intervals of the samples’ stochastic encodings z∼p(z|x)=N(μ,σ2I); the points are their means μ. The displayed encodings consist only in the test samples obtained from random training-test splits (i.e. the 20% of the dataset not used for training). The K dimension of the latent space has been set to 2 in order to allow a 2D visualisation. Plots derived from both the optimisation of the JMVIB−T objective (Eq 8) and the optimisation of the JMVIB objective (Eq 5) are included. Five copies of all plots are available, as they are obtained by training the model with five different independent training-test random splits. (ZIP) [file pcbi.1010050.s007.zip › s5-file/bce-only/J/IBD/2_embeddings.png]

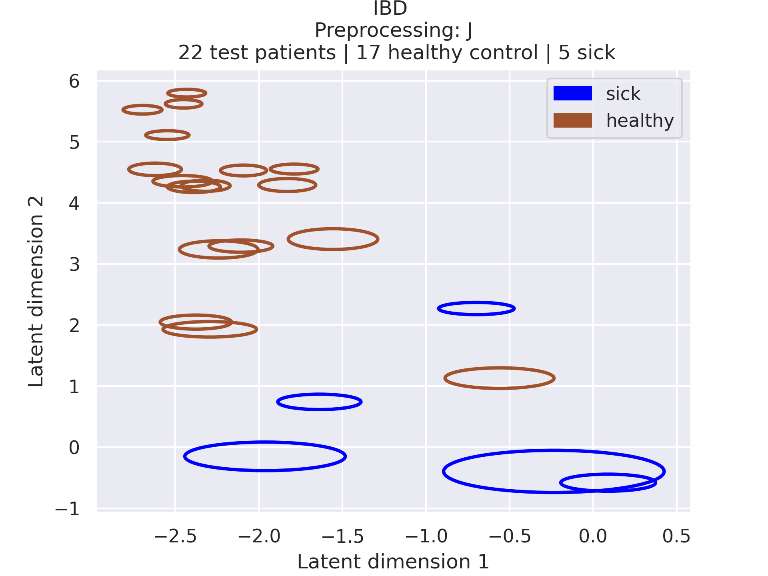

Supplement: S2 File — For all datasets considered in this work, this file presents plots of the 2D MVIB stochastic encodings analogous to Fig 3. The depicted curves are the 95% confidence intervals of the samples’ stochastic encodings z∼p(z|x)=N(μ,σ2I); the points are their means μ. The displayed encodings consist only in the test samples obtained from random training-test splits (i.e. the 20% of the dataset not used for training). The K dimension of the latent space has been set to 2 in order to allow a 2D visualisation. Plots derived from both the optimisation of the JMVIB−T objective (Eq 8) and the optimisation of the JMVIB objective (Eq 5) are included. Five copies of all plots are available, as they are obtained by training the model with five different independent training-test random splits. (ZIP) [file pcbi.1010050.s007.zip › s5-file/bce-only/J/IBD/2_embeddings_95_confidence.png]

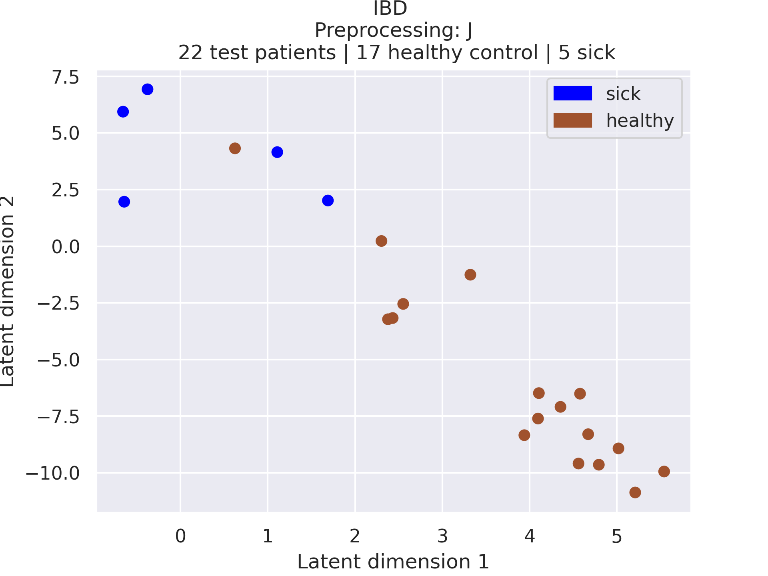

Supplement: S2 File — For all datasets considered in this work, this file presents plots of the 2D MVIB stochastic encodings analogous to Fig 3. The depicted curves are the 95% confidence intervals of the samples’ stochastic encodings z∼p(z|x)=N(μ,σ2I); the points are their means μ. The displayed encodings consist only in the test samples obtained from random training-test splits (i.e. the 20% of the dataset not used for training). The K dimension of the latent space has been set to 2 in order to allow a 2D visualisation. Plots derived from both the optimisation of the JMVIB−T objective (Eq 8) and the optimisation of the JMVIB objective (Eq 5) are included. Five copies of all plots are available, as they are obtained by training the model with five different independent training-test random splits. (ZIP) [file pcbi.1010050.s007.zip › s5-file/bce-only/J/IBD/3_embeddings.png]

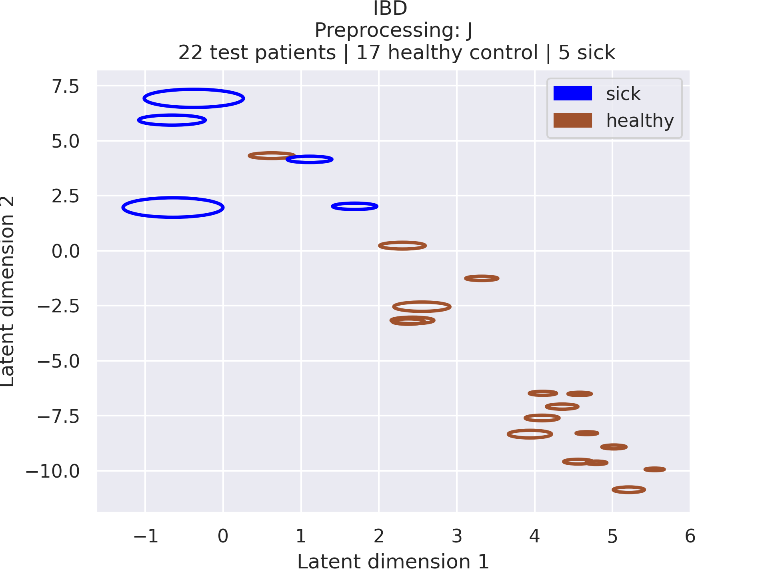

Supplement: S2 File — For all datasets considered in this work, this file presents plots of the 2D MVIB stochastic encodings analogous to Fig 3. The depicted curves are the 95% confidence intervals of the samples’ stochastic encodings z∼p(z|x)=N(μ,σ2I); the points are their means μ. The displayed encodings consist only in the test samples obtained from random training-test splits (i.e. the 20% of the dataset not used for training). The K dimension of the latent space has been set to 2 in order to allow a 2D visualisation. Plots derived from both the optimisation of the JMVIB−T objective (Eq 8) and the optimisation of the JMVIB objective (Eq 5) are included. Five copies of all plots are available, as they are obtained by training the model with five different independent training-test random splits. (ZIP) [file pcbi.1010050.s007.zip › s5-file/bce-only/J/IBD/3_embeddings_95_confidence.png]

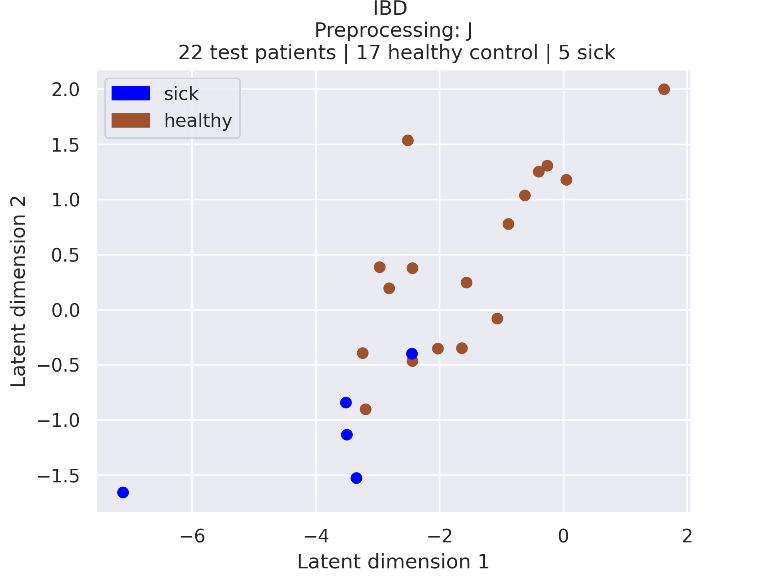

Supplement: S2 File — For all datasets considered in this work, this file presents plots of the 2D MVIB stochastic encodings analogous to Fig 3. The depicted curves are the 95% confidence intervals of the samples’ stochastic encodings z∼p(z|x)=N(μ,σ2I); the points are their means μ. The displayed encodings consist only in the test samples obtained from random training-test splits (i.e. the 20% of the dataset not used for training). The K dimension of the latent space has been set to 2 in order to allow a 2D visualisation. Plots derived from both the optimisation of the JMVIB−T objective (Eq 8) and the optimisation of the JMVIB objective (Eq 5) are included. Five copies of all plots are available, as they are obtained by training the model with five different independent training-test random splits. (ZIP) [file pcbi.1010050.s007.zip › s5-file/bce-only/J/IBD/4_embeddings.png]
