# Supplementary figures and images for: Microbiome-based disease prediction with multimodal variational information bottlenecks (part 4 of 4)
Source: PLoS Comput Biol. 2022 Apr 11;18(4):e1010050. doi: 10.1371/journal.pcbi.1010050 (PMC9022840; doi:10.1371/journal.pcbi.1010050)

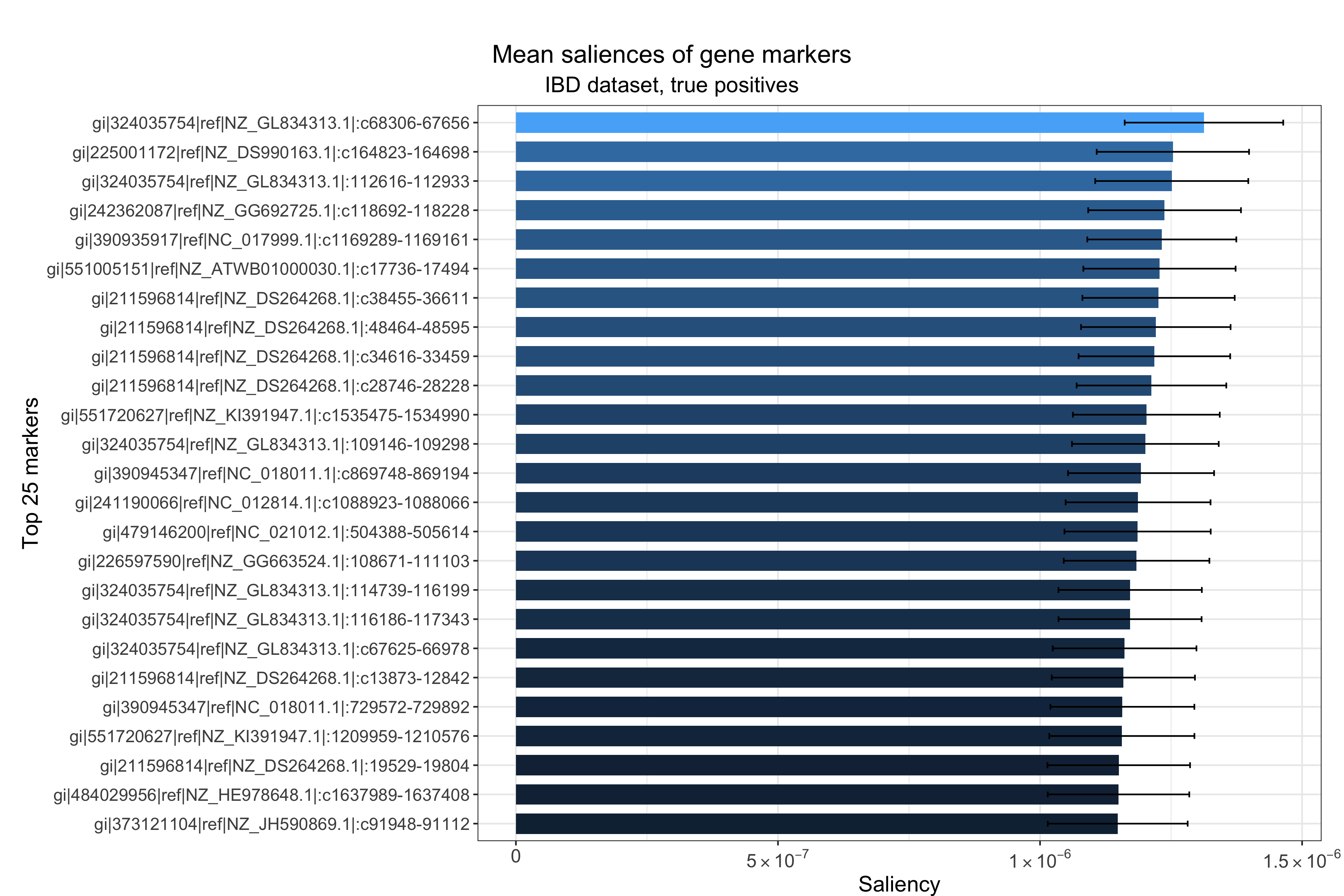

Supplement: S4 File — These files present the plots of the top 25 microbial species and strain markers for all datasets considered in this work, analogous to what Fig 5A depicts for the species from the Colorectal-EMBL dataset. Additionally, the scripts used to create the plots are included. (ZIP) [file pcbi.1010050.s009.zip › s8-file/IBD/markers_errbarplot_TP_saliences_no_rescale.png]

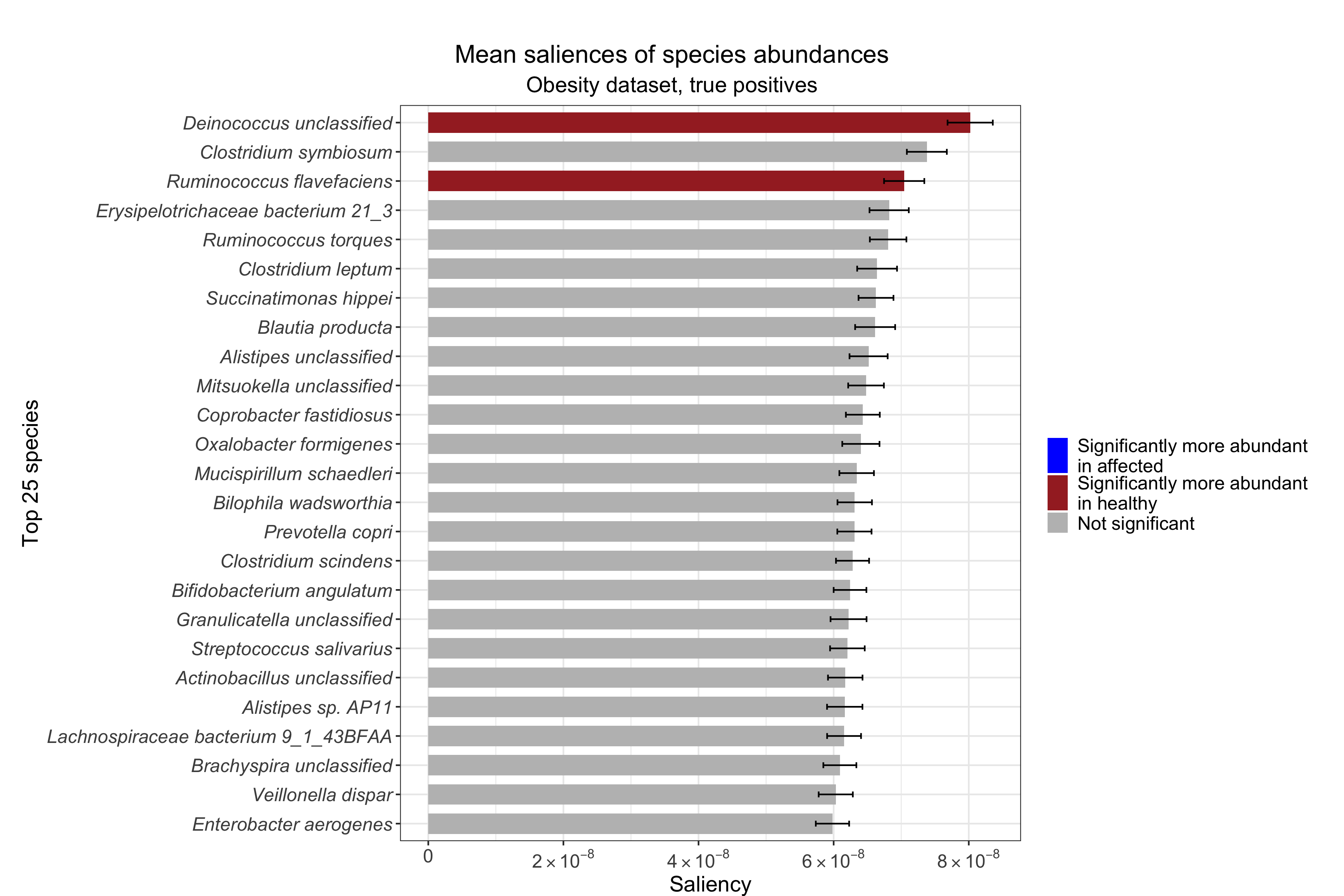

Supplement: S4 File — These files present the plots of the top 25 microbial species and strain markers for all datasets considered in this work, analogous to what Fig 5A depicts for the species from the Colorectal-EMBL dataset. Additionally, the scripts used to create the plots are included. (ZIP) [file pcbi.1010050.s009.zip › s8-file/Obesity/abundance_errbarplot_TP_saliences_no_rescale_pval-0.1.png]

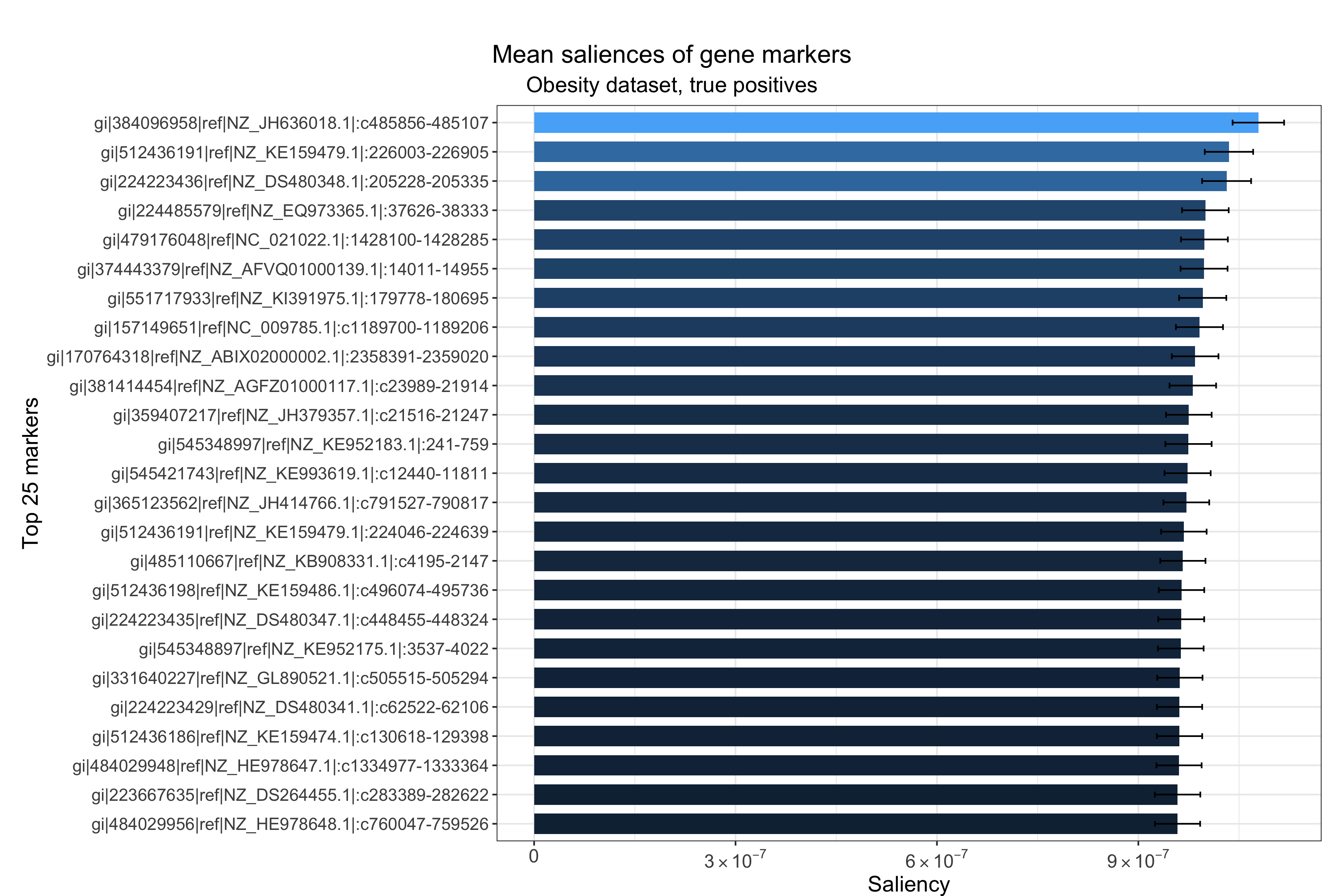

Supplement: S4 File — These files present the plots of the top 25 microbial species and strain markers for all datasets considered in this work, analogous to what Fig 5A depicts for the species from the Colorectal-EMBL dataset. Additionally, the scripts used to create the plots are included. (ZIP) [file pcbi.1010050.s009.zip › s8-file/Obesity/markers_errbarplot_TP_saliences_no_rescale.png]

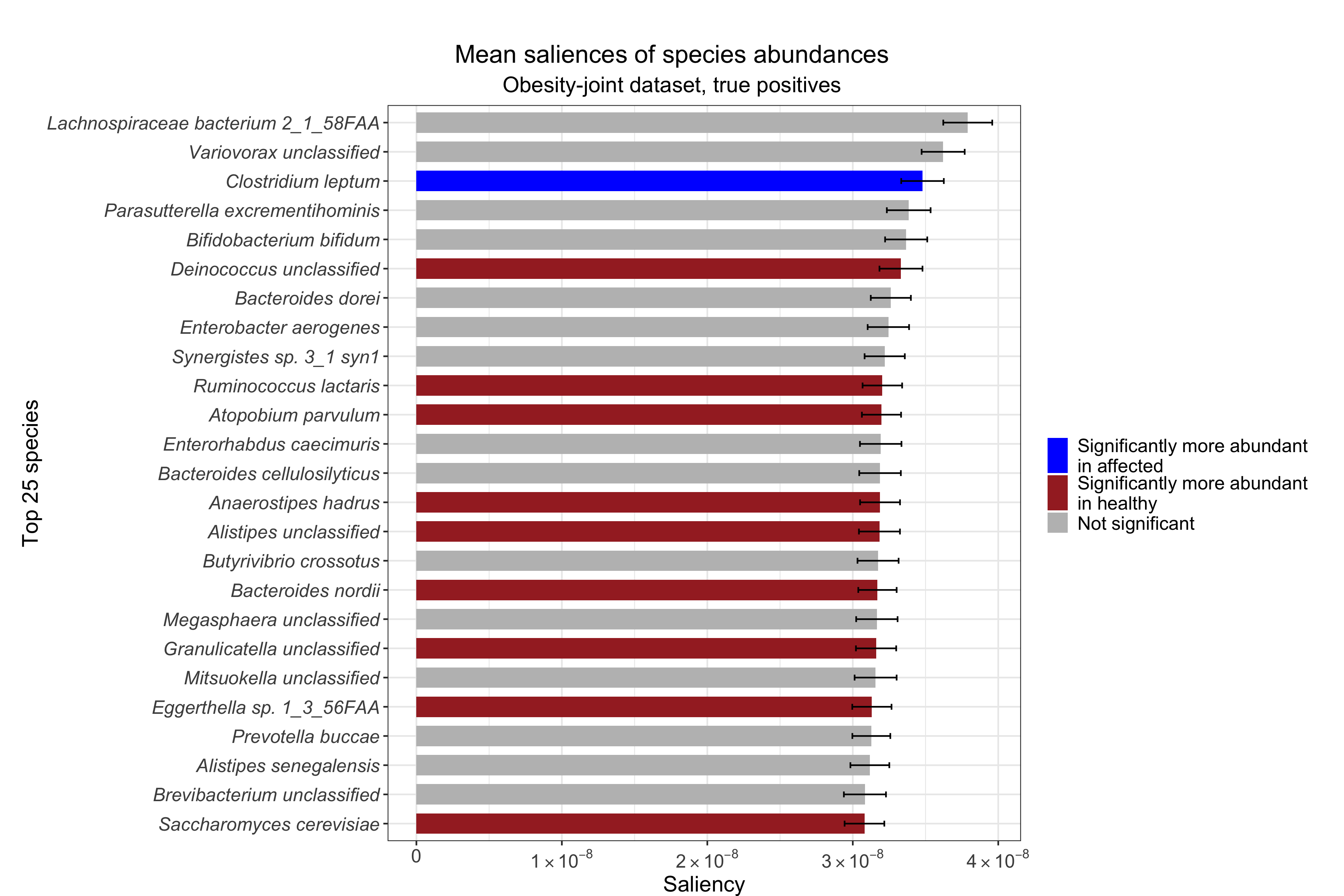

Supplement: S4 File — These files present the plots of the top 25 microbial species and strain markers for all datasets considered in this work, analogous to what Fig 5A depicts for the species from the Colorectal-EMBL dataset. Additionally, the scripts used to create the plots are included. (ZIP) [file pcbi.1010050.s009.zip › s8-file/Obesity-joint/abundance_errbarplot_TP_saliences_no_rescale_pval-0.1.png]

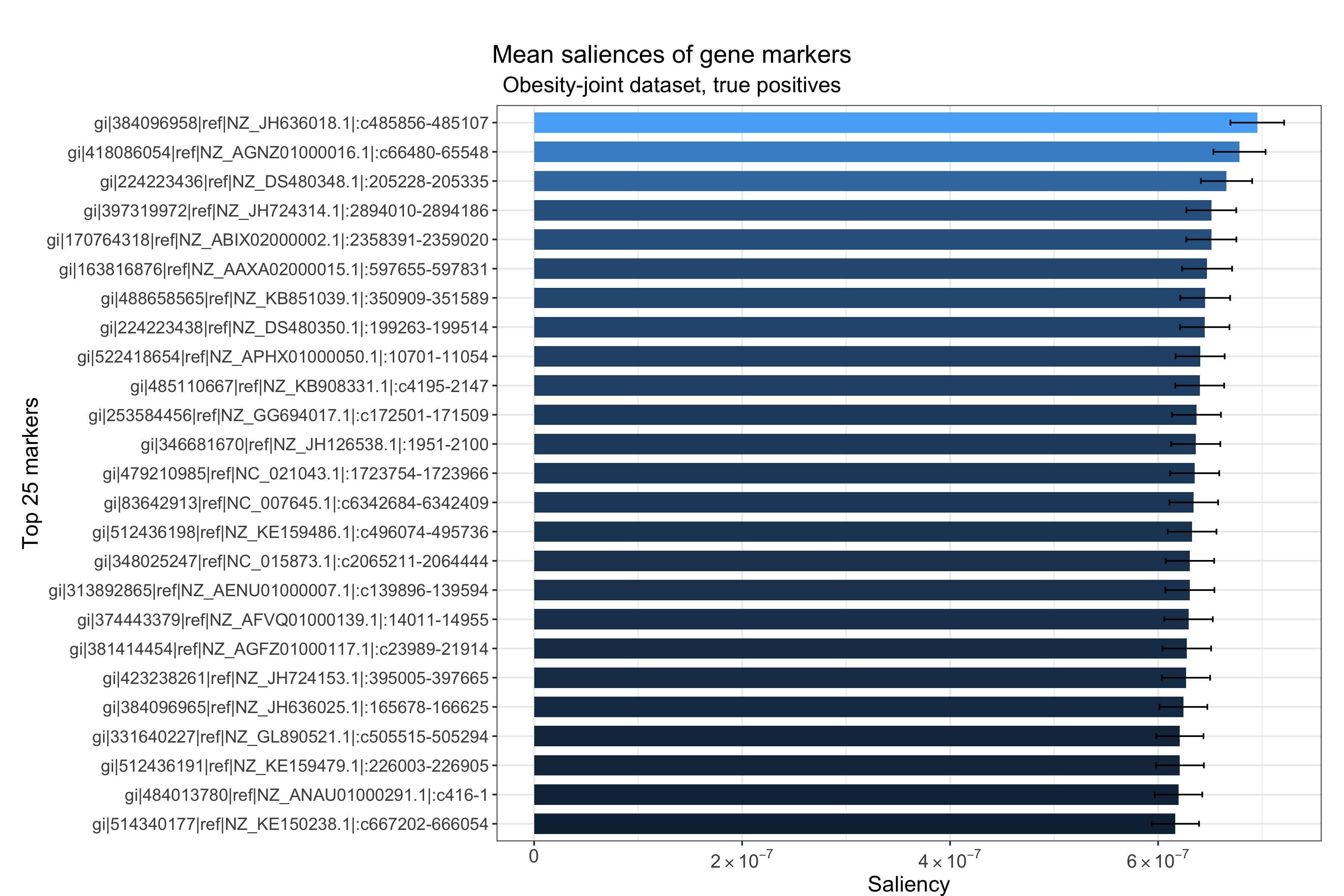

Supplement: S4 File — These files present the plots of the top 25 microbial species and strain markers for all datasets considered in this work, analogous to what Fig 5A depicts for the species from the Colorectal-EMBL dataset. Additionally, the scripts used to create the plots are included. (ZIP) [file pcbi.1010050.s009.zip › s8-file/Obesity-joint/markers_errbarplot_TP_saliences_no_rescale.png]

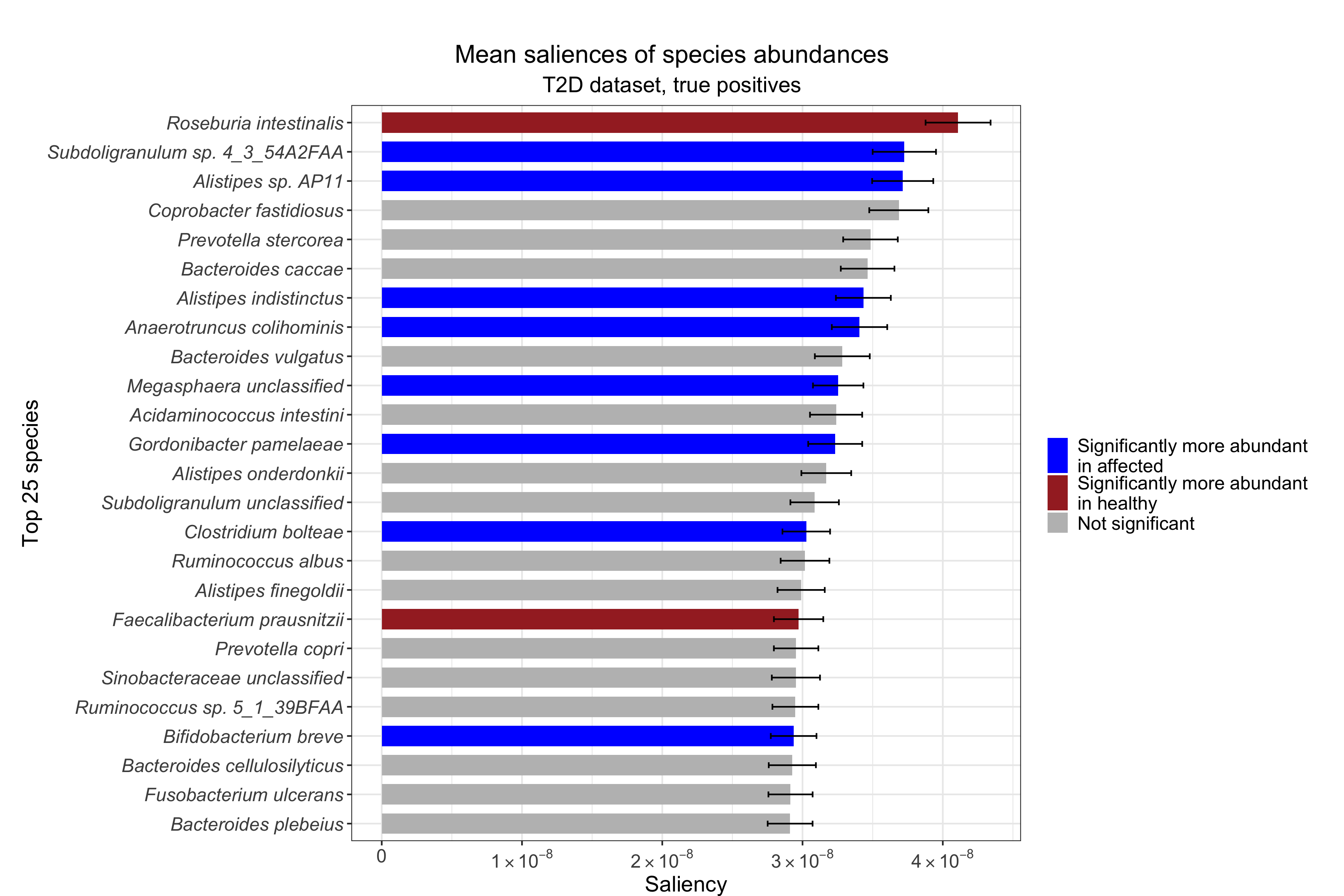

Supplement: S4 File — These files present the plots of the top 25 microbial species and strain markers for all datasets considered in this work, analogous to what Fig 5A depicts for the species from the Colorectal-EMBL dataset. Additionally, the scripts used to create the plots are included. (ZIP) [file pcbi.1010050.s009.zip › s8-file/T2D/abundance_errbarplot_TP_saliences_no_rescale_pval-0.1.png]

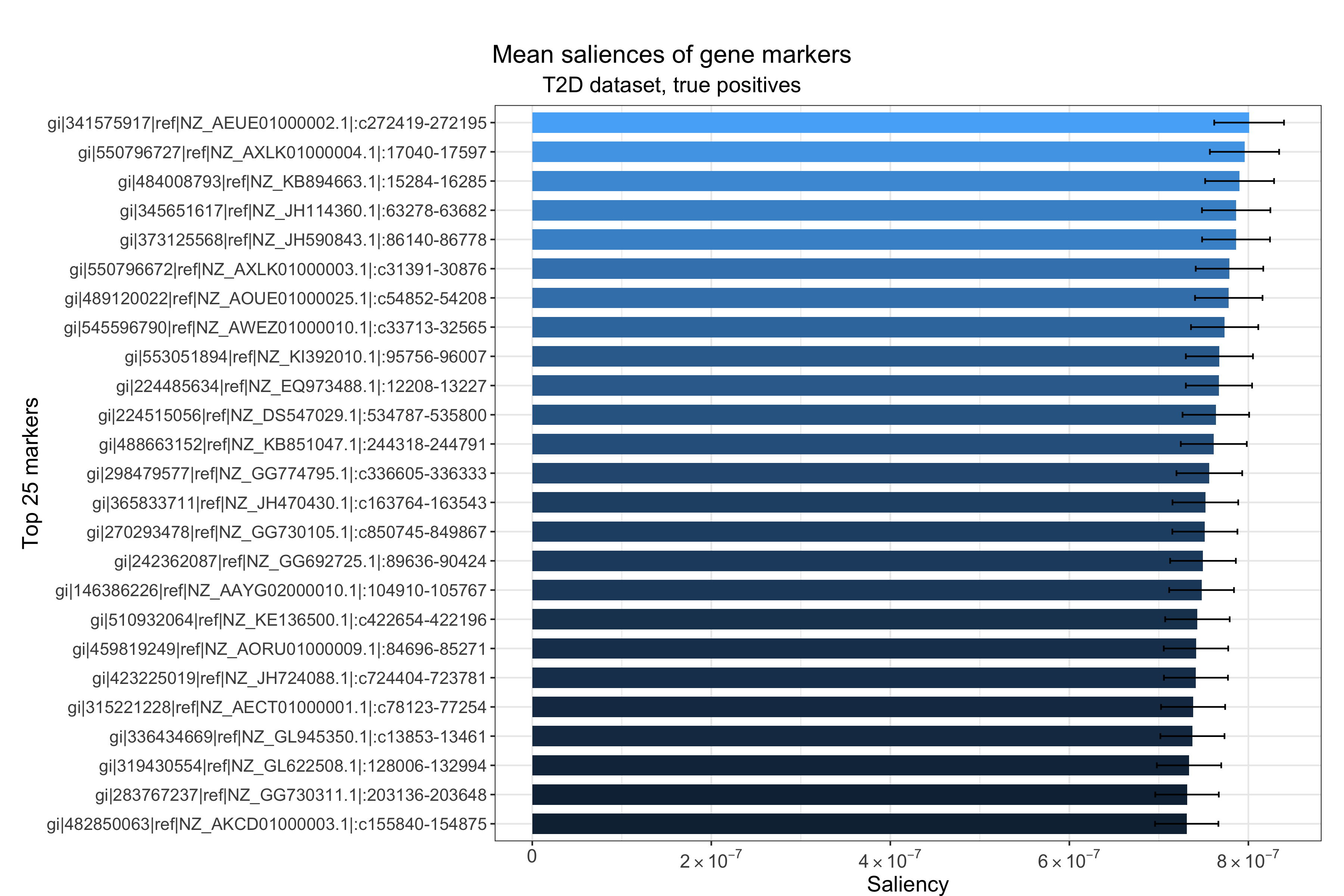

Supplement: S4 File — These files present the plots of the top 25 microbial species and strain markers for all datasets considered in this work, analogous to what Fig 5A depicts for the species from the Colorectal-EMBL dataset. Additionally, the scripts used to create the plots are included. (ZIP) [file pcbi.1010050.s009.zip › s8-file/T2D/markers_errbarplot_TP_saliences_no_rescale.png]

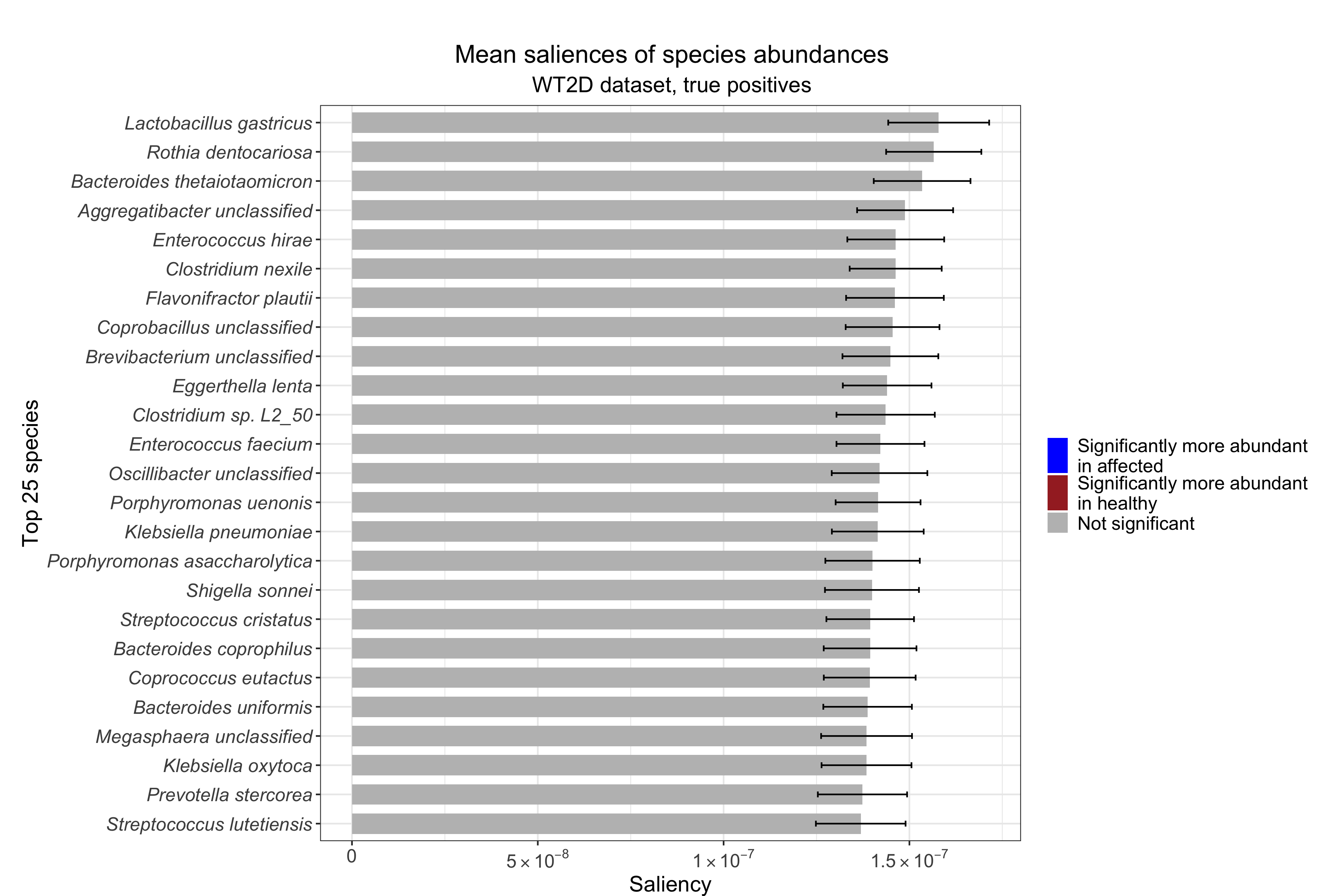

Supplement: S4 File — These files present the plots of the top 25 microbial species and strain markers for all datasets considered in this work, analogous to what Fig 5A depicts for the species from the Colorectal-EMBL dataset. Additionally, the scripts used to create the plots are included. (ZIP) [file pcbi.1010050.s009.zip › s8-file/WT2D/abundance_errbarplot_TP_saliences_no_rescale_pval-0.1.png]

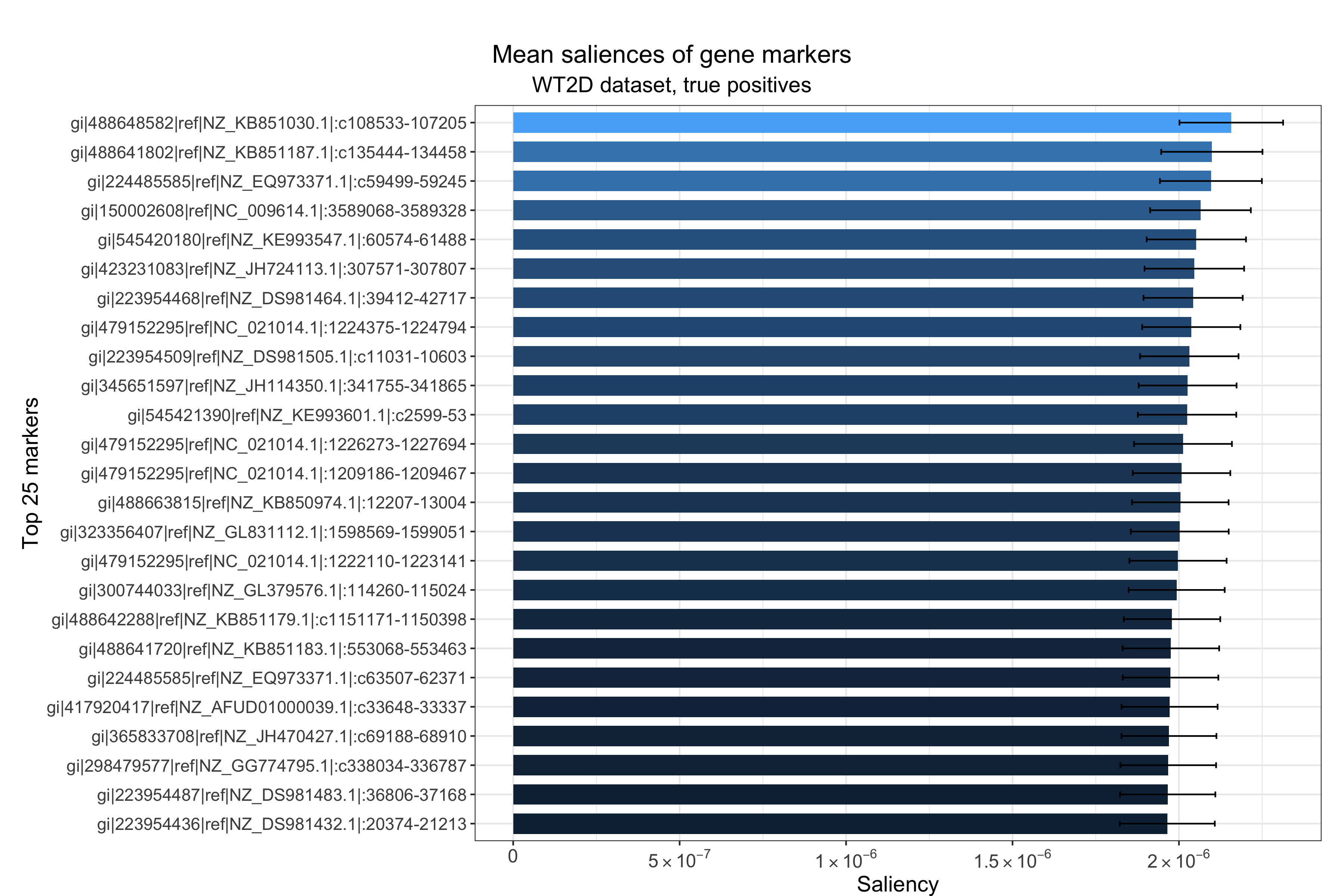

Supplement: S4 File — These files present the plots of the top 25 microbial species and strain markers for all datasets considered in this work, analogous to what Fig 5A depicts for the species from the Colorectal-EMBL dataset. Additionally, the scripts used to create the plots are included. (ZIP) [file pcbi.1010050.s009.zip › s8-file/WT2D/markers_errbarplot_TP_saliences_no_rescale.png]

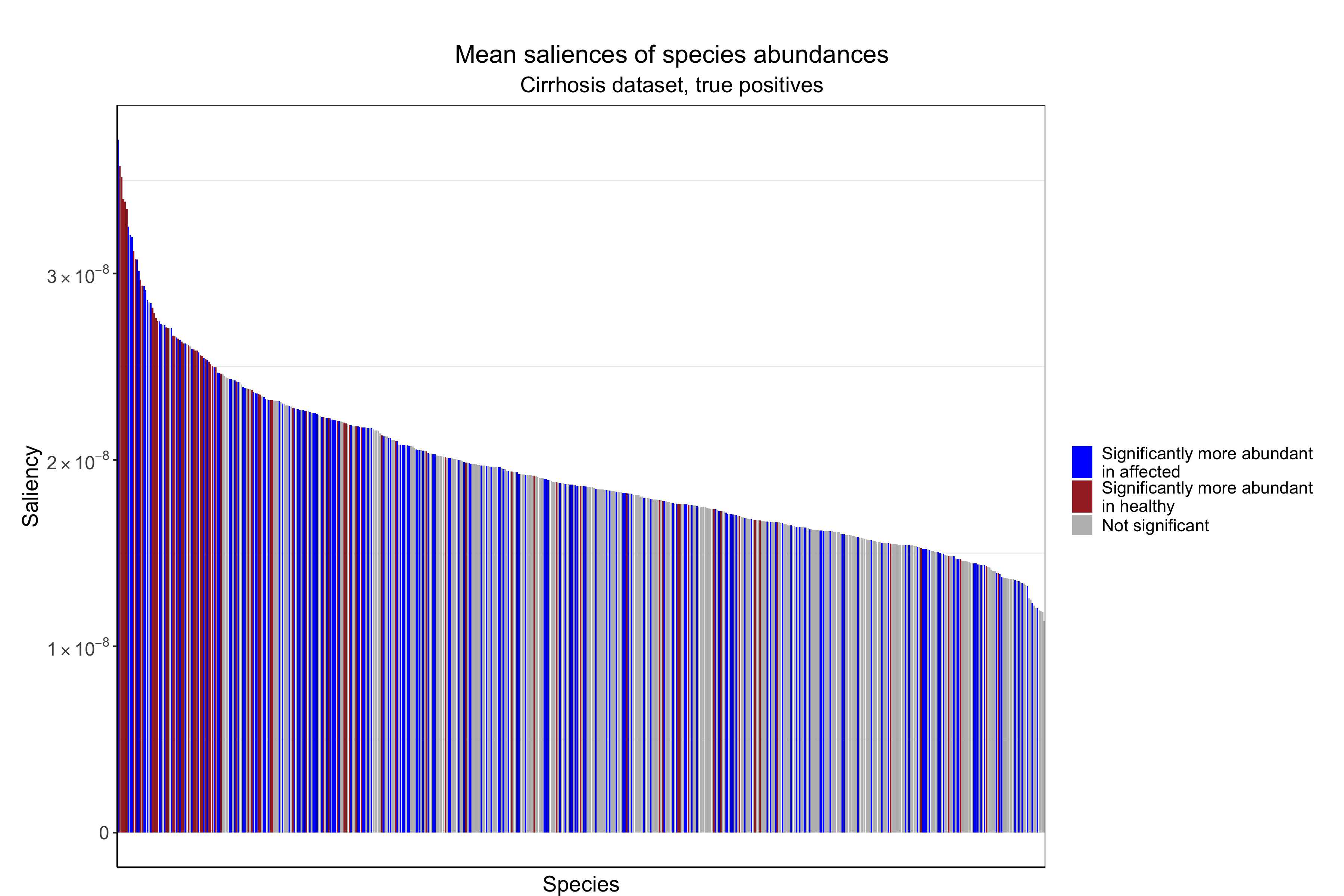

Supplement: S5 File — For each dataset, two different kinds of plots are available. (A) the histogram of the average saliency distribution over microbial species. Species are sorted from left to right by decreasing saliency. Species abundance significance in healthy (red) and affected (blue) individuals was calculated using a Wilcoxon test for each microbial species for two unpaired samples: healthy and affected individuals. (B) violin plots of the saliency distributions for microbial species grouped by significance: significantly more abundant in affected (blue), significantly more abundant in healthy (red), no significance (grey). (ZIP) [file pcbi.1010050.s010.zip › s9-file/Cirrhosis/abundance_barplot_TP_saliences_no_rescale_pval-0.1.png]

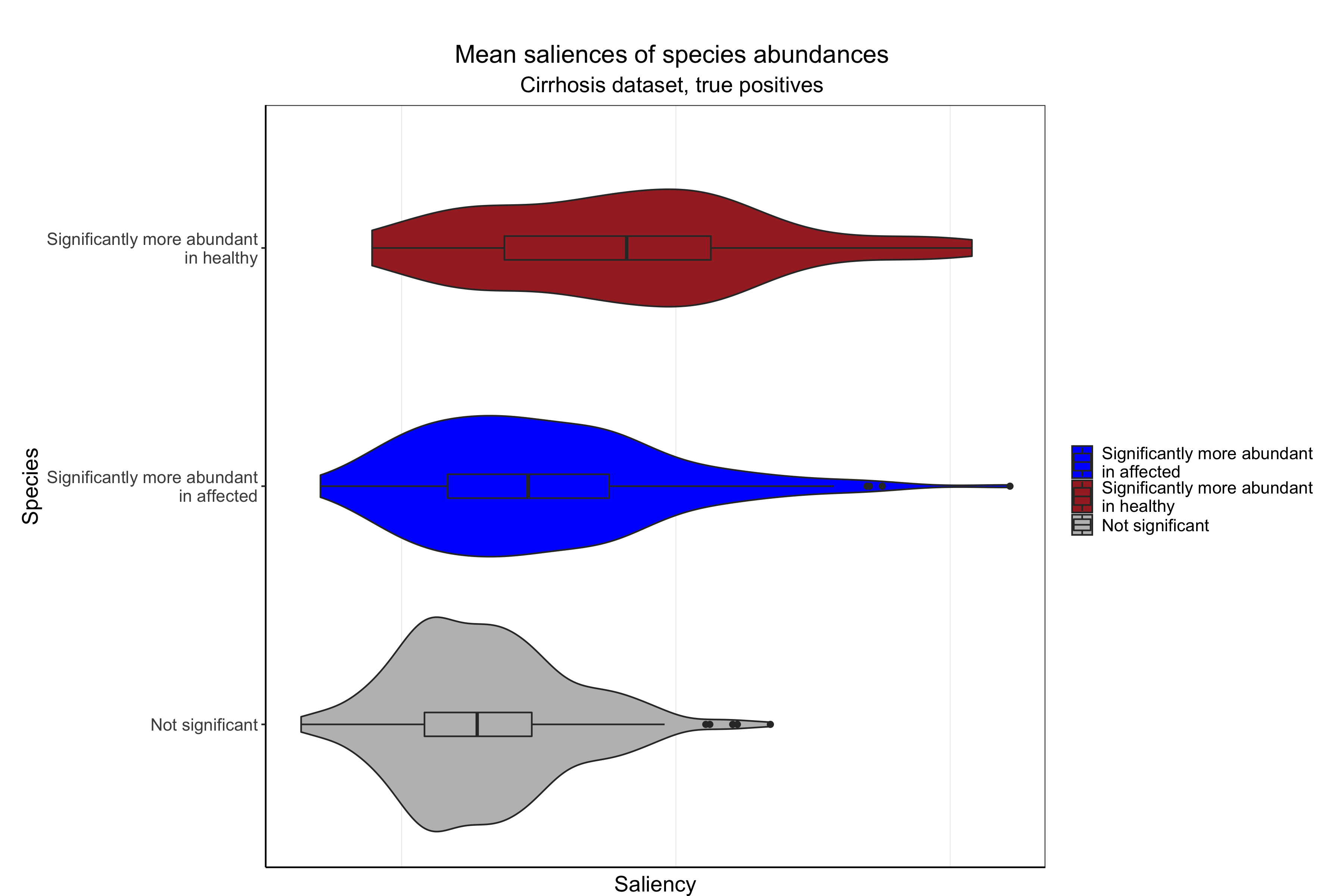

Supplement: S5 File — For each dataset, two different kinds of plots are available. (A) the histogram of the average saliency distribution over microbial species. Species are sorted from left to right by decreasing saliency. Species abundance significance in healthy (red) and affected (blue) individuals was calculated using a Wilcoxon test for each microbial species for two unpaired samples: healthy and affected individuals. (B) violin plots of the saliency distributions for microbial species grouped by significance: significantly more abundant in affected (blue), significantly more abundant in healthy (red), no significance (grey). (ZIP) [file pcbi.1010050.s010.zip › s9-file/Cirrhosis/abundance_violin_TP_saliences_no_rescale_pval-0.1.png]

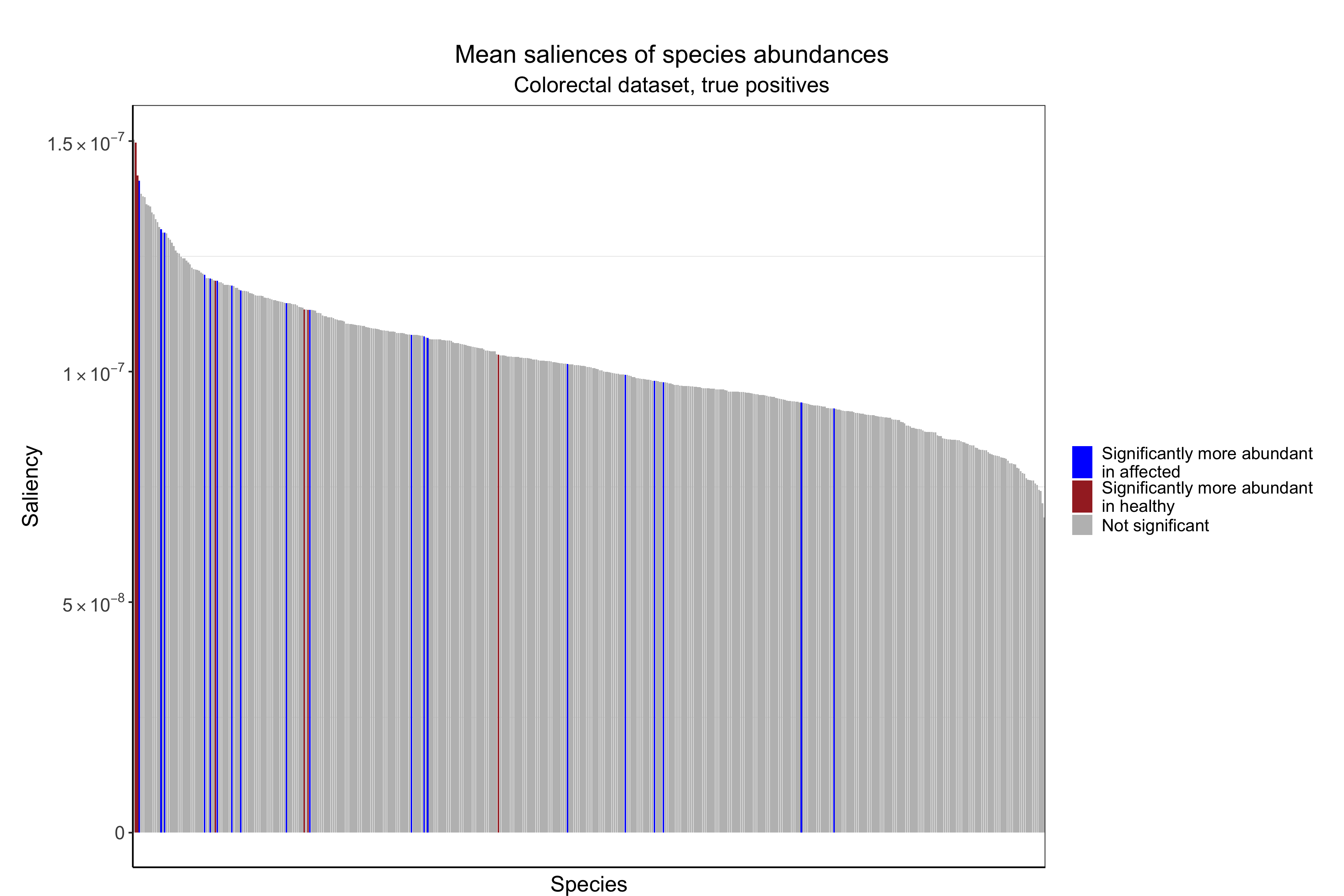

Supplement: S5 File — For each dataset, two different kinds of plots are available. (A) the histogram of the average saliency distribution over microbial species. Species are sorted from left to right by decreasing saliency. Species abundance significance in healthy (red) and affected (blue) individuals was calculated using a Wilcoxon test for each microbial species for two unpaired samples: healthy and affected individuals. (B) violin plots of the saliency distributions for microbial species grouped by significance: significantly more abundant in affected (blue), significantly more abundant in healthy (red), no significance (grey). (ZIP) [file pcbi.1010050.s010.zip › s9-file/Colorectal/abundance_barplot_TP_saliences_no_rescale_pval-0.1.png]

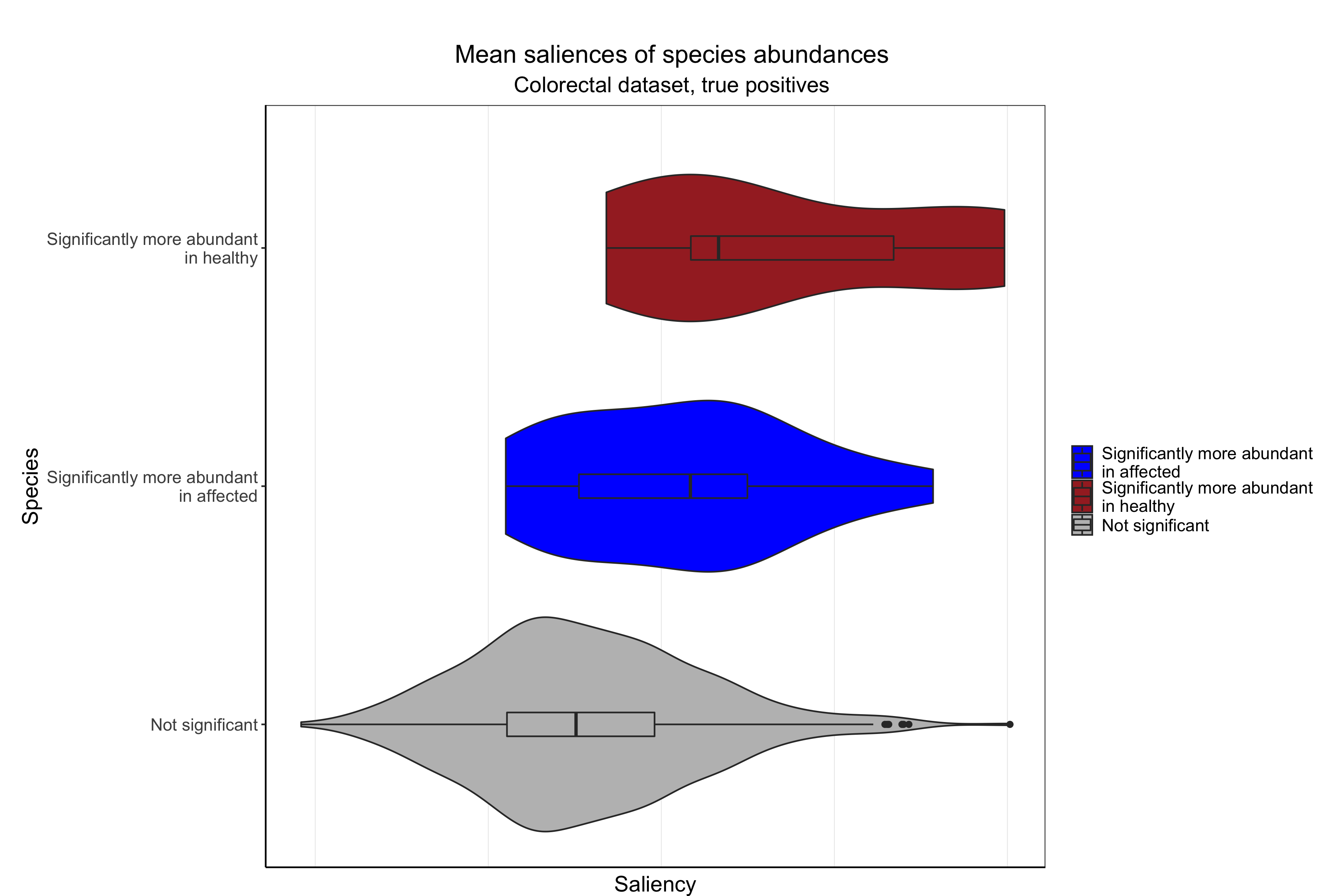

Supplement: S5 File — For each dataset, two different kinds of plots are available. (A) the histogram of the average saliency distribution over microbial species. Species are sorted from left to right by decreasing saliency. Species abundance significance in healthy (red) and affected (blue) individuals was calculated using a Wilcoxon test for each microbial species for two unpaired samples: healthy and affected individuals. (B) violin plots of the saliency distributions for microbial species grouped by significance: significantly more abundant in affected (blue), significantly more abundant in healthy (red), no significance (grey). (ZIP) [file pcbi.1010050.s010.zip › s9-file/Colorectal/abundance_violin_TP_saliences_no_rescale_pval-0.1.png]

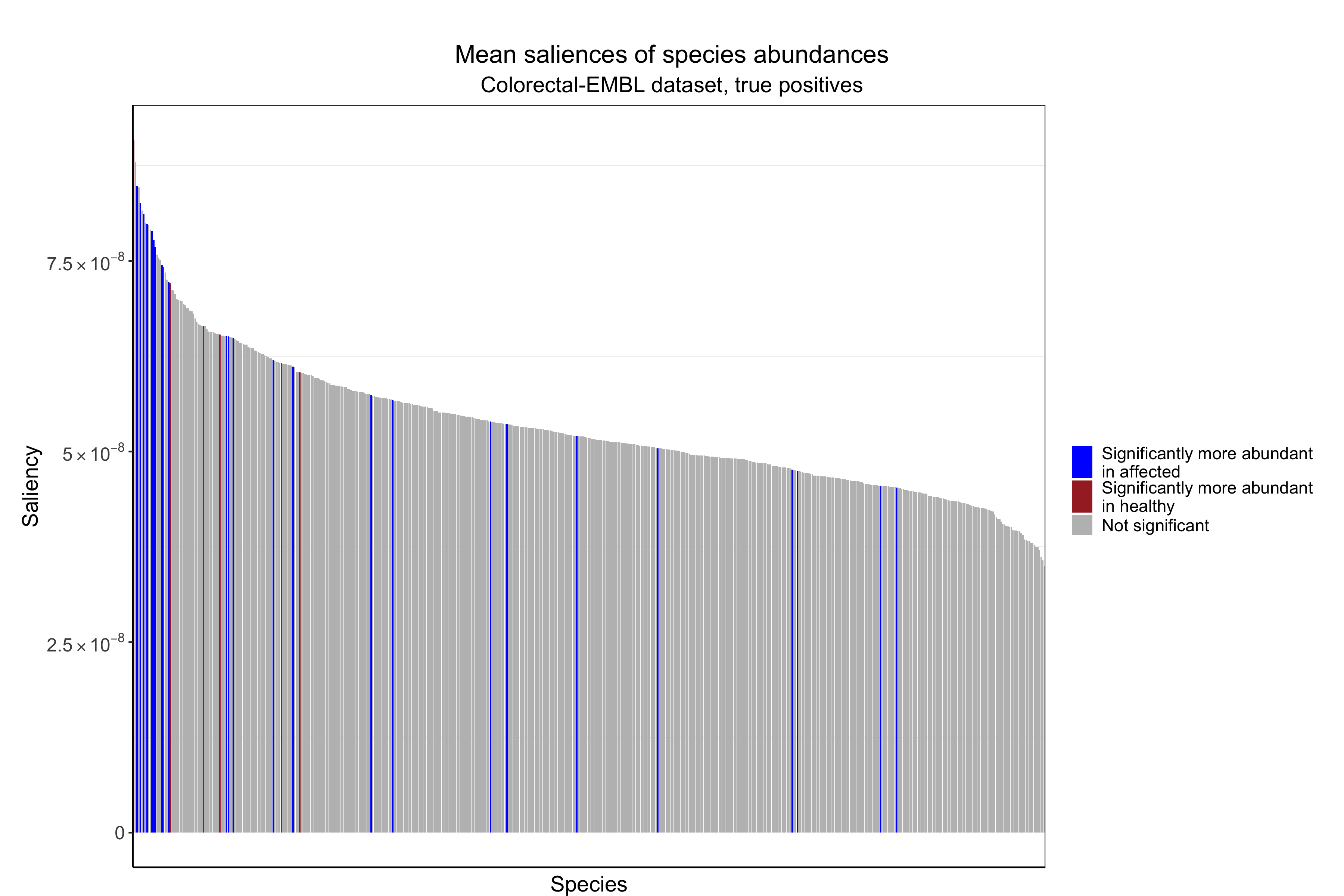

Supplement: S5 File — For each dataset, two different kinds of plots are available. (A) the histogram of the average saliency distribution over microbial species. Species are sorted from left to right by decreasing saliency. Species abundance significance in healthy (red) and affected (blue) individuals was calculated using a Wilcoxon test for each microbial species for two unpaired samples: healthy and affected individuals. (B) violin plots of the saliency distributions for microbial species grouped by significance: significantly more abundant in affected (blue), significantly more abundant in healthy (red), no significance (grey). (ZIP) [file pcbi.1010050.s010.zip › s9-file/Colorectal-EMBL/abundance_barplot_TP_saliences_no_rescale_pval-0.1.png]

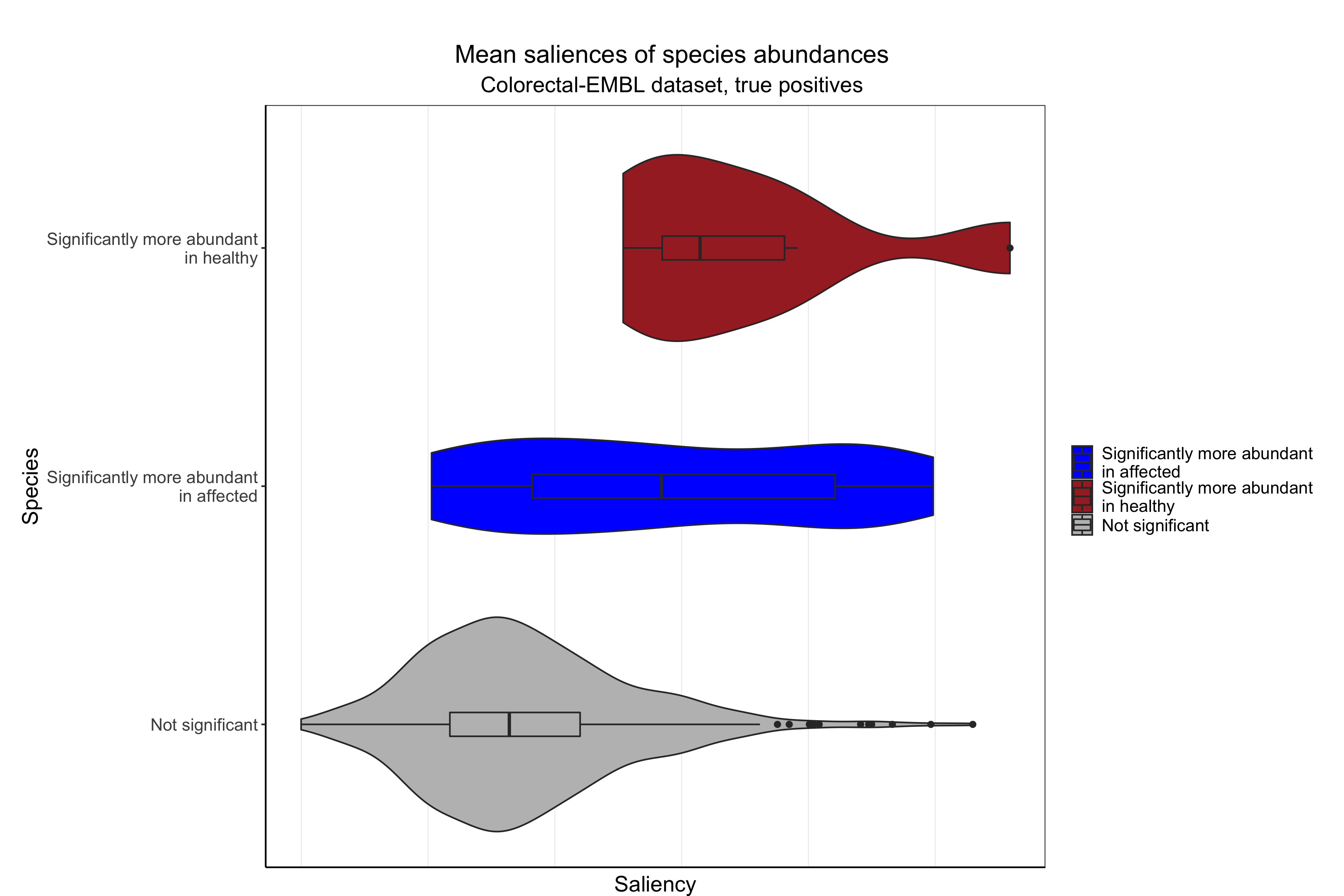

Supplement: S5 File — For each dataset, two different kinds of plots are available. (A) the histogram of the average saliency distribution over microbial species. Species are sorted from left to right by decreasing saliency. Species abundance significance in healthy (red) and affected (blue) individuals was calculated using a Wilcoxon test for each microbial species for two unpaired samples: healthy and affected individuals. (B) violin plots of the saliency distributions for microbial species grouped by significance: significantly more abundant in affected (blue), significantly more abundant in healthy (red), no significance (grey). (ZIP) [file pcbi.1010050.s010.zip › s9-file/Colorectal-EMBL/abundance_violin_TP_saliences_no_rescale_pval-0.1.png]

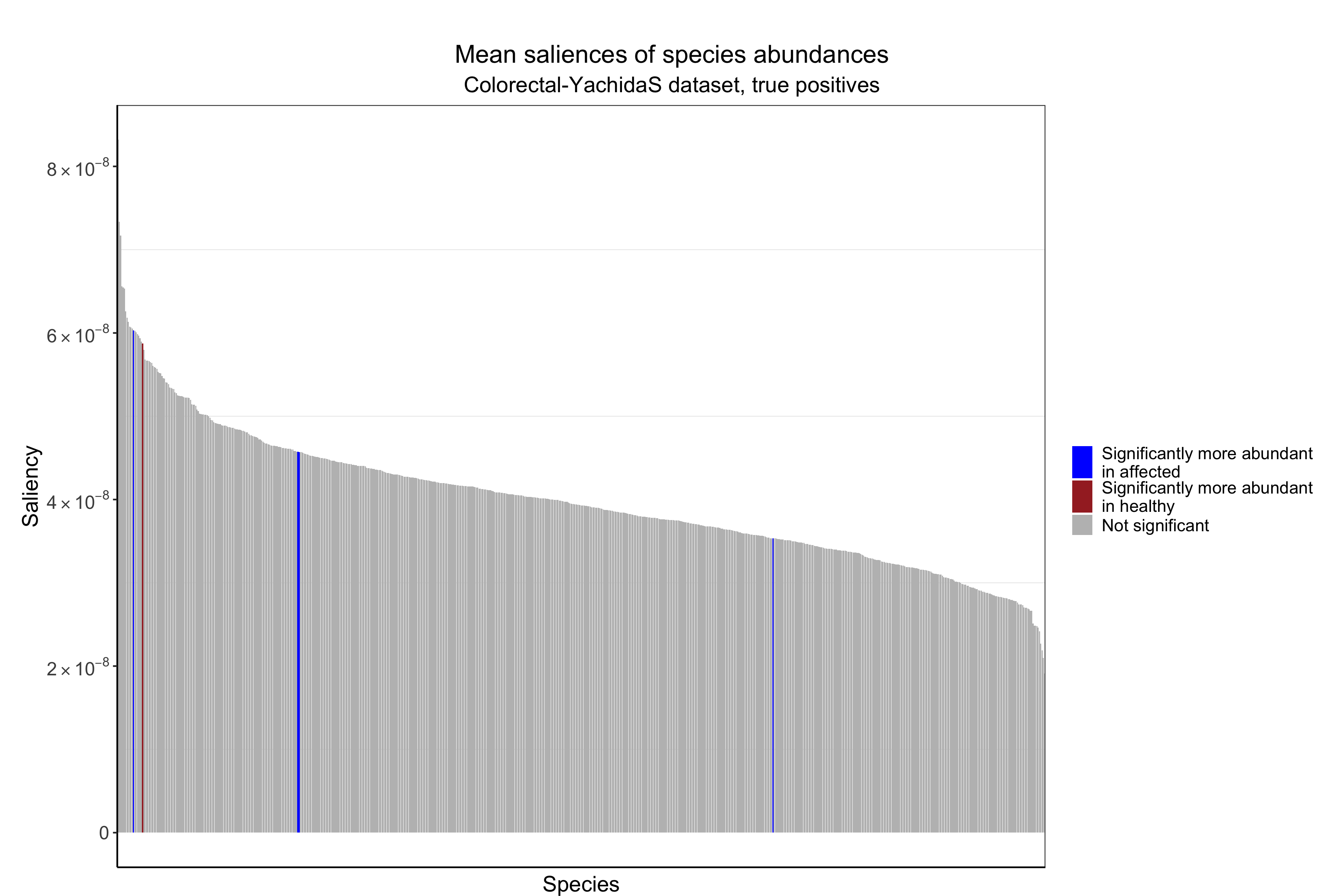

Supplement: S5 File — For each dataset, two different kinds of plots are available. (A) the histogram of the average saliency distribution over microbial species. Species are sorted from left to right by decreasing saliency. Species abundance significance in healthy (red) and affected (blue) individuals was calculated using a Wilcoxon test for each microbial species for two unpaired samples: healthy and affected individuals. (B) violin plots of the saliency distributions for microbial species grouped by significance: significantly more abundant in affected (blue), significantly more abundant in healthy (red), no significance (grey). (ZIP) [file pcbi.1010050.s010.zip › s9-file/Colorectal-YachidaS/abundance_barplot_TP_saliences_no_rescale_pval-0.1.png]

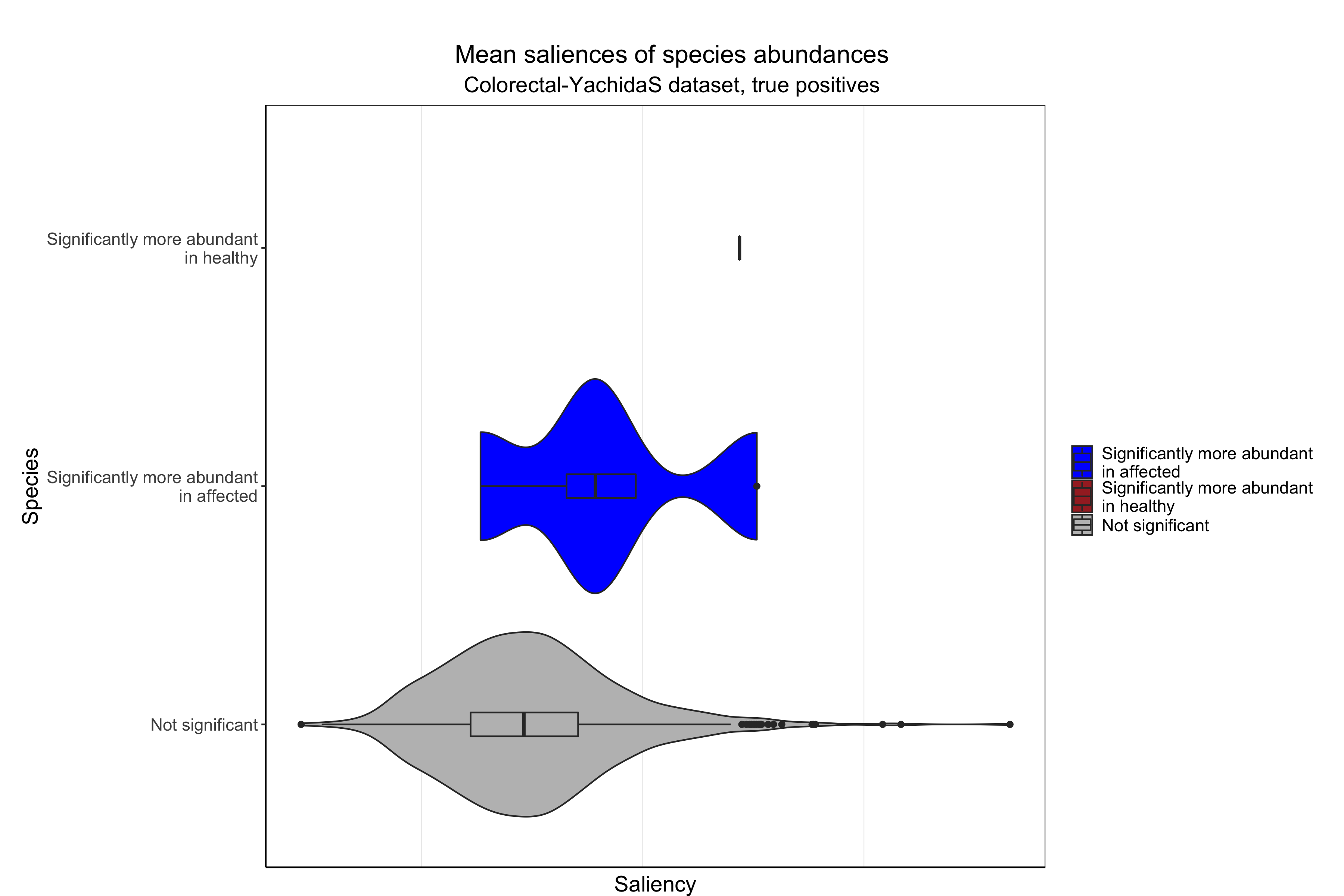

Supplement: S5 File — For each dataset, two different kinds of plots are available. (A) the histogram of the average saliency distribution over microbial species. Species are sorted from left to right by decreasing saliency. Species abundance significance in healthy (red) and affected (blue) individuals was calculated using a Wilcoxon test for each microbial species for two unpaired samples: healthy and affected individuals. (B) violin plots of the saliency distributions for microbial species grouped by significance: significantly more abundant in affected (blue), significantly more abundant in healthy (red), no significance (grey). (ZIP) [file pcbi.1010050.s010.zip › s9-file/Colorectal-YachidaS/abundance_violin_TP_saliences_no_rescale_pval-0.1.png]

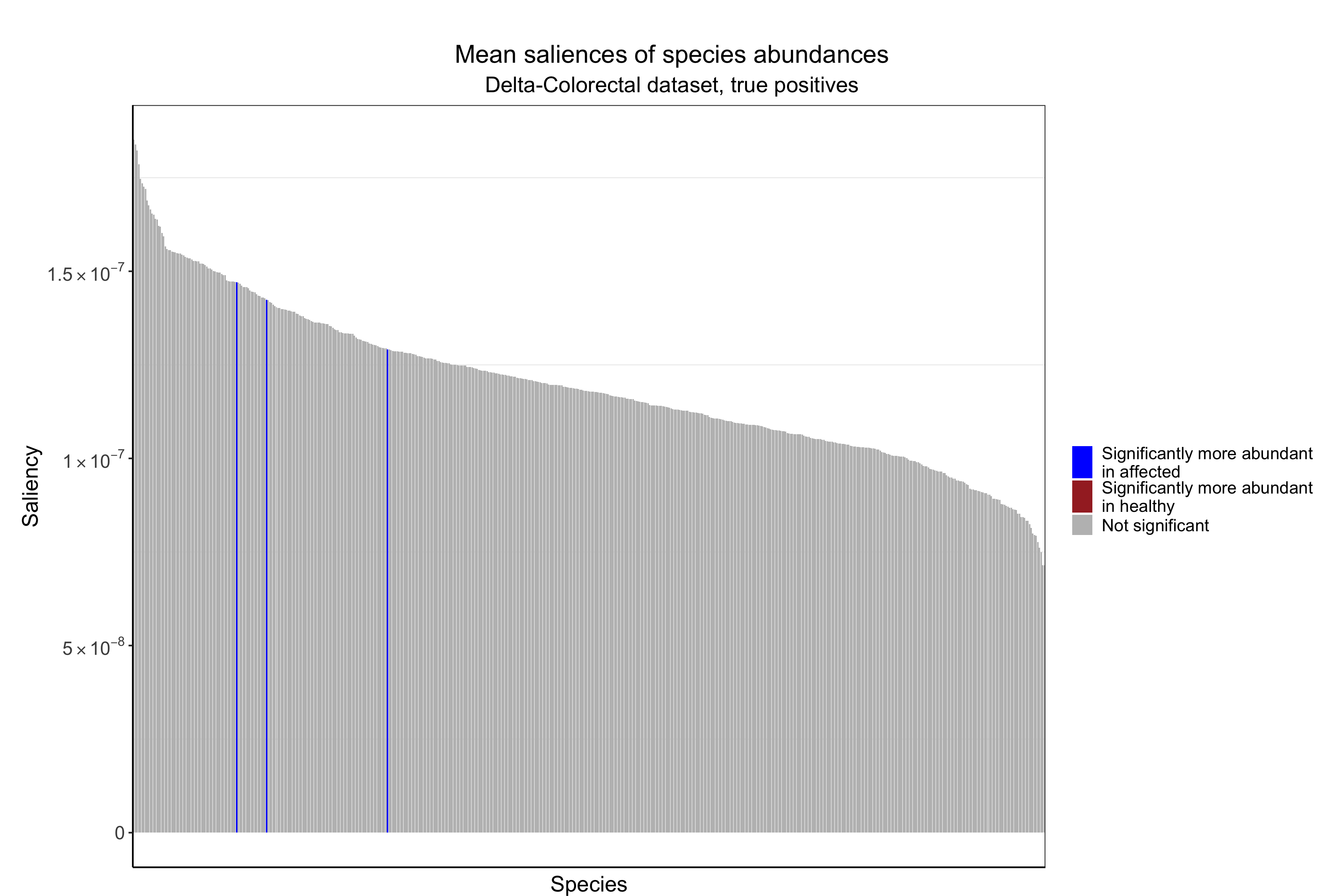

Supplement: S5 File — For each dataset, two different kinds of plots are available. (A) the histogram of the average saliency distribution over microbial species. Species are sorted from left to right by decreasing saliency. Species abundance significance in healthy (red) and affected (blue) individuals was calculated using a Wilcoxon test for each microbial species for two unpaired samples: healthy and affected individuals. (B) violin plots of the saliency distributions for microbial species grouped by significance: significantly more abundant in affected (blue), significantly more abundant in healthy (red), no significance (grey). (ZIP) [file pcbi.1010050.s010.zip › s9-file/Delta-Colorectal/abundance_barplot_TP_saliences_no_rescale_pval-0.1.png]

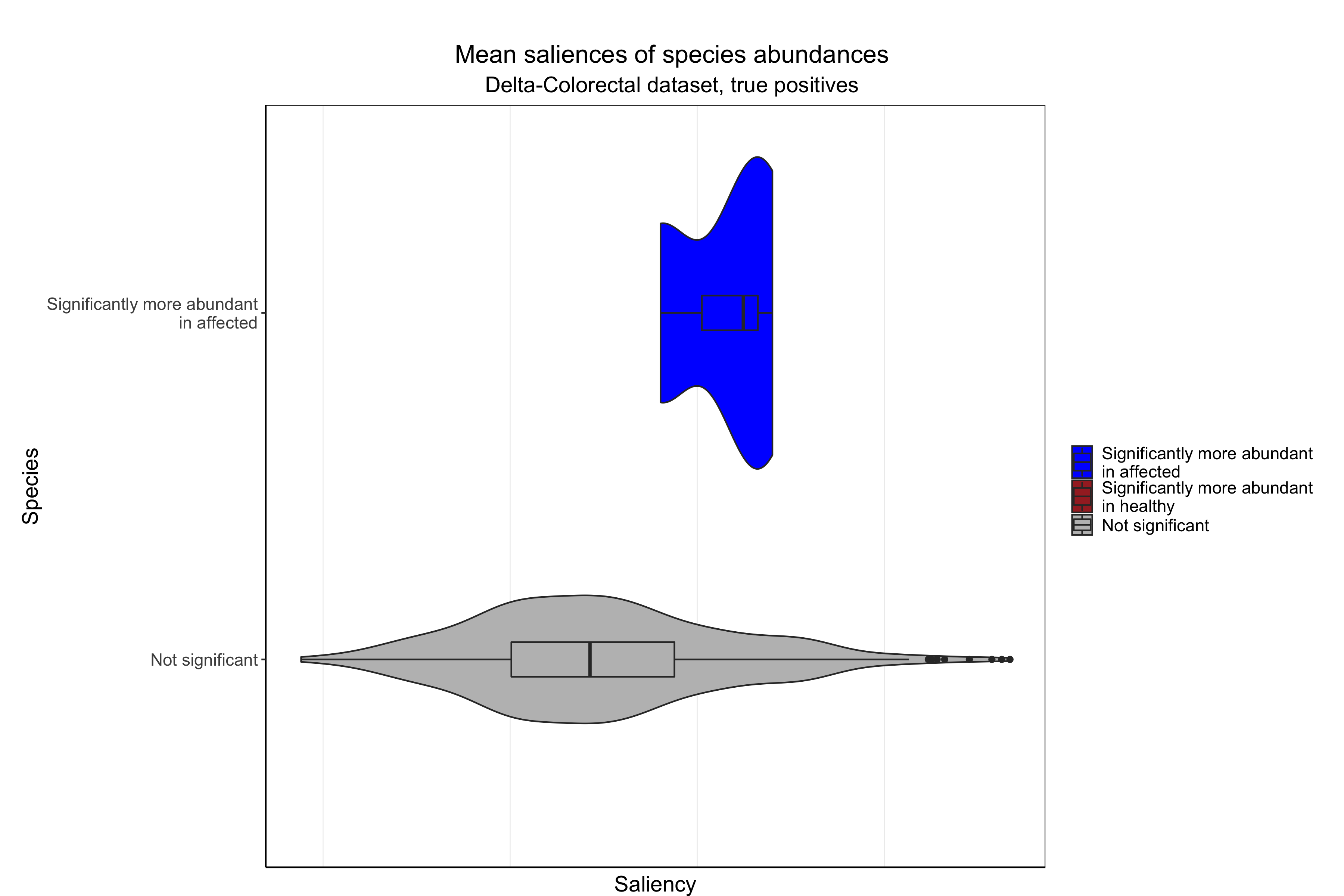

Supplement: S5 File — For each dataset, two different kinds of plots are available. (A) the histogram of the average saliency distribution over microbial species. Species are sorted from left to right by decreasing saliency. Species abundance significance in healthy (red) and affected (blue) individuals was calculated using a Wilcoxon test for each microbial species for two unpaired samples: healthy and affected individuals. (B) violin plots of the saliency distributions for microbial species grouped by significance: significantly more abundant in affected (blue), significantly more abundant in healthy (red), no significance (grey). (ZIP) [file pcbi.1010050.s010.zip › s9-file/Delta-Colorectal/abundance_violin_TP_saliences_no_rescale_pval-0.1.png]

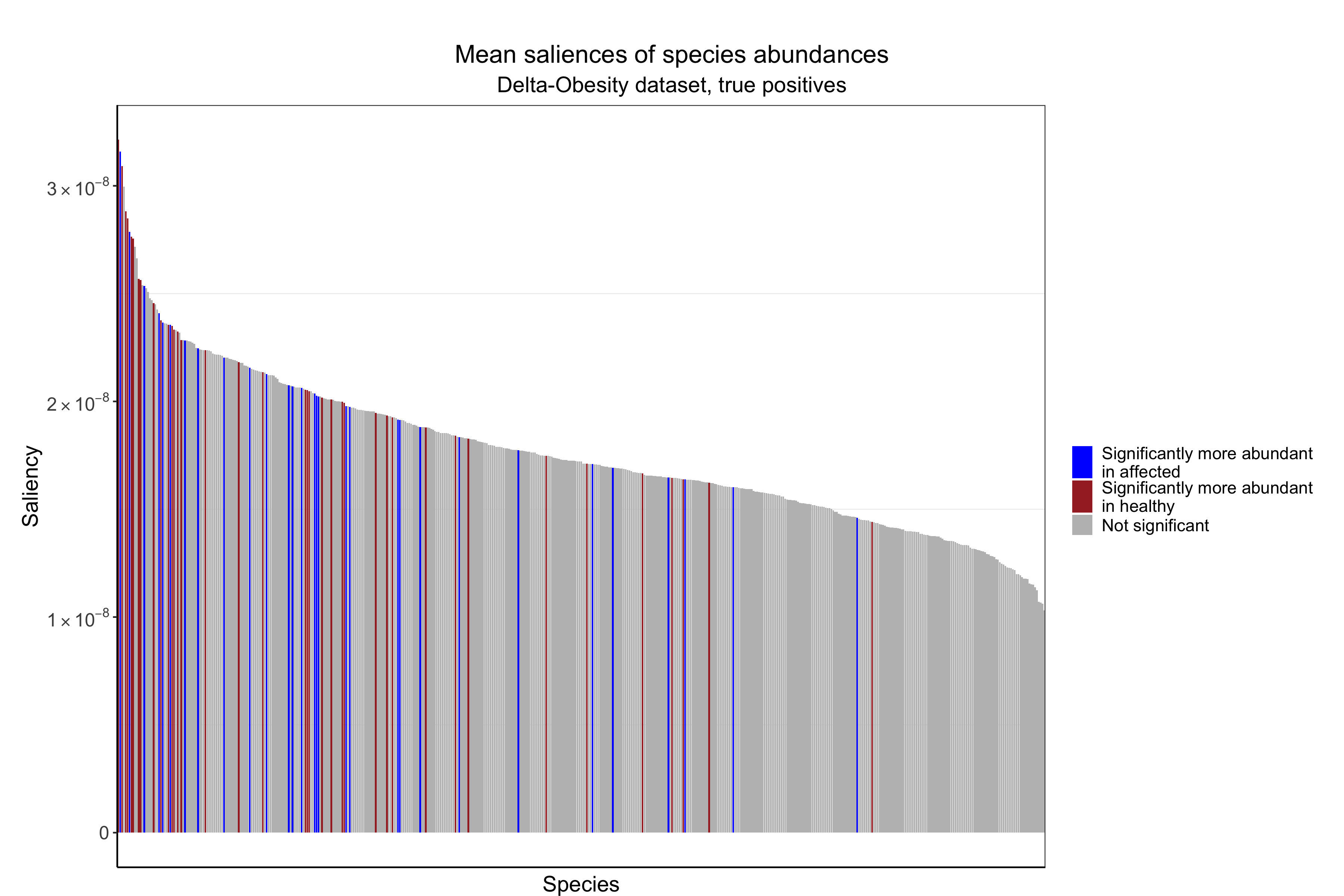

Supplement: S5 File — For each dataset, two different kinds of plots are available. (A) the histogram of the average saliency distribution over microbial species. Species are sorted from left to right by decreasing saliency. Species abundance significance in healthy (red) and affected (blue) individuals was calculated using a Wilcoxon test for each microbial species for two unpaired samples: healthy and affected individuals. (B) violin plots of the saliency distributions for microbial species grouped by significance: significantly more abundant in affected (blue), significantly more abundant in healthy (red), no significance (grey). (ZIP) [file pcbi.1010050.s010.zip › s9-file/Delta-Obesity/abundance_barplot_TP_saliences_no_rescale_pval-0.1.png]

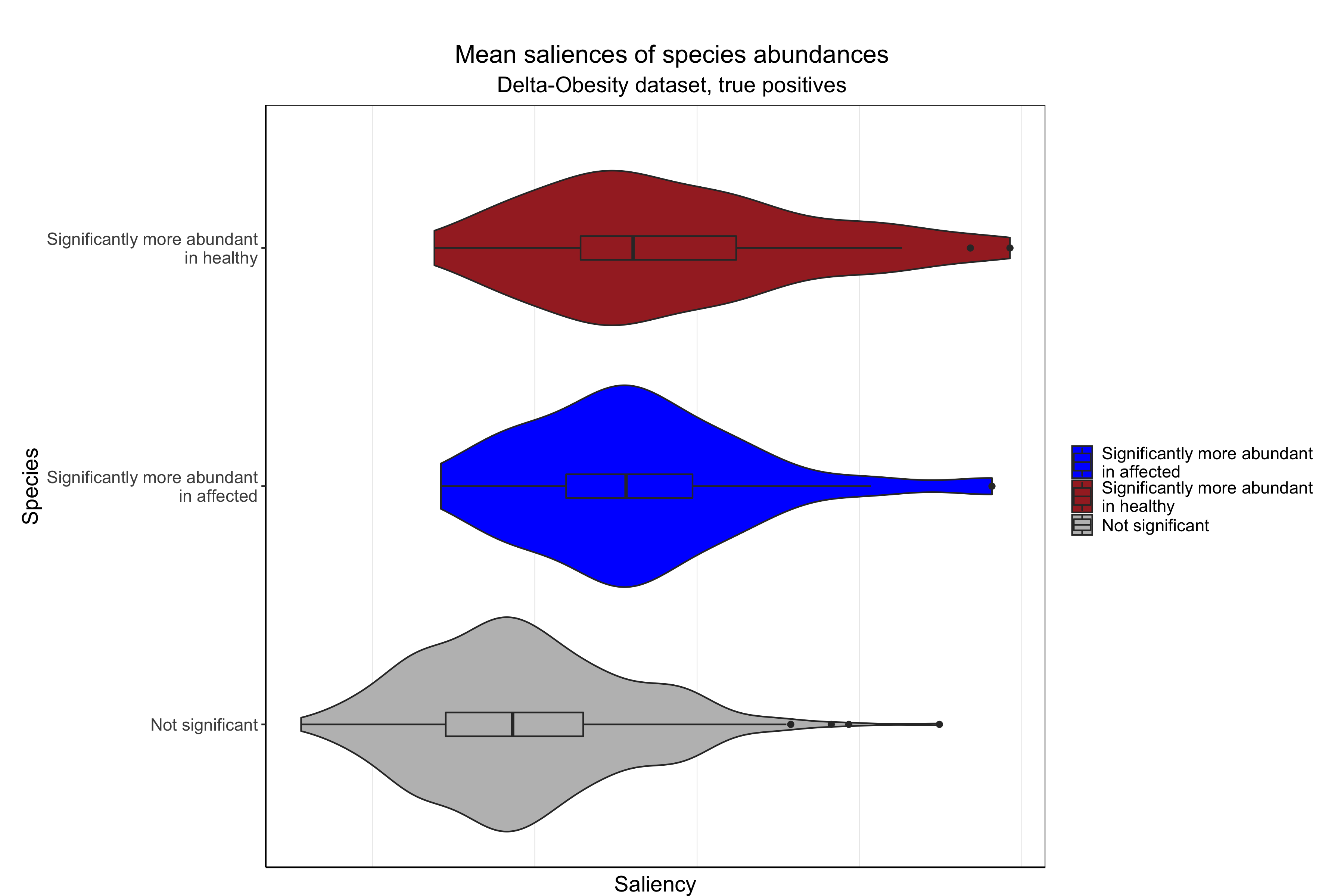

Supplement: S5 File — For each dataset, two different kinds of plots are available. (A) the histogram of the average saliency distribution over microbial species. Species are sorted from left to right by decreasing saliency. Species abundance significance in healthy (red) and affected (blue) individuals was calculated using a Wilcoxon test for each microbial species for two unpaired samples: healthy and affected individuals. (B) violin plots of the saliency distributions for microbial species grouped by significance: significantly more abundant in affected (blue), significantly more abundant in healthy (red), no significance (grey). (ZIP) [file pcbi.1010050.s010.zip › s9-file/Delta-Obesity/abundance_violin_TP_saliences_no_rescale_pval-0.1.png]

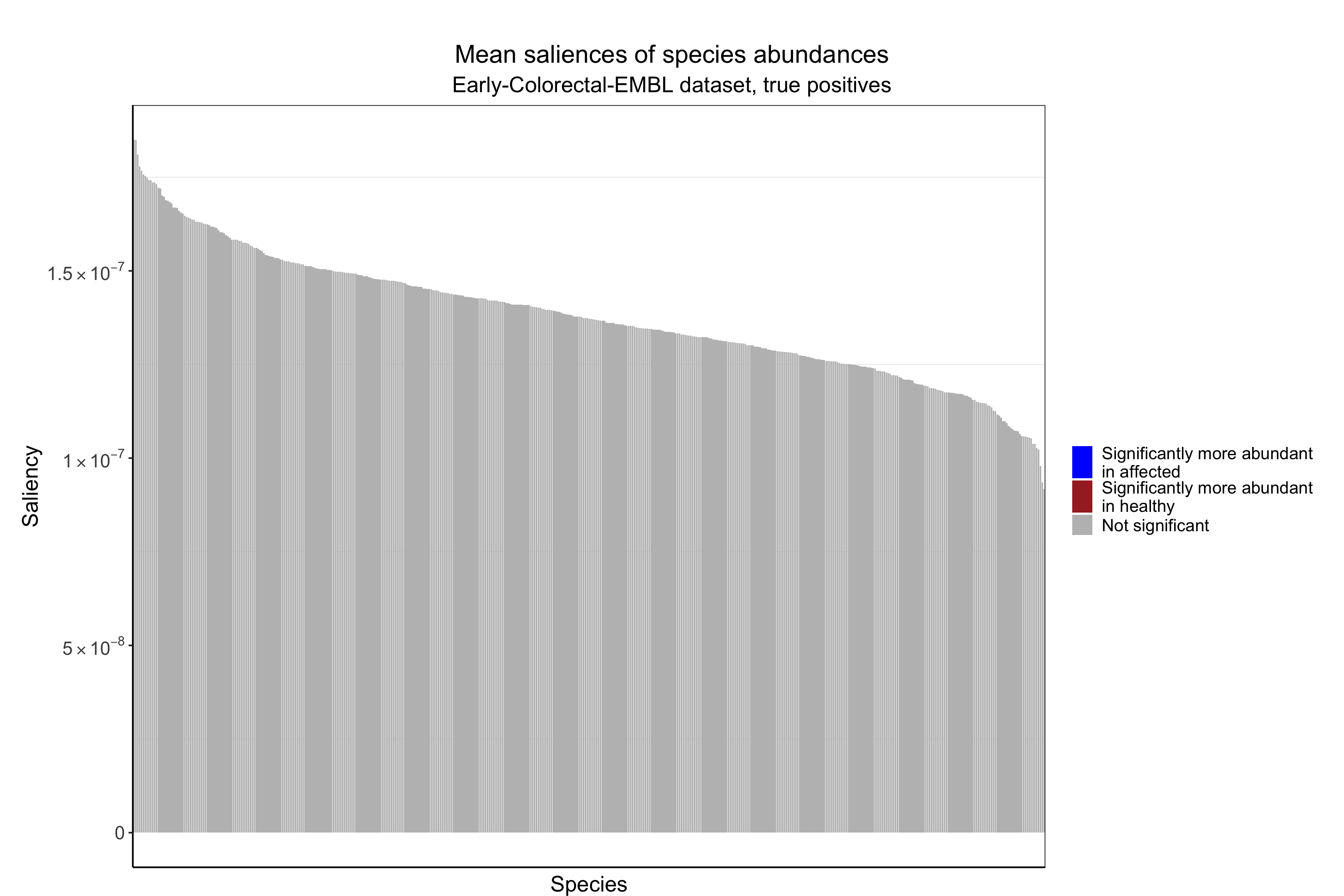

Supplement: S5 File — For each dataset, two different kinds of plots are available. (A) the histogram of the average saliency distribution over microbial species. Species are sorted from left to right by decreasing saliency. Species abundance significance in healthy (red) and affected (blue) individuals was calculated using a Wilcoxon test for each microbial species for two unpaired samples: healthy and affected individuals. (B) violin plots of the saliency distributions for microbial species grouped by significance: significantly more abundant in affected (blue), significantly more abundant in healthy (red), no significance (grey). (ZIP) [file pcbi.1010050.s010.zip › s9-file/Early-Colorectal-EMBL/abundance_barplot_TP_saliences_no_rescale_pval-0.1.png]

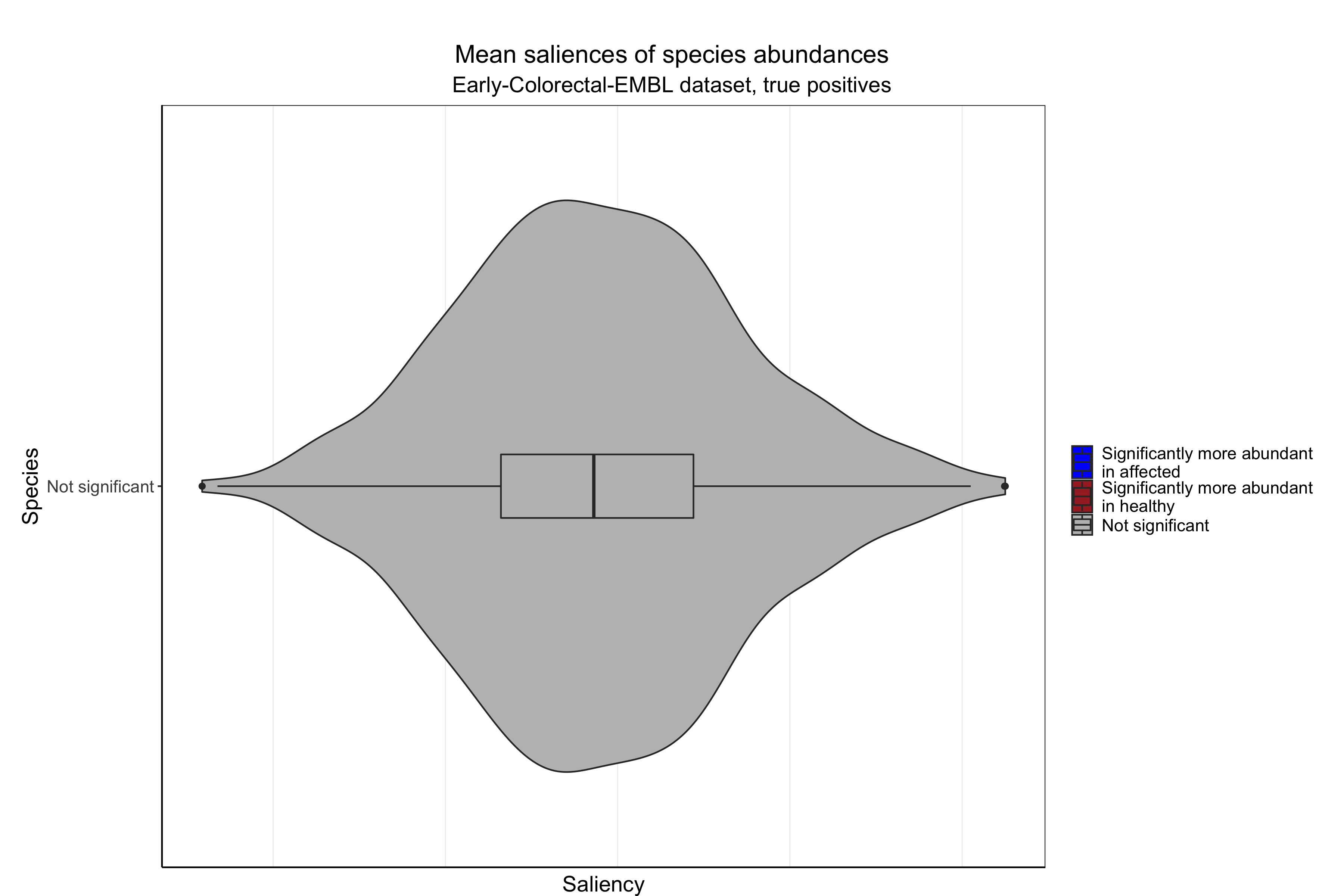

Supplement: S5 File — For each dataset, two different kinds of plots are available. (A) the histogram of the average saliency distribution over microbial species. Species are sorted from left to right by decreasing saliency. Species abundance significance in healthy (red) and affected (blue) individuals was calculated using a Wilcoxon test for each microbial species for two unpaired samples: healthy and affected individuals. (B) violin plots of the saliency distributions for microbial species grouped by significance: significantly more abundant in affected (blue), significantly more abundant in healthy (red), no significance (grey). (ZIP) [file pcbi.1010050.s010.zip › s9-file/Early-Colorectal-EMBL/abundance_violin_TP_saliences_no_rescale_pval-0.1.png]

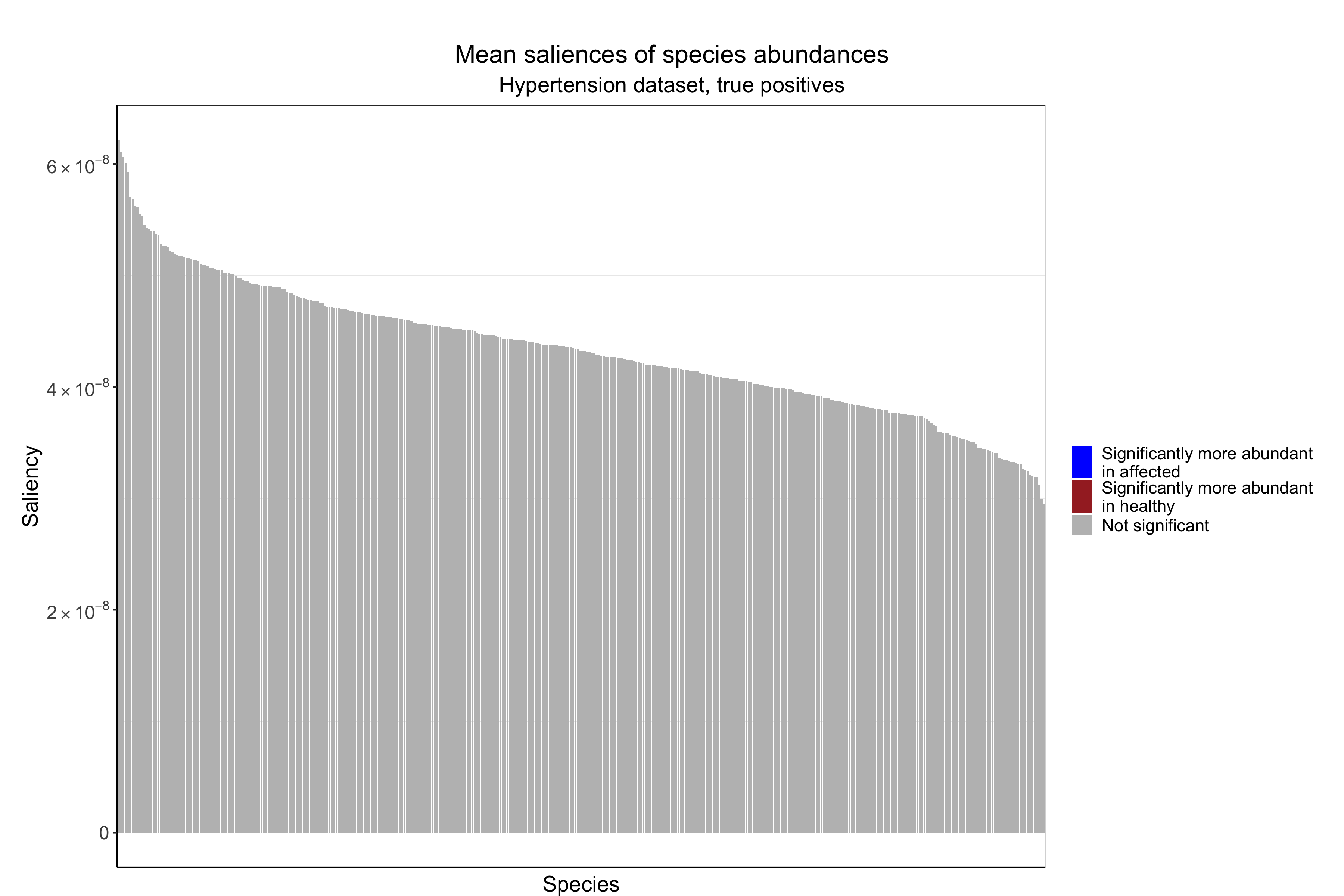

Supplement: S5 File — For each dataset, two different kinds of plots are available. (A) the histogram of the average saliency distribution over microbial species. Species are sorted from left to right by decreasing saliency. Species abundance significance in healthy (red) and affected (blue) individuals was calculated using a Wilcoxon test for each microbial species for two unpaired samples: healthy and affected individuals. (B) violin plots of the saliency distributions for microbial species grouped by significance: significantly more abundant in affected (blue), significantly more abundant in healthy (red), no significance (grey). (ZIP) [file pcbi.1010050.s010.zip › s9-file/Hypertension/abundance_barplot_TP_saliences_no_rescale_pval-0.1.png]

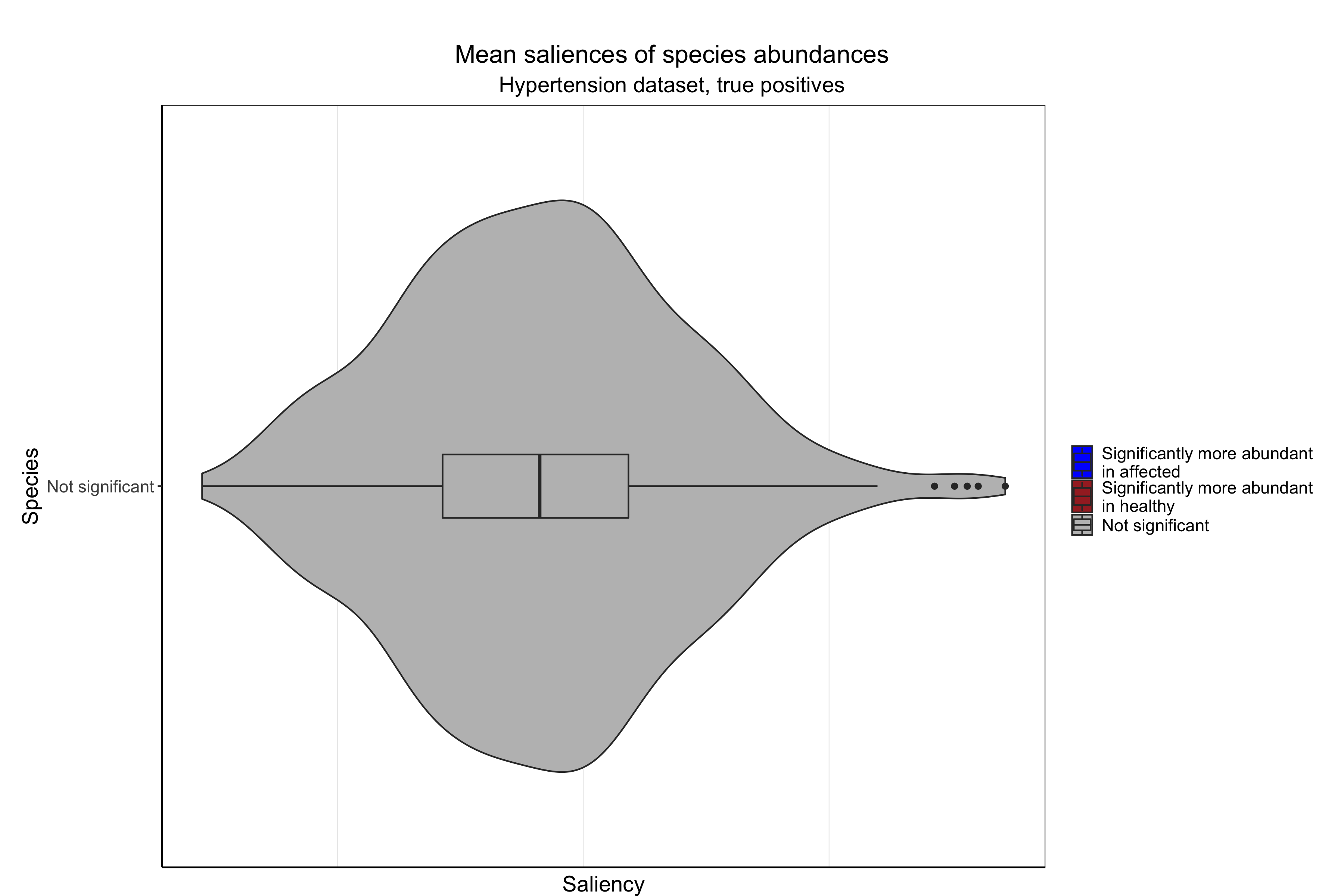

Supplement: S5 File — For each dataset, two different kinds of plots are available. (A) the histogram of the average saliency distribution over microbial species. Species are sorted from left to right by decreasing saliency. Species abundance significance in healthy (red) and affected (blue) individuals was calculated using a Wilcoxon test for each microbial species for two unpaired samples: healthy and affected individuals. (B) violin plots of the saliency distributions for microbial species grouped by significance: significantly more abundant in affected (blue), significantly more abundant in healthy (red), no significance (grey). (ZIP) [file pcbi.1010050.s010.zip › s9-file/Hypertension/abundance_violin_TP_saliences_no_rescale_pval-0.1.png]

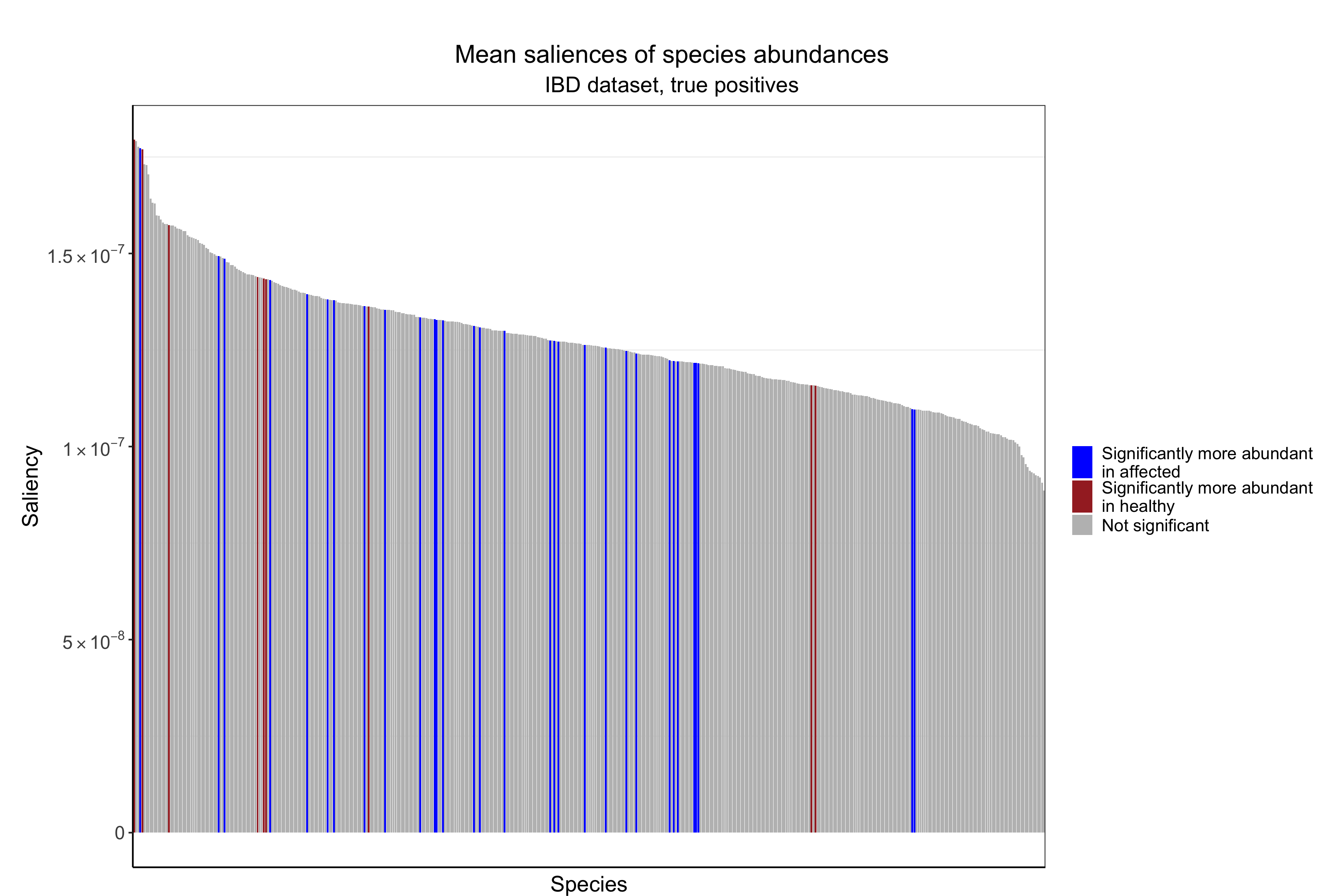

Supplement: S5 File — For each dataset, two different kinds of plots are available. (A) the histogram of the average saliency distribution over microbial species. Species are sorted from left to right by decreasing saliency. Species abundance significance in healthy (red) and affected (blue) individuals was calculated using a Wilcoxon test for each microbial species for two unpaired samples: healthy and affected individuals. (B) violin plots of the saliency distributions for microbial species grouped by significance: significantly more abundant in affected (blue), significantly more abundant in healthy (red), no significance (grey). (ZIP) [file pcbi.1010050.s010.zip › s9-file/IBD/abundance_barplot_TP_saliences_no_rescale_pval-0.1.png]

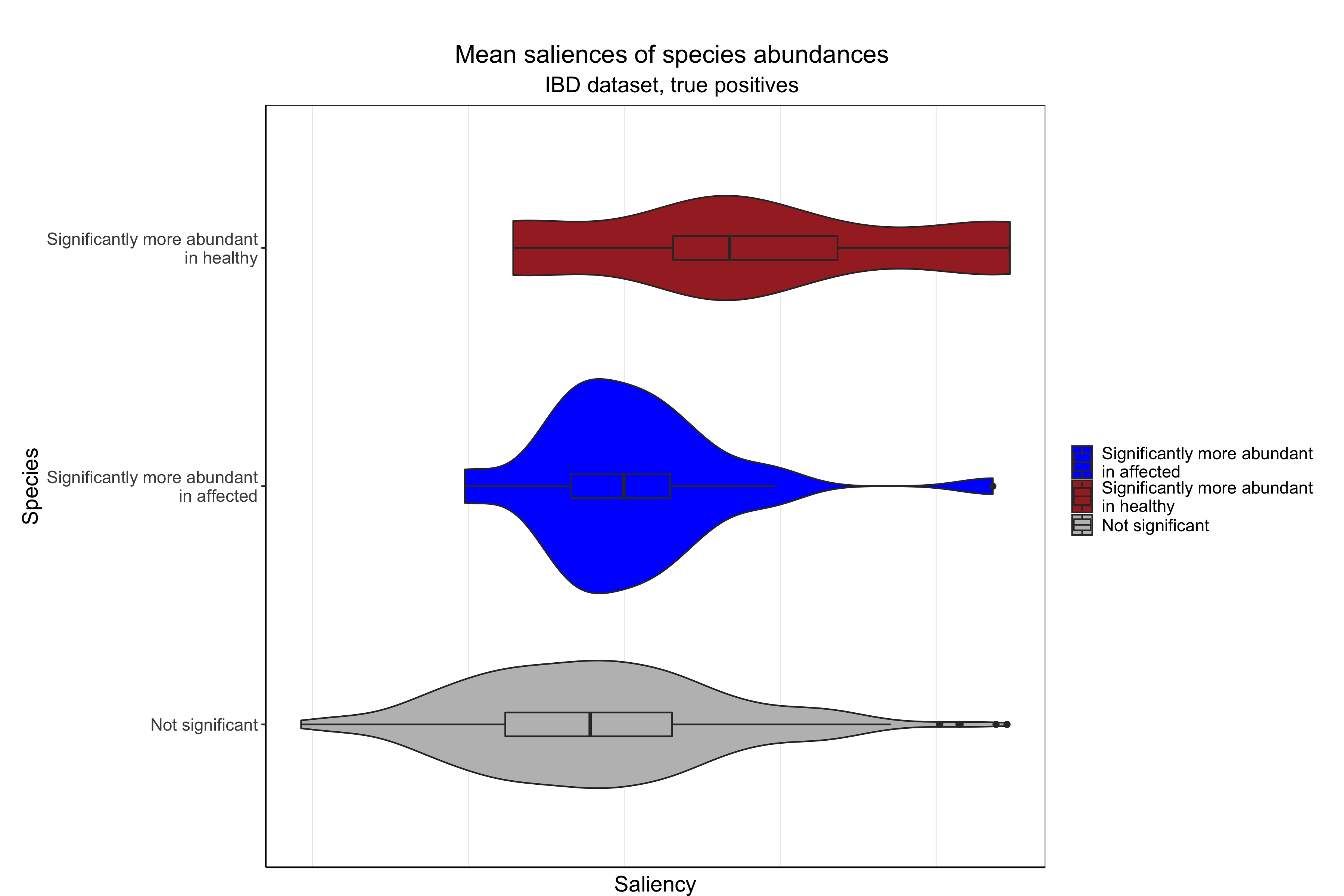

Supplement: S5 File — For each dataset, two different kinds of plots are available. (A) the histogram of the average saliency distribution over microbial species. Species are sorted from left to right by decreasing saliency. Species abundance significance in healthy (red) and affected (blue) individuals was calculated using a Wilcoxon test for each microbial species for two unpaired samples: healthy and affected individuals. (B) violin plots of the saliency distributions for microbial species grouped by significance: significantly more abundant in affected (blue), significantly more abundant in healthy (red), no significance (grey). (ZIP) [file pcbi.1010050.s010.zip › s9-file/IBD/abundance_violin_TP_saliences_no_rescale_pval-0.1.png]

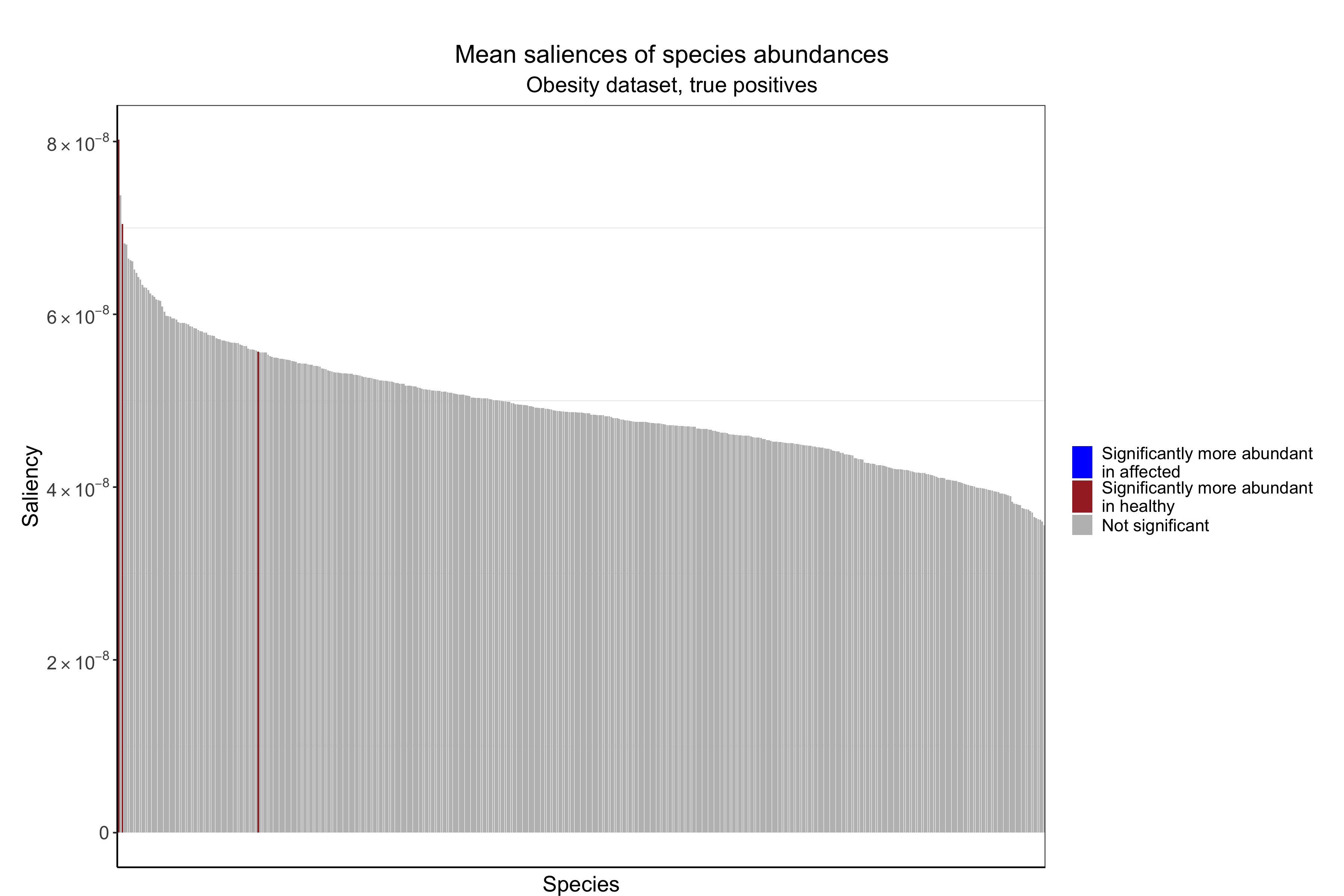

Supplement: S5 File — For each dataset, two different kinds of plots are available. (A) the histogram of the average saliency distribution over microbial species. Species are sorted from left to right by decreasing saliency. Species abundance significance in healthy (red) and affected (blue) individuals was calculated using a Wilcoxon test for each microbial species for two unpaired samples: healthy and affected individuals. (B) violin plots of the saliency distributions for microbial species grouped by significance: significantly more abundant in affected (blue), significantly more abundant in healthy (red), no significance (grey). (ZIP) [file pcbi.1010050.s010.zip › s9-file/Obesity/abundance_barplot_TP_saliences_no_rescale_pval-0.1.png]

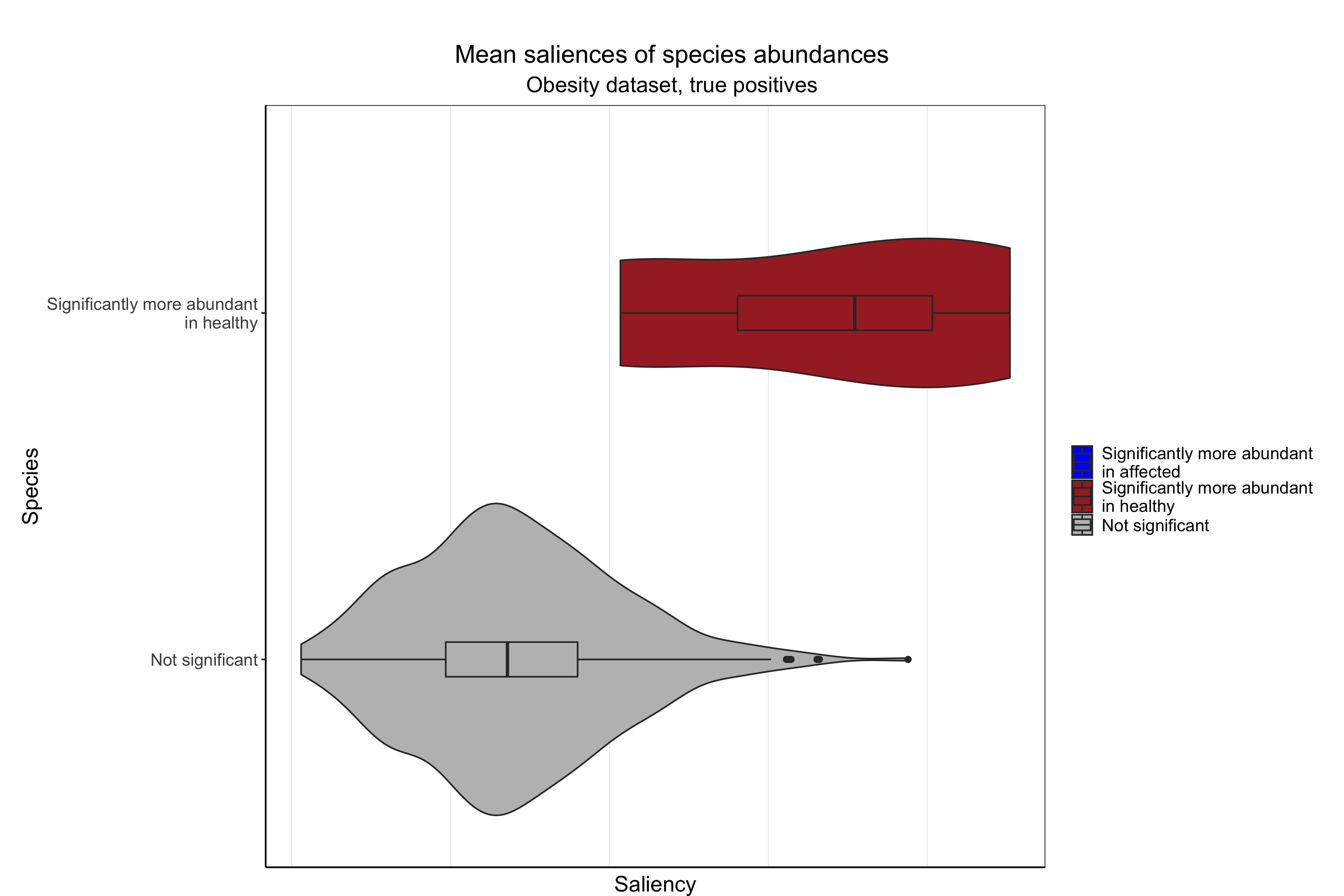

Supplement: S5 File — For each dataset, two different kinds of plots are available. (A) the histogram of the average saliency distribution over microbial species. Species are sorted from left to right by decreasing saliency. Species abundance significance in healthy (red) and affected (blue) individuals was calculated using a Wilcoxon test for each microbial species for two unpaired samples: healthy and affected individuals. (B) violin plots of the saliency distributions for microbial species grouped by significance: significantly more abundant in affected (blue), significantly more abundant in healthy (red), no significance (grey). (ZIP) [file pcbi.1010050.s010.zip › s9-file/Obesity/abundance_violin_TP_saliences_no_rescale_pval-0.1.png]

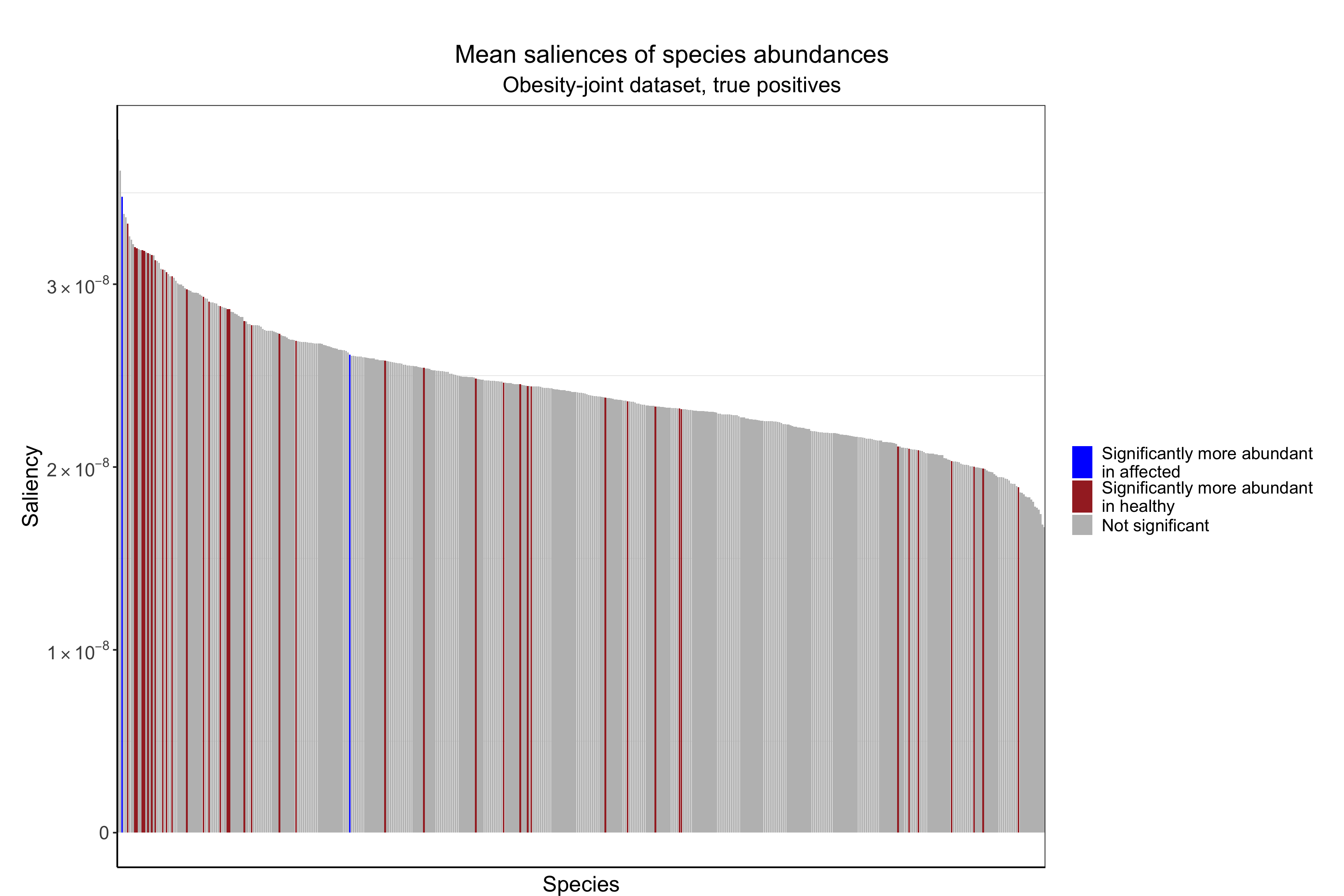

Supplement: S5 File — For each dataset, two different kinds of plots are available. (A) the histogram of the average saliency distribution over microbial species. Species are sorted from left to right by decreasing saliency. Species abundance significance in healthy (red) and affected (blue) individuals was calculated using a Wilcoxon test for each microbial species for two unpaired samples: healthy and affected individuals. (B) violin plots of the saliency distributions for microbial species grouped by significance: significantly more abundant in affected (blue), significantly more abundant in healthy (red), no significance (grey). (ZIP) [file pcbi.1010050.s010.zip › s9-file/Obesity-joint/abundance_barplot_TP_saliences_no_rescale_pval-0.1.png]

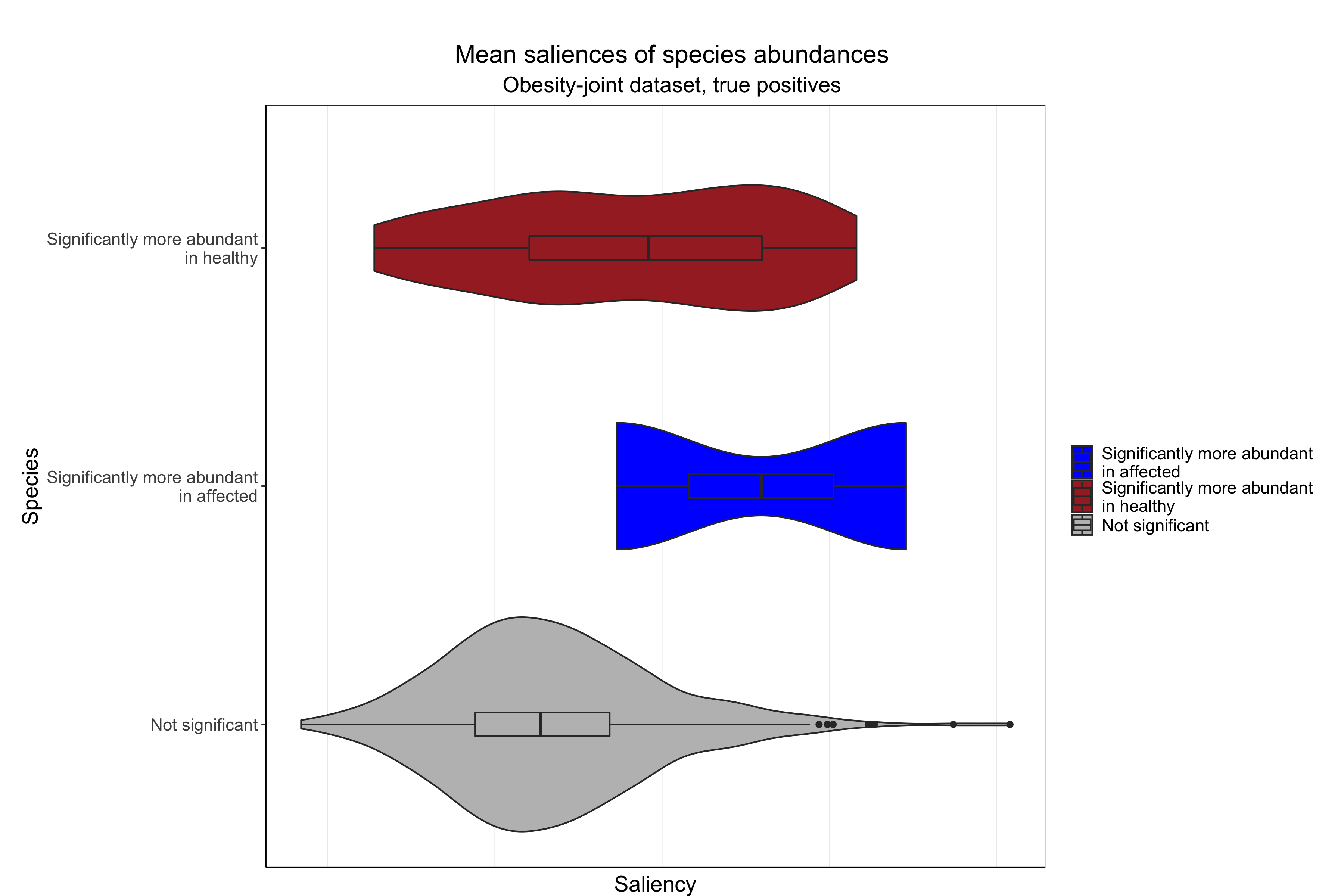

Supplement: S5 File — For each dataset, two different kinds of plots are available. (A) the histogram of the average saliency distribution over microbial species. Species are sorted from left to right by decreasing saliency. Species abundance significance in healthy (red) and affected (blue) individuals was calculated using a Wilcoxon test for each microbial species for two unpaired samples: healthy and affected individuals. (B) violin plots of the saliency distributions for microbial species grouped by significance: significantly more abundant in affected (blue), significantly more abundant in healthy (red), no significance (grey). (ZIP) [file pcbi.1010050.s010.zip › s9-file/Obesity-joint/abundance_violin_TP_saliences_no_rescale_pval-0.1.png]

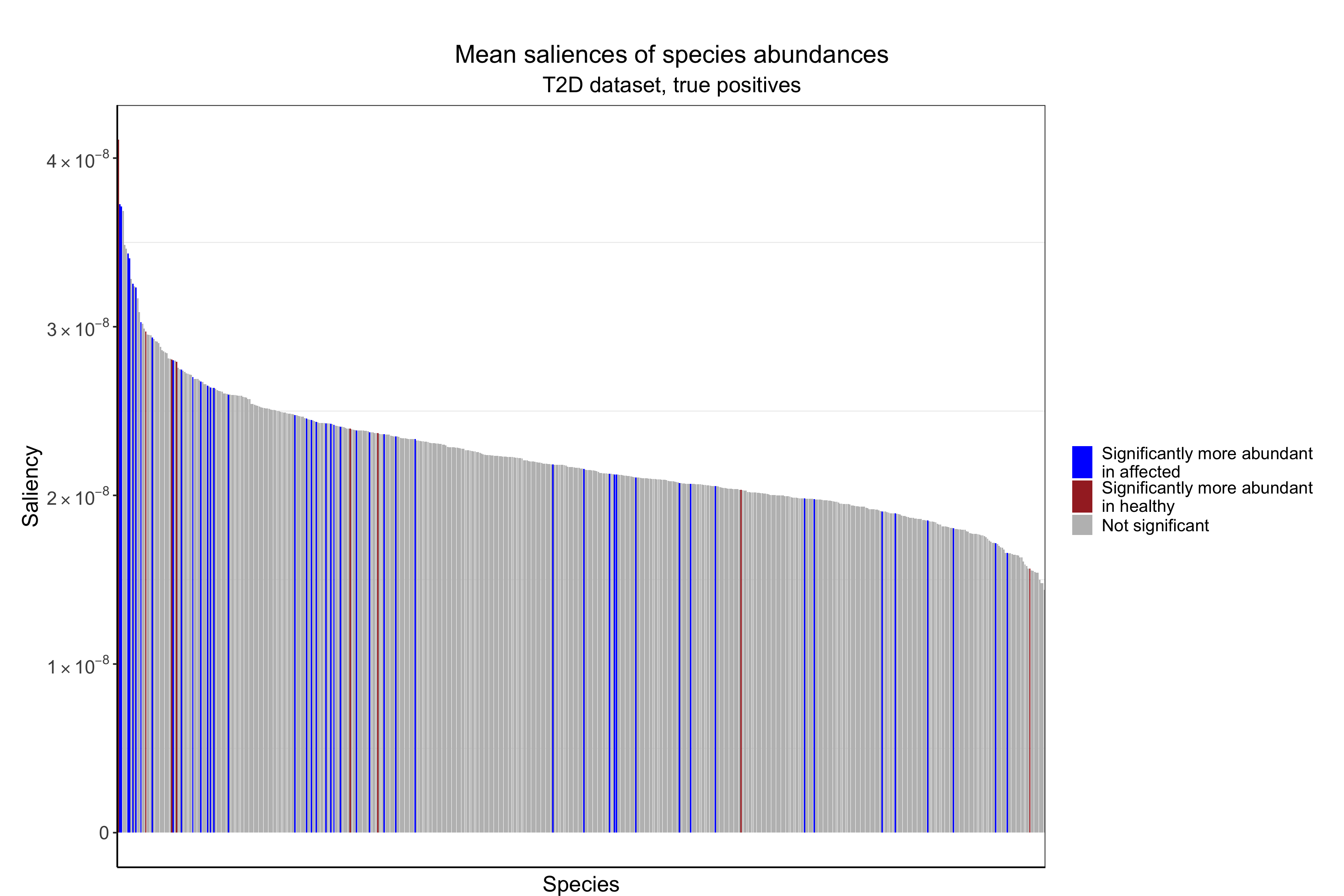

Supplement: S5 File — For each dataset, two different kinds of plots are available. (A) the histogram of the average saliency distribution over microbial species. Species are sorted from left to right by decreasing saliency. Species abundance significance in healthy (red) and affected (blue) individuals was calculated using a Wilcoxon test for each microbial species for two unpaired samples: healthy and affected individuals. (B) violin plots of the saliency distributions for microbial species grouped by significance: significantly more abundant in affected (blue), significantly more abundant in healthy (red), no significance (grey). (ZIP) [file pcbi.1010050.s010.zip › s9-file/T2D/abundance_barplot_TP_saliences_no_rescale_pval-0.1.png]

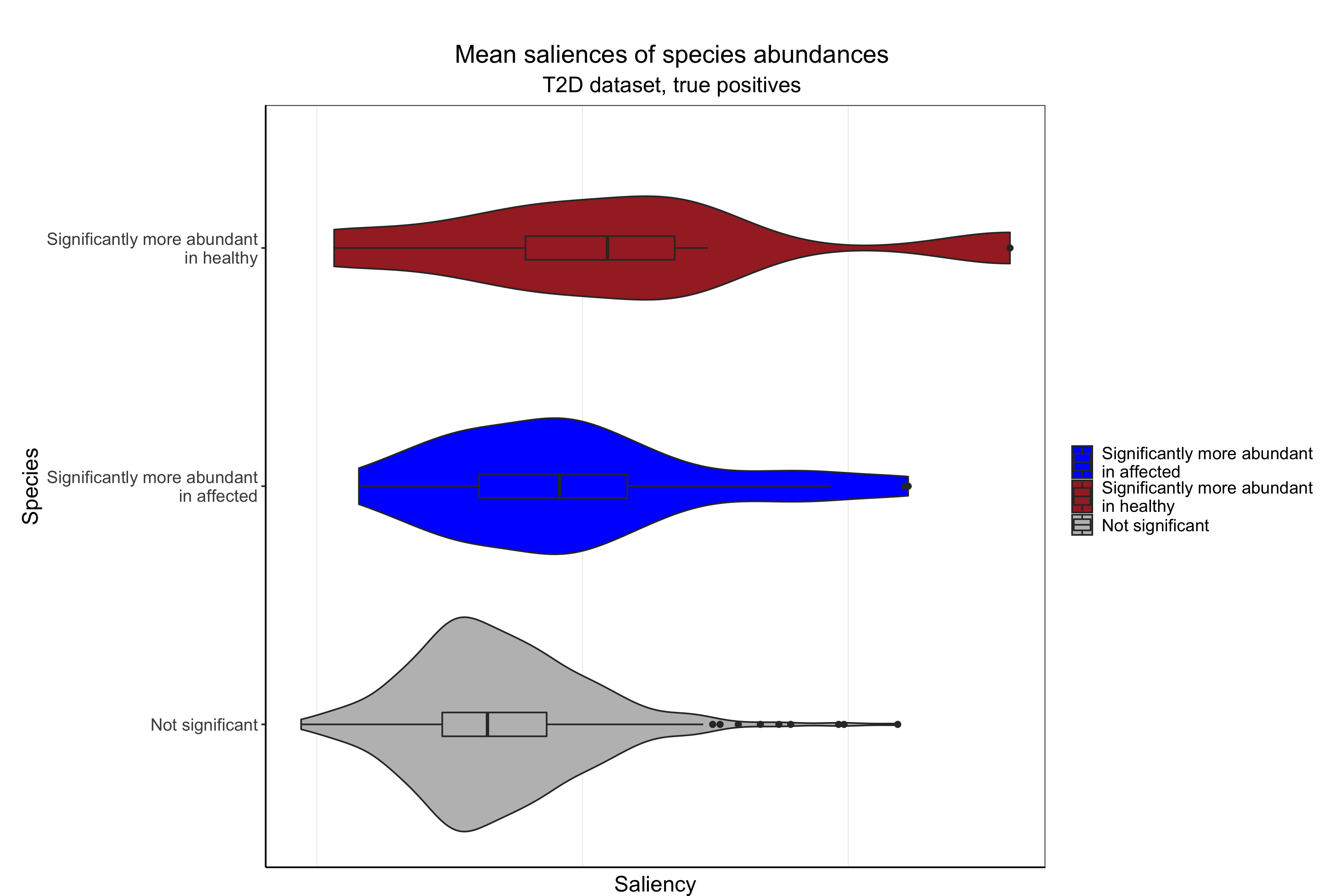

Supplement: S5 File — For each dataset, two different kinds of plots are available. (A) the histogram of the average saliency distribution over microbial species. Species are sorted from left to right by decreasing saliency. Species abundance significance in healthy (red) and affected (blue) individuals was calculated using a Wilcoxon test for each microbial species for two unpaired samples: healthy and affected individuals. (B) violin plots of the saliency distributions for microbial species grouped by significance: significantly more abundant in affected (blue), significantly more abundant in healthy (red), no significance (grey). (ZIP) [file pcbi.1010050.s010.zip › s9-file/T2D/abundance_violin_TP_saliences_no_rescale_pval-0.1.png]

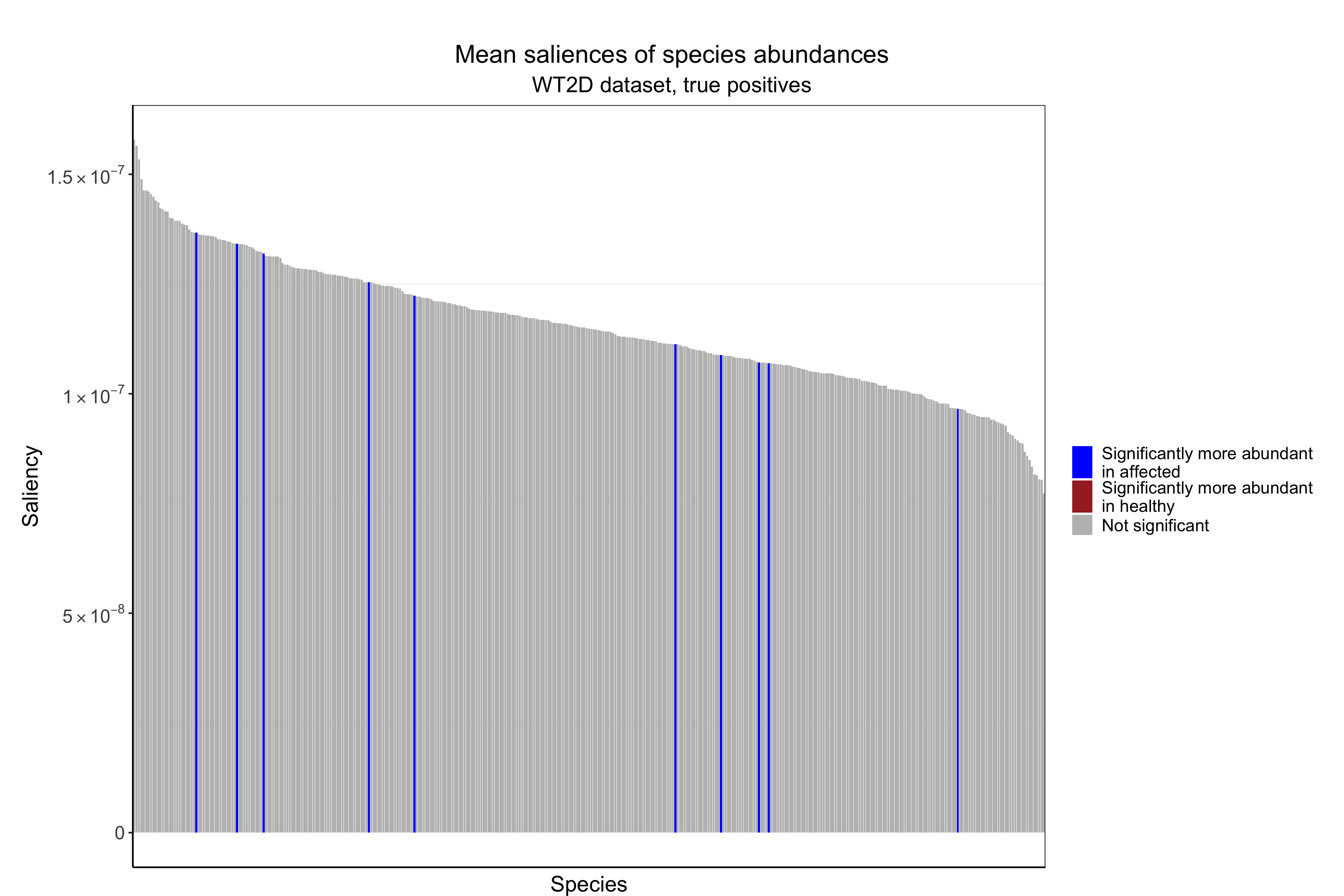

Supplement: S5 File — For each dataset, two different kinds of plots are available. (A) the histogram of the average saliency distribution over microbial species. Species are sorted from left to right by decreasing saliency. Species abundance significance in healthy (red) and affected (blue) individuals was calculated using a Wilcoxon test for each microbial species for two unpaired samples: healthy and affected individuals. (B) violin plots of the saliency distributions for microbial species grouped by significance: significantly more abundant in affected (blue), significantly more abundant in healthy (red), no significance (grey). (ZIP) [file pcbi.1010050.s010.zip › s9-file/WT2D/abundance_barplot_TP_saliences_no_rescale_pval-0.1.png]

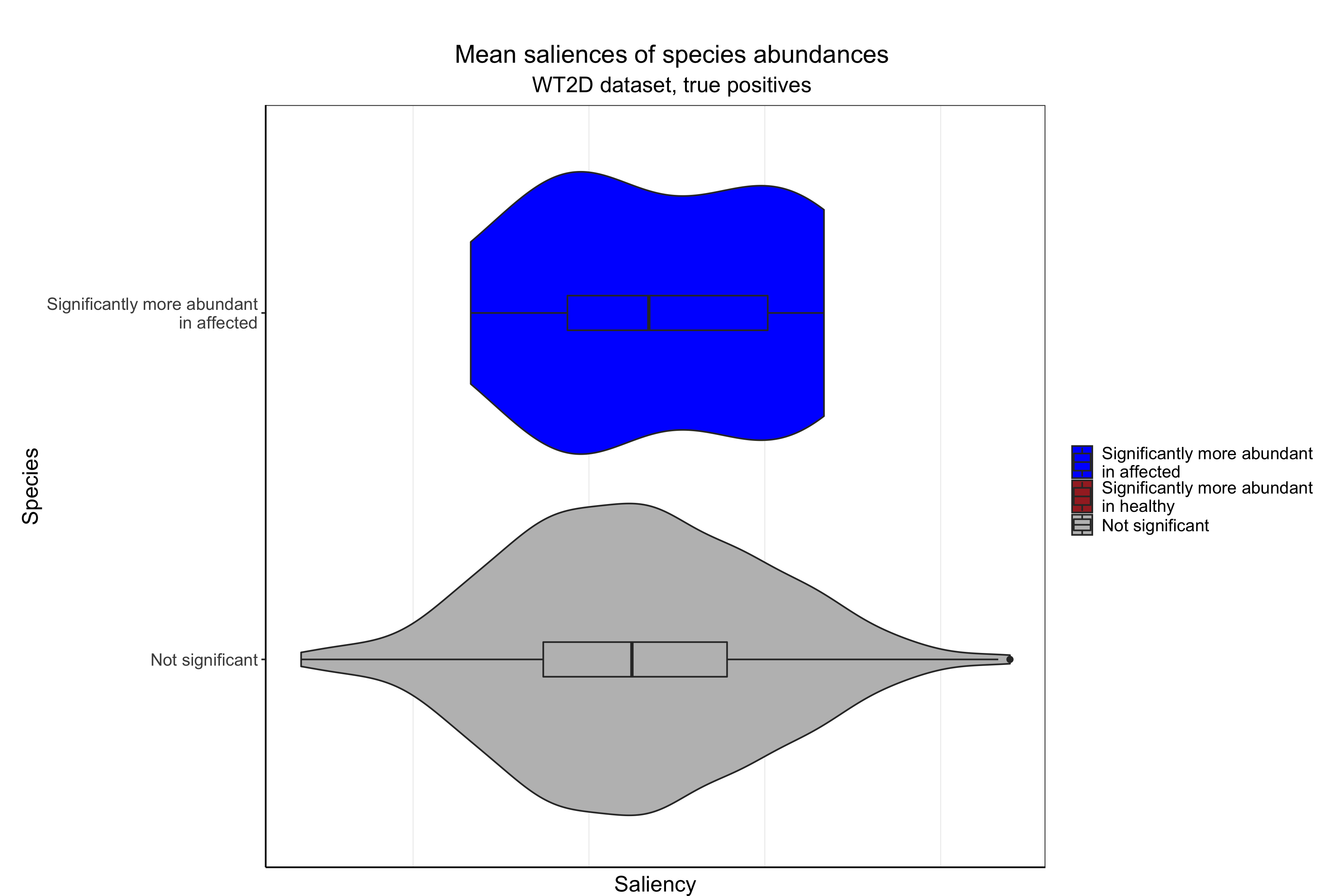

Supplement: S5 File — For each dataset, two different kinds of plots are available. (A) the histogram of the average saliency distribution over microbial species. Species are sorted from left to right by decreasing saliency. Species abundance significance in healthy (red) and affected (blue) individuals was calculated using a Wilcoxon test for each microbial species for two unpaired samples: healthy and affected individuals. (B) violin plots of the saliency distributions for microbial species grouped by significance: significantly more abundant in affected (blue), significantly more abundant in healthy (red), no significance (grey). (ZIP) [file pcbi.1010050.s010.zip › s9-file/WT2D/abundance_violin_TP_saliences_no_rescale_pval-0.1.png]
